# Genomic Organization, Molecular Diversification, and Evolution of Antimicrobial Peptide Myticin-C Genes in the Mussel (*Mytilus galloprovincialis*)

**DOI:** 10.1371/journal.pone.0024041

**Published:** 2011-08-31

**Authors:** Manuel Vera, Paulino Martínez, Laura Poisa-Beiro, Antonio Figueras, Beatriz Novoa

**Affiliations:** 1 Departamento de Genética, Facultad de Veterinaria. Universidad de Santiago de Compostela, Lugo, Spain; 2 Instituto de Investigaciones Marinas, CSIC, Vigo, Spain; UMDNJ-New Jersey Medical School, United States of America

## Abstract

Myticin-C is a highly variable antimicrobial peptide associated to immune response in Mediterranean mussel (*Mytilus galloprovincialis*). In this study, we tried to ascertain the genetic organization and the mechanisms underlying myticin-C variation and evolution of this gene family. We took advantage of the large intron size variation to find out the number of myticin-C genes. Using fragment analysis a maximum of four alleles was detected per individual at both introns in a large mussel sample suggesting a minimum of two myticin-C genes. The transmission pattern of size variants in two full-sib families was also used to ascertain the number of myticin-C genes underlying the variability observed. Results in both families were in accordance with two myticin-C genes organized in tandem. A more detailed analysis of myticin-C variation was carried out by sequencing a large sample of complementary (cDNA) and genomic DNA (gDNA) in 10 individuals. Two basic sequences were detected at most individuals and several sequences were constituted by combination of two different basic sequences, strongly suggesting somatic recombination or gene conversion. Slight within-basic sequence variation detected in all individuals was attributed to somatic mutation. Such mutations were more frequently at the C-terminal domain and mostly determined non-synonymous substitutions. The mature peptide domain showed the highest variation both in the whole cDNA and in the basic-sequence samples, which is in accordance with the pathogen recognition function associated to this domain. Although most tests suggested neutrality for myticin-C variation, evidence indicated positive selection in the mature peptide and C-terminal region. Three main highly supported clusters were observed when reconstructing phylogeny on basic sequences, meiotic recombination playing a relevant role on myticin-C evolution. This study demonstrates that mechanisms to generate molecular variation similar to that observed in vertebrates are also operating in molluscs.

## Introduction

Invertebrates are a heterogeneous group of animals which constitute the huge majority of the surviving animal phyla. Interestingly, only one of about 35 known animal phyla includes vertebrates. Usually the environments where invertebrates dwell are abundant on potentially pathogenic microorganims. Although in recent years there have been advances in the knowledge of invertebrate immunity, a comprehensive view of the immune mechanisms deployed across the broad spectrum of invertebrate phyla [1] is not available. One recurrent question is how these animals survive without an acquired immune system. In particular, marine invertebrates, such as bivalves, are in contact with all sort of potential pathogens such as viruses, bacteria and parasites due to their filtering activities. Mussels (ex: *Mytilus galloprovincialis*) present a high filtering activity: one adult mussel can filter roughly eight liters of water in one hour [2]–[4], which implies that they are in intimate contact with a wide variety of microorganisms.

A key element of the immune system is the discrimination between self and non-self. This implies, especially in complex pluricellular organisms, a molecular code to provide the singularity of each individual, whose molecular basis is particularly well known within vertebrates [5]. On the other hand, recognition of non-self can be achieved by identifying pathogen-associated molecular patterns (PAMPs), mostly related to innate immunity, or by detecting foreign (non-self) molecules, characteristic of the adaptive immune response [6], [7]. Therefore, generation of molecular diversity is essential for some key elements of the immune system and different strategies have been developed along evolution for molecular diversification. The primary mechanisms are related to the exploitation of some genome properties such as recombination, mutation, alternative splicing and exon shuffling on specific genes which require high diversity to fulfill their function. These mechanisms act mainly in the somatic cell line, while germline cells maintain these genes unaltered through generations and are only subjected to the general processes of genome variation [1], [8], [9]. Other proposed mechanisms are related to interaction of different molecules which can promote variability taking advantage of the high combinatory of different elements (synergism); to changes in the amount of specific molecules by gene duplication; and to variation at regulatory elements (dosage) [9].

Generation of genetic diversity of vertebrate immunoglobulin constitutes one of the best studied processes of the immune system, and it involves both intragenic recombination and hypermutation [10], [11]. The high allelic variation of the Major Histocompatibility Complex (MHC) genes and the main evolutionary forces driven it, is also well documented [12], [13]. Within invertebrates, high molecular diversity has been reported at Dscam (related to the immunoglobulin superfamily) in *Drosophila* due to alternative splicing leading to more than 30.000 different isoforms [14], and at fibrinogen related proteins (FREPs) in the snail *Biomphalaria glabrata* relying on somatic mutation and recombination mechanisms [1]. However, further studies are needed to increase knowledge on invertebrates immunity, and particularly, for understanding the mechanisms responsible of molecular diversification.

**Figure 1:**
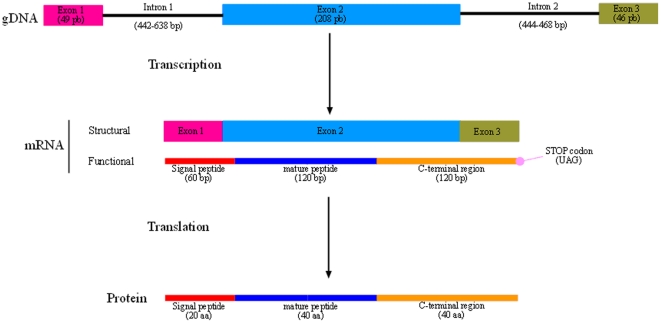
Structure and functional domains of myticin-C gene from Mediterranean mussel (*Mytilus galloprovincialis*).

Antimicrobial peptides (AMPs) are peptides of small size which promote efficient binding to structural components of microorganisms, facilitating their elimination through different effector mechanisms in a wide variety of organisms [15]. The presence of AMP isoforms has been reported to be correlated with an improved defense against pathogens in well established AMP systems such as defensins and cathelicidins [16], [17]. AMPs can act as modifiers of innate and adaptive immune response [18]. Further, AMPs synthesis within insects has demonstrated to be activated through Tumour Necrosis Factor (TNF) receptor and Toll Like-Receptor (TLR) molecules, following similar pathways to mammals [19]. Within molluscs an important variety of AMPs has been reported, including defensins, mytilins, myticins and mytimycins [20], [21]. The high variation observed in some AMP families has been attributed to the high copy number at specific gene families [22]. Myticin-C is a highly expressed AMP during Mediterranean mussel (*Mytilus galloprovincialis*) diseases [23], which shows a typical AMP structure including signal peptide, mature peptide and C-terminal region domains (Figure 1). High sequence variability has been reported at myticin-C, suggesting that this wide repertoire of sequences may be related to the high disease resistance observed in Mediterranean mussel (*Mytilus galloprovincialis*) [24]. Remarkably, this AMP variability was not observed in libraries from other bivalves [25]–[32]. Recently, we have demonstrated that myticin-C presents antiviral activity against two different fish viruses (enveloped and non-enveloped) and that is able to modulate the mussel immune response by modifying the expression of mussel immune-related genes and attracting hemocytes [33].

The Mediterranean mussel is a species of great relevance in aquaculture with a world production above million Tons [34]. Mortalities are frequent in bivalves, but mussels do not seem to be susceptible to the same pathogens responsible of massive deaths of other molluscs. By their sesile character and resistance, mussels are used as a model to monitor pollution in oceans all over the world [35]. Although these animals are being cultured extensively, we still far from understand how they react against pathogens. In this work, we have addressed the study of the genomic organization of myticin-C genes taking advantage of the high variability described at their introns. For this purpose, intraindividual and intrapopulation genetic diversity for introns 1 and 2 were studied in natural populations and the pattern of genetic transmission analyzed in full-sib families. Besides, we investigated the mechanisms that may explain the high genetic diversity reported for myticin-C by analyzing and comparing a large sample of high quality transcriptomic and genomic sequences from several individuals. Using this information, we evaluated the role of selection on the evolution of myticin-C.

## Results

### Genetic diversity of myticin-C introns 1 and 2 in natural populations

**Figure 2:**
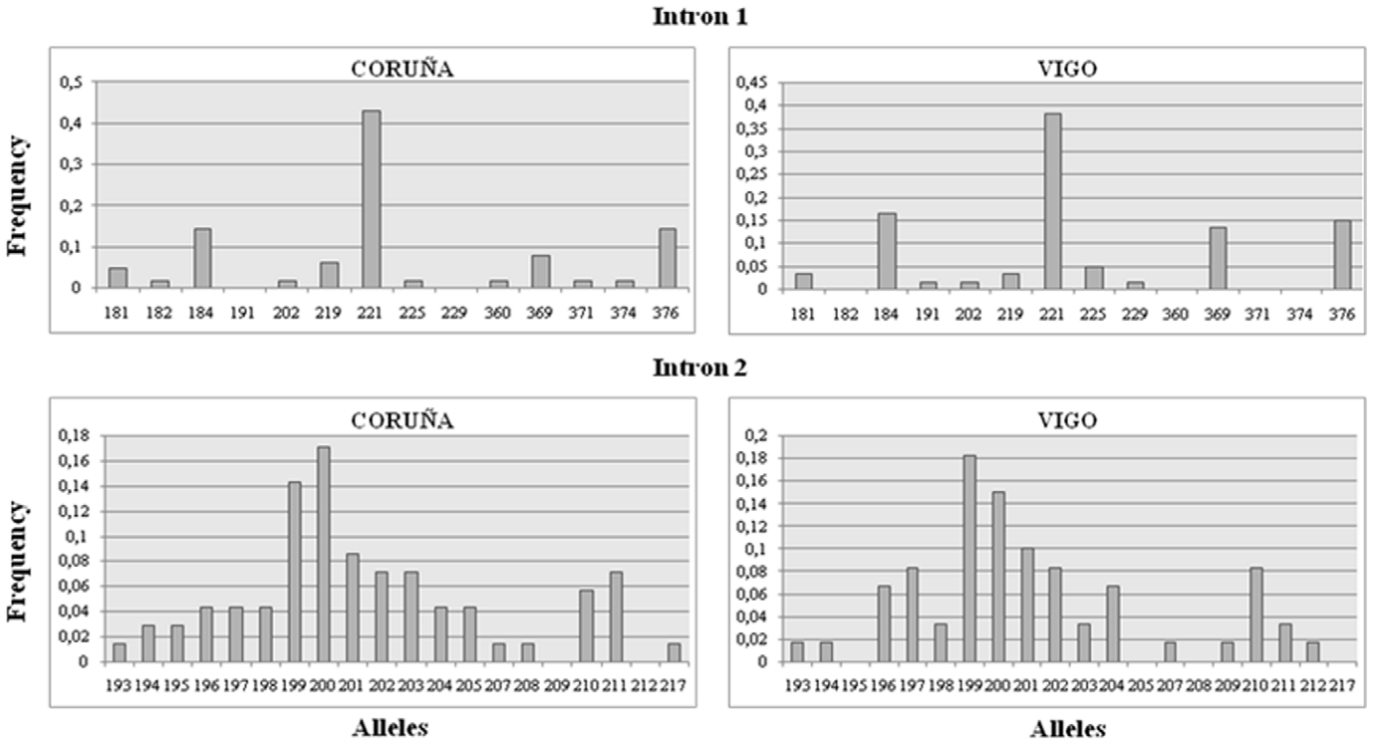
Allelic frequency distribution (in base pairs) at myticin-C introns 1 and 2 in two populations from NW Spain (Coruña and Vigo).

Two mussel samples from NW Spain were studied to evaluate genetic variability of myticin-C at individual and population levels. Myticin-C introns 1 and 2 were chosen for this analysis because of the high length variability previously reported at these gene regions [23]. Accordingly, gDNA fragment analysis was performed to reveal their variability. We expected that this analysis provided new information on myticin-C variation in natural populations and some insights into its evolution, but especially it should be useful to infer the minimum number of genes underlying the variation observed. Up to four peaks (alleles) per individual were detected for both introns in the whole sample: from 1 to 4 alleles at intron1 in both populations and at intron 2 in Coruña; and from 1 to 3 alleles at intron 2 in Vigo. This suggests the existence of at least two myticin-C loci. Genetic variation of myticin-C was higher at intron 2 than at intron 1 in both populations, despite the larger allelic range of intron 1. Three main modes were observed in allelic frequency distributions for intron 1 (184, 221 and 376) and two for intron 2 (199/200 and 210/211) in both populations (Figure 2). Genetic diversity was very similar in both populations: Vigo (Intron 1: A = 12, gene diversity = 0.779; Intron 2: A = 16; gene diversity = 0.899); Coruña (Intron 1: A = 12, gene diversity = 0.761; Intron 2: A = 18; gene diversity = 0.913). No significant differences were observed for allele frequency distributions between Coruña and Vigo populations for both introns (Wilcoxon-Mann-Whitney test intron 1: Z = −0.258, P = 0.796; intron 2: Z = −0.657; P = 0.511).

### Mendelian segregation of myticin-C introns 1 and 2 size variants

**Figure 3:**
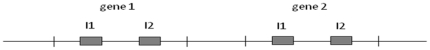
Hypothesis on genomic architecture of myticin-C genes from familiar and population fragment analysis data of intron 1 and intron 2.

As expected for introns of the same gene, full genotypic disequilibrium was observed between intron 1 and intron 2 in both families (Table 1). Thus, in the first family, the allele 376 of intron 1 in the father was transmitted always linked to allele 211 of intron 2, while allele 223 was transmitted linked to allele 202. Offspring genotypes adjusted to 1∶1∶1∶1 proportions in both families for both introns (family 1: χ^2^ = 1.842; P = 0.606; family 2: χ^2^ = 1.759; P = 0.624). These are the expected proportions under a single locus segregation hypothesis, when both parents are heterozygous for different alleles. However, one parent in both crosses exhibited more than two alleles in both introns and segregation between them was not at random. In fact, alleles 221/223 from the mother in the first cross and alleles 221/372 from the father in the second cross were always transmitted jointly as a single Mendelian unit at intron 1. The same occurred at intron 2, where alleles 199/201 from the mother in the first cross and alleles 201/205 from the father in the second cross were transmitted together. The existence of two 201 alleles in the father of the second cross was inferred by the 200/201/205 offspring detected and confirmed by the roughly double height of the 201 peak in the father and in the 201/201/205 offspring. The most plausible explanation for these observations is the existence of two myticin-C closely linked genes arranged in tandem (Figure 3).

**Table 1:** Inheritance of myticin-C intron size variants in *M. galloprovincialis*.

Family 1	Intron 1	Intron 2	Family 2	Intron 1	Intron 2
Father	223/376	202/211	Father	218/221/372	201/201/205
Mother	221/223/369	198/199/201	Mother	219/362	201/201

Frequency	Offspring genotypes		Frequency	Offspring genotypes	
4	221/223	199/201/202	6	218/219	200/201
7	221/223/376	199/201/211	7	219/221/372	200/201/205
3	223/369	198/202	9	218/362	201/201
5	369/376	198/211	7	221/372/362	201/201/205

### Transcriptomic variation of myticin-C

**Figure 4:**
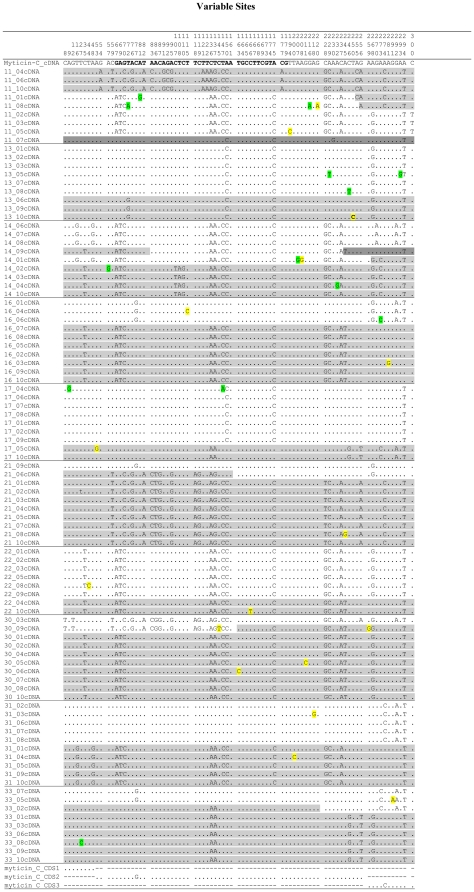
Variable positions of myticin-C cDNA in 10 mussels from Coruña natural population. In white, gray or dark gray background, the different basic sequences identified at each individual. Single nucleotide variants within basic sequences highlighted in green (synonymous) and yellow (non-synonymous). The sequence AM497977 from Genebank was included for reference in the analysis.

Ninety three high quality cDNA sequences from 10 *M. galloprovincialis* individuals (GenBank Accession numbers = JF990711–JF990804) were finally selected among the 100 sequences obtained (10 individuals×10 cDNA sequences) to study genetic variation of myticin-C at transcriptomic level (Figure 4). Between two and, more occasionally, three highly divergent cDNA sequences were observed within each individual (marked with different background color in Figure 4). Small differences were observed within each of these sequences due to single nucleotide substitutions (singletons), and in some cases, sequences appeared to be constituted by combination of two of the aforementioned highly divergent sequences. As explained below, all data point toward somatic mutation and recombination to explain the differences observed within these highly divergent cDNA sequences. Thus, we defined basic sequences as those divergent cDNA sequences existing in each individual excluding singletons and/or recombination events.

Two different basic sequences were identified at most individuals, and only two mussels showed either three basic cDNA sequences (individual 11) or evidences of a third one (individual 14). Slight differences were detected among each one of these basic sequences within individuals mostly due to single nucleotide substitutions: 80.0% sequences showed one; 15% two; and 1% three. A total of 30 substitutions were detected in the whole cDNA sample (all unique), mostly representing singletons (86.7%) and the remaining four being new nucleotide variants at extant variable sites (13.3%). Three of these recurrent mutation sites were located in a highly polymorphic region (between nucleotides 229 and 246; 35.3% variable sites) that could include mutation hotspot sites. These observations suggest that variation within each basic sequence is a consequence of somatic point mutation mechanisms. Mutation rate per site would be 1.1*10^−3^ in the 93 sequences analyzed. Somatic mutations were non-randomly distributed according to the myticin-C polypeptide structure. Thus, the mature peptide showed the lowest proportion of mutants (7/120 = 0.058), while the signal peptide (0.083) and, especially the C-terminal region (0.150), displayed a higher proportion. A high percentage of these point mutations (56.7%) constituted non-synonymous variants giving rise to aminoacid substitutions, mostly affecting the C-terminal region (64.7%).

A second relevant feature of transcriptomic analysis of myticin-C was the evidence of recombination events at some individuals reflected by the presence of new sequences arising as combination of basic cDNA sequences (Figure 4). Five individuals out of ten analyzed showed one or two recombinants at the 9–10 sequences analyzed per individual. Recombinants were the result of the combination of two basic sequences, but in one case apparently three basic sequences could be involved (sequence 14_09). The average rate of recombination per individual was 0.064±0.023.

A total of 21 basic sequences (excluding single nucleotide variants and recombinants, as defined above) were detected among the 93 cDNA sequences studied.

### Genomic variation of myticin-C

To compare the features observed at transcriptome level and to get new insights into the genetic basis of myticin-C variation, we analyzed 88 high quality forward and reverse myticin-C gDNA sequences in eight individuals (between 8–15 per individual; GenBank Accession numbers = JF990616–JF990710) previously evaluated for cDNA variation (Fig. S1). The pattern of myticin-C gDNA variation was similar to that observed at cDNA. Between one and three basic gDNA sequences (defined as for cDNA sequences) were observed in each individual with slight differences within basic sequences due to one or two presumed point mutations. Basic gDNA and cDNA sequences at each individual were identical, although only one basic gDNA sequence was detected in individuals 16 and 17, while they showed two different basic cDNAs. Point mutation rate at gDNA (9.1*10^−4^) was slightly lower than that observed in cDNA analysis and only 1 out of the 21 mutations detected at gDNA was also observed in their correspondent cDNAs. Finally, it was remarkable that no recombinant sequences were detected at gDNA, when around six should be expected according to recombination rate observed at cDNA.

**Figure 5:**
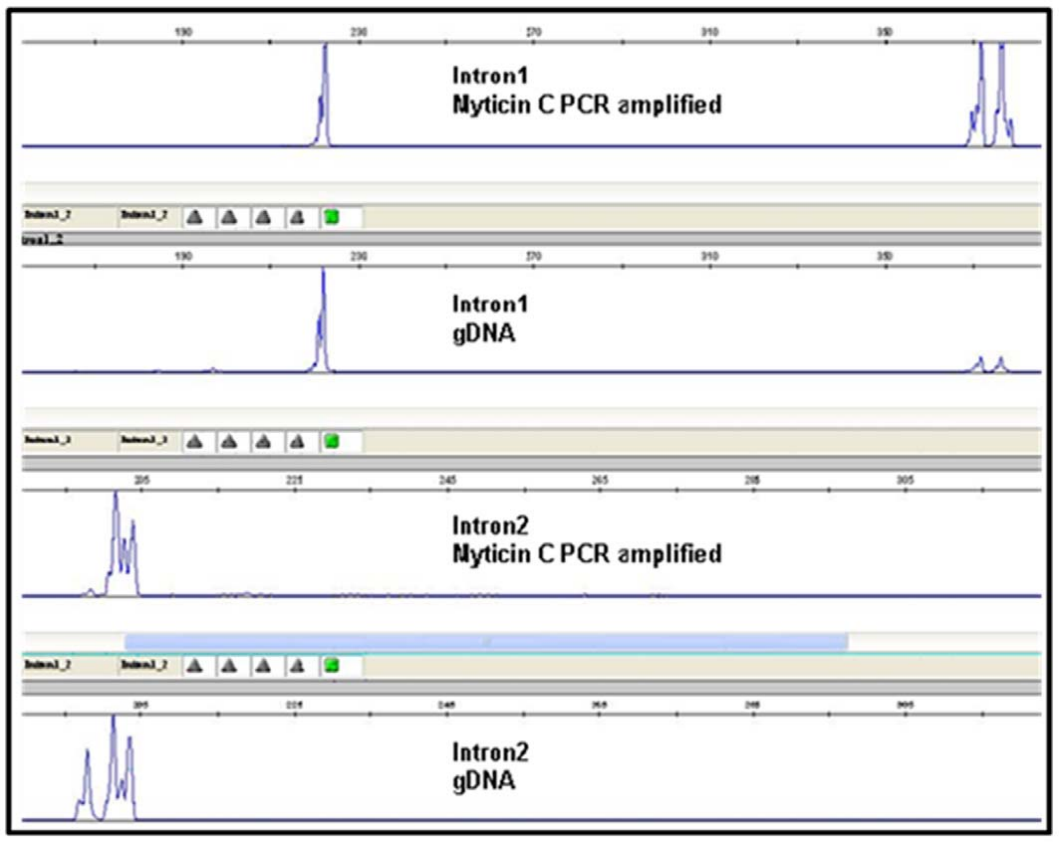
Comparison of fragment analysis in the same individual for intron 1 and intron 2 starting from myticin-C amplified genes and from total DNA.

Analysis of gDNA also enabled us a more detailed evaluation of variation at intronic regions and its comparison with fragment analysis data. Firstly, the three and two allelic modes observed, respectively, at intron 1 and intron 2 fragment analysis distributions (Figure 2), were explained by the presence of two (35 and 155 bp) and one (10 bp) large indel/s at introns 1 and 2, respectively (Fig. S1). Minor and less frequent indels (between 9–24 bp) and variable single mononucleotide repetitions (poli A, poli C, but especially poli T) gave account for size variation around these main modes. Second, excluding indels, nucleotide variation at both introns was higher than that observed at exons (intron 1: segregating sites (S) = 35.1%; haplotype diversity (Hd) = 0.973, and Waterson's estimator of nucleotide diversity based on the proportion of segregating sites (θ_W_) = 0.07759; intron 2: S = 33.6%; Hd = 0.987, and θ_W_ = 0.07554), although exon 2 showed diversity figures very close to both introns (Tables 2 and 3). Third, similar variation to that described for cDNA basic sequences attributed to somatic mutations was observed within basic sequences at both introns. Fourth, some discordance was detected between fragment analysis and sequencing at gDNA. Thus, some length variants observed in the fragment analysis were not detected in the gDNA sequencing in several individuals. It appeared like some myticin-C genes showed low or no amplification with the primers used. To check this possibility, we repeated the fragment analysis in these individuals but using a two-step PCR. We first amplified myticin-C genes from total gDNA material, and then used this DNA in a second step using specific primers to amplify introns 1 and 2. If there were differences in amplification of the two hypothesized myticin-C genes, we should observe differences in their correspondent introns 1 and 2 fragments in comparison with fragment analysis starting from total DNA. Results showed that, indeed, some fragments observed in our previous fragment analysis (starting from total DNA) were missed when amplified myticin-C gDNA was used to perform intron PCRs (Figure 5). This strongly suggests that one of the two hypothesized myticin-C genes could be under-amplified, probably due to mismatches at the primer template regions.

**Table 2:** Genetic diversity pattern at myticin-C in *M. galloprovincialis* with all cDNA sequences.

Summary statistics	Signal peptide	Mature peptide	C-terminal region	All regions
N	94	94	94	94
Sites	60	120	120	300
S	12 (5, 7)	31 (5, 26)	29 (15, 14)	72 (25,47)
π	0.02137 (0.00216)	0.05415 (0.00393)	0.03769 (0.00140)	0.04119 (0.00202)
θ_W_	0.03910 (0.01448)	0.05009 (0.01495)	0.04724 (0.01422)	0,04692 (0.01258)
D_Tajima_	−1.2148 (*p>0.10*)	−0.1321 (*p>0.10*)	−0.9911 (*p>0.10*)	−0.7214 (*p>0.10*)
D_Fu and Li_	−1.6291 (*p>0.10*)	−0.0244 (*p>0.10*)	−3.3483 (*p<0.02*)	−2.0354 (*0.10>p>0.05*)
H-test				0.0000 (*p = 0.3265*)
ω (Ka/Ks)	0.948	1.027	2.771	1.275
M8-M8a LRT (Selection)	Non Significant	*p<0.05*	*p<0.001*	*p<0.001*

The sequence AM497977 from Genebank was included in the analysis. N: number of cDNA sequences; S: number of segregating site (in parentheses singletons and parsimony informative sites, respectively); π: average number of nucleotide differences per site; θ_W_: nucleotide diversity based on the proportion of segregating sites; ω: ratio (Ka/Ks) between non-synonymous substitutions (Ka) and synonymous substitutions (Ks). M8-M8a LRT: likelihood ratio test among the model M8 and M8a to evaluate positive selection.

**Table 3:** Genetic diversity pattern at myticin-C in *M. galloprovincialis* with basic cDNA sequences.

Summary statistics	Signal peptide	Mature peptide	C-terminal region	All regions
n	22	22	22	22
Sites	60	120	120	300
S	7 (3, 4)	26 (10, 16)	15 (4, 11)	48 (17,31)
π	0.02056 (0.00510)	0.05725 (0.00821)	0.03723 (0.00270)	0.04190 (0.00456)
θ_W_	0.03200 (0.01551)	0.05944 (0.02237)	0.03429 (0.01401)	0,04389 (0.01558)
D_Tajima_	−1.1440 (*p>0.10*)	−0.4034 (*p>0.10*)	−0.1536 (*p>0.10*)	−0.4692 (*p>0.10*)
D_Fu and Li_	−0.6353 (*p>0.10*)	−0.6093 (*p>0.10*)	−0.0364 (*p>0.10*)	−0.4749 (*p>0.10*)
H-test				*0.0000 (p = 0.3246)*
ω (Ka/Ks)	0.858	1.004	3.068	1.302
M8-M8a LRT (Selection)	Non Significant	*p<0.05*	*p<0.05*	*p<0.001*

Abbreviations correspond to those indicated in Table 2.

### Evolution of myticin-C

All the 93 cDNA sequences analyzed and the 21 basic sequences detected in the transcriptomic analysis were used to analyze the evolutionary pattern of myticin-C genes. Estimators of genetic diversity confirmed the high genetic variation in the whole cDNA sample (24.0% variable sites; π = 0.04119; θ_W_ = 0.04692; Table 2) and in the basic sequences (16.0%; π = 0.04190 θ_W_ = 0.04389; Table 3). Basic cDNA sequences showed a lower proportion of variable sites (16%) than whole cDNA sequences (24%) because somatic mutations were excluded by definition in their composition. However, average number of nucleotide differences per site (nucleotide diversity, π) and nucleotide diversity based on the proportion of segregating sites (θ_W_) were very similar in both cDNA samples probably because the higher proportion of variable sites in the whole cDNA sample was counterbalanced by the repetition of basic sequences.

Dissection of genetic diversity according to the different regions of myticin-C protein revealed that mature peptide displayed higher genetic diversity than C-terminal region and signal peptide. The difference was even higher in the basic sequences than in the whole cDNA sample. The higher proportion of singletons in the whole cDNA sample at the C-terminal region and even at the signal peptide reflects the higher impact of somatic point mutation at these regions regarding the mature peptide, as outlined before. However, a large proportion of singletons in mature peptide were detected in the basic sample indicating the higher evolutionary diversification of this domain. Most neutrality tests were not significant in the whole peptide and when applied to the specific domains of myticin-C, thus suggesting no effects of selection on the variation observed. Only the Fu and Li test for the whole cDNA sample resulted significant (D = −3.3483, *p<0.*02), thus suggesting purifying selection. However, the ratio between non-synonymous *vs* synonymous variation was higher than 1 and significant under a M8-M8a model at mature peptide and C-terminal region both in the whole cDNA sample and in the basic cDNA sequences. This suggests that, although variation is neutral at most myticin-C nucleotide sites, especially at signal peptide, positive selection could be occurring at some regions of mature peptide and C-terminal region, thus rendering global significant tests.

**Figure 6:**
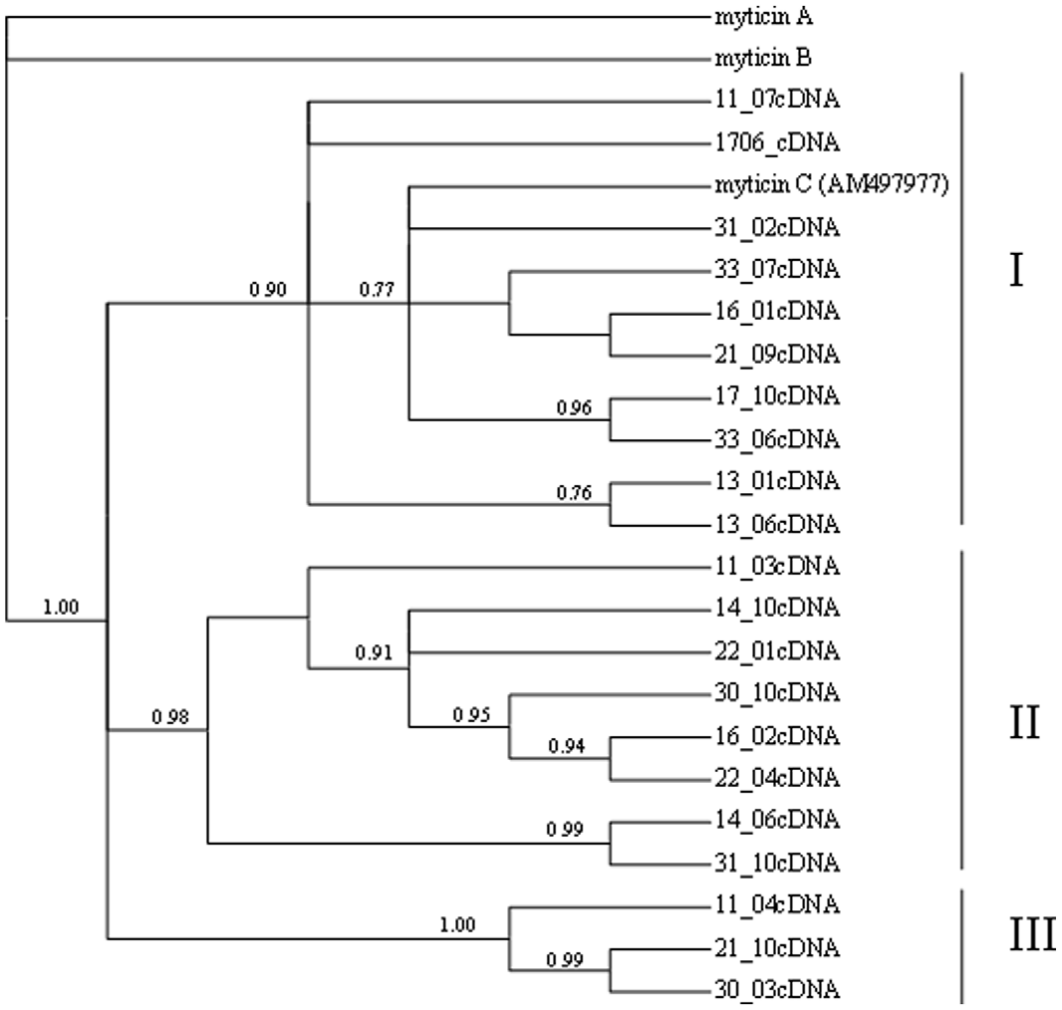
Bayesian tree of basic cDNA sequences of myticin-C. Values on branches indicate Bayesian posterior probability (only values >0.75 are showed). Tree was rooted using Myticin A (AF162334) and myticin B (AF1623354) sequences as outgroups.

A phylogenetic tree was constructed using a Bayesian method implemented in MrBayes 3.1.2 program to analyze phylogenetic relationships of the 21 basic myticin-C cDNA sequences (Figure 6). Three highly supported clades with bootstrap values close to 100 were identified. The two sister groups I and II were much more diversified than the III one. Visual inspection of basic sequences suggested that a major recombination event could have occurred in the origin of these three main groups (Figure S2). In fact, five recombination events were estimated for all sequences using Dnasp V5.0, evidencing the role of recombination on the evolution of myticin-C genes.

## Discussion

### Genomic organization of myticin-C

High nucleotide variability was previously reported for myticin-C introns 1 and 2 by sequence analysis [23] and DGGE electrophoresis [24]. The variation detected with DGGE was so high, that DGGE patterns were unique for each mussel irrespective of its sex or origin and only full-sibs shared common bands in electrophoretic profiles [24]. In our study, we confirmed this high variability using both fragment analysis and gDNA sequencing and demonstrated that large indels are on the basis of the main size variants at both introns. Accordingly, two large indels at intron 1 would explain the three main size variants (445, 480 and 615 bp) reported at intronic regions [23]. As reported by these authors [23] and suggested by the DGGE analysis [24], we also identified minor indels and a large amount of single nucleotide substitutions scattered along introns sequences. No differences in allelic size frequency were detected between the two natural populations analyzed at intron 1 and intron 2, which agrees with the very low genetic structure of Mediterranean mussel observed previously with microsatellites in this area (F_ST_ = 0.0122) [36].

The number of the FREP (Fibrinogen-related proteins) genes, a family of highly variable hemolymph lectins involved in non-self recognition in *B. glabrata* [37], was investigated by using Southern blot analysis [1]. In our study, we took advantage of the high variation at introns to ascertain the number of genes of myticin-C and their genomic organization in Mediterranean mussel. Pallavicini et al. [23] suggested that a single gene could account for myticin-C variation considering that only two clusters were identified at each individual after analyzing genetic relationships in a large amount of cDNA clones. However, the detection in our study of up to four size variants at specific individuals for both introns supports the existence of at least two genes. The family analysis performed strongly suggested the existence of two closely linked genes that could be the consequence of a tandem duplication. Costa et al. [24] also analyzed the pattern of inheritance of DGGE myticin-C variants and observed shared banding patterns between full-sibs, but they could not clearly trace back the patterns observed from offspring to parents. On the other hand, the analysis of a large sample of cDNA clones in ten mussels in our study showed that more than two basic variants were present at specific individuals, confirming the necessity of at least two myticin-C genes to explain intraindividual variation. This result is coherent with the identification of three different genomic clones in a single mussel [24]. All data indicate the existence of two genes tandemly organized in Mediterranean mussel underlying the high molecular diversity observed at myticin-C. This result contrasts with the very high copy number detected in other AMP families in molluscs (defensines: 48 copies; prolin-rich: 13 copies) [22], and highlights that the molecular variability required for pathogen recognition and elimination by AMPs in molluscs may follow different diversification strategies.

Despite two myticin-C genes are hypothesized in our work, only two cDNA or gDNA variants were detected in most individuals, a third variant being detected in some individuals but a very low frequency in the clones analyzed. The comparison of fragment analysis with gDNA sequencing showed that some allelic variants detected in the fragment analysis at specific individuals were missed in the gDNA sequencing analysis, despite that in some individuals were sequenced up to 15 clones. The most likely explanation for these apparent discrepancies is the lack of correct matching of myticin-C primers in one of the two hypothesized genes. Specific peaks in the fragment analysis were missed when amplified myticin-C genes were used as raw DNA material for intron PCR amplifications, thus supporting this explanation.

According to the existence of two loci and the high size variability observed at both introns (He between 0.8 and 0.9), a high frequency of double heterozygous individuals (four different alleles) should be expected in mussels. This would be particularly stressed if both loci showed similar variability at introns and despite the probable gametic disequilibrium occurring at these loci since their close linkage. However, only 6.8% and 3.2% individuals showed four alleles at intron 1 in Vigo and Coruña, respectively, while 60.7% and 57.9% should be expected according to allelic frequencies. The same occurred at intron 2, where 0% and 3.0% double heterozygotes were observed in the same populations, while 80.8% and 83.3% should be expected. This is an expectable result if the hypothesized myticin-C duplication had taken place recently and mutation would have no time enough for increasing genetic variability in the new locus. Alternatively, it is possible that duplication is not fixed in mussel populations and a segregating polymorphism exists, individuals showing between two and four alleles.

### Mechanisms underlying myticin-C diversification

Several AMP families demonstrated high genetic variability at coding regions in invertebrate [9], [22], [38] and vertebrate species [39]. In the Mediterranean mussel, Pallavicini et al. [20] demonstrated much higher variability in myticin-C than in other AMP families. These authors also reported that other non immune-related housekeeping genes, such as β-actine, showed much lower genetic diversity and only two single nucleotide variants were detected among the 33 sequences analyzed, thus suggesting diversification mechanisms specifically acting on myticin-C genes. The study by Pallavicini et al. [23] and that by Costa et al. [24] were conducted to describe myticin-C variability, rather than to find out the underlying mechanisms of such variation. In our study, we analyzed a large sample of cDNA and gDNA sequences to ascertain the mechanisms responsible of transcriptomic and genomic myticin-C diversification, trying to balance the two sources of sampling variance, individuals and clones within individuals. A key point to address this type of studies is to manage high quality sequences to ensure the confidence of the variants detected. Thus, all clones in our study were sequenced from both 5′ and 3′ ends and only high quality sequences were considered for further analysis. As previously reported [23], a few basic sequences (2–3) were identified at each individual both at cDNA and gDNA in our work, which agrees with the low number of myticin-C genes hypothesized. However, frequent single nucleotide differences were detected among the different copies of each basic sequence, both in cDNA and gDNA analysis, strongly suggesting an origin due to somatic mutation. A similar mechanism of somatic variation was reported in FREP peptides in the snail *B. glabrata* [1]. In accordance with its random mutation origin, most of these variants represented singletons in the whole sample and only a few nucleotide variants were detected at extant variable sites. These appeared at highly variable regions that could represent mutation hotspots, as reported in vertebrate immunoglobulins [40], [41]. Global somatic mutation rate (1.1*10^−3^) was in the upper range of that described in mouse IgH immunoglobulins (from 10^−3^ to 10^−5^) [42]. Somatic mutations affected in a similar fashion to both introns and exons and, despite some hotspots mutation sites occurred, the whole myticin-C gene appeared under their influence. However, somatic mutations seemed to be unevenly distributed among the myticin-C domains and a higher rate was observed especially at the C-terminal domain, which nearly doubled that observed at mature peptide. This observation should be confirmed in a larger sample, and suggests a different impact of somatic mutation along myticin-C gene that may be related to different functional constraints at its different domains. Remarkably, point mutation sites within each individual showed large divergence between gDNA and cDNA sequences. This could indicate that the mechanism of somatic mutation occurs along all the life of the individual, thus determining large intercellular differences within individuals. The observation of myticin-C expression at different adult tissues (mantle, digestive gland and haemolymph) and even at larval stages and ovocites by Costa et al. [24] supports this explanation. Also, mRNA editing or the existence of specific molecular processes at mRNA or mRNA intermediaries could contribute to the process of diversification explaining the differences observed between cDNA and gDNA sequences within individuals. A more detailed study on myticin-C diversification along mussel ontogeny using large cDNA and gDNA samples in a few individuals would be required to discern between these hypotheses.

Some myticin-C cDNA sequences appeared to be constituted by pieces of two different basic sequences, strongly suggesting crossing-over or gene conversion events occurring at somatic tissues. Recombinant sequences were also identified by Zhang et al. [1] in *B. glabrata* when analyzing the causes of FREPs diversification. Thus, apparently, somatic recombination could also be occuring in Mediterranean mussel to generate molecular variability at myticin-C genes. The average rate of recombination per individual was not too high (0.064±0.023), but half individuals showed at least one recombinant in the sample of clones analyzed. The role of somatic recombination in the genesis of variation in vertebrate immunoglobulin is well known [43], [44]. On the contrary, no role for recombination was suggested to explain variation in other AMPs like in the amphibian *Bombina maxima* [45]. In Mediterranean mussel, remarkably, no recombination signs were detected in the gDNA sample, when a certain proportion should be expected according to recombination rate at cDNA. This may be a result of the short cDNA and gDNA sample size regarding the low frequency of recombination, but also some molecular mechanism specifically acting on mRNA cannot be discarded.

### Evolution of myticin-C

The evolution of AMP gene families has been addressed in different species [22], [45]. In the Mediterranean mussel, a phylogenetic analysis was carried out starting from large samples of cDNA sequences [23], [46]. However, these authors included all genetic variants detected in their cDNA sample. According to our results, much of this variation is of somatic origin and therefore not subjected to evolutionary agents. In our study, we split the analysis using on one hand all cDNA sequences and on the other only the 21 basic sequences identified in the 10 individuals analyzed. The mature peptide domain showed the highest nucleotide diversity among the three myticin-C domains both in the whole cDNA sample and in the basic cDNA sequences, which highlights the relevance of molecular diversification at this myticin-C domain, directly related to pathogen recognition. However, the C-terminal region showed the highest impact of somatic mutation (nearly twice than mature peptide), suggesting the relevance of random mutation on this likely intracellular domain of unknown function. The signal peptide domain was the least variable one in accordance with its membrane recognition function for transference of immature myticin-C into the reticulum endoplasmic for further processing. Our results contrast with that found by other authors [23], who reported a higher variability at the C-terminal region. This may be explained by the inclusion of somatic variants in their study that, as outlined above, showed a higher impact on C-terminal region. Padhi and Verghese [46] found that some myticin-C variable codons could be subjected to positive selection despite purifying selection would explain the pattern at most variable sites. According to these authors, we detected evidences of positive selection in the mature peptide domain, likely indicating selective pressures of pathogen diversity determining genetic diversification at this region. Considering the high intrinsic variability observed at mature peptide, the signals of positive selection at this domain should be more probably related to balancing selection than to directional one. Sound signals of balancing selection have been reported in other immune-genes related to pathogen recognition or individual self-recognition [47]. Higher conservation of signal peptide than mature peptide in Cg-defensines was also reported in *Crassostrea gigas*, several sites being under positive selection [22]. However, the highest evidence of positive selection was detected at the C-terminal, a domain apparently not directly related to pathogen recognition. Further investigation on the functional role of C-terminal domain will be crucial to interpret these observations.

Unlike previous phylogenetic studies on myticin-C [23], [46], we focused our phylogenetic analysis on the basic cDNA sequences eliminating the noise produced by somatic mutation. Our results depicted a much clearer picture of myticin-C phylogeny, three highly supported clusters being identified. Contrary to other authors [46], we also found strong evidences of meiotic recombination in the evolution of myticin-C genes, as previously reported in defensins for *C. gigas* [22].

## Materials and Methods

All experiments in this work have been reviewed and approved by the CSIC National Committee on Bioethics. Molluscs care and experiments were conducted according the CSIC National Committee on Bioethics guidelines under approval number ID 10.02.11.

### Fragment analysis of myticin-C intron 1 and intron 2

Genomic DNA from mussel mantle was extracted with DNAzol (Invitrogen) following the manufacturer's instructions. Specific amplifications of myticin-C introns 1 and 2 were conducted using primer pairs designed using Primer 3 software [48] (Intron 1: F: 5′ AAAAATACGGCAGACCGACTT 3′, R: GGAGCTGAACAACACGTATAAA 3′; Intron 2: F: 5′ TGTCGTTATCGTCTGAATCG 3′, R: GTGCAATTGACCAGTGATCG 3′). Forward primers were labeled with fluorescein (Invitrogen) for fragment analysis. The PCR reaction was performed with a high fidelity Taq polymerase (TaKaRa ExTaq™ Hot Start Version; TaKaRa Bio Inc., Otsu, Siga, Japan), and the cycling protocol was 94°C for 5 min, 40 cycles of 94°C for 30 s, 55°C for 1 min and 72°C for 1 min followed by a final extension of 72°C for 7 min. PCR products were analyzed in an ABI PRISM® 3730xl automatic sequencer (Applied Biosystems, Foster City, CA). Allele scoring was resolved with GeneMapper 4.0 software (Applied Biosystems, Foster City, CA). The GeneScan-500 LIZ was used as internal size standard.

#### Population analysis

Two mussel samples of 30 individuals each were collected in NW Spain to analyze myticin-C variability in individuals and populations from the wild. Samples were taken in an area where previous studies on genetic structure had been carried out using microsatellites [36], thus providing a neutral reference to which contrasts our results. Within this area, we selected two rather distant locations to gather as much variation as possible according to the goals pursued in our study. Identification of allelic variants (genotypes) at each individual in the analyzed populations was carried out considering the peaks detected in the fragment analysis, because the number of myticin-C genes in mussel is unknown. Additionally, the existence of more than one gene would preclude the assignment of detected alleles to each specific locus. This procedure probably introduces a slight bias in allele frequency estimation because heterozygous individuals (two peaks) contribute more than homozygous (one peak) to the whole allele number in the sample. Thus the frequency of the most frequent allelic variants, more represented in homozygous individuals according to Hardy-Weinberg expectations, could be slightly underestimated. Genotypes were used to construct allelic frequency distributions and to estimate genetic diversity. The number of alleles (A) and gene diversity were used as estimators of genetic diversity in populations [49]. The number of alleles was used as an estimation of intraindividual genetic diversity. Allelic distributions of intron 1 and intron 2 were compared between populations and non-parametric Wilcoxon-Mann-Whitney tests were used to check for the significance of genetic differentiation between populations.

#### Family analysis: pattern of transmission

Two full-sib families (19 and 29 offspring, respectively) with known parents were used to analyze the pattern of inheritance of myticin-C variants in order to obtain information about the number and organization of myticin-C genes. Mature mussels were taken out of seawater and placed in individual containers at room temperature. After 1 h, spawning was induced by a sudden thermal shock using flowing seawater at 23°C [50]. Gametes were released after 2 h of stimulation. Eggs derived from a single female were fertilized by sperm from a single male. Gametes were gently stirred in a sterile glass beaker to suspend the eggs and sperm to facilitate fertilization. One hour after fertilization, embryos were placed into 150 L tanks at a density of 2 larvae/ml. Larvae were reared on a mixed diet of *Isochrysis galbana* and *Phaeodactylum tricornutum*. Water was changed every two days by sieving the larvae with screens ranging from 40 to 100 µm according to the different size of the larvae. All fertilization steps and rearing were performed in 1 µm-filtered seawater (FSW) at 23°C and aerated under air bubbling. Isolation of DNA and intron analysis was performed in each individual at 5 months age as explained before. Mendelian segregation at introns 1 and 2 was checked using chi-square tests (P<0.05).

### Analysis of genomic and transcriptomic variation: genetic mechanisms on the origin of myticin-C variability

To establish the mechanism underlying myticin-C variation, we first evaluated a large sample of complementary DNA (cDNA) sequences from 10 individuals pertaining to Coruña natural population. Ten cDNA sequences were analyzed per individual. Also, genomic DNA (gDNA) was analyzed in eight of these same individuals, to confirm the features observed at cDNA level and to get further information on the mechanisms underlying myticin-C variation. Between 10 and 20 gDNA sequences were obtained per individual.

Total RNA was isolated from mussel hemocytes using Trizol reagent following the manufacturer instructions, resuspended in DEPC-treated water and stored at −80°C. The quality of total RNA was confirmed by agarose gel electrophoresis. After DNaseI treatment, 1 µg of total RNA was used to obtain cDNA by the SuperScript III first-strand synthesis supermix (Invitrogen). Myticin-C cDNA and gDNA were amplified using the same specific primers: Myticin 7F-F (5′ ATATTCCTCAAAACTCAAAACATTCA 3′) and Myticin 7F-R (5′ TTCAAGCTGAAAACGTCGAA 3′). PCR products from individual mussels were subsequently cloned into the pCR2.1-TOPO vector (Invitrogen) using DH5α™ Competent Cells (Invitrogen). Blue/white screening of colonies was performed on plates containing X-gal. White colonies were screened by PCR using M13 vector-specific primers. Finally, positive clones were sequenced using the same primers.

All sequences analysed in this study, either gDNA or cDNA, were obtained following the ABI Prism BigDye™ Terminator v3.1 Cycle Sequencing Kit protocol on an ABI 3730 DNA sequencer (Applied Biosystems, Foster City, CA, USA). Forward and reverse sequences were obtained in all cases for accuracy. Alignment of the different sequences was carried out using the program ClustalX 2.0 [51] taking as reference the sequences EU927441 and AM497977 to align gDNA and cDNA sequences, respectively. We used default parameters for gap opening ( = 15) and gap extension costs ( = 6.66). Variable sites were checked by hand with the program SeqScape 2.5 (Applied Biosystems, Foster City, CA). Due to the presence of large indels in the intronic regions, BioEdit v. 7.0 [52] was used to include these important gaps in the genomic alignment created by Clustal program. This enabled us to compare fragment length variation at both introns with genomic sequences within each individual. BioEdit was also used to align the genomic and cDNA sequences at each individual. Haplotypes were identified using the program Mega 4.0 [53].

### Evolution of myticin-C

Myticin-C variation at coding region was analyzed considering the whole cDNA and the three functional peptide domains: signal peptide, mature peptide and the anionic C-terminal region [21], [46]. Polymorphism values (polymorphic sites, number of singletons and parsimony informative sites) and nucleotide variability (π and θ_W_) were used for estimating genetic diversity at myticin-C using DnaSP V. 5.0 [54]. cDNA variation was contrasted with that of gDNA considering both coding region and introns. To check the effect of selection on myticin-C variability, several neutrality tests were applied (Tajima's D and Fu and Li D) using the same program. Also, the ratio of non-synonymous substitutions (Ka) and synonymous substitutions (Ks) for each codon (Ka/Ks = ω) was calculated with Selecton web server (freely available on http://selecton.tau.ac.il/index.html), based on M8 evolutionary model which allows to check for positive selection. Statistical significance was assessed using the likelihood ratio test (LRT) which compares the log likelihood of M8 model to the log likelihood of M8a alternative model. This allows checking for negative or neutral selection models. Detection of variable sites and translation of the coding regions to amino acid sequences was performed using MEGA v 4.0 [53].

To analyze phylogenetic relationships of myticin-C variants, a Bayesian Inference (BI) tree was constructed applying the best nucleotide substitution model using the basic cDNA sequences (see Results section) and using myticin-A and myticin-B sequences as outgroups (GenBank Accession Numbers: AF162334 and AF162335, respectively). MrModeltest v.2.0 [55] was used to determine the optimal model of nucleotide evolution for the data set following the Akaike Information Criterion (AIC; [56]). The General Time Reversible model with a gamma parameter of 0.9360 and a proportion of invariable sites of 0.5559 (GTR+I+G) was identified as the most appropriate. BI analyses was implemented using MrBayes 3.1.2 [57], [58]. The Metropolis-Coupled Markov Chain Monte Carlo process was run for 1 cold and 3 hot chains and 1,000,000 generations, with trees being sampled every 100 generations for a total of 10,000 trees in the initial sample. The minimum number of recombination events in the phylogeny (Rm, [59]) was estimated using DnaSP V. 5.0.

## Supporting Information

Figure S1 **Alignment of all myticin gDNA and cDNA sequences used in this study in fasta format (it is possible to open this file in any alignment program such as BioEdit, Mega, Clustal, DNAsp,…).** All sequences have been aligned using the myticin-C genomic sequence EU927441 as reference. All sequenced individuals belong to Coruña natural population. The first and second numbers of sequence code correspond to individual and clone, respectively. “Gen” and “cDNA” correspond to genomic and coding DNA sequences, respectively. Genomic sequences show both exons and introns whereas coding DNA sequences only show exons. Indels in introns correspond to length polymorphisms. Gaps showed in exons were included to align both types of sequences. Myticin_C_CDS1, myticin_C_CDS2 and myticin_C_CDS3 correspond to exon 1, exon 2 and exon 3, respectively. These last sequences have been included to locate easily the coding regions.
(DOC)

Figure S2 **Variable positions of myticin-C cDNA in the 21 different basic sequences detected.** The sequence AM497977 from Genebank was included for reference in the analysis.
(DOC)
