# Supplementary material for: Genomic Organization, Molecular Diversification, and Evolution of Antimicrobial Peptide Myticin-C Genes in the Mussel (Mytilus galloprovincialis)
Source: PLoS One. 2011 Aug 31;6(8):e24041. doi: 10.1371/journal.pone.0024041 (PMC3164099; doi:10.1371/journal.pone.0024041)
Supplement: Figure S1 — Alignment of all myticin gDNA and cDNA sequences used in this study in fasta format (it is possible to open this file in any alignment program such as BioEdit, Mega, Clustal, DNAsp,…). All sequences have been aligned using the myticin-C genomic sequence EU927441 as reference. All sequenced individuals belong to Coruña natural population. The first and second numbers of sequence code correspond to individual and clone, respectively. “Gen” and “cDNA” correspond to genomic and coding DNA sequences, respectively. Genomic sequences show both exons and introns whereas coding DNA sequences only show exons. Indels in introns correspond to length polymorphisms. Gaps showed in exons were included to align both types of sequences. Myticin_C_CDS1, myticin_C_CDS2 and myticin_C_CDS3 correspond to exon 1, exon 2 and exon 3, respectively. These last sequences have been included to locate easily the coding regions. (DOC) [file pone.0024041.s001.doc]

>MyticinC_Gen_EU927441

ATGAAGGCAACGATCTTGTTAGCTGTTGTAGTGGCAGTCATTGTTGGAGGTAAATATCTT

TATTGAAATATATCTGTGTGTACGTGTTTTAGAAGTATAGATCCAGCATTTTT-AAGATA

AAAATACGGCAGACCGACTTTTCCAAAATTTGATTGCGAATCCCATATT---------TG

TATATTATGTTAAATTATACTACTTAATTGTAATAATTTTTCATTTA---TAATAACTGT

TTTAAGTAAGAAAGCTGTATTTGGCAAAACTTTTAGGAATTTTGGTCCTCAATGCTCTTC

TTTTTT-AACTTTTATGATTCGAGCGTCACTGATGAATCTTTTGTAGACGAAACGCGCGG

CTGGCGTATATTTAATTTAGTTCTGGTATCTATGATGAGTTTATTGTTAATGTTTACAAC

TGAAATATGTTTTGTTGTTAAAATTAAATCACTCAAATACTCAAACAAATATTTTTT-CT

TTATACGTGTTGTTCAGTTCGTAGTTTTCTATGCTATGCTTTGTATACTGTTGTTTTTCT

TTTTTTTTCTCCTTTTTTTT---GCCATGGCGTTGTTAGTTTATTTTCAACTTATGATAA

TAAATGTCCC-TGTGGTATATTT--AGCCTCTCTCTAAGACAGGCATCGTT-ATTTGTGT

CGAAA-CTTAAGCAATTTTATTC-A-TTTCATTTTT-CTAC------TTATTCA--GTTC

AGGAAGCCCAATCAGTAGCTTGTAGATCATACTACTGTAGTAAGTTCTGTGGGTCTGCTG

GTTGCTCATTATATGGATGTTACCTACTTCATCCTGGAAAAATTTGCTACTGCCTTCATT

GTAGCAGAGCTGAGTCTCCATTGGCACTTTCTGGAAGCGCTAGGAATGTGAACGACAAGA

ACAACGAGATGGACAACTCTCCAGTGTAAGTACTCAATACTAGATTTCGTTACACAGTAT

AAACATTTAAAAATTTGGAAAAAAAA------GTTTTCTACATTCCAACATATATTGTAA

TTAAGGTGTTTACCAATTTTTGAAACAGATAATTTGAT-TTTTTTTTAAATAAAATATTT

CATTTT-----AATTCATTATGTCGTTATCGTCTGAATCGTATTA------TGCAGTCAT

TGAAAAAAAA--TAAACACCTAAACTGCTCCTGCAAGAGAAAGAAGGGAT-GT-TTT-TT

-CTAAGATCGGTTAATGTGGTGGGAGTCAGATAAGATATCTTCTAGTGTGTCTCTCAATT

-ATTTACATTCAAAAACATTTTTTGT---ACATAAATCGATCACTGGTCAATTGCACGAG

AATAGTCAGAAAAAAAC---TGTTATATAAAACGTTTTATTTGAATTTGTGAAAAAAAAC

GCCACATATAAGAGAACATCTACTTTGTTTCAGGATGAATGAGATGGAACATTTGGACCA

AGAAATGGAAATGTTCTAG

>11_02Gen

ATGAAGGCAACGATCTTGTTAGCTGTTGTAGTGGCAGTCATTGTTGGAGGTGAATATCTT

TATTGAAATATATCTGTGTGTACGTATTTTAGAAGTATAGATCCAATATTTTTTAAGATA

AAAATACGGCAGACCGACTTTTCCAAAATTTGATTGCGAAACCCATATT---------TG

TATATTATGTAAAATTGTACTACTTTATTGTAATATTTGTTCATTAA---TGAATACTGT

TTTATGTAATATAA----------------------------------------------

------------------------------------------------------------

------------------------------------------ATTGTTAATGTTTACAAC

TGAAATATGTTTTGTTATTAAAATTCAATCATTCAAATATTCAAACAAATATTTTTTTCT

TTATACGTGTTGTTCAGTTTCTAGTTTTCTATGCTATGCTTTGTATACTGTTGTTTGTCT

TTTA--------------------CCATGTCGTTGTCAGTAGATTTTTAACTTATGATAT

CGAATGTCCC-TGTGGTATATTT--TGTCTCTCTTTTAGACAGGCATCGTT-ATTTGTG-

CATTA-TTTAATCAATTTTATTT-AATTTCATTTTTTCTCCGTTTTTCATTTATAAGTTC

AAGAAGTCCAATCAGTACCTTGTGCATCAACCTACTGTGCTAGGTTCTGTGGGTCTGCTG

GTTGCTCAAAATATGGATGTAACAGACTTCATCCCGGCAAAATTTGCTACTGCCTTCATT

GTAGAAGAGCTGAGTCTCCATTGGCACTTTCTGGAAGCGCTAGGAATGTGAACGAGCAGA

ACAAAGAGATGGACAACTCTCCCATGTTAGTAC--AATACAAGACTTCGTTACATAATAT

AAACGTTTACAAATTTACTAAAGAAAAACATTGTATTCTACATGCCAATATATACTGTAT

TCAAGGTATAT--CATTTTTTTAAACAGATCATTTGAATTTTTTTT-AAATAAAGCATTT

CATCTTTGTTTAATTCAATAGGTCGTTATCGTCTGCA-CGTATTA------TTTACAGTC

AT-----------AAACACATGAACTACTCCTGCAAGAGAAGGAAGGAAT-GT-TTT-TT

-TTTATATCGGTTAATGTGTTGGGAGGCAGATAAGATATTTTTTATTGTGTCTCTCAATT

-ATTTACATTCAAAAACATTTTT------ACATAAATCAATCACTGGTTAATTGCACGAG

AATAGTCAGAAAAAAAC---TGTTATATGAAACGTTTTAATTGAATTTGTGAAAAAAAAC

GCCACATATAAGAGACCATCTACTTTGTTTCAGGATGAATGAGATGGAACATTTGGACCA

AGAAATGGATATGTTCTAG

>11_03Gen

ATGAAGGCAACGATCTTGTTAGCTGTTGTAGTGGCAGTCATTGTTGGAGGTAAATATCTT

TATTGAAATATATCTGTGTGTACGTATTTTAGAAGTATAGATCCAATATTTTTTAAGATA

AAAATACGGCAGACCGACTTTTCCAAAATTTGATTGCGAAACCCATATT---------TG

TATATTATGTAAAATTGTACTACTTTATTGTAATATTTGTTCATTAA---TGAATACTGT

TTTATGTAATATAA----------------------------------------------

------------------------------------------------------------

------------------------------------------ATTGTTAATGTTTACAAC

TGAAATATGTTTTGTTATTAAAATTCAATCATTCAAATATTCAAACAAATATTTTTTTCT

TTATACGTGTTGTTCAGTTTCTAGTTTTCTATGCTATGCTTTGTATACTGTTGTTTGTCT

TTTA--------------------CCATGTCGTTGTCAGTAGATTTTTAACTTATGATAT

CGAATGTCCC-TGTGGTATATTT--TGTCTCTCTTTTAGACAGGCATCGTT-ATTTGTG-

CATTA-TTTAATCAATTTTATTT-AATTTCATTTTTTCTCCGTTTTTCATTTATAAGTTC

AAGAAGTCCAATCAGTACCTTGTGCATCAACCTACTGTGCTAGGTTCTGTGGGTCTGCTG

GTTGCTCAAAATATGGATGTTACAGACTTCATCCCGGCAAAATTTGCTACTGCCTTCATT

GTAGAAGAGCTGAGTCTCCATTGGCACTTTCTGGAAGCGCTAGGAATGTGAACGAGCAGA

ACAAAGAGATGGACAACTCTCCCATGTAAGTAC--AATACAAGACTTCGTTACATAATAT

AAACGTTTACAAATTTACTAAAGAAAAACAAAGTATTCTACATGCCAATATATACTGTAT

TCAAGGTATAT--CATTTTTTTAAACAGATCATTTGAATTTTTTTT-AAATAAAGCATTT

CATCTTTGTTTAATTCAATAGGTCGTTATCGTCTGCA-CGTATTA------TTTACAGTC

AT-----------AAACACATGAACTACTCCTGCAAGAGAAGGAAGGAAT-GT-TTT-TT

-TTTATATCGGTTAATGTGTTGGGAGGCAGATAAGATATTTTTTATTGTGTCTCTCAATT

-ATTTACATTCAAAAACATTTTT------ACATAAATCAATCACTGGTTAATTGCACGAG

AATAGTCAGAAAAAAAC---TGTTATATGAAACGTTTTAATTGAATTTGTGAAAAAAAAC

GCCACATATAAGAGACCATCTACTTTGTTTCAGGATGAATGAGATGGAACATTTGGACCA

AGAAATGGATATGTTCTAG

>11_04Gen

ATGAAGGCAACGATCTTGTTAGCTGTTGTAGTGGCAGTCATTGTTGGAGGTAAATATCTT

TATTGAAATATATCTGTGTGTACGTATTTTAGAAGTATAGATCCAATATTTTTTAAGATA

AAAATACGGCAGACCGACTTTTCCAAAATTTGATTGCGAAACCCATATT---------TG

TATATTATGTAAAATTGTACTACTTTATTGTAATATTTGTTCATTAA---TGAATACTGT

TTTATGTAATATAA----------------------------------------------

------------------------------------------------------------

------------------------------------------ATTGTTAATGTTTACAAC

TGAAATATGTTTTGTTATTAAAATTCAATCATTCAAATATTCAAACAAATATTTTTTTCT

TTATACGTGTTGTTCAGTTTCTAGTTTTCTATGCTATGCTTTGTATACTGTTGTTTGTCT

TTTA--------------------CCATGTCGTTGTCAGTAGATTTTTAACTTATGATAT

CGAATGTCCC-TGTGGTATATTT--TGTCTCTCTTTTAGACAGGCATCGTT-ATTTGTG-

CATTA-TTTAATCAATTTTATTT-AATTTCATTTTTTCTCCGTTTTTCATTTATAAGTTC

AAGAAGTCCAATCAGTACCTTGTGCATCAACCTACTGTGCTAGGTTCTGTGGGTCTGCTG

GTTGCTCAAAATATGGATGTTACAGACTTCATCCCGGCAAAATTTGCTACTGCCTTCATT

GTAGAAGAGCTGAGTCTCCATTGGCACTTTCTGGAAGCGCTAGGAATGTGAACGAGCAGA

ACAAAGAGATGGACAACTCTCCCATGTAAGTAC--AATACAAGACTTCGTTACATAATAT

AAACGTTTACAAATTTACTAAAGAAAAACATTGTATTCTACATGCCAATATATACTGTAT

TCAAGGTATAT--CATTTTTTTAAACAGATCATTTGAATTTTTTTT-AAATAAAGCATTT

CATCTTTGTTTAATTCAATAGGTCGTTATCGTCTGCA-CGTATTA------TTTACAGTC

AT-----------AAACACATGAACTACTCCTGCAAGAGAAGGAAGGAAT-GT-TTT-TT

-TTTATATCGGTTAATGTGTTGGGAGGCTGATAAGATATTTTTTATTGTGTCTCTCAATT

-ATTTACATTCAAAAACATTTTT------ACATAAATCAATCACTGGTTAATTGCACGAG

AATAGTCAGAAAAAAAC---TGTTATATGAAACGTTTTAATTGAATTTGTGAAAAAAAAC

GCCACATATAAGAGACCATCTACTTTGTTTCAGGATGAATGAGATGGAACATTTGGACCA

AGAAATGGATATGTTCTAG

>11_06Gen

ATGAAGGCAACGATCTTGTTAGCTGTTGTAGTGGCAGTCATTGTTGGAGGTAAATATCTT

TATTGAAATATATCTGTGTGTACGTATTTTAGAAGTATAGATCCAATATTTTTTAAGATA

AAAATACGGCAGACCGACTTTTCCAAAATTTGATTGCGAAACCCATATT---------TG

TATATTATGTAAAATTGTACTACTTTATCGTAATATTTTTTTATTAA---AAAATACTGT

TTTATGCAAGATAA----------------------------------------------

------------------------------------------------------------

------------------------------------------ATTATTAATGTTTACAAC

TGAAATATGTTTTGTTATTAAAATTCAATCATTCAAATATTCAAACAAATCTTTTTTTCT

TTATACGTGTTGTTCAGTTTGTAGTTTTCCATGCTATGCTTTGTATACTATTGTTTGTCT

TTTA--------------------CCGTGTCGTTGTCAGTAGATTTGTAACTTATGATAA

CGAATGTCCC-TGTGGTATATTT--TGTCTCTCTTTTAGACAGGCATCGTT-ATTTGTG-

CACTA-TTTAATCAATTTTATTTTA-TTTCATTTTTTCTCCGTTTTTTATTTATAAGTTC

AGGAAGTCCAATCAGTACCTTGTGTATCAACCTACTGTGCTAGGTTCTGTGGGTCTGCTG

GTTGCTCATTATATGGATGTTACAGACGTACTTACTGCAAAATGTGCTACTGCCTTCATT

GTAGAAGAGCTGAGTCTCCATTGGCACTTTCTGGAAGCGCTAGGAATGTGAACGAGCAGA

ACAAAGATATTGACAACTCTCCAATGTAAGTAC--AATACACGACTTCGTTACATAATAT

AAACGTTTACAAATTTACAAAAGAAAAACATTGTATTCTACATGCCGACATATACTGTAT

TCAAGGTATAT--CATTTTTTTAAACAGATCATTTGAATTTTTTTTTAAATAAAGCATTT

CATCTTTGTTTAATTCAATAGGTCGTTATCGTCTGCA-CGTATTA------TATACAGTC

AT-----------AAACACATGAACTACTCCTGCAAGAGAAGGAAAGGAT-GT-TTT-TT

-T--ATATTGGTTAATGTGTTGGGAGGCAGATTAGATATTTTTTATTGTGTCTCTCAATT

-ATTTACATCCAAAAACATTTTT------ACATAAATCAATCACTGGTCAATTGCACGAG

AATAGTCAGAAAAAAAC---TGTTATATGAAACGTTTTAATTGAATTTGTGAAAAAAAAC

GCCACATATAAGAGACCATCTACTTTGTTTCAGGATGAATGAGATGGAACATTTGGACCA

AGAAATGGATATGTTCTAG

>11_08Gen

ATGAAGGCAACGATCTTGTTAGCTGTTGTAGTGGCAGTCATTGTTGGAGGTAAATATCTT

TATTGAAATATATCTGTGTGTACGTATTTTAGAAGTATAGATCCAATATTTTTTAAGATA

AAAATACGGCAGACCGACTTTTCCAAAATTTGATTGCGAAACCCATATT---------TG

TATATTATGTAAAATTGTACTACTTTATTGTAATATTTGTTCATTAA---TGAATACTGT

TTTATGTAATATAA----------------------------------------------

------------------------------------------------------------

------------------------------------------ATTGTTAATGTTTACAAC

TGAAATATGTTTTGTTATTAAAATTCAATCATTCAAATATTCAAACAAATATTTTTTTCT

TTATACGTGTTGTTCAGTTTCTAGTTTTCTATGCTATGCTTTGTATACTGTTGTTTGTCT

TTTA--------------------CCATGTCGTTGTCAGTAGATTTTTAACTTATGATAT

CGAATGTCCC-TGTGGTATATTT--TGTCTCTCTTTTAGACAGGCATCGTT-ATTTGTG-

CATTA-TTTAATCAATTTTATTT-AATTTCATTTTTTCTCCGTTTTTCATTTATAAGTTC

AAGAAGTCCAATCAGTACCTTGTGCATCAACCTACTGTGCTAGGTTCTGTGGGTCTGCTG

GTTGCTCAAAATATGGATGTTACAGACTTCATCCCGGCAAAATTTGCTACTGCCTTCATT

GTAGAAGAGCTCAGTCTCCATTGGCACTTTCTGGAAGCGCTAGGAATGTGAACGAGCAGA

ACAAAGAGATGGACAACTCTCCCATGTAAGTAC--AATACAAGACTTCGTTACATAATAT

AAACGTTTACAAATTTACTAAAGAAAAACATTGTATTCTACATGCCAATATATACTGTAT

TCAAGGTATAT--CATTTTTTTAAACAGATCATTTGAATTTTTTTT-AAATAAAGCATTT

CATCTTTGTTTAATTCAATAGGTCGTTATCGTCTGCA-CGTATTA------TTTACAGTC

AT-----------AAACACATGAACTACTCCTGCAAGAGAAGGAAGGAAT-GT-TTT-TT

-TTTATATCGGTTAATGTGTTGGGAGGCAGATAAGATATTTTTTATTGTGTCTCTCAATT

-ATTTACATTCAAAAACATTTTT------ACATAAATCAATCACTGGTTAATTGCACGAG

AATAGTCAGAAAAAAAC---TGTTATATGAAACGTTTTAATTGAATTTGTGAAAAAAAAC

GCCACATATAAGAGACCATCTACTTTGTTTCAGGATGAATGAGATGGAACATTTGGACCA

AGAAATGGATATGTTCTAG

>11_10Gen

ATGAAGGCAACGATCTTGTTAGCTGTTGTAGTGGCAGTCATTGTTGGAGGTAAATATCTT

TATTGAAGTATATCTGTGTGTACGTATTTTAGAAGTATAGATCCAATATTTTTTAAGATA

AAAATACGGCAGACCGACTTTTCCAAAATTTGATTGCGAAACCCATATT---------TG

TATATTATGTAAAATTGTACTACTTTATCGTAATATTTTTTTATTAA---AAAATACTGT

TTTATGCAAGATAA----------------------------------------------

------------------------------------------------------------

------------------------------------------ATTATTAATGTTTACAAC

TGAAATATGTTTTGTTATTAAAATTCAATCATTCAAATATTCAAACAAATCTTTTTTTCT

TTATACGTGTTGTTCAGTTTGTAGTTTTCTATGCTATGCTTTGTATACTATTGTTTGTCT

TTTA--------------------CCGTGTCGTTGTCAGTAGATTTGTAACTTATGATAA

CGAATGTCCC-TGTGGTATATTT--TGTCTCTCTTTTAGACAGGCATCGTT-ATTTGTG-

CACTA-TTTAATCAATTTTATTTTA-TTTCATTTTTTCTCCGTTTTTTATTTATAAGTTC

AGGAAGTCCAATCAGTACCTTGTGTATCAACCTACTGTGCTAGGTTCTGTGGGTCTGCTG

GTTGCTCATTATATGGATGTTACAGACGTACTTACTGCAAAATGTGCTACTGCCTTCATT

GTAGAAGAGCTGAGTCTCCATTGGCACTTTCTGGAAGCGCTAGGAATGTGAACGAGCAGA

ACAAAGATATTGACAACTCTCCAATGTAAGTAC--AATACACGACTTCGTTACATAATAT

AAACGTTTACAAATTTACAAAAGAAAAACATTGTATTCTACATGCCGACATATACTGTAT

TCAAGGTATAT--CATTTTTTTAAACAGATCATTTGAATTTTTTTTTAAATAAAGCATTT

CATCTTTGTTTAATTCAATAGGTCGTTATCGTCTGCA-CGTATTA------TATACAGTC

AT-----------AAACACATGAACTACTCCTGCAAGAGAAGGAAAGGAT-GT-TTT-TT

-T--ATATTGGTTAATGTGTTGGGAGGCAGATTAGATATTTTTTATTGTGTCTCTCAATT

-ATTTACATTCAAAAACATTTTT------ACATAAATCAATCACTGGTCAATTGCACGAG

AATAGTCAGAAAAAAAC---TGTTATATGAAACGTTTTAATTGAATTTGTGAAAAAAAAC

GCCACATATAAGAGACCATCTACTTTGTTTCAGGATGAATGAGATGGAACATTTGGACCA

AGAAATGGATATGTTCTAG

>11_11Gen

ATGAAGGCAACGATCTTGTTAGCTGTTGTAGTGGCAGTCATTGTTGGAGGTAAATATCTT

TATTGAAGTATATCTGTGTGTACGTATTTTAGAAGTATAGATCCAATATTTTTTAAGATA

AAAATACGGCAGACCGACTTTTCCAAAATTTGATTGCGAAACCCATATT---------TG

TATATTATGTAAAATTGTACTACTTTATCGTAATATTTTTTTATTAA---AAAATACTGT

TTTATGCAAGATAA----------------------------------------------

------------------------------------------------------------

------------------------------------------ATTATTAATGTTTACAAC

TGAAATATGTTTTGTTATTAAAATTCAATCATTCAAATATTCAAACAAATCTTTTTT-CT

TTATACGTGTTGTTCAGTTTGTAGTTTTCTATGCTATGCTTTGTATACTATTGTTTGTCT

TTTA--------------------CCGTGTCGTTGTCAGTAGATTTGTAACTTATGATAA

CGAATGTCCC-TGTGGTATATTT--TGTCTCTCTTTTAGACAGGCATCGTT-ATTTGTG-

CACTA-TTTAATCAATTTTATTTTA-TTTCATTTTTTCTCCGTTTTTTATTTATAAGTTC

AGGAAGTCCAATCAGTACCTTGTGTATCAACCTACTGTGCTAGGTTCTGTGGGTCTGCTG

GTTGCTCATTATATGGATGTTACAGACGTACTTACTGCAAAATGTGCTACTGCCTTCATT

GTAGAAGAGCTGAGTCTCCATTGGCACTTTCTGGAAGCGCTAGGAATGTGAACGAGCAGA

ACAAAGATATTGACAACTCTCCAATGTAAGTAC--AATACACGACTTCGTTACATAATAT

AAACGTTTACAAATTTACAAAAGAAAAACATTGTATTCTACATGCCGACATATACTGTAT

TCAAGGTATAT--CATTTTTTTAAACAGATCATTTGAATTTTTTTTTAAATAAAGCATTT

CATCTTTGTTTAATTCAATAGGTCGTTATCGTCTGCA-CGTATTA------TATACAGTC

AT-----------AAACACATGAACTACTCCTGCAAGAGAAGGAAAGGAT-GT-TTT-TT

-T--ATATTGGTTAATGTGTTGGGAGGCAGATTAGATATTTTTTATTGTGTCTCTCAATT

-ATTTACATTCAAAAACATTTTT------ACATAAATCAATCACTGGTCAATTGCACGAG

AATAGTCAGAAAAAAAC---TGTTATATGAAACGTTTTAATTGAATTTGTGAAAAAAAAC

GCCACATATAAGAGACCATCTACTTTGTTTCAGGATGAATGAGATGGAACATTTGGACCA

AGAAATGGATATGTTCTAG

>11_12Gen

ATGAAGGCAACGATCTTGTTAGCTGTTGTAGTGGCAGTCATTGTTGGAGGTAAATATCTT

TATTGAAATATATCTGTGTGTACGTATTTTAGAAGTATAGATCCAGCATTTTT-AAGATA

AAAATACGGCAGACCGACTTCTCCAAAATTTGATTGCGAATCACATATTGTTAATTTATG

TATTATGTTAAATTATACTACTTCATTGTAATAATTTTTCATTTATA-----ATAACTGT

TTTAAGTAAGAAAGCTGTATTTGGCAAAACTTTTAGGAATTTTGGTCCTCAATGCTCTTC

TTTTTT-AACTTTTATGATTCGAGCGTCACTGATGAGTCTTTTGTAGACGAAACGCGCGT

CTGGCGTATACGTGATTTAGTTCTGGTATCTATGATGAGTTTATTGTTAATGTTTACAAC

TGAAATATGTTTTGTTATTAAAATTAAATCACTCAAATACTCAAACAAATATTTTTTTCT

TTATACGTGTTGTTCAGTTCGTTGTTTTCTATGCTATGCTTTGTATACTGTTGTTTGTCT

TTTTGTTTCTCCTTTTTT-----GCCATGGCATTGTCAGTTTATTTTCAACATATGATAA

TAAATGTCCC-TGTGGTATATTT--AGCCTCTCTCTTAGA--------------------

---AA-CTTAAGCAATTTTATTC-A-TTTTATTTTTC---------TACTTATTCAGTTC

AGGAAGCCCAATCAGTAGCTTGTACATCATACTACTGTAGTAAGTTCTGTGGGTCTGCTG

GTTGCTCATTATATGGATGTTACCTACTTCATCCTGGCAAAATTTGCTACTGCCTTCATT

GTCGCAGAGCTGAGTCTCCATTGGCACTTTCTGGAAGCGCTAGGAATGTGAACGACAGGA

ACAACGAGATGGACAACTCTCCAGTGTAAGTACTCAATACTAGATTTCGTTACACAATAT

AAAATTT----------GAAAAAAAA------GTTTTCTACATGCCAACATATATTTTAA

TTAAGGTGTTTACCAATTTTTGAAACAGATAATTTGAATTTTTTT-AAAATAAAATATTT

CATCTT-----AATTCAATATGTCGTTATCGTCTGAATCGTATTA------TGCAGTCAT

TGAAAAAAAA--TAAACACCTGAACTACTCCTGCAAGTGAAAGAAAGGAT-GT-TTT-TT

TCTGATATCGGTTAATGTGGTGGGAGGCAGATAAGATATCTTCTAGTGTGTCTCTCAATT

-ATTTACATTCAAAAACATTTTTTT----ACATAAATCGATCACTGGTCAATTGCACGAG

AGTAGTCAGAAAAAAACCCCTGTTATATGAAACGTTTTAATTGAATTTGTGAAAATAA-C

ACCACATATAAGAGAATATCTACTTTGTTTCAGGATGAATGAGATGGAAAATTTGGACCA

AGAAATGGATATGTTCTAG

>11_13Gen

ATGAAGGCAACGATCTTGTTAGCTGTTGTAGTGGCAGTCATTGTTGGAGGTAAATATCTT

TATTGAAATATATCTGTGTGTACGTATTTTAGAAGTATAGATCCAATATTTTTTAAGATA

AAAATACGGCAGACCGACTTTTCCAAAATTTGATTGCGAAACCCATATT---------TG

TATATTATGTAAAATTGTACTACTTTATCGTAATATTTTTTTATTAA---AAAATACTGT

TTTATGCAAAATAA----------------------------------------------

------------------------------------------------------------

------------------------------------------ATTATTAATGTTTACAAC

TGAAATATGTTTTGTTGTTAAAATTCAATCATTCAAATATTCAAACAAATCTTTTTTTCT

TTATACGTGTTGTTCAGTTTGTAGTTTTCTATGCTATGCTTTGTATACTATTGTTTGTCT

TTTA--------------------CCGTGTCGTTGTCAGTAGATTTGTAACTTATGATAA

CGAATGTCCC-TGTGGTATATTT--TGTCTCTCTTTTAGACAGGCATCGTT-ATTTGTG-

CACTA-TTTAATCAATTTTATTTTA-TTTCATTTTTTCTCCGTTTTTTATTTATAAGTTC

AGGAAGTCCAATCAGTACCTTGTGTATCAACCTACTGTGCTAGGTTCTGTGGGTCTGCTG

GTTGCTCATTATATGGATGTTACAGACGTACTTACTGCAAAATGTGCTACTGCCTTCATT

GTAGAAGAGCTGAGTCTCCATTGGCACTTTCTGGAAGCGCTAGGAATGTGAACGAGCAGA

ACAAAGATATTGACAACTCTCCAATGTAAGTAC--AATACACGACTTCGTTACATAATAT

AAACGTTTACAAATTTACAAAAGAAAAACATTGTATTCTACATGCCGACATATACTGTAT

TCAAGGTATAT--CATTTTTTTAAACAGATCATTTGAATTTTTTTTTAAATAAAGCATTT

CATCTTTGTTTAATTCAATAGGTCGTTATCGTCTGCA-CGTATTA------TATACAGTC

AT-----------AAACACATGAACTACTCCTGCAAGAGAAGGGAAGGAT-GT-TTT-TT

-T--ATATTGGTTAATGTGTTGGGAGGCAGATTAGATATCTTTTATTGTGTCTCTCAATT

-ATTTACATTCAAAAACATTTTT------ACATAAATCAATCACTGGTCAATTGCACGAG

AATAGTCAGAAAAAAAC---TGTTATATGAAACGTTTTAATTGAATTTGTGAAAAAAAAC

GCCACATATAAGAGACCATCTACTTTGTTTCAGGATGAATGAGATGGAACATTTGGACCA

AGAAATGGATATGTTCTAG

>11_15Gen

ATGAAGGCAACGATCTTGTTAGCTGTTGTAGTGGCAGTCATTGTTGGAGGTAAATATCTT

TATTGAAATATATCTGTGTGTACGTATTTTAGAAGTATAGATCCAGCATTTTT-AAGATA

AAAATACGGCAGACCGACTTCTCCAAAATTTGATTGCGAATCACATATTGTTAATTTATG

TATTATGTTAAATTATACTACTTCATTGTAATAATTTTTCATTTATA-----ATAACTGT

TTTAAGTAAGAAAGCTGTATTTGGCAAAACTTTTAGGAATTTTGGTCCTCAATGCTCTTC

TTTTTT-AACTTTTATGATTCGAGCGTCACTGATGAGTCTTTTGTAGACGAAACGCGCGT

CTGGCGTATACGTGATTTAGTTCTGGTATCTATGATGAGTTTATTGTTAATGTTTACAAC

TGAAATATGTTTTGTTATTAAAATTAAATCACTCAAATACTCAAACAAATATTTTTTTCT

TTATACGTGTTGTTCAGTTCGTTGTTTTCTATGCTATGCTTTGTATACTGTTGTTTGTCT

TTTTGTTTCTCCTTTTTT-----GCCATGGCATTGTCAGTTTATTTTCAACATATGATAA

TAAATGTCCC-TGTGGTATATTT--AGCCTCTCTCTTAGA--------------------

---AA-CTTAAGCAATTTTATTC-A-TTTTATTTTTC---------TACTTATTCAGTTC

AGGAAGCCCAATCAGTAGCTTGTACATCATACTACTGTAGTAAGTTCTGTGGGTCTGCTG

GTTGCTCATTATATGGATGTTACCTACTTCATCCTGGCAAAATTTGCTACTGCCTTCATT

GTCGCAGAGCTGAGTCTCCATTGGCACTTTCTGGAAGCGCTAGGAATGTGAACGACAGGA

ACAACGAGATGGACAACTCTCCAGTGTAAGTACTCAATACTAGATTTCGTTACACAATAT

AAAATTT----------GAAAAAAAA------GTTTTCTACATGCCAACATATATTTTAA

TTAAGGTGTTTACCAATTTTTGAAACAGATAATTTGAATTTTTTT-AAAATAAAATATTT

CATCTT-----AATTCAATATGTCGTTATCGTCTGAATCGTATTA------TGCAGTCAT

TGAAAAAAAA--TAAACACCTGAACTACTCCTGCAAGTGAAAGAAAGGATGGT-TTT-TT

-CTGATATCGGTTAATGTGGCGGGAGGCAGATAAGATATCTTCTAGTGTGTCTCTCAATT

-ATTTACATTCAAAAACATTTTTTT----ACATAAATCGATCACTGGTCAATTGCACGAG

AGTAGTCAGAAAAAAACCCCTGTTATATGAAACGTTTTAATTGAATTTGTGAAAATAA-C

ACCACATATAAGAGAATATCTACTTTGTTTCAGGATGAATGAGATGGAAAATTTGGACCA

AGAAATGGATATGTTCTAG

>11_16Gen

ATGAAGGCAACGATCTTGTTAGCTGTTGTAGTGGCAGTCATTGTTGGAGGTAAATATCTT

TATTGAAATATATCTGTGTGTACGTATTTTAGAAGTATAGATCCAGCATTTTT-AAGATA

AAAATACGGCAGACCGACTTCTCCAAAATTTGATTGCGAATCACATATTGTTAATTTATG

TATTATGTTAAATTATACTACTTCATTGTAATAATTTTTCATTTATA-----ATAACTGT

TTTAAGTAAGAAAGCTGTATTTGGCAAAACTTTTAGGAATTTTGGTCCTCAATGCTCTTC

TTTTTT-AACTTTTATGATTCGAGCGTCACTGATGAGTCTTTTGTAGACGAAACGCGCGT

CTGGCGTATACGTGATTTAGTTCTGGTATCTATGATGAGTTTATTGTTAATGTTTACAAC

TGAAATATGTTTTGTTATTAAAATTAAATCACTCAAATACTCAAACAAATATTTTTTTCT

TTGTACGTGTTGTTCAGTTCGTTGTTTTCTATGCTATGCTTTGTATACTGTTGTTTGTCT

TTTTGTTTCTCCTTTTTT-----GCCATGGCATTGTCAGTTTATTTTCAACATATGATAA

TAAATGTCCC-TGTGGTATATTT--AGCCTCTCTCTTAGA--------------------

---AA-CTTAAGCAATTTTATTC-A-TTTTATTTTTC---------TACTTATTCAGTTC

AGGAAGCCCAATCAGTAGCTTGTACATCATACTACTGTAGTAAGTTCTGTGGGTCTGCTG

GTTGCTCATTATATGGATGTTACCTACTTCATCCTGGCAAAATTTGCTACTGCCTTCATT

GTCGCAGAGCTGAGTCTCCATTGGCACTTTCTGGAAGCGCTAGGAATGTGAACGACAGGA

ACAACGAGATGGACAACTCTCCAGTGTAAGTACTCAATACTAGATTTCGTTACACAATAT

AAAATTT----------GAAAAAAAA------GTTTTCTACATGCCAACATATATTTTAA

TTAAGGTGTTTACCAATTTTTGAAACAGATAATTTGAATTTTTTT-AAAATAAAATATTT

CATCTT-----AATTCAATATGTCGTTATCGTCTGAATCGTATTA------TGCAGTCAT

TGAAAAAAAA--TAAACACCTGAACTACTCCTGCAAGTGAAAGAAAGGAT-GT-TTT-TT

TCTGATATCGGTTAATGTGGTGGGAGGCAGATAAGATATCTTCTAGTGTGTCTCTCAATT

-ATTTACATTCAAAAACATTTTTTT----ACATAAATCGATCACTGGTCAATTGCACGAG

AGTAGTCAGAAAAAAACCCCTGTTATATGAAACGTTTTAATTGAATTTGTGAAAATAA-C

ACCACATATAAGAGAATATCTACTTTGTTTCAGGATGAATGAGATGGAAAATTTGGACCA

AGAAATGGATATGTTCTAG

>11_17Gen

ATGAAGGCAACGATCTTGTTAGCTGTTGTAGTGGCAGTCATTGTTGGAGGTAAATATCTT

TATTGAAATATATCTGTGTGTACGTATTTTAGAAGTATAGATCCAGCATTTTT-AAGATA

AAAATACGGCAGACCGACTTCTCCAAAATTTGATTGCGAATCACATATTGTTAATTTATG

TATTATGTTAAATTATACTACTTCATTGTAATAATTTTTCATTTATA-----ATAACTGT

TTTAAGTAAGAAAGCTGTATTTGGCAAAACTTTTAGGAATTTTGGTCCTCAATGCTCTTC

TTTTTT-AACTTTTATGATTCGAGCGTCACTGATGAGTCTTTTGTAGACGAAACGCGCGT

CTGGCGTATACGTGATTTAGTTCTGGTATCTATGATGAGTTTATTGTTAATGTTTACAAC

TGAAATATGTTTTGTTATTAAAATTAAATCACTCAAATACTCAAACAAATATTTTTTTCT

TTATACGTGTTGTTCAGTTCGTTGTTTTCTATGCTATGCTTTGTATACTGTTGTTTGTCT

TTTTGTTTCTCCTTTTTT-----GCCATGGCATTGTCAGTTTATTTTCAACATATGATAA

TAAATGTCCC-TGTGGTATATTT--AGCCTCTCTCTTAGA--------------------

---AA-CTTAAGCAATTTTATTC-A-TTTTATTTTTC---------TACTTATTCAGTTC

AGGAAGCCCAATCAGTAGCTTGTACATCATACTACTGTAGTAAGTTCTGTGGGTCTGCTG

GTTGCTCATTATATGGATGTTACCTACTTCATCCTGGCAAATTTTGCTACTGCCTTCATT

GTCGCAGAGCTGAGTCTCCATTGGCACTTTCTGGAAGCGCTAGGAATGTGAACGACAGGA

ACAACGAGATGGACAACTCTCTAGTGTAAGTACTCAATACTAGATTTCGTTACACAATAT

AAAATTT----------GAAAAAAAA------GTTTTCAACATGCCAACATATATTTTAA

TTAAGGTGTTTACCAATTTTTGAAACAGATAATTTGAATTTTTTT-AAAATAAAATATTT

CATCTT-----AATTCAATATGTCGTTATCGTCTGAATCGTATTA------TGCAGTCAT

TGAAAAAAAA--TAAACACCTGAACTACTCCTGCAAGTGAAAGAAAGGAT-GT-TTT-TT

TCTGATATCGGTTAATGTGGTGGGAGGCAGATAAGATATCTTCTAGTGTGTCTCTCAATT

-ATTTACATTCAAAAACATTTTTTT----ACATAAATCGATCACTGGTCAATTGCACGAG

AGTAGTCAGAAAAAAACCCCTGTTATATGAAACGTTTTAATTGAATTTGTGAAAATAA-C

ACCACATATAAGAGAATATCTACTTTGTTTCAGGATGAATGAGATGGAAAATTTGGACCA

AGAAATGGATATGTTCTAG

>11_18Gen

ATGAAGGCAACGATCTTGTTAGCTGTTGTAGTGGCAGTCATTGTTGGAGGTAAATATCTT

TATTGAAATATATCTGTGTGTACGTATTTTAGAAGTATAGATCCAATATTTTTTAAGATA

AAAATACGGCAGACCGACTTTTCCAAAATTTGATTGCGAAACCCATATT---------TG

TATATTATGTAAAATTGTACTACTTTATTGTAATATTTGTTCATTAA---TGAATACTGT

TTTATGTAATATAA----------------------------------------------

------------------------------------------------------------

------------------------------------------ATTGTTAATGTTTACAAC

TGAAATATGTTTTGTTATTAAAATTCAATCATTCAAATATTCAAACAAATATTTTTTTCT

TTATACGTGTTGTTCAGTTTCTAGTTTTCTATGCTATGCTTTGTATACTGTTGTTTGTCT

TTTA--------------------CCATGTCGTTGTCAGTAGATTTTTAACTTATGATAT

CGAATGTCCC-TGTGGTATATTT--TGTCTCTCTTTTAGACAGGCATCGTT-ATTTGTG-

CATTA-TTTAATCAATTTTATTT-AATTTCATTTTTTCTCCGTTTTTCATTTATAAGTTC

AAGAAGTCCAATCAGTACCTTGTGCATCAACCTACTGTGCTAGGTTCTGTGGGTCTGCTG

GTTGCTCAAAATATGGATGTTACAGACTTCATCCCGGCAAAATTTGCTACTGCCTTCATT

GTAGAAGAGCTGAGTCTCCATTGGCACTTTCTGGAAGCGCTAGGAATGTGAACGAGCAGA

ACAAAGAGATGGACAACTCTCCCATGTAAGTAC--AATACAAGACTTCGTTACATAATAT

AAACGTTTACAAATTTACTAAAGAAAAACATTGTATTCTACATGCCAATGTATACTGTAT

TCAAGGTATAT--CATTTTTTTAAACAGATCATTTGAATTTTTTTT-AAATAAAGCATTT

CATCTTTGTTTAATTCAATAGGTCGTTATCGTCTGCA-CGTATTA------TTTACAGTC

AT-----------AAACACATGAACTACTCCTGCAAGAGAAGGAAGGAAT-GT-TTT-TT

-TTTATATCGGTTAATGTGTTGGGAGGCAGATAAGATATTTTTTATTGTGTCTCTCAATT

-ATTTACATTCAAAAACATTTTT------ACATAAATCAATCACTGGTTAATTGCACGAG

AATAGTCAGAAAAAAAC---TGTTATATGAAACGTTTTAATTGAATTTGTGAAAAAAAAC

GCCACATATAAGAGACCATCTACTTTGTTTCAGGATGAATGAGATGGAACATTTGGACCA

AGAAATGGATATGTTCTAG

>11_19Gen

ATGAAGGCAACGATCTTGTTAGCTGTTGTAGTGGCAGTCATTGTTGGAGGTAAATATCTT

TATTGAAATATATCTGTGTGTACGTATTTTAGAAGTATAGATCCAATATTTTTTAAGATA

AAAATACGGCAGACCGACTTTTCCAAAATTTGATTGCGAAACCCATATT---------TG

TATATTATGTAAAATTGTACTACTTTATCGTAATATTTTTTTATTAA---AAAATACTGT

TTTATGCAAGATAA----------------------------------------------

------------------------------------------------------------

------------------------------------------ATTATTAATGTTTACAAC

TGAAATATGTTTTGTTATTAAAATTCAATCATTCAAATATTCAAACAAATCTTTTTTTCT

TTATACGTGTTGTTCAGTTTGTAGTTTTCTATGCTATGCTTTGTATACTATTGTTTGTCT

TTTA--------------------CCGTGTCGTTGTCAGTAGATTTGTAACTTATGATAA

CGAATGTCCC-TGTGGTATATTT--TGTCTCTCTTTTAGACAGGCATCGTT-ATTTGTG-

CACTA-TTTAATCAATTTTATTTTA-TTTCATTTTTTCTCCGTTTTTTATTTATAAGTTC

AGGAAGTCCAATCAGTACCTTGTGTATCAACCTATTGTGCTAGGTTCTGTGGGTCTGCTG

GTTGCTCATTATATGGATGTTACAGACGTACTTACTGCAAAATGTGCTACTGCCTTCATT

GTAGAAGAGCTGAGTCTCCATTGGCACTTTCTGGAAGCGCTAGGAATGTGAACGAGCAGA

ACAAAGATATTGACAACTCTCCAATGTAAGTAC--AATACACGACTTCGTTACATAATAT

AAACGTTTACAAATTTACAAAAGAAAAACATTGTATTCTACATGCCGACATATACTGTAT

TCAAGGTATAT--CATTTTTTTAAACAGATCATTTGAATTTTTTTTTAAATAAAGCATTT

CATCTTTGTTTAATTCAATAGGTCGTTATTGTCTGCA-CGTATTA------TATACAGTC

AT-----------AAACACATGAACTACTCCTGCAAGAGAAGGAAAGGAT-GT-TTT-TT

-T--ATATTGGTTAATGTGTTGGGAGGCAGATTAGATATTTTTTATTGTGTCTCTCAATT

-ATTTACATTCAAAAACATTTTT------ACATAAATCAATCACTGGTCAATTGCACGAG

AATAGTCAGAAAAAAAC---TGTTATATGAAACGTTTTAATTGAATTTGTGAAAAAAAAC

GCCACATATAAGAGACCATCTACTTTGTTTCAGGATGAATGAGATGGAACATTTGGACCA

AGAAATGGATATGTTCTAG

>11_20Gen

ATGAAGGCAACGATCTTGTTAGCTGTTGTAGTGGCAGTCATTGTTGGAGGTAAATATCTT

TATTGAAATATATCTGTGTGTACGTATTTTAGAAGTATAGATCCAATATTTTTTAAGATA

AAAATACGGCAGACCGACTTTTCCAAAATTTGATTGCGAAACCCATATT---------TG

TATATTATGTAAAATTGTACTACTTTATCGTAATATTTTTTTATTAA---AAAATACTGT

TTTATGCAAGATAA----------------------------------------------

------------------------------------------------------------

------------------------------------------ATTATTAATGTTTACAAC

TGAAATATGTTTTGTTATTAAAATTCAATCATTCAAATATTCAAACAAATCTTTTTTTCT

TTATACGTGTTGTTCAGTTTGTAGTTTTCTATGCTATGCTTTGTATACTATTGTTTGTCT

TTTA--------------------CCGTGTCGTTGTCAGTAGATTTGTAACTTATGATAA

CGAATGTCCC-TGTGGTATATTT--TGTCTCTCTTTTAGACAGGCATCGTT-ATTTGTG-

CACTA-TTTAATCAATTTTATTTTA-TTTCATTTTTTCTCCGTTTTTTATTTATAAGTTC

AGGAAGTCCAATCAGTACCTTGTGTATCAACCTACTGTGCTAGGTTCTGTGGGTCTGCTG

GTTGCTCATTATATGGATGTTACAGACGTACTTACTGCAAAATGTGCTACTGCCTTCATT

GTAGAAGAGCTGAGTCTCCATTGGCACTTTCTGGAAGCGCTAGGAATGTGAACGAGCAGA

ACAAAGATATTGACAACTCTCCAATGTAAGTAC--AATACACGACTTCGTTACATAATAT

AAACGTTTACAAATTTACAAAAGAAAAACATTGTATTCTACATGCCGACATATACTGTAT

TCAAGGTATAT--CATTTTTTTAAACAGATCATTTGAATTTTTTTTTAAATAAAGCATTT

CATCTTTGTTTAATTCAATAGGTCGTTATCGTCTGCA-CGTATTA------TATACAGTC

AT-----------AAACACATGAACTACTCCTGCAAGAGAAGGAAAGGAT-GT-TTT-TT

-T--ATATTGGTTAATGTGTTGGGAGGCAGATTAGATATTTTTTATTGTGTCTCTCAATT

-ATTTACATTCAAAAACATTTTT------ACATAAATCAATCACTGGTCAATTGCACGAG

AATAGTCAGAAAAAAAC---TGTTATATGAAACGTTTTAATTGAATTTGTGAAAAAAA-C

GCCACATATAAGAGACCATCTACTTTGTTTCAGGATGAATGAGATGGAACATTTGGACCA

AGAAATGGATATGTTCTAG

>11_01cDNA

ATGAAGGCAACGATCTTGTTAGCTGTTGTAGTGGCAGTCATTGTTGGAG-----------

------------------------------------------------------------

------------------------------------------------------------

------------------------------------------------------------

------------------------------------------------------------

------------------------------------------------------------

------------------------------------------------------------

------------------------------------------------------------

------------------------------------------------------------

------------------------------------------------------------

------------------------------------------------------------

---------------------------------------------------------TTC

AGGAAGCCCAATCAATTCCTTGTACATCGTACTACTGTAGTAAGTTCTGTGGGTCTGCTG

GTTGCTCATTATATGGATGTTACAAACTTCATCCCGGCAAAATTTGCTACTGCCTTCATT

GTCGCAGAGCTGAGTCTCCATTGGCACTTTCTGGAAGCGCTAGGAATGTGAACGAGCAGA

ACAAAGAGATGGACAACTCTCCCAT-----------------------------------

------------------------------------------------------------

------------------------------------------------------------

------------------------------------------------------------

------------------------------------------------------------

------------------------------------------------------------

------------------------------------------------------------

------------------------------------------------------------

---------------------------------GATGAATGAGATGGAACATTTGGACCA

AGAAATGGATATGTTCTAG

>11_02cDNA

ATGAAGGCAACGATCTTGTTAGCTGTTGTAGTGGCAGTCATTGTTGGAG-----------

------------------------------------------------------------

------------------------------------------------------------

------------------------------------------------------------

------------------------------------------------------------

------------------------------------------------------------

------------------------------------------------------------

------------------------------------------------------------

------------------------------------------------------------

------------------------------------------------------------

------------------------------------------------------------

---------------------------------------------------------TTC

AGGAAGCCCAATCAATTCCTTGTACATCATACTACTGTAGTAAGTTCTGTGGGTCTGCTG

GTTGCTCATTATATGGATGTTACAAACTTCATCCCGGCAAAATTTGCTACTGCCTTCATT

GTCGCAGAGCTGAGTCTCCATTGGCACTTTCTGGAAGCGCTAGGAATGTGAACGAGCAGA

ACAAAGAGATGGACAACTCTCCAGT-----------------------------------

------------------------------------------------------------

------------------------------------------------------------

------------------------------------------------------------

------------------------------------------------------------

------------------------------------------------------------

------------------------------------------------------------

------------------------------------------------------------

---------------------------------GATGAATGAGGTGGAAAATTTGGACCA

AGAAATGGATATGTTTTAG

>11_03cDNA

ATGAAGGCAACGATCTTGTTAGCTGTTGTAGTGGCAGTCATTGTTGGAG-----------

------------------------------------------------------------

------------------------------------------------------------

------------------------------------------------------------

------------------------------------------------------------

------------------------------------------------------------

------------------------------------------------------------

------------------------------------------------------------

------------------------------------------------------------

------------------------------------------------------------

------------------------------------------------------------

---------------------------------------------------------TTC

AGGAAGCCCAATCAATTCCTTGTACATCATACTACTGTAGTAAGTTCTGTGGGTCTGCTG

GTTGCTCATTATATGGATGTTACAAACTTCATCCCGGCAAAATTTGCTACTGCCTTCATT

GTCGCAGAGCTGAGTCTCCATTGGCACTTTCTGGAAGCGCTAGGAATGTGAACGAGCAGA

ACAAAGAGATGGACAACTCTCCAGT-----------------------------------

------------------------------------------------------------

------------------------------------------------------------

------------------------------------------------------------

------------------------------------------------------------

------------------------------------------------------------

------------------------------------------------------------

------------------------------------------------------------

---------------------------------GATGAATGAGGTGGAAAATTTGGACCA

AGAAATGGATATGTTTTAG

>11_04cDNA

ATGAAGGCAACGATCTTGTTAGCTGTTGTAGTGGCAGTCATTGTTGGAG-----------

------------------------------------------------------------

------------------------------------------------------------

------------------------------------------------------------

------------------------------------------------------------

------------------------------------------------------------

------------------------------------------------------------

------------------------------------------------------------

------------------------------------------------------------

------------------------------------------------------------

------------------------------------------------------------

---------------------------------------------------------TTC

AAGAAGTCCAATCAGTACCTTGTGCATCAACCTACTGTGCTAGGTTCTGTGGGTCTGCTG

GTTGCTCAAAATATGGATGTTACAGACTTCATCCCGGCAAAATTTGCTACTGCCTTCATT

GTAGAAGAGCTGAGTCTCCATTGGCACTTTCTGGAAGCGCTAGGAATGTGAACGAGCAGA

ACAAAGAGATGGACAACTCTCCCAT-----------------------------------

------------------------------------------------------------

------------------------------------------------------------

------------------------------------------------------------

------------------------------------------------------------

------------------------------------------------------------

------------------------------------------------------------

------------------------------------------------------------

---------------------------------GATGAATGAGATGGAACATTTGGACCA

AGAAATGGATATGTTCTAG

>11_05cDNA

ATGAAGGCAACGATCTTGTTAGCTGTTGTAGTGGCAGTCATTGTTGGAG-----------

------------------------------------------------------------

------------------------------------------------------------

------------------------------------------------------------

------------------------------------------------------------

------------------------------------------------------------

------------------------------------------------------------

------------------------------------------------------------

------------------------------------------------------------

------------------------------------------------------------

------------------------------------------------------------

---------------------------------------------------------TTC

AGGAAGCCCAATCAATTCCTTGTACATCATACTACTGTAGTAAGTTCTGTGGGTCTGCTG

GTTGCTCATTATATGGATGTTACAAACTTCATCCCGGCAAAATTTGCTACTGCCTTCATT

GTCGCAGAGCTGAGTCTCCATCGGCACTTTCTGGAAGCGCTAGGAATGTGAACGAGCAGA

ACAAAGAGATGGACAACTCTCCAGT-----------------------------------

------------------------------------------------------------

------------------------------------------------------------

------------------------------------------------------------

------------------------------------------------------------

------------------------------------------------------------

------------------------------------------------------------

------------------------------------------------------------

---------------------------------GATGAATGAGGTGGAAAATTTGGACCA

AGAAATGGATATGTTTTAG

>11_06cDNA

ATGAAGGCAACGATCTTGTTAGCTGTTGTAGTGGCAGTCATTGTTGGAG-----------

------------------------------------------------------------

------------------------------------------------------------

------------------------------------------------------------

------------------------------------------------------------

------------------------------------------------------------

------------------------------------------------------------

------------------------------------------------------------

------------------------------------------------------------

------------------------------------------------------------

------------------------------------------------------------

---------------------------------------------------------TTC

AAGAAGTCCAATCAGTACCTTGTGCATCAACCTACTGTGCTAGGTTCTGTGGGTCTGCTG

GTTGCTCAAAATATGGATGTTACAGACTTCATCCCGGCAAAATTTGCTACTGCCTTCATT

GTAGAAGAGCTGAGTCTCCATTGGCACTTTCTGGAAGCGCTAGGAATGTGAACGAGCAGA

ACAAAGAGATGGACAACTCTCCCAT-----------------------------------

------------------------------------------------------------

------------------------------------------------------------

------------------------------------------------------------

------------------------------------------------------------

------------------------------------------------------------

------------------------------------------------------------

------------------------------------------------------------

---------------------------------GATGAATGAGATGGAACATTTGGACCA

AGAAATGGATATGTTCTAG

>11_07cDNA

ATGAAGGCAACGATCTTGTTAGCTGTTGTAGTGGCAGTCATTGTTGGAG-----------

------------------------------------------------------------

------------------------------------------------------------

------------------------------------------------------------

------------------------------------------------------------

------------------------------------------------------------

------------------------------------------------------------

------------------------------------------------------------

------------------------------------------------------------

------------------------------------------------------------

------------------------------------------------------------

---------------------------------------------------------TTC

AGGAAGCCCAATCAGTAGCTTGTACATCATACTACTGTAGTAAGTTCTGTGGGTCTGCTG

GTTGCTCATTATATGGATGTTACCTACTTCATCCTGGCAAAATTTGCTACTGCCTTCATT

GTCGCAGAGCTGAGTCTCCATTGGCACTTTCTGGAAGCGCTAGGAATGTGAACGACAGGA

ACAACGAGATGGACAACTCTCCAGT-----------------------------------

------------------------------------------------------------

------------------------------------------------------------

------------------------------------------------------------

------------------------------------------------------------

------------------------------------------------------------

------------------------------------------------------------

------------------------------------------------------------

---------------------------------GATGAATGAGATGGAAAATTTGGACCA

AGAAATGGATATGTTCTAG

>11_08cDNA

ATGAAGGCAACGATCTTGTTAGCTGTTGTAGTGGCAGTCATTGTTGGAG-----------

------------------------------------------------------------

------------------------------------------------------------

------------------------------------------------------------

------------------------------------------------------------

------------------------------------------------------------

------------------------------------------------------------

------------------------------------------------------------

------------------------------------------------------------

------------------------------------------------------------

------------------------------------------------------------

---------------------------------------------------------TTC

AGGAAGCCCAATCAATTCCATGTACATCATACTACTGTAGTAAGTTCTGTGGGTCTGCTG

GTTGCTCATTATATGGATGTTACAAACTTCATCCCGGCAAAATTTGCTACTGCCTTCATT

GTCGCAGAGCTGAGTCTCCATTGGCACTTTCTGGAAGCGCTAGAAATATGAACGAGCAGA

ACAAAGAGATGGACAACTCTCCAAT-----------------------------------

------------------------------------------------------------

------------------------------------------------------------

------------------------------------------------------------

------------------------------------------------------------

------------------------------------------------------------

------------------------------------------------------------

------------------------------------------------------------

---------------------------------GATGAATGAGATGGAACATTTGGACCA

AGAAATGGATATGTTCTAG

>11_10cDNA

ATGAAGGCAACGATCTTGTTAGCTGTTGTAGTGGCAGTCATTGTTGGAG-----------

------------------------------------------------------------

------------------------------------------------------------

------------------------------------------------------------

------------------------------------------------------------

------------------------------------------------------------

------------------------------------------------------------

------------------------------------------------------------

------------------------------------------------------------

------------------------------------------------------------

------------------------------------------------------------

---------------------------------------------------------TTC

AAGAAGTCCAATCAGTACCTTGTGCATCAACCTACTGTGCTAGGTTCTGTGGGTCTGCTG

GTTGCTCAAAATATGGATGTTACAGACTTCATCCCGGCAAAATTTGCTACTGCCTTCATT

GTAGAAGAGCTGAGTCTCCATTGGCACTTTCTGGAAGCGCTAGGAATGTGAACGAGCAGA

ACAAAGAGATGGACAACTCTCCCAT-----------------------------------

------------------------------------------------------------

------------------------------------------------------------

------------------------------------------------------------

------------------------------------------------------------

------------------------------------------------------------

------------------------------------------------------------

------------------------------------------------------------

---------------------------------GATGAATGAGATGGAACATTTGGACCA

AGAAATGGATATGTTCTAG

>13_01Gen

ATGAAGGCAACGATCTTGTTAGCTGTTGTAGTGGCAGTCATTGTTGGAGGTAAATATCTT

TATTGAAATATAAATGTGTATGCGTATTTTAGAAGTATAGATCCAGCATTTTT-AAGATA

AAAATACGGCAGACCGACTCCTCCAAAATTTGATTGCGAATCCCATATT---------TG

TATATTATGTTAAATTATACTACTTTATTGTAATAATTTTTCATTTA---TAATAACTGT

TTTAAGTAAGGAAGCTGTAATTGGCAAAACTTTTAGGAATTTTGGTCCCCAATGCTCTTC

TTTTTTTAACTTTTATGATTCGAGCGTCACTGATGAGTCTTTTGCAGACGAAACGCGCGT

CTTGCGTATACGTAATTTAGTTCTGGTATCTATGATGAGTTTATTGTTAATGTTTACAAC

TGAAATACGTTTTGTTGTTAAAATTAAATCACTCTAATACTCAAACAAATATTTTTT-CT

TTATACGTGTTGTTCAGTTCGTAGTTTTCTATGCTATGCTTTGTATACTGTTGTTTTTCT

GTTTTTT-CTCCTTTTTTT----GCCATGGCGTTGTCAGTTTATTTTCAACTTATGATAA

TAAATGTCCC-TGTGGTATATTT--AGCCTCTCTCTAAGACAGGCATCGTT-ATTTATGT

CGAAA-CTTAAGCAATTTTATTC-A-TTTTATTTTT-CTAC------TTATTCA--GTTC

AGGAAGCCCAATCAGTAGCTTGTACATCATACTACTGTAGTAAGTTCTGTGGGTCTGCTG

GTTGCTCATTATATGGATGTTACCTACTTCATCCTGGCAAAATTTGCTACTGCCTTCATT

GTCGCAGAGCTGAGTCTCCATTGGCACTTTCTGGAAGCGCTAGGAATGTGAACGACAAGA

ACAACGAGATGGACAACTCTCCAGTGTAAGTACTCAATACTAGATTTCGTTACACAATAT

AAAATTT----------GAAAAAAAA------GTTTTCTACATGCCAGCATATATTTTAA

TTAAGGTGTTTACCAATTTTTGAAACAGATAATTTGAA-TTTTTTTAAAATAAAATATTT

CATCTT-----AATTCAATATGTCGTTATCGTCTGAATCGTATTA------TGCAGTCAT

TGAAAAAAAA--TAAACACCTGAACTACTCCTGCAAGTGAAAGAAAGGAT-GT-TTT-TT

TCTGATATCGGTTAATGTGTTGGGAGGCAGATAAGATATCTTCTAGTGTTTCTCACAATT

-ATTTACATTCAAAAACATTTTTTT----ACATAAATCGATCACTGGTCAATTGCACGAG

AGTAGTCAGAAAAAAAACCCTGTTATATGAAACGTTTTAATTGAATTTGTGAAAATAA-C

ACCACATATAAGAGAACATCTACTTTGTTTCAGGATGAATGAGGTGGAAAATTTGGACCA

AGAAATGGATATGTTCTAG

>13_04Gen

ATGAAGGCAACGATCTTGTTAGCTGTTGTAGTGGCAGTCATTGTTGGAGGTAAATATCTT

TATTGAAATATATCTGTGTGTACGTATTTTAGAAGTATAGATCCAGCATTTTT-AAGATA

AAAATACGGCAGACCGACTTCTTCAAAATTTGATTGCGAATCCCATATT---------TG

TATATTATGTTAAATTATACTACTTTATTGTAATAATTTTTCATTTA---TAATAACTGT

TTTAAGTAAGAAAGCTGTATTTGGCAAAACTGTTAGAAATTTTGGTCCTCAATGCTCTTC

TTTTTT-GACTTTTATGATTCGAGCGTCACTGATGAGTCTTTTGTAGACGAAACGCGCGT

CTGGCGTATACGTAATTTAGTCCTGGTATCTATGATGAGTTTATTGTTAATGTTTACAAC

TGAAATATGTTTTGTTATTAAAATTAAATCACTCAAATACTCAAACAAATATTTTTT-CT

TTATACGTGTTGTTCAGTTCGTAGTTTTCTATGCTATGCTTTGTATACTGTTGTTTGTCT

TTTTT---CTCCTTTTTTT----GACATGGCGTTGTCAGTTTATTTTCAACGTATGATAA

TAAATGTCCC-TGTGGTATATTT--AGCCTCTCTCTAAGACAGTCATCGTT-ATTTGTGT

CGAAA-CTTGAGCAATTTTATTC-A-TTTTATTTTT-CTAC------TTATTCA--GTTC

ACGAAGCCCAATCAGTAGCGTGTACATCATACTACTGTAGTAAGTTCTGTGGGTCTGCTG

GTTGCTCATTATATGGATGTTACCTACTTCATCCTGGCAAAATTTGCTACTGCCTTCATT

GTCGCAGAGCTGAGTCTCCATTGGCACTTTCTGGAAGCGCTAGGAATGTGAACGACAAGA

ACAACGAGATGGACAACTCTCCAGTGTAAGTACTCAATACTAGATTTCGTTACACAATAT

AAA-ATTT---------GAAAAAAAA------GTTTTCTACATGCCAACATATATTGTAA

TTAAGGTGTTTACCAATTTTTGAAACAGATAATTTGATCTTTTTTTTTAATAAAATATTT

CATCTT-----AATTCAATATGTCGTTATCGTCTGAATCGTATTA------TGCAGTCAT

TGAAAAAAAAAATAAACACCTGAACTACTCCTGCAAGAGAAAGAAGGGAT-GT-TTT-TT

TCTAAGATCGGTTAATGTGTTGGGAGGTAGATAAGATATCTTGTATTGTGTCTCTCAACT

-ATTTACATTCAAAAACATTTTT------ACATAAATCGATCACTGGTTAACTGCACGAG

AATAGTCAGAAAACAA-CCCTGTTATATGAAACGTTTTAATTGAATTTGTGAAAATAA-C

ACCACATATAAGAAAACATCTACTTTGTTTCAGGATGAATGAGGTGGAAAATTTGGACCA

AGAAATGGATATGTTCTAG

>13_05Gen

ATGAAGGCAACGATCTTGTTAGCTGTTGTAGTGGCAGTCATTGTTGGAGGTAAATATCTT

TATTGAAATATAAATGTGTATGCGTATTTTAGAAGTATAGATCCAGCATTTTT-AAGATA

AAAATACGGCAGACCGACTTCTCCAAAATTTGATTGCGAATCCCATATT---------TG

TATATTATGTTAAATTATACTACTTTATTGTAATAATTTTTCATTTA---TAATAACTGT

TTTAAGTAAGAAAGCTGTAATTGGCAAAACTTTTAGGAATTTTGGTCCCCAATGCTCTTC

TTTTTTTAACTTTTATGATTCGAGCGTCACTGATGAGTCTTTTGCAGACGAAACGCGCGT

CTTGCGTATACGTAATTTAGTTCTGGTATCTATGATGAGTTTATTGTTAATGTTTACAAC

TGAAATATGTTTTGTTGTTAAAATTAAATCACTCTAATACTCAAACAAATATTTTTT-CT

TTATACGTGTTGTTCAGTTCGTAGTTTTCTATGCTATGCTTTGTATACTGTTGTTTTTCT

GTTTTTT-CTCCTTTTTTT----GCCATGGCGTTGTCAGTTTATTTTCAACTTATGATAA

TAAATGTCCC-TGTGGTATATTT--AGCCTCTCTCTAAGACAGGCATCGTT-ATTTATGT

CGAAA-CTTAAGCAATTTTATTC-A-TTTTATTTTT-CTAC------TTATTCA--GTTC

AGGAAGCCCAATCAGTAGCTTGTACATCATACTACTGTAGTAAGTTCTGTGGGTCTGCTG

GTTGCTCATTATATGGATGTTACCTACTTCATCCTGGCAAAATTTGCTACTGCCTTCATT

GTCGCAGAGCTGAGTCTCCATTGGCACTTTCTGGAAGCGCTAGGAATGTGAACGACAAGA

ACAACGAGATGGACAACTCTCCAGTGTAAGTACTCAATACTAGATTTCGTTACACAATAT

AAAATTT----------GAAAAAAAA------GTTTTCTACATGCCAGCATATATTTCAA

TTAAGGTGTTTACCAATTTTTGAAACAGATAATTTGAA-TTTTTTTAAAATAAAATATTT

CATCTT-----AATTCAATATGTCGTTATCGTCTGAATCGTATTA------TGCAGTCAT

TGAAAAAAAA--TAAACACCTGAACTACTCCTGCAAGTGAAAGAAAGGAT-GT-TTT-TT

TCTGATATCGGTTAATGTGTTGGGAGGCAGATAAGATATCTTCTAGTGTTTCTCACAATT

-ATTTACATTCAAAAACATTTTTTT----ACATAAATCGATCACTGGTCAATTGCACGAG

AGTAGTCAGAAAAAAAACCCTGTTATATGAAACGTTTTAATTGAATTTGTGAAAATAA-C

ACCACATATAAGAGAACATCTACTTTGTTTCAGGATGAATGAGGTGGAAAATTTGGACCA

AGAAATGGATATGTTCTAG

>13_06Gen

ATGAAGGCAACGATCTTGTTAGCTGTTGTAGTGGCAGTCATTGTTGGAGGTAAATATCTT

TATTGAAATATATCTGTGTGTACGTATTTTAGAAGTATAGATCCAGCATTTTT-AAGATA

AAAATACGGCAGACCGACTTCTTCAAAATTTGATTGCGAATCCCATATT---------TG

TATATTATGTTAAATTATACTACTTTATTGTAATAATTTTTCACTTA---TAATAACTGT

TTTAAGTAAGAAAGCTGTATTTGGCAAAACTGTTAGAAATTTTGGTCCTCAATGCTCTTC

TTTTTT-GACTTTTATGATTCGAGCGTCACTGATGAGTCTTTTGTAGACGAAACGCGCGT

CTGGCGTATACGTAATTTAGTCCTGGTATCTATGATGAGTTTATTGTTAATGTTTACAAC

TGAAATATGTTTTGTTATTAAAATTAAATCACTCAAATACTCAAACAAATATTTTTT-CT

TTATACGTGTTGTTCAGTTCGTAGTTTTCTATGCTATGCTTTGTATACTGTTGTTTGTCT

TTTTT---CTCCTTTTTTT----GACATGGCGTTGTCAGTTTATTTTCAACGTATGATAA

TAAATGTCCC-TGTGGTATATTT--AGCCTCTCTCTAAGACAGTCATCGTT-ATTTGTGT

CGAAA-CTTGAGCAATTTTATTC-A-TTTTATTTTT-CTAC------TTATTCA--GTTC

AGGAAGCCCAATCAGTAGCGTGTACATCATACTACTGTAGTAAGTTCTGTGGGTCTGCTG

GTTGCTCATTATATGGATGTTACCTACTTCATCCTGGCAAAATTTGCTACTGCCTTCATT

GTCGCAGAGCTGAGTCTCCATTGGCACTTTCTGGAAGCGCTAGGAATGTGAACGACAAGA

ACAACGAGATGGACAACTCTCCAGTGTAAGTACTCAATACTAGATTTCGTTACACAATAT

AAAATTT----------GAAAAAAAA------GTTTTCTACATGCCAACATATATTGTAA

TTAAGGTGTTTACCAATTTTTGAAACAGATAATTTGATCTTTTTTTTTAATAAAATATTT

CATCTT-----AATTCAATATGTCGTTATCGTTTGAATCGTATTA------TGCAGTCAT

TGAAAAAAAAA-TAAACACCTGAACTACTCCTGCAAGAGAAAGAAGGGAT-GT-TTT-TT

TCTAAGATCGGTTAATGTGTTGGGAGGTAGATAAGATATCTTGTATTGTGACTCTCAACT

-ATTTACATTCAAAAACATTTTT------ACATAAATCGATCACTGGTTAACTGCACGAG

AATAGTCAGAAAACAA-CCCTGTTATATGAAACGTTTTAATTGAATTTGTGAAAATAA-C

ACCACATATAAGAAAACATCTACCTTGTTTCAGGATGAATGAGGTGGAAAATTTGGACCA

AGAAATGGATATGTTCTAG

>13_08Gen

ATGAAGGCAACGATCTTGTTAGCTGTTGTAGTGGCAGTCATTGTTGGAGGTAAATATCTT

TATTGAAATATATCTGTGTGTACGTATTTTAGAAGTATAGATCCAGCATTTTT-AAGATA

AAAATACGGCAGACCGACTTCTTCAAAATTTGATTGCGAATCCCATATT---------TG

TATATTATGTTAAATTATACTACTTTATTGTAATAATTTTTCATTTA---TAATAACTGT

TTTAAGTAAGAAAGCTGTATTTGGCAAAACTGTTAGAAATTTTGGTCCTCAATGCTCTTC

TTTTTT-GACTTTTATGATTCGAGCGTCACTGATGAGTCTTTTGTAGACGAAACGCGCGT

CTGGCGTATACGTAATTTAGTCCTGGTATCTATGATGAGTTTATTGTTAATGTTTACAAC

TGAAATATGTTTTGTTATTAAAATTAAATCACTCAAATACTCAAACAAATATTTTTT-CT

TTATACGTGTTGTTCAGTTCGTAGTTTTCTATGCTATGCTTTGTATACTGTTGTTTGTCT

TTTTT---CTCCTTTTTTT----GACATGGCGTTGTCAGTTTATTTTCAACGTATGATAA

TAAATGTCCCCTGTGGTATATTTTAAGCCTCTCTCTAAGACAGTCATCGTT-ATTTGTGT

CGAAAACTTGAGCAATTTTATTC-A-TTTTATTTTTTCTAC------TTATTCA--GTTC

AGGAGCCCCAACCAGTAGCTTGTACATCATACTACTGTAGTAAGTTCTGTGGGTCTGCTG

GTTGCTCATTATATGGATGTTACCTACTTCATCCTGGCAAAATTTGCTACTGCCTTCATT

GTCGCAGAGCTGAGTCTCCATTGGCACTTTCTGGAAGCGCTAGGAATGTGAACGACAAGA

ACAACGAGATGGACAACTCTCCAGTGTAAGTACTCAATACTAGATTTCGTTACACAATAT

AAAATTT----------GAAAAAAAA------GTTTTCTACATGCCAACATATATTGTAA

TTAAGGTGTTTACCAATTTTTGAAACAGATAATTTGATCTTTTTTTTTAATAAAATATTT

CATCTT-----AATTCAATATGTCGTTATCGTTTGAATCGTATTA------TGCAGTCAT

TGAAAAAAAAAATAAACACCTGAACTACTCCTGCAAGAGAAAGAAGGGAT-GT-TTT-TT

TCTAAGATCGGTTAATGTGTTGGGAGGTAGATAAGATATCTTGTATTGTGTCTCTCAACT

-ATTTACATTCAAAAACATTTTT------ACATAAATCGATCACTGGTTAACTGCACGAG

AATAGTCAGAAAACAA-CCCTGTTATATGAAACGTTTTAATTGAATTTGTGAAAATAA-C

ACCACATATAAGAAAACATCTACTTTGTTTCAGGATGAATGAGGTGGAAAATTTGGACCA

AGAAATGGATATGTTCTAG

>13_09Gen

ATGAAGGCAACGATCTTGTTAGCTGTTGTAGTGGCAGTCATTGTTGGAGGTAAATATCTT

TATTGAAATATATCTGTGTGTACGTATTTTAGAAGTATAGTTCCAGCATTTTT-AAGATA

AAAATACGGCAGACCGACTTCTTCAAAATTTGATTGCGAATCCCATATT---------TG

TATATTATGTTAAATTATACTACTTTATTGTAATAATTTTTCATTTA---TAATAACTGT

TTTAAGTAAGAAAGCTGTATTTGGCAAAACTGTTAGAAATTTTGGTCCTCAATGCTCTTC

TTTTTT-GACTTTTATGATTCGAGCGTCACTGATGAGTCTTTTGTAGACGAAACGCGCGT

CTGGCGTATACGTAATTTAGTCCTGGTATCTATGATGAGTTTATTGTTAATGTTTACAAC

TGAAATATGTTTTGTTATTAAAATTAAATCACTCAAATACTCAAACAAATATTTTTT-CT

TTATACGTGTTGTTCAGTTCGTAGTTTTCTATGCTATGCTTTGTATACTGTTGTTTGTCT

TTTTT---CTCCTTTTTTT----GACATGGCGTTGTCAGTTTATTTTCAACGTATGATAA

TAAATGTCCC-TGTGGTATATTT--AGCCTCTCTCTAAGACAGTCATCGTT-ATTTGTGT

CGAAA-CTTGAGCAATTTTATTC-A-TTTTATTTTT-CTAC------TTATTCA--GTTC

AGGAAGCCCAATCAGTAGCGTGTACATCATACTACTGTAGTAAGTTCTGTGGGTCTGCTG

GTTGCTCATTATATGGATGTTACCTACTTCATCCTGGCAAAATTTGCTACTGCCTTCATT

GTCGCAGAGCTGAGTCTCCATTGGCACTTTCTGGAAGCGCTAGGAATGTGAACGACAAGA

ACAACGAGATGGACAACTCTCCAGTGTAAGTACTCAATACTAGATTTCGTTACACAATAT

AAAATTT----------GAAAAAAAA------GTTTTCTACATGCCAACATATATTGTAA

TTAAGGTGTTTACCAATTTTTGAAACAGATAATTTGATCTTTTTTTTTAATAAAATATTT

CATCTT-----AATTCAATATGTCGTTATCGTCTGAATCGTATTA------TGCAGTCAT

TGAAAAAAAAA-TAAACACCTGAACTACTCCTGCAAGAGAAAGAAGGGAT-GT-TTT-TT

TCTAAGATCGGTTAATGTGTTGGGAGGTAGATAAGATATCTTGTATTGTGTCTCTCAACT

-ATTTACATTCAAAAACATTTTT------ACATAAATCGATCACTGGTTAACTGCACGAG

AATAGTCAGAAAACAA-CCCTGTTATATGAAACGTTTTAATTGAATTTGTGAAAATAA-C

ACCACATATAAGAAAACATCTACTTTGTTTCAGGATGAATGAGGTGGAAAATTTGGACCA

AGAAATGGATATGTTCTAG

>13_10Gen

ATGAAGGCAACGATCTTGTTAGCTGTTGTAGTGGCAGTCATTGTTGGAGGTAAATATCTT

TATTGAAATATAAATGTGTATGCGTATTTTAGAAGTATAGATCCAGCATTTTT-AAGATA

AAAATACGGCAGACCGACTTCTCCAAAATTTGATTGCGAATCCCATATT---------TG

TATATTATGTTAAATTATACTACTTTATTGTAATAATTTTTCATTTA---TAATAACTGT

TTTAAGTAAGAAAGCTGTAATTGGCAAAACTTTTAGGAATTTTGGTCCCCAATGCTCTTC

TTTTTTTAACTTTTATGATTCGAGCGTCACTGATGAGTCTTTTGCAGACGAAACGCGCGT

CTTGCGTATACGTAATTTAGTTCTGGTATCTATGATGAGTTTATTGTTAATGTTTACAAC

TGAAATATGTTTTGTTGTTAAAATTAAATCACTCTAATACTCAAACAAATATTTTTT-CT

TTATACGTGTTGTTCAGTTCGTAGTTTTCTATGCTATGCTTTGTATACTGTTGTTTTTCT

GTTTTTT-CTCCTTTTTTT----GCCATGGCGTTGTCAGTTTATTTTCAACTTATGATAA

TAAATGTCCC-TGTGGTATATTT--AGCCTCTCTCTAAGACAGGCATCGTTTATTTATGT

CGAAA-CTTAAGCAATTTTATTC-A-TTTTATTTTT-CTAC------TTATTCA--GTTC

AGGAAGCCCAATCAGTAGCTTGTACATCATACTACTGTAGTAAGTTCTGTGGGTCTGCTG

GTTGCTCATTATATGGATGTTACCTACTTCATCCTGGCAAAATTTGCTACTGCCTTCATT

GTCGCAGAGCTGAGTCTCCATTGGCACTTTCTGGAAGCGCTAGGAATGTGAACGACAAGA

ACAACGAGATGGACAACTCTCCAGTGTAAGTACTCAATACTAGATTTCGTTACACAATAT

AAAATTT----------GAAAAAAAA------GTTTTCTACATGCCAGCATATATTTTAA

TTAAGGTGTTTACCAATTTTTGAAACAGATAATTTGAA-TTTTTTTAAAATAAAATATTT

CATCTT-----AATTCAATATGTCGTTATCGTCTGAATCGTATTA------TGCAGTCAT

TGAAAAAAAA--TAAACACCTGAACTACTCCTGCAAGTGAAAGAAAGGAT-GTATTT-TT

-CTGATATCGGTTAATGTGTTGGGAGGCAGATAAGATATCTTCTAGTGTTTCTCACAATT

-ATTTACATTCAAAAACATTTTTTT----ACATAAATCGATCACTGGTCAATTGCACGAG

AGTAGTCAGAAAAAAAACCCTGTTATATGAAACGTTTTAATTGAATTTGTGAAAATAA-C

ACCACATATAAGAGAACATCTACTTTGTTTCAGGATGAATGAGGTGGAAAATTTGGACCA

AGAAATGGATATGTTCTAG

>13_11Gen

ATGAAGGCAACGATCTTGTTAGCTGTTGTAGTGGCAGTCATTGTTGGAGGTAAATATCTT

TATTGAAATATAAATGTGTATGCGTATTTTAGAAGTATAGATCCAGCATTTTT-AAGATA

AAAATACGGCAGACCGACTTCTCCAAAATTTGATTGCGAATCCCATATT---------TG

TATATTATGTTAAATTATACTACTTTATTGTAATAATTTTTCATTTA---TAATAACTGT

TTTAAGTAAGAAAGCTGTAATTGGCAAAACTTTTAGGAATTTTGGTCCCCAATGCTCTTC

TTTTTTTAACTTTTATGATTCGAGCGTCACTGATGAGTCTTTTGCAGACGAAACGCGCGT

CTTGCGTATACGTAATTTAGTTCTGGTATCTATGATGAGTTTATTGTTAATGTTTACAAC

TGAAATATGTTTTGTTGTTAAAATTAAATCACTCTAATACTCAAACAAATATTTTTT-CT

TTATACGTGTTGTTCAGTTCGTAGTTTTCTATGCTATGCTTTGTATACTGTTGTTTTTCT

GTTTTTT-CTCCTTTTTTT----GCCATGGCGTTGTCAGTTTATTTTCAACTTATGATAA

TAAATGTCCC-TGTGGTATATTT--AGCCTCTCTCTAAGACAGGCATCGTT-ATTTATGT

CGAAA-CTTAAGCAATTTTATTC-A-TTTTATTTTT-CTAC------TTATTCA--GTTC

AGGAAGCCCAATCAGTAGCTTGTACATCATACTACTGTAGTAAGTTCTGTGGGTCTGCTG

GTTGCTCATTATATGGATGTTACCTACTTCATCCTGGCAAAATTTGCTACTGCCTTCATT

GTCGCAGAGCTGAGTCTCCATTGGCACTTTCTGGAAGCGCTAGGAATGTGAACGACAAGA

ACAACGAGATGGACAACTCTCCAGTGTAAGTACTCAATACTAGATTTCGTTACACAATAT

AAA-ATTT---------GAAAAAAAA------GTTTTCTACATGCCAGCATATATTTTAA

TTAAGGTGTTTACCAATTTTTGAAACAGATAATTTGAA-TTTTTTCAAAATAAAATATTT

CATCTT-----AATTCAATATGTCGTTATCGTCTGAATCGTATTA------TGCAGTCAT

TGAAAAAAAA--TAAACACCTGAACTACTCCTGCAAGTGAAAGAAAGGAT-GT-TTT-TT

TCTGATATCGGTTAATGTGTTGGGAGGCAGATAAGATATCTTCTAGTGTTTCTCACAATT

-ATTTACATTCAAAAACATTTTTTT----ACATAAATCGATCACTGGTCAATTGCACGAG

AGTAGTCAGAAAAAAAACCCTGTTATATGAAACGTTTTAATTGAATTTGTGAAAATAA-C

ACCACATATAAGAGAACATCTACTTTGTTTCAGGATGAATGAGGTGGAAAATTTGGACCA

AGAAATGGATATGTTCTAG

>13_12Gen

ATGAAGGCAACGATCTTGTTAGCTGTTGTAGTGGCAGTCATTGTTGGAGGTAAATATCTT

TATTGAAATATAAATGTGTATGCGTATTTTAGAAGTATAGATCCAGCATTTTT-AAGATA

AAAATACGGCAGACCGACTTCTCCAAAATTTGATTGCGAATCCCATATT---------TG

TATATTATGTTAAATTATACTACTTTATTGTAATAATTTTTCATTTA---TAATAACTGT

TTTAAGTAAGAAAGCTGTAATTGGCAAAACTTTTAGGAATTTTGGTCCCCAATGCTCTTC

TTTTTTTAACTTTTATGATTCGAGCGTCACTGATGAGTCTTTTGCAGACGAAACGCGCGT

CTTGCGTATACGTAATTTAGTTCTGGTATCTATGATGAGTTTATTGTTAATGTTTACAAC

TGAAATATGTTTTGTTGTTAAAATTAAATCACTCTAATACTCAAACAAATATTTTTT-CT

TTATACGTGTTGTTCAGTTCGTAGTTTTCTATGCTATGCTTTGTATACTGTTGTTTTTCT

GTTTTTT-CTCCTTTTTTT----GCCATGGCGTTGTCAGTTTATTTTCAACTTATGATAA

TAAATGTCCC-TGTGGTATATTT--AGCCTCTCTCTAAGACAGGCATCGTT-ATTTATGT

CGAAA-CTTAAGCAATTTTATTC-A-TTTTATTTTT-CTAC------TTATTCA--GTTC

AGGAAGCCCAATCAGTAGCTTGTACATCATACTACTGTAGTAAGTTCTGTGGGTCTGCTG

GTTGCTCATTATATGGATGTTACCTACTTCATCCTGGCAAAATTTGCTACTGCCTTCATT

GTCGCAGAGCTGAGTCTCCATTGGCACTTTCTGGAAGCGCTAGGAATGTGAACGACAAGA

ACAACGAGATGGACAACTCTCCAGTGTAAGTACTCAATACTAGATTTCGTTACACAATAT

AAAATTT----------GAAAAAAAA------GTTTTCTACATGCCAGCATATATTTTAA

TTAAGGTGTTTACCAAATTTTGAAACAGATAATTTGAA-TTTTTTTAAAATAAAATATTT

CATCTT-----AATTCAATATGTCGTTATCGTCTGAATCGTATTA------TGCAGTCAT

TGAAAAAAAA--TAAACACCTGAACTACTCCTGCAAGTGAAAGAAAGGAT-GT-TTT-TT

TCTGATATCGGTTAATGTGTTGGGAGGCAGATAAGATATCTTCTAGTGTTTCTCACAATT

-ATTTACATTCAAAAACATTTTTTT----ACATAAATCGATCACTGGTCAATTGCACGAG

AGTAGTCAGAAAAAAAACCCTGTTATATGAAACGTTTTAATTGAATTTGTGAAAATAA-C

ACCACATATAAGAGAACATCTACTTTGTTTCAGGATGAATGAGGTGGAAAATTTGGACCA

AGAAATGGATATGTTCTAG

>13_13Gen

ATGAAGGCAACGATCTTGTTAGCTGTTGTAGTGGCAGTCATTGTTGGAGGTAAATATCTT

TATTGAAATATAAATGTGTATGCGTATTTTAGAAGTATAGATCCAGCATTTTT-AAGATA

AAAATACGGCAGACCGACTTCTCCAAAATTTGATTGCGAATCCCATATT---------TG

TATATTATGTTAAATTATACTACTTTATTGTAATAATTTTTCATTTA---TAATAACTGT

TTTAAGTAAGAAAGCTGTAATTGGCAAAACTTTTAGGAATTTTGGTCCCCAATGCTCTTC

TTTTTTTAACTTTTATGATTCGAGCGTCACTGATGAGTCTTTTGCAGACGAAACGCGCGT

CTTGCGTATACGTAATTTAGTTCTGGTATCTATGATGAGTTTATTGTTAATGTTTACAAC

TGAAATATGTTTTGTTGTTAAAATTAAATCACTCTAATACTCAAACAAATATTTTTT-CT

TTATACGTGTTGTTCAGTTCGTAGTTTTCTATGCTATGCTTTGTATACTGTTGTTTTTCT

GTTTTTT-CTCCTTTTTTT----GCCATGGCGTTGTCAGTTTATTTTCAACTTATGATAA

TAAATGTCCC-TGTGGTATATTT--AGCCTCTCTCTAAGACAGGCATCGTT-ATTTATGT

CGAAA-CTTAAGCAATTTTATTC-A-TTTTATTTTT-CTAC------TTATTCA--GTTC

AGGAAGCCCAATCAGTAGCTTGTACATCATACTACTGTAGTAAGTTCTGTGGGTCTGCTG

GTTGCTCATTATATGGATGTTACCTACTTCATCCTGGCAAAATTTGCTACTGCCTTCATT

GTCGCAGAGCTGAGTCTCCATTGGCACTTTCTGGAAGCGCTAGGAATGTGAACGACAAGA

ACAACGAGATGGACAACTCTCCAGTGTAAGTACTCAATACTAGATTTCGTTACACAATAT

AAAATTT----------GAAAAAAAA------GTTTTCTACATGCCAGCATATATTTTAA

TTAAGGTGTTTACCAATTTTTGAAACAGATAATTTGAA-TTTTTTTAAAATAAAATATTT

CATCTT-----AATTCAATATGTCGTTATCGTCTGAATCGTATTA------TGCAGTCAT

TGAAAAAAAA--TAAACACCTGAACTACTCCTGCAAGTGAAAGAAAGGAT-GT-TTT-TT

TCTGATATCGGTTAATGTGTTGGGAGGCAGATAAGATATCTTCTAGTGTTTCTCACAATT

-ATTTACATTCAAAAACATTTTTTT----ACATAAATCGATCACTGGTCAATTGCACGAG

AGTAGTCAGAAAAAAAACCCTGGTATATGAAACGTTTTAATTGAATTTGTGAAAATAA-C

ACCACATATAAGAGAACATCTACTTTGTTTCAGGATGAATGAGGTGGAAAATTTGGACCA

AGAAATGGATATGTTCTAG

>13_18Gen

ATGAAGGCAACGATCTTGTTAGCTGTTGTAGTGGCAGTCATTGTTGGAGGTAAATATCTT

TATTGAAATATATCTGTGTGTACGTATTTTAGAAGTATAGATCCAGCATTTTT-AAGATA

AAAATACGGCAGACCGACTTCTTCAAAATTTGATTGCGAATCCCATATT---------TG

TATATTATGTTAAATTATACTACTTTATTGTAATAATTTTTCATTTA---TAATAACTGT

TTTAAGTAAGAAAGCTGTATTTGGCAAAACTGTTAGAAATTTTGGTCCTCAATGCTCTTC

TTTTTT-GACTTTTATGATTCGAGCGTCACTGATGAGTCTTTTGTAGACGAAACGCGCGT

CTGGCGTATACGTAATTTAGTCCGGGTATCTATGATGAGTTTATTGTTAATGTTTACAAC

TGAAATATGTTTTGTTATTAAAATTAAATCACTCAAATACTCAAACAAATATTTTTT-CT

TTATACGTGTTGTTCAGTTCGTAGTTTTCTATGCTATGCTTTGTATACTGTTGTTTGTCT

TTTTT---CTCCTTTTTTT----GACATGGCGTTGTCAGTTTATTTTCAACGTATGATAA

TAAATGTCCC-TGTGGTATATTT--AGCCTCTCTCTAAGACAGTCATCGTT-ATTTGTGT

CGAAA-CTTGAGCAATTTTATTC-A-TTTTATTTTT-CTAC------TTATTCA--GTTC

AGGAAGCCCAATCAGTAGCGTGTACATCATACTACTGTAGTAAGTTCTGTGGGTCTGCTG

GTTGCTCATTATATGGATGTTACCTACTTCATCCTGGCAAAATTTGCTACTGCCTTCATT

GTCGCAGAGCTGAGTCTCCATTGGCACTTTCTGGAAGCGCTAGGAATGTGAACGACAAGA

ACAACGAGATGGACAACTCTCCAGTGTAAGTACTCAATACTAGATTTCGTTACACAATAT

AAA-ATTT---------GAAAAAAAA------GTTTTCTACATGCCAACATATATTGTAA

TTAAGGTGTTTACCAATTTTTGAAACAGATAATTTGATCTTTTTTTTTAATAAAATATTT

CATCTT-----AATTCAATATGTCGTTATCGTTTGAATCGTATTA------TGCAGTCAT

TGAAAAAAAAAATAAACACCTGAACTACTCCTGCAAGAGAAAGAAGGGAT-GT-TTT-TT

TCTAAGATCGGTTAATGTGTTGGGAGGTAGATAAGATATCTTGTATTGTGTCTCTCAACT

-ATTTACATTCAAAAACATTTTT------ACATAAATCGATCACTGGTTAACTGCACGAG

AATAGTCAGAAAACAA-CCCTGTTATATGAAACGTTTTAATTGCATTTGTGAAAATAA-C

ACCACATATAAGAAAACATCTACTTTGTTTCAGGATGAATGAGGTGGAAAATTTGGACCA

AGAGATGGATATGTTCTAG

>13_21Gen

ATGAAGGCAACGATCTTGTTAGCTGTTGTAGTGGCAGTCGTTGTTGGAGGTAAATATCTT

TATTGAAATATATCTGTGTGTACGTATTTTAGAAGTATAGATCCAGCATTTTT-AAGATA

AAAATACGGCAGACCGACTTCTTCAAAATTTGATTGCGAATCCCATATT---------TG

TATATTATGTTAAATTATACTACTTTATTGTAATAATTTTTCATTTA---TAATAACTGT

TTTAAGTAAGAAAGCTGTATTTGGCAAAACTGTTAGAAATTTTGGTCCTCAATGCTCTTC

TTTTTT-GACTTTTATGATTCGAGCGTCACTGATGAGTCTTTTGTAGACGAAACGCGCGT

CTGGCGTATACGTAATTTAGTCCTGGTACCTATGATGAGTTTATTGTTAATGTTTACAAC

TGAAATATGTTTTGTTATTAAAATTAAATCACTCAAATACTCAAACAAATATTTTTT-CT

TTATACGTGTTGTTCGGTTCGTAGTTTTCTATGCTATGCTTTGTATACTGTTGTTTGTCT

TTTTT---CTCCTTTTTTT----GACATGGCGTTGTCAGTTTATTTTCAACGTATGATAA

TAAATGTCCC-TGTGGTATATTT--AGCCTCTCTCTAAGACAGTCATCGTT-ATTTGTGT

CGAAA-CTTGAGCAATTTTATTC-A-TTTTATTTTT-CTAC------TTATTCA--GTTC

AGGAAGCCCAATCAGTAGCGTGTACATCATACTACTGTAGTAAGTTCTGTGGGTCTGCTG

GTTGCTCATTATATGGATGTTACCTACTTCATCCTGGCAAAATTTGCTACTGCCTTCATT

GTCGCAGAGCTGAGTCTCCATTGGCACTTTCTGGAAGCGCTAGGAATGTGAACGACAAGA

ACAACGAGATGGACAACTCTCCAGTGTAAGTACTCAATACTAGATTTCGTTACACAATAT

AAA-ATTT---------GAAAAAAAA------GTTTTCTACATGCCAACATATATTGTAA

TTAAGGTGTTTACCAATTTTTGAAACAGATAATTTGATCTTTTTTTTTAATAAAATATTT

CATCTT-----AATTCAATATGTCGTTATCGTTTGAATCGTATTA------TGCAGTCAT

TGAAAAAAAAAATAAACACCTGAACTACTCCTGCAAGAGAAAGAAGGGAT-GT-TTT-TT

TCTAAGATCGGTTAATGTGTTGGGAGGTAGATAAGATATCTTGTATTGTGTCTCTCAACT

-ATTTACATTCAAAAACATTTTT------ACATAAATCGATCACTGGTTAACTGCACGAG

AATAGTCAGAAAACAA-CCCTGTTATATGAAACGTTTTAATTGAATTTGTGAAAATAA-C

ACCACATATAAGAAAACATCTACTTTGTTTCAGGATGAATGAGGTGGAAAATTTGGACCA

AGAAATGGATATGTTCTAG

>13_24Gen

ATGAAGGCAACGATCTTGTTAGCTGTTGTAGTGGCAGTCATTGTTGGAGGTAAATATCTT

TATTGAAATATATCTGTGTATGCGTATTTTAGAAGTATAGATCCAGCATTTTT-AAGATA

AAAATACGGCAGACCGACTTCTCCAAAATTTGATTGCGAATCCCATATT---------TG

TATATTATGTTAAATTATACTACTTTATTGTAATAATTTTTCATTTA---TAATAACTGT

TTTAAGTAAGAAAGCTGTAATTGGCAAAACTTTTAGGAATTTTGGTCCCCAATGCTCTTC

TTTTTTTAACTTTTATGATTCGAGCGTCACTGATGAGTCTTTTGCAGACGAAACGCGCGT

CTTGCGTATACGCAATTTAGTTCTGGTATCTATGATGAGTTTATTGTTAATGTTTACAAC

TGAAATATGTTTTGTTGTTAAAATTAAATCACTCTAATACTCAAACAAATATTTTTT-CT

TTATACGTGTTGTTCAGTTCGTAGTTTTCTATGCTATGCTTTGTATACTGTTGTTTTTCT

GTTTTTT-CTCCTTTTTTT----GCCATGGCGTTGTCAGTTTATTTTCAACTTATGATAA

TAAATGTCCC-TGTGGTATATTT--AGCCTCTCTCTAAGACAGGCATCGTT-ATTTATGT

CGAAA-CTTAAGCAATTTTATTC-A-TTTTATTTTT-CTAC------TTATTCA--GTTC

AGGAAGCCCAATCAGTAGCTTGTACATCATACTACTGTAGTAAGTTCTGTGGGTCTGCTG

GTTGCTCATTATATGGATGTTACCTACTTCATCCTGGCAAAATTTGCTACTGCCTTCATT

GTCGCAGAGCTGAGTCTCCATTGGCACTTTCTGGAAGCGCTAGGAATGTGAACGACAAGA

ACAACGAGATGGACAACTCTCCAGTGTAAGTACTCAATACTAGATTTCGTTACACAATAT

AAA-ATTT---------GAAAAAAAA------GTTTTCTACATGCCAGCATATATTTTAA

TTAAGGTGTTTACCAATTTTTGAAACAGATAATTTGAA-TTTTTTTAAAATAAAATATTT

CATCTT-----AATTCAATATGTCGTTATCGTTTGAATCGTATTA------TGCAGTCAT

TGAAAAAAAA--TAAACACCTGAACTACTCCTGCAAGTGAAAGAAAGGAT-GT-TTT-TT

TCTGATATCGGTTAATGTGTTGGGAGGCAGATAAGATATCTTCTAGTGTTTCTCACAATT

-ATTTACATTCAAAAACATTTTTTT----ACATAAATCGATCACTGGTCAATTGCACGAG

AGTAGTCAGAAAAAAAACCCTGTTATATGAAACGTTTTAATTGAATTTGTGAAAATAA-C

ACCACATATAAGAGAACATCTACTTTGTTTCAGGATGAATGAGGTGGAAAATTTGGACCA

AGAAATGGATATGTTCTAG

>13_25Gen

ATGAAGGCAACGATCTTGTTAGCTGTTGTAGTGGCAGTCATTGTTGGAGGTAAATATCTT

TATTGAAATATAAATGTGTATGCGTATTTTAGAAGTATAGATCCAGCATTTTT-AAGATA

AAAATACGGCAGACCGACTTCTCCAAAATTTGATTGCGAATCCCATATT---------TG

TATATTATGTTAAATTATACTACTTTATTGTAGTAATTTTTCATTTA---TAATAACTGT

TTTAAGTAAGAAAGCTGTAATTGGCAAAACTTTTAGGAATTTTGGTCCCCAATGCTCTTC

TTTTTTTAACTTTTATGATTCGAGCGTCACTGATGAGTCTTTTGCAGACGAAACGCGCGT

CTTGCGTATACGTAATTTAGTTCTGGTATCTATGATGAGTTTATTGTAAATGTTTACAAC

TGAAATATGTTTTGTTGTTAAAATTAAATCACTCTAATACTCAAACAAATATTTTTT-CT

TTATACGTGTTGTTCAGTTCGTAGTTTTCTATGCTATGCTTTGTATACTGTTGTTTTTCT

GTTTTTT-CTCCTTTTTTT----GCCATGGCGTTGTCAGTTTATTTTCAACTTATGATAA

TAAATGTCCC-TGTGGTATATTT--AGCCTCTCTCTAAGACAGGCATCGTT-ATTTATGT

CGAAA-CTTAAGCAATTTTATTC-A-TTTTATTTTT-CTAC------TTATTCA--GTTC

AGGAAGCCCAATCAGTAGCTTGTACATCATACTACTGTAGTAAGTTCTGTGGGTCTGCTG

GTTGCCCATTATATGGATGTTACCTACTTCATCCTGGCAAAATTTGCTACTGCCTTCATT

GTCGCAGAGCTGAGTCTCCATTGGCACTTTCTGGAAGCGCTAGGAATGTGAACGACAAGA

ACAACGAGATGGACAACTCTCCAGTGTAAGTACTCAATACTAGATTTCGTTACACAATAT

AAA-ATTT---------GAAAAAAA-------GTTTTCTACATGCCAGCATATATTTTAA

TTAAGGTGTCTACCAATTTTTGAAACAGATAATTTGAA-TTTTTTTAAAATAAAATATTT

CATCTT-----AATTCAATATGTCGTTATCGTCTGAATCGTATTA------TGCAGTCAT

TGAAAAAAA---TAAACACCTGAACTACTCCTGCAAGTGAAAGAAAGGAT-GT-TTT-TT

TCTGATATCGGTTAATGTGTTGGGAGGCAGATAAGATATCTTCTAGTGTTTCTCACAATT

-ATTTACATTCAAAAACATTTTTTT----ACATAAATCGATCACTGGTCAATTGCACGAG

AGTAGTCAGAAAAAAAACCCTGTTATATGAAACGTTTTAATTGAATTTGTGAAAATAA-C

ACCACATATAAGAGAACATCTACTTTGTTTCAGGATGAATGAGGTGGAAAATTTGGACCA

AGAAATGGATATGTTCTAG

>13_01cDNA

ATGAAGGCAACGATCTTGTTAGCTGTTGTAGTGGCAGTCATTGTTGGAG-----------

------------------------------------------------------------

------------------------------------------------------------

------------------------------------------------------------

------------------------------------------------------------

------------------------------------------------------------

------------------------------------------------------------

------------------------------------------------------------

------------------------------------------------------------

------------------------------------------------------------

------------------------------------------------------------

---------------------------------------------------------TTC

AGGAAGCCCAATCAGTAGCTTGTACATCATACTACTGTAGTAAGTTCTGTGGGTCTGCTG

GTTGCTCATTATATGGATGTTACCTACTTCATCCTGGCAAAATTTGCTACTGCCTTCATT

GTCGCAGAGCTGAGTCTCCATTGGCACTTTCTGGAAGCGCTAGGAATGTGAACGACAAGA

ACAACGAGATGGACAACTCTCCAGT-----------------------------------

------------------------------------------------------------

------------------------------------------------------------

------------------------------------------------------------

------------------------------------------------------------

------------------------------------------------------------

------------------------------------------------------------

------------------------------------------------------------

---------------------------------GATGAATGAGGTGGAAAATTTGGACCA

AGAAATGGATATGTTCTAG

>13_02cDNA

ATGAAGGCAACGATCTTGTTAGCTGTTGTAGTGGCAGTCATTGTTGGAG-----------

------------------------------------------------------------

------------------------------------------------------------

------------------------------------------------------------

------------------------------------------------------------

------------------------------------------------------------

------------------------------------------------------------

------------------------------------------------------------

------------------------------------------------------------

------------------------------------------------------------

------------------------------------------------------------

---------------------------------------------------------TTC

AGGAAGCCCAATCAGTAGCTTGTACATCATACTACTGTAGTAAGTTCTGTGGGTCTGCTG

GTTGCTCATTATATGGATGTTACCTACTTCATCCTGGCAAAATTTGCTACTGCCTTCATT

GTCGCAGAGCTGAGTCTCCATTGGCACTTTCTGGAAGCGCTAGGAATGTGAACGACAAGA

ACAACGAGATGGACAACTCTCCAGT-----------------------------------

------------------------------------------------------------

------------------------------------------------------------

------------------------------------------------------------

------------------------------------------------------------

------------------------------------------------------------

------------------------------------------------------------

------------------------------------------------------------

---------------------------------GATGAATGAGGTGGAAAATTTGGACCA

AGAAATGGATATGTTCTAG

>13_03cDNA

ATGAAGGCAACGATCTTGTTAGCTGTTGTAGTGGCAGTCATTGTTGGAG-----------

------------------------------------------------------------

------------------------------------------------------------

------------------------------------------------------------

------------------------------------------------------------

------------------------------------------------------------

------------------------------------------------------------

------------------------------------------------------------

------------------------------------------------------------

------------------------------------------------------------

------------------------------------------------------------

---------------------------------------------------------TTC

AGGAAGCCCAATCAGTAGCTTGTACATCATACTACTGTAGTAAGTTCTGTGGGTCTGCTG

GTTGCTCATTATATGGATGTTACCTACTTCATCCTGGCAAAATTTGCTACTGCCTTCATT

GTCGCAGAGCTGAGTCTCCATTGGCACTTTCTGGAAGCGCTAGGAATGTGAACGACAAGA

ACAACGAGATGGACAACTCTCCAGT-----------------------------------

------------------------------------------------------------

------------------------------------------------------------

------------------------------------------------------------

------------------------------------------------------------

------------------------------------------------------------

------------------------------------------------------------

------------------------------------------------------------

---------------------------------GATGAATGAGGTGGAAAATTTGGACCA

AGAAATGGATATGTTCTAG

>13_05cDNA

ATGAAGGCAACGATCTTGTTAGCTGTTGTAGTGGCAGTCATTGTTGGAG-----------

------------------------------------------------------------

------------------------------------------------------------

------------------------------------------------------------

------------------------------------------------------------

------------------------------------------------------------

------------------------------------------------------------

------------------------------------------------------------

------------------------------------------------------------

------------------------------------------------------------

------------------------------------------------------------

---------------------------------------------------------TTC

AGGAAGCCCAATCAGTAGCTTGTACATCATACTACTGTAGTAAGTTCTGTGGGTCTGCTG

GTTGCTCATTATATGGATGTTACCTACTTCATCCTGGCAAAATTTGCTACTGCCTTCATT

GTCGCAGAGCTGAGTCTCCATTGGCACTTTCTGGAAGCGCTAGGAATGTGAACGACTAGA

ACAACGAGATGGACAACTCTCCAGT-----------------------------------

------------------------------------------------------------

------------------------------------------------------------

------------------------------------------------------------

------------------------------------------------------------

------------------------------------------------------------

------------------------------------------------------------

------------------------------------------------------------

---------------------------------GATGAATGAGGTGGAAAATTTGGACCA

AGAAATGGGTATGTTCTAG

>13_06cDNA

ATGAAGGCAACGATCTTGTTAGCTGTTGTAGTGGCAGTCATTGTTGGAG-----------

------------------------------------------------------------

------------------------------------------------------------

------------------------------------------------------------

------------------------------------------------------------

------------------------------------------------------------

------------------------------------------------------------

------------------------------------------------------------

------------------------------------------------------------

------------------------------------------------------------

------------------------------------------------------------

---------------------------------------------------------TTC

AGGAAGCCCAATCAGTAGCGTGTACATCATACTACTGTAGTAAGTTCTGTGGGTCTGCTG

GTTGCTCATTATATGGATGTTACCTACTTCATCCTGGCAAAATTTGCTACTGCCTTCATT

GTCGCAGAGCTGAGTCTCCATTGGCACTTTCTGGAAGCGCTAGGAATGTGAACGACAAGA

ACAACGAGATGGACAACTCTCCAGT-----------------------------------

------------------------------------------------------------

------------------------------------------------------------

------------------------------------------------------------

------------------------------------------------------------

------------------------------------------------------------

------------------------------------------------------------

------------------------------------------------------------

---------------------------------GATGAATGAGGTGGAAAATTTGGACCA

AGAAATGGATATGTTCTAG

>13_07cDNA

ATGAAGGCAACGATCTTGTTAGCTGTTGTAGTGGCAGTCATTGTTGGAG-----------

------------------------------------------------------------

------------------------------------------------------------

------------------------------------------------------------

------------------------------------------------------------

------------------------------------------------------------

------------------------------------------------------------

------------------------------------------------------------

------------------------------------------------------------

------------------------------------------------------------

------------------------------------------------------------

---------------------------------------------------------TTC

AGGAAGCCCAATCAGTAGCTTGTACATCATACTACTGTAGTAAGTTCTGTGGGTCTGCTG

GTTGCTCATTATATGGATGTTACCTACTTCATCCTGGCAAAATTTGCTACTGCCTTCATT

GTCGCAGAGCTGAGTCTCCATTGGCACTTTCTGGAAGCGCTAGGAATGTGAACGACAAGA

ACAACGAGATGGACAACTCTCCAGT-----------------------------------

------------------------------------------------------------

------------------------------------------------------------

------------------------------------------------------------

------------------------------------------------------------

------------------------------------------------------------

------------------------------------------------------------

------------------------------------------------------------

---------------------------------GATGAATGAGGTGGAAAATTTGGACCA

AGAAATGGATATGTTCTAG

>13_08cDNA

ATGAAGGCAACGATCTTGTTAGCTGTTGTAGTGGCAGTCATTGTTGGAG-----------

------------------------------------------------------------

------------------------------------------------------------

------------------------------------------------------------

------------------------------------------------------------

------------------------------------------------------------

------------------------------------------------------------

------------------------------------------------------------

------------------------------------------------------------

------------------------------------------------------------

------------------------------------------------------------

---------------------------------------------------------TTC

AGGAAGCCCAATCAGTAGCTTGTACATCATACTACTGTAGTAAGTTCTGTGGGTCTGCTG

GTTGCTCATTATATGGATGTTACCTACTTCATCCTGGCAAAATTTGCTACTGCCTTCATT

GTCGCAGAGCTGAGTCTCCATTGGCACTTTCTGGAAGCGCTAGGAATGTGAACGACAAGA

ACAACGAGATGGATAACTCTCCAGT-----------------------------------

------------------------------------------------------------

------------------------------------------------------------

------------------------------------------------------------

------------------------------------------------------------

------------------------------------------------------------

------------------------------------------------------------

------------------------------------------------------------

---------------------------------GATGAATGAGGTGGAAAATTTGGACCA

AGAAATGGATATGTTCTAG

>13_09cDNA

ATGAAGGCAACGATCTTGTTAGCTGTTGTAGTGGCAGTCATTGTTGGAG-----------

------------------------------------------------------------

------------------------------------------------------------

------------------------------------------------------------

------------------------------------------------------------

------------------------------------------------------------

------------------------------------------------------------

------------------------------------------------------------

------------------------------------------------------------

------------------------------------------------------------

------------------------------------------------------------

---------------------------------------------------------TTC

AGGAAGCCCAATCAGTAGCGTGTACATCATACTACTGTAGTAAGTTCTGTGGGTCTGCTG

GTTGCTCATTATATGGATGTTACCTACTTCATCCTGGCAAAATTTGCTACTGCCTTCATT

GTCGCAGAGCTGAGTCTCCATTGGCACTTTCTGGAAGCGCTAGGAATGTGAACGACAAGA

ACAACGAGATGGACAACTCTCCAGT-----------------------------------

------------------------------------------------------------

------------------------------------------------------------

------------------------------------------------------------

------------------------------------------------------------

------------------------------------------------------------

------------------------------------------------------------

------------------------------------------------------------

---------------------------------GATGAATGAGGTGGAAAATTTGGACCA

AGAAATGGATATGTTCTAG

>13_10cDNA

ATGAAGGCAACGATCTTGTTAGCTGTTGTAGTGGCAGTCATTGTTGGAG-----------

------------------------------------------------------------

------------------------------------------------------------

------------------------------------------------------------

------------------------------------------------------------

------------------------------------------------------------

------------------------------------------------------------

------------------------------------------------------------

------------------------------------------------------------

------------------------------------------------------------

------------------------------------------------------------

---------------------------------------------------------TTC

AGGAAGCCCAATCAGTAGCGTGTACATCATACTACTGTAGTAAGTTCTGTGGGTCTGCTG

GTTGCTCATTATATGGATGTTACCTACTTCATCCTGGCAAAATTTGCTACTGCCTTCATT

GTCGCAGAGCTGAGTCTCCATTGGCACTTTCTGGAAGCGCTAGGAATGTGAACGACAAGA

ACAACGAGATGGACAACCCTCCAGT-----------------------------------

------------------------------------------------------------

------------------------------------------------------------

------------------------------------------------------------

------------------------------------------------------------

------------------------------------------------------------

------------------------------------------------------------

------------------------------------------------------------

---------------------------------GATGAATGAGGTGGAAAATTTGGACCA

AGAAATGGATATGTTCTAG

>14_01Gen

ATGAAGGCAACGATCTTGTTAGCTGTTGTAGTGGTAGTCATTGTTGGAGGTAAATATCTT

TATTGAAATATATCTGCGTGTACGTATTTTAGAAGTATAGATCCAACATTTTTTAAGATA

AAAATACGGCAGACCGACTTTCCCAAAATTTGATTGCGAATCCCATATT---------TG

TATATTATGTTAAATTATACTACTTTATTGTAATAATTTTTCATTTA---TAATAACTGT

TTTAAGTAAGAAAC----------------------------------------------

------------------------------------------------------------

-------------------------------------------TTGTTAATGTTTACAAC

TGAAATATGTTTTGTTATTAAAATTAAATCATTCAAATATTCAAACAAATATATTTT-CT

TTATACGTGTTGTTCAGTTCGTAGTTTTCTATGCTATGCTTTGTATACTGGTTTGTTTTT

TTTT----CTCCTTTTTTT----GCCATGGCGTTGTCAGTTTATTTTCAACTTAAGATAA

TAAATGTCCC-TGTGGTATATTT--AGCCTCTCTCTAAGACAGGCATCGTT-ATTTGTGT

CGAAA-CTTAAGCAATTTTATTC-A-TTTTATTTTT-CTAC------TTATTCA--GTTC

AGGAAGCCCAATCAATTCCTTGTACATCATACTACTGTAGTAAGTTCTGTGGGTTAGGTG

GTTGCTCATTATATGGATGTTACAAACTTCATCCCGGCAAAATTTGCTACTGCCTTCATT

GTCGCAGAGCTGAGTCTCCATTGGCACTTTCTGGAAGCGCTAGGAATGTGAACGAGCAGA

ACAAAGAGATGGACAACTCTCCAGTGTAAGTACTCAATACTAGATTTCGTTACACAATAT

AAACATTTAAAA-TTT-GAAAAAACA------GTTTTCTTCATGCCAACATATATTGTAA

TTAAGGTGTTTACCAATTTTTGAAACAGATAATTTGAT-TTTTTTTTAAATTAAATATTT

CATCTT-----AATTCAATATGTCGTTATCGTCTGAATCGTATTA------TGCAGTCAT

TGAAAAAAAAA-TAAACACCTGAACTACTCCTGCAAAAGAAGGAAGGGAT-GT-TTT-GT

-TTTATATTGGTTAATGTGTTGGGAGGCAGATAAGATATCTTCTAGTGTGTCTCTCAATT

-ATTTACATTCGAAAACATTTTTTTT---ACATAAATCGATCACTGGTCAATTGCACGAG

AGTAGTCAGAAAAAAAC---TGTTATATAAAACGTTTTAATTGAATTTGTGAAAATAA-C

ACCACATATAAGAGAACATCTACTTTGTTTCAGGATGAATGAGGTGGACAATTTGGACCA

AGAAATGGATATGTTCTAG

>14_02Gen

ATGAAGGCAACGATCGTGTTAGCTGTTGTAGTGGCAGTCATTGTTGGGGGTAAATATCTT

TATTGAAATATATCTGTGTGTACGTATTTTAGAAGTATAGATCCAGCATTTTT-AAGATA

AAAATACGGCAGACCGACTTCTCCAAAATTTGATTGCGAATCACATATTGTAAATT--TA

TGTATTATGTTAAATTATACTACTTTATTGTAATAATTTTTCATTTA---TAATAACTGT

TTTAAGTAAGAAAGCTGTATTTGGCAAAACTTTTAGGAATTTTGGTCCTCAATGCTCTTC

TTTTTT-AACTTTTATGATTCGAGCGACACTGATGAGTCTTTTGTAGACGAAACGCGCGT

CTGGCGTATACGTAATTTAGTTCTGGTATCTATGATGAGTTTATTGTTAATGTTTACAAC

TGAAATATGTTTTGTTATTAAAATTAAATCACTCTAATACTCAAACAAATATTTTTT-CT

TTATACGTGTTGTTCAGTTCGTAGTTTTCTATTCTATGCTTTGTATACTGTTGTTTTTCT

TTTTTTT-CTCTTTTTTTTT---GCCATGGCGTTGTCAGTTTATTTTCAACTTATGATAA

TAAATGTCCC-TGTGGTATATTT--AGCCTCTCTCTAAGACAGGCATCG---ATTTGTGT

CGAAA-CTTAAGCAATTTTATTC-A-TTTTATTTTT-CTAC------TTATTCA--GTTC

AGGAAGCCCAATCAATTCCTTGTACATCATACTACTGTAGTAAGTTCTGTGGGTCTGCTG

GTTGCTCATTATATGGATGTTACAAACTTCATCCCGGCAAAATTTGCTACTGCCTTCATT

GTCGCAGAGCTGAGTCTCCATTGGCACTTTCTGGAAGCGCTAGGAATGTGAACGAGCAGA

ACAAAGAGATGGACAACTCTCCAGTGTAAGTACTCAATACTAGATTTCGTTACACAATAT

AAACATTTAAAA-TTT-GAAAAAACA------GTTTTCTTCATGCCAACATATATTGTAA

TTAAGGTGTTTACCAATTTTTGAAACAGATAATTTGAT-TTTTTTTTAAATAAAATATTT

CATCTT-----AATTCAATATGTCGTTATCGTTTGAATCGTATTACATGTATGCAGTCAT

TGAAAAAAACA-TAAACACCTGAACTACTCCTGCAAGAGAAAGAAGGGAT-GT-TTT-TT

TCTTAGATCTGTTAATGTGTTGGGAGGCAGATAAGATATCTTCTAGTGTGTCTCTCAACT

-ATTTACATTCAAAAACATTTTTTTTTTTACATTAATCGATCACTGGTCAATTGCACGAG

AGTAGTCAGAAAAAAAACCCTGTTACATGAAACGTTTTAATTGAATTTGTGAAAATAA-C

ACCACATATAAGAGAACATCTACTTTGTTTCAGGATGAATGAGATAGAAAATTTGGACCA

AGAAATGAATATGTTCTAG

>14_04Gen

ATGAAGGCAACGATCTTGTTAGCTGTTGTAGTGGTAGTCATTGTTGGAGGTAAATATCTT

TATTGAAATATATCTGCGTGTACGTATTTTAGAAGTATAGATCCAACATTTTTTAAGATA

AAAATACGGCAGACCGACTTTCCCAAAATTTGATTGCGAATCCCATATT---------TG

TATATTATGTTAAATTATACTACTTTATTGTAATAATTTTTCATTTA---TAATAACTGT

TTTAAGTAAGAAAC----------------------------------------------

------------------------------------------------------------

-------------------------------------------TTGTTAATGTTTACAAC

TGAAATATGTTTTGTTATTAAAATTAAATCATTCAAATATTCAAACAAATATATTTT-CT

TTATACGTGTTGTTCAGTTCGTAGTTTTCTATGCTATGCTTTGTATACTGGTTTGTTTTT

TTTT----CTCCTTTTTTT----GCCATGGCGTTGTCAGTTTATTTTCAACTTAAGATAA

TAAATGTCCC-TGTGGTATATTT--AGCCTCTCTCTAAGACAGGCATCGTT-ATTTGTGT

CGAAA-CTTAAGCAATTTTATTC-A-TTTTATTTTT-CTAC------TTATTCA--GTTC

AGGAGGCCCAATCAATTCCTTGTACATCATACTACTGTAGTAAGTTCTGTGGGTTAGGTG

GTTGCTCATTATATGGATGTTACAAACTTCATCCCGGCAAAATTTGCTACTGCCTTCATT

GTCGCAGAGCTGAGTCTCCATTGGCACTTTCTGGAAGCGCTAGGAATGTGAACGAGCAGA

ACAAAGAGATGGACAACTCTCCAGTGTAAGTACTCAATACTAGATTTCGTTACACAATAT

AAACATTTAAAA-TTT-GAAAAAACA------GTTTTCTTCATGCCAACATATATTGTAA

TTAAGGTGTTTACCAATTTTTGAAACAGATAATTTGAT-TTTTTTTTAAATTAAATATTT

CATCTT-----AATTCAATATGTCGTTATCGTCTGAATCGTATTA------TGCAGTCAT

TGAAAAAAAAA-TAAACACCTGAACTACTCCTGCAAAAGAAGGAAGGGAT-GT-TTT-GT

-TTTATATTGGTTAATGTGTTGGGAGACAGATAAGATATCTTCTAGTGTGTCTCTCAATT

-ATTTACATTCAAAAACATTTTTTTT---ACATAAATCGATCACTGGTCAATTGCACGAG

AGTAGTCAGAAAAAAAC---TGTTATATAAAACGTTTTAATTGAATTTGTGAAAATAA-C

ACCACATATAAGAGAACATCTACTTTGTTTCAGGATGAATGAGGTGGACAATTTGGACCA

AGAAATGGATATGTTCTAG

>14_05Gen

ATGAAGGCAACGATCTTGTTAGCTGTTGTAGTGGTAGTCATTGTTGGAGGTAAATATCTT

TATTGAAATATATCTGCGTGTACGTATTTTAGAAGTATAGATCCAACATTTTTTAAGATA

AAAATACGGCAGACCGACTTTCCCAAAATTTGATTGCGAATCCCATATT---------TG

TATATTATGTTAAATTATACTACTTTATTGTAATAATTTTTCATTTA---TAATAACTGT

TTTAAGTAAGAAAC----------------------------------------------

------------------------------------------------------------

-------------------------------------------TTGTTAATGTTTACAAC

TGAAATATGTTTTGTTATTAAAATTAAATCATTCAAATATTCAAACAAGTATATTTT-CT

TTATACGTGTTGTTCAGTTCGTAGTTTTCTATGCTATGCTTTGTATACTGGTTTGTTTTT

TTTT----CTCCTTTTTTT----GCCATGGCGTTGTCAGTTTATTTTCAACTTAAGATAA

TAAATGTCCC-TGTGGTATATTT--AGCCTCTCTCTAAGACAGGCATCGTT-ATTTGTGT

CGAAA-CTTAAGCAATTTTATTC-A-TTTTATTTTT-CTAC------TTATTCA--GTTC

AGGAAGCCCAATCAATTCCTTGTACATCATACTACTGTAGTAAGTTCTGTGGGTTAGGTG

GTTGCTCATTATATGGATGTTACAAACTTCATCCCGGCAAAATTTGCTACTGCCTTCATT

GTCGCAGAGCTGAGTCTCCATTGGCACTTTCTGGAAGCGCTAGGAATGTGAACGAGCAGA

ACAAAGAGATGGACAACTCTCCAGTGTAAGTACTCAATACTAGATTTCGTTACACAATAT

AAACATTTAAAG-TTT-GAAAAAACA------GTTTTCTTCATGCCAACATATATTGTAA

TTAAGGTGTTTACCAATTTTTGAAACAGATAATTTGAT-TTTTTTTTAAATTAAATATTT

CATCTT-----AATTCAATATGTCGTTATCGTCTGAATCGTATTA------TGCAGTCAT

TGAAAAAAAAA-TAAACACCTGAACTACTCCTGCAAAAGAAGGAAGGGAT-GT-TTT-GT

-TTTATATTGGTTAATGTGTTGGGAGGCAGATAAGATATCTTCTAGTGTGTCTCTCAATT

-ATTTACATTCAAAAACATTTTTTTT---ACATAAATCGATCACTGGTCAATTGCACGAG

AGTAGTCAGAAAAAAAC---TGTTATATAAAACGTTTTAATTGAATTTGTGAAAATAA-C

ACCACATATAAGAGAACATCTACTTTGTTTCAGGATGAATGAGGTGGACAATTTGGACCA

AGAAATGGATATGTTCTAG

>14_12Gen

ATGAAGGCAACGATCTTGTTAGCTGTTGTAGTGGTAGTCATTGTTGGAGGTAAATATCTT

TATTGAAATATATCTGCGTGTACGTATTTTAGAAGTATAGATCCAACATTTTTTAAGATA

AAAATACGGCAGACCGACTTTCCCAAAATTTGATTGCGAATCCCATATT---------TG

TATATTATGTTAAATTATACTACTTTATTGTAATAATTTTTCATTTA---TAATAACTGT

TTTAAGTAAGAAAC----------------------------------------------

------------------------------------------------------------

-------------------------------------------TTGTTAATGTTTACAAC

TGAAATATGTTTTGTTATTAAAATTAAATCATTCAAATATTCAAACAAATATATTTT-CT

TTATACGTGTTGTTCAGTTCGTAGTTTTCTATGCTATGCTTTGTATACTGGTTTGTTTTT

TGTT----CTCCTTTTTTT----GCCATGGCGTTGTCAGTTTATTTTCAACTTAAGATAA

TAAATGTCCC-TGTGGTATATTT--AGCCTCTCTCTAAGACAGGCATCGTT-ATTTGTGT

CGAAA-CTTAAGCAATTTTATTC-A-TTTTATTTTT-CTAC------TTATTCA--GTTC

AGGAAGCCCAATCAATTCCTTGTACATCATACTACTGTAGTAAGTTCTGTGGGTTAGGTG

GTTGCTCATTATATGGATGTTACAAACTTCATCCCGGCAAAATTTGCTACTGCCTTCATT

GTCGCAGAGCTGAGTCTCCATTGGCACTTTCTGGAAGCGCTAGGAATGTGAACGAGCAGA

ACAAAGAGATGGACAACTCTCCAGTGTAAGTACTCAATACTAGATTTCGTTACACAATAT

AAACATTTAAAA-TTT-GAAAAAACA------GTTTTCTTCATGCCAACATATATTGTAA

TTAAGGTGTTTACCAATTTTTGAAACAGATAATTTGAT-TTTTTTTTAAATTAAATATTT

CATCTT-----AATTCAATATGTCGTTATCGTCTGAATCGTATTA------TGCAGTCAT

TGAAAAAAAAA-TAAACACCTGAACTACTCCTGCAAAAGAAGGAAGGGAT-GT-TTT-GT

-TTTATATTGGTGAATGTGTTGGGAGGCAGATAAGATATCTTCTAGTGTGTCTCTCAATT

-ATTTACATTCAAAAACATTTTTTTT---ACATAAATCGATCACTGGTCAATTGCACGAG

AGTAGTCAGAAAAAAAC---TGTTATATAAAACGTTTTAATTGAATTTGTGAGAATAA-C

ACCACATATAAGAGAACATCTACTTTGTTCCAGGATGAATGAGGTGGACAATTTGGACCA

AGAAATGGATATGTTCTAG

>14_14Gen

ATGAAGGCAACGATCTTGTTAGCTGTTGTAGTGGTAGTCATTGTTGGAGGTAAATATCTT

TATTGAAATATATCTGCGTGTACGTATTTTAGAAGTATAGATCCAACATTTTTTAAGATA

AAAATACGGCAGACCGACTTTCCCAAAATTTGATTGCGAATCCCATATT---------TG

TATATTATGTTAAATTATACTACTTTATTGTAATAATTTTTCATTTA---TAATAACTGT

TTTAAGTAAGAAAC----------------------------------------------

------------------------------------------------------------

-------------------------------------------TTGTTAATGTTTACAAC

TGAAATATGTTTTGTTATTAAAATTAAATCATTCAAATATTCAAACAAATATATTTT-CT

TTATACGTGTTGTTCAGTTCGTAGTTTTCTATGCTATGCTTTGTATACTGGTTTGTTTTT

TTTT----CTCCTTTTTTT----GCCATGGCGTTGTCAGTTTATTTTCAACTTAAGATAA

TAAATGTCCC-TGTGGTATATTT--AGCCTCTCTCTAAGACAGGCATCGTT-ATTTGTGT

CGAAA-CTTAAGCAATTTTATTC-A-TTTTATTTTT-CTAC------TTATTCA--GTTC

AGGAAGCCCAATCAATTCCTTGTACATCATACTACTGTAGTAAGTTCTGTGGGTTAGGTG

GTTGCTCATTATATGGATGTTACAAACTTCATCCCGGCAAAATTTGCTACTGCCTTCATT

GTCGCAGAGCTGAGTCTCCATTGGCACTTTCTGGAAGCGCTAGGAATGTGAACGAGCAGA

ACAAAGAGATGGACAACTCTCCAGTGTAAGTACTCAATACTAGATTTCGTTACACAATAT

AAACATTTAAAA-TTT-GAAAAAACA------GTTTTCTTCATGCCAACATATATTGTAA

TTAAGGTGTTTACCAATTTTTGAAACAGATAATTTGAT-TTTTTTTTAAATTAAATATTT

CATCTT-----AATTCAATATGTCGTTATCGTCTGAATCGTATTA------TGCAGTCAT

TGAAAAAAAAA-TAAACACCTGAACTACTCCTGCAAAAGAAGGAAGGGAT-GT-TTT-GT

-TTTATATTGGTTAATGTGTTGGGAGGCAGATAAGATATCTTCTAGTGTGTCTCTCAATT

-ATTTACATTCGAAAACATTTTTTTT---ACATAAATCGATCACTGGTCAATTGCACGAG

AGTAGTCAGAAAAAAAC---TGTTATATAAAACGTTTTAATTGAATTTGTGAAAATAA-C

ACCACATATAAGAGAACATCTACTTTGTTTCAGGATGAATGAGGTGGACAATTTGGACCA

AGAAATGGATATGTTCTAG

>14_16Gen

ATGAAGGCAACGATCTTGTTAGCTGTTGTAGTGGTAGTCATTGTTGGAGGTAAATATCTT

TATTGAAATATATCTGCGTGTACGTATTTTAGAAGTATAGATCCAACATTTTTTAAGATA

AAAATACGGCAGACCGACTTTCCCAAAATTTGATTGCGAATCCCATATT---------TG

TATATTATGTTAAATTATACTACTTTATTGTAATAATTTTTCATTTA---TAATAACTGT

TTTAAGTAAGAAAC----------------------------------------------

------------------------------------------------------------

-------------------------------------------TTGTTAATGTTTACAAC

TGAAATATGTTTTGTTATTAAAATTAAATCATTCAAATATTCAAACAAATATATTTT-CT

TTATACGTGTTGTTCAGTTCGTAGTTTTCTATGCTATGCTTTGTATACTGGTTTGTTTTT

TTTT----CTCCTTTTTTT----GCCATGGCGTTGTCAGTTTATTTTCAACTTAAGATAA

TGAATGTCCC-TGTGGTATATTT--AGCCTCTCTCTAAGACAGGCATCGTT-ATTTGTGT

CGAAA-CTTAAGCAATTTTATTC-A-TTTTATTTTT-CTAC------TTATTCA--GTTC

AGGAAGCCCAATCAATTCCTTGTACATCATACTACTGTAGTAAGTTCTGTGGGTTAGGTG

GTTGCTCATTATATGGATGTTACAAACTTCATCCCGGCAAAATTTGCTACTGCCTTCATT

GTCGCAGAGCTGAGTCTCCATTGGCACTTTCTGGAAGCGCTAGGAATGTGAACGAGCAGA

ACAAAGAGATGGACAACTCTCCAGTGTAAGTACTCAATACTAGATTTCGTTACACAATAT

AAACATTTAAAA-TTT-GAAAAAACA------GTTTTCTTCATGCCAACATATATTGTAA

TTAAGGTGTTTACCAATTTTTGAAACAGATAATTTGAT-TTTTTTTTAAATTAAATATTT

CATCTT-----AATTCGATATGTCGTTATCGTCTGAATCGTATTA------TGCAGTCAT

TGAAAAAAAAA-TAAACACCTGAACTACTCCTGCAAAAGAAGGAAGGGAT-GT-TTT-GT

-TTTATATTGGTTAATGTGTTGGGAGGCAGATAAGATATCTTCTAGTGTGTCTCTCAATT

-ATTTACATTCAAAAACATTTTTTTT---ACATAAATCGATCACTGGTCAATTGCACGAG

AGTAGTCAGAAAAAAAC---TGTTATATAAAACGTTTTAATTGAATTTGTGAAAATAA-C

ACCACATATAAGAGAACATCTACTTTGTTTCAGGATGAATGAGGTGGACAATTTGGACCA

AGAAATGGATATGTTCTAG

>14_17Gen

ATGAAGGCAACGATCTTGTTAGCTGTTGTAGTGGTAGTCATTGTTGGAGGTAAATATCTT

TATTGAAATATATCTGCGTGTACGTATTTTAGAAGTATAGATCCAACATTTTTTAAGATA

AAAATACGGCAGACCGACTTTCCCAAAATTTGATTGCGAATCCCATATT---------TG

TATATTATGTTAAATTATACTACTTTATTGTAATAATTTTTCATTTA---TAATAACTGT

TTTAAGTAAGAAAC----------------------------------------------

------------------------------------------------------------

-------------------------------------------TTGTTAATGTTTACAAC

TGAAATATGTTTTGTTATTAAAATTAAATCATTCAAATATTCAAACAAATATATTTT-CT

TTATACGTGTTGTTCAGTTCGTAGTTTTCTATGCTATGCTTTGTATACTGGTTTGTTTTT

TTTT----CTCCTTTTTTT----GCCATGGCGTTGTCAGTTTATTTTCAACTTAAGATAA

TAAATGTCCC-TGTGGTATATTT--AGCCTCTCTCTAAGACAGGCATCGTT-ATTTGTGT

CGAAA-CTTAAGCAATTTTATTC-A-TTTTATTTTT-CTAC------TTATTCA--GTTC

AGGAAGCCCAATCAATTCCTTGTACATCATACTACTGTAGTAAGTTCTGTGGGTTAGGTG

GTTGCTCATTATATGGATGTTACAAACTTCATCCCGGCAAAATTTGCTACTGCCTTCATT

GTCGCAGAGCTGAGTCTCCATTGGCACTTTCTGGAAGCGCTAGGAATGTGAACGAGCAGA

ACAAAGAGATGGACAACTCTCCAGTGTAAGTACTCAATACTAGATTTCGTTACACAATAT

AAACATTTAAAA-TTT-GAAAAAACA------GTTTTCTTCATGCCAACATATATTGTAA

TTAAGGTGTTTACCAATTTTTGAAACAGATAATTTGAT-TTTTTTTTAAATTAAATATTT

CATCTT-----AATTCAATATGTCGTTATCGTCTGAATCGTATTA------TGCAGTCAT

TGAAAAAAAAA-TAAACACCTGAACTACTCCTGCAAAAGAAGGAAGGGAT-GT-TTT-GT

-TTTATATTGGTTAATGTGTTGGGAGGCAGATAAGATATCTTCTAGTGTGTCTCTCAATT

-ATTTACATTCGAAAACATTTTTTTT---ACATAAATCGATCACTGGTCAATTGCACGAG

AGTAGTCAGAAAAAAAC---TGTTATATAAAACGTTTTAATTGAATTTGTGAAAATAA-C

ACCACATATAAGAGAACATCTACTTTGTTTCAGGATGAATGAGGTGGACAATTTGGACCA

AGAAATGGATATGTTCTAG

>14_01cDNA

ATGAAGGCAACGATCGTGTTAGCTGTTGTAGTGGCAGTCATTGTTGGGG-----------

------------------------------------------------------------

------------------------------------------------------------

------------------------------------------------------------

------------------------------------------------------------

------------------------------------------------------------

------------------------------------------------------------

------------------------------------------------------------

------------------------------------------------------------

------------------------------------------------------------

------------------------------------------------------------

---------------------------------------------------------TTC

AGGAAGCCCAATCAATTCCTTGTACATCATACTACTGTAGTAAGTTCTGTGGGTCTGCTG

GTTGCTCATTATATGGATGTTACAAACTTCATCCCGGCAAAATTTGCTACTGCCTTCATT

GTCGCAGAGCTGAGTCTCCATTGGCACTTTCTGGGGGCGCTAGGAATGTGAACGAGCAGA

ACAAAGAGATGGACAACTCTCCAGT-----------------------------------

------------------------------------------------------------

------------------------------------------------------------

------------------------------------------------------------

------------------------------------------------------------

------------------------------------------------------------

------------------------------------------------------------

------------------------------------------------------------

---------------------------------GATGAATGAGGTGGACAATTTGGACCA

AGAAATGGATATGTTCTAG

>14_02cDNA

ATGAAGGCAACGATCTTGTTAGCTGTTGTAGTGGTAGTCATTGTTGGAG-----------

------------------------------------------------------------

------------------------------------------------------------

------------------------------------------------------------

------------------------------------------------------------

------------------------------------------------------------

------------------------------------------------------------

------------------------------------------------------------

------------------------------------------------------------

------------------------------------------------------------

------------------------------------------------------------

---------------------------------------------------------TTC

AGGAGGCCCAATCAATTCCTTGTACATCATACTACTGTAGTAAGTTCTGTGGGTTAGGTG

GTTGCTCATTATATGGATGTTACAAACTTCATCCCGGCAAAATTTGCTACTGCCTTCATT

GTCGCAGAGCTGAGTCTCCATTGGCACTTTCTGGAAGCGCTAGGAATGTGAACGAGCAGA

ACAAAGAGATGGACAACTCTCCAGT-----------------------------------

------------------------------------------------------------

------------------------------------------------------------

------------------------------------------------------------

------------------------------------------------------------

------------------------------------------------------------

------------------------------------------------------------

------------------------------------------------------------

---------------------------------GATGAATGAGGTGGACAATTTGGACCA

AGAAATGGATATGTTCTAG

>14_03cDNA

ATGAAGGCAACGATCTTGTTAGCTGTTGTAGTGGTAGTCATTGTTGGAG-----------

------------------------------------------------------------

------------------------------------------------------------

------------------------------------------------------------

------------------------------------------------------------

------------------------------------------------------------

------------------------------------------------------------

------------------------------------------------------------

------------------------------------------------------------

------------------------------------------------------------

------------------------------------------------------------

---------------------------------------------------------TTC

AGGAAGCCCAATCAATTCCTTGTACATCATACTACTGTAGTAAGTTCTGTGGGTTAGGTG

GTTGCTCATTATATGGATGTTACAAACTTCATCCCGGCAAAATTTGCTACTGCCTTCATT

GTCGCAGAGCTGAGTCTCCATTGGCACTTTCTGGAAGCGCTAGGAATGTGAACGAGCAGA

ACAAAGAGATGGACAACTCTCCAGT-----------------------------------

------------------------------------------------------------

------------------------------------------------------------

------------------------------------------------------------

------------------------------------------------------------

------------------------------------------------------------

------------------------------------------------------------

------------------------------------------------------------

---------------------------------GATGAATGAGGTGGACAATTTGGACCA

AGAAATGGATATGTTCTAG

>14_04cDNA

ATGAAGGCAACGATCTTGTTAGCTGTTGTAGTGGTAGTCATTGTTGGAG-----------

------------------------------------------------------------

------------------------------------------------------------

------------------------------------------------------------

------------------------------------------------------------

------------------------------------------------------------

------------------------------------------------------------

------------------------------------------------------------

------------------------------------------------------------

------------------------------------------------------------

------------------------------------------------------------

---------------------------------------------------------TTC

AGGAAGCCCAATCAATTCCTTGTACATCATACTACTGTAGTAAGTTCTGTGGGTTAGGTG

GTTGCTCATTATATGGATGTTACAAACTTCATCCCGGCAAAATTTGCTACTGCCTTCATT

GTCGCAGAGCTGAGTCTCCATTGGCACTTTCTGGAAGCGCTAGGAATGTGAACGAGCAGG

ACAAAGAGATGGACAACTCTCCAGT-----------------------------------

------------------------------------------------------------

------------------------------------------------------------

------------------------------------------------------------

------------------------------------------------------------

------------------------------------------------------------

------------------------------------------------------------

------------------------------------------------------------

---------------------------------GATGAATGAGGTGGACAATTTGGACCA

AGAAATGGATATGTTCTAG

>14_06cDNA

ATGAAGGCAACGATCGTGTTAGCTGTTGTAGTGGCAGTCATTGTTGGGG-----------

------------------------------------------------------------

------------------------------------------------------------

------------------------------------------------------------

------------------------------------------------------------

------------------------------------------------------------

------------------------------------------------------------

------------------------------------------------------------

------------------------------------------------------------

------------------------------------------------------------

------------------------------------------------------------

---------------------------------------------------------TTC

AGGAAGCCCAATCAATTCCTTGTACATCATACTACTGTAGTAAGTTCTGTGGGTCTGCTG

GTTGCTCATTATATGGATGTTACAAACTTCATCCCGGCAAAATTTGCTACTGCCTTCATT

GTCGCAGAGCTGAGTCTCCATTGGCACTTTCTGGAAGCGCTAGGAATGTGAACGAGCAGA

ACAAAGAGATGGACAACTCTCCAGT-----------------------------------

------------------------------------------------------------

------------------------------------------------------------

------------------------------------------------------------

------------------------------------------------------------

------------------------------------------------------------

------------------------------------------------------------

------------------------------------------------------------

---------------------------------GATGAATGAGATAGAAAATTTGGACCA

AGAAATGAATATGTTCTAG

>14_07cDNA

ATGAAGGCAACGATCGTGTTAGCTGTTGTAGTGGCAGTCATTGTTGGGG-----------

------------------------------------------------------------

------------------------------------------------------------

------------------------------------------------------------

------------------------------------------------------------

------------------------------------------------------------

------------------------------------------------------------

------------------------------------------------------------

------------------------------------------------------------

------------------------------------------------------------

------------------------------------------------------------

---------------------------------------------------------TTC

AGGAAGCCCAATCAATTCCTTGTACATCATACTACTGTAGTAAGTTCTGTGGGTCTGCTG

GTTGCTCATTATATGGATGTTACAAACTTCATCCCGGCAAAATTTGCTACTGCCTTCATT

GTCGCAGAGCTGAGTCTCCATTGGCACTTTCTGGAAGCGCTAGGAATGTGAACGAGCAGA

ACAAAGAGATGGACAACTCTCCAGT-----------------------------------

------------------------------------------------------------

------------------------------------------------------------

------------------------------------------------------------

------------------------------------------------------------

------------------------------------------------------------

------------------------------------------------------------

------------------------------------------------------------

---------------------------------GATGAATGAGATAGAAAATTTGGACCA

AGAAATGAATATGTTCTAG

>14_08cDNA

ATGAAGGCAACGATCGTGTTAGCTGTTGTAGTGGCAGTCATTGTTGGGG-----------

------------------------------------------------------------

------------------------------------------------------------

------------------------------------------------------------

------------------------------------------------------------

------------------------------------------------------------

------------------------------------------------------------

------------------------------------------------------------

------------------------------------------------------------

------------------------------------------------------------

------------------------------------------------------------

---------------------------------------------------------TTC

AGGAAGCCCAATCAATTCCTTGTACATCATACTACTGTAGTAAGTTCTGTGGGTCTGCTG

GTTGCTCATTATATGGATGTTACAAACTTCATCCCGGCAAAATTTGCTACTGCCTTCATT

GTCGCAGAGCTGAGTCTCCATTGGCACTTTCTGGAAGCGCTAGGAATGTGAACGAGCAGA

ACAAAGAGATGGACAACTCTCCAGT-----------------------------------

------------------------------------------------------------

------------------------------------------------------------

------------------------------------------------------------

------------------------------------------------------------

------------------------------------------------------------

------------------------------------------------------------

------------------------------------------------------------

---------------------------------GATGAATGAGATAGAAAATTTGGACCA

AGAAATGAATATGTTCTAG

>14_09cDNA

ATGAAGGCAACGATCTTGTTAGCTGTTGTAGTGGTAGTCATTGTTGGAG-----------

------------------------------------------------------------

------------------------------------------------------------

------------------------------------------------------------

------------------------------------------------------------

------------------------------------------------------------

------------------------------------------------------------

------------------------------------------------------------

------------------------------------------------------------

------------------------------------------------------------

------------------------------------------------------------

---------------------------------------------------------TTC

AGGAAGCCCAATCAATTCCTTGTACATCATACTACTGTAGTAAGTTCTGTGGGTCTGCTG

GTTGCTCATTATATGGATGTTACAAACTTCATCCCGGCAAAATTTGCTACTGCCTTCATT

GTCGCAGAGCTGAGTCTCCATTGGCACTTTCTGGAAGCGCTAGGAATGTGAACGAGCAGA

ACAAAGAGATGGTCAACTCTCCAGT-----------------------------------

------------------------------------------------------------

------------------------------------------------------------

------------------------------------------------------------

------------------------------------------------------------

------------------------------------------------------------

------------------------------------------------------------

------------------------------------------------------------

---------------------------------GATGAATGAGATGGAAAATTTGGACCA

AGAAATGGATATGTTCTAG

>14_10cDNA

ATGAAGGCAACGATCTTGTTAGCTGTTGTAGTGGTAGTCATTGTTGGAG-----------

------------------------------------------------------------

------------------------------------------------------------

------------------------------------------------------------

------------------------------------------------------------

------------------------------------------------------------

------------------------------------------------------------

------------------------------------------------------------

------------------------------------------------------------

------------------------------------------------------------

------------------------------------------------------------

---------------------------------------------------------TTC

AGGAAGCCCAATCAATTCCTTGTACATCATACTACTGTAGTAAGTTCTGTGGGTTAGGTG

GTTGCTCATTATATGGATGTTACAAACTTCATCCCGGCAAAATTTGCTACTGCCTTCATT

GTCGCAGAGCTGAGTCTCCATTGGCACTTTCTGGAAGCGCTAGGAATGTGAACGAGCAGA

ACAAAGAGATGGACAACTCTCCAGT-----------------------------------

------------------------------------------------------------

------------------------------------------------------------

------------------------------------------------------------

------------------------------------------------------------

------------------------------------------------------------

------------------------------------------------------------

------------------------------------------------------------

---------------------------------GATGAATGAGGTGGACAATTTGGACCA

AGAAATGGATATGTTCTAG

>16_01Gen

ATGAAGGCAACGATCTTGTTAGCTGTTGTAGTGGTAGTCATTGTTGGAGGTAAATATCTT

TATTGAAATATATCTGTGTGTACGTATTTTAGAAGTATAGATCCAACATTTTTTAAGATA

AAAATACGGTAGACCGACTTTCCCAAAATTTGATTGCGAATCCCATATT---------TG

TATATTATGTTAAATTATACTACTTTATTGTAATAATTTTCCATTTATTATAATAACTGT

TTTAAGTAAGAAAC----------------------------------------------

------------------------------------------------------------

-------------------------------------------TTGTTAATGTTTACAAT

TGAAATATGTTTTGTTATTAAAATTAAATCATTCAAATATTCAAACAAATATTTTTT-CT

TTATACGTGTTGTTCAGTTCGTAGTTTTCTATGCTATGCTTTGTATACTGTTGTTTGGTT

TTTTT---CTCCTTTTTTT----ACCATGGCGTTGTCAGTTTATTTTCAACTTAAGATAA

TAAATGTCCC-TGTGGTATATTT--AGCCTCTCTCTAAGACAGGCATCGTT-ATTTGTAT

CGAAA-CTTAAGCAATTTTATTC-A-TTTTATTTTT-CTAC------TTATTCA--GTTC

AGGAAGCCCAATCAATTCCTTGTACATCATACTACTGTAGTAAGTTCTGTGGGTCTGCTG

GTTGCTCATTATATGGATGTTACAAACTTCATCCCGGCAAAATTTGCTACTGCCTTCATT

GTCGCAGAGCTGAGTCTCCATTGGCACTTTCTGGAAGCGCTAGGAATGTGAACGAGCAGA

ACAAAGAGATGGTCAACTCTCCAGTGTAAGTACTCAATACTAGATTTCGTTACACAATAT

AAA-ATTTAAAAATTTGGAAAAAAAA------GTTTTCTACATGCCAACATATATTGTAA

TTAAGGTGTTTACCAATTTTTGAAACAGATAATTTGAT-TTTTTTTTAAATAAAATATTT

CATCTT-----AATTCAATATGTCGTTATCGTCTGAATCGTATTA------TGCAGTCAT

TGAAAAAAA---TAAACACTTGAACTACTCCTGCAAAAGAAGGAAGGGAT-GT-TTT-TT

TCTAAGATCGGTTAATGTGGTGGGAGGCAGATAAGATATCTTTTAGTGTGTCTCTCAACT

-ATTTACATTCAAAAACATTTTTTTTTT-ACATAAATCGATCACTGGTCAATTGCACGAG

AGAAGTCAGAAAAAAAC---TGTTATATAAAACGTTTTAATTGAATTTGTGAAAATAA-C

ACCACATATAAGAGAACATCTACTTTGTTTCAGGATGAATGAGATGGAAAATTTGGACCA

AGAAATGGAAATGTTCTAG

>16_02Gen

ATGAAGGCAACGATCTTGTTAGCTGTTGTAGTGGTAGTCATTGTTGGAGGTAAATATCTT

TATTGAAATATATCTGTGTGTACGTATTTTAGAAGTATAGATCCAACATTTTTTAAGATA

AAAATACGGTAGACCGACTTTCCCAAAATTTGATTGCGAATCCCATATT---------TG

TATATTATGTTAAATTATACTACTTTATTGTAATAATTTTCCATTTATTATAATAACTGT

TTTAAGTAAGAAAC----------------------------------------------

------------------------------------------------------------

-------------------------------------------TTGTTAATGTTTACAAT

TGAAATATGTTTTGTTATTAAAATTAAATCATTCAAATATTCAAACAAATATTTTTT-CT

TTATACGTGTTGTTCAGTTCGTAGTTTTCTATGCTATGCTTTGTATACTGTTGTTTGGTT

TTTTT---CTCCTTTTTTT----ACCATGGCGTTGTCAGTTTATTTTCAACTTAAGATAA

TAAATGTCCC-TGTGGTATATTT--AGCCTCTCTCTAAGACAGGCATCGTT-ATTTGTAT

CGAAA-CTTAAGCAATTTTATTC-A-TTTTATTTTT-CTAC------TTATTCA--GTTC

AGGAAGCCCAATCAATTCCTTGTACATCATACTACTGTAGTAAGTTCTGTGGGTCTGCTG

GTTGCTCATTATATGGATGTTACAAACTTCATCCCGGCAAAATTTGCTACTGCCTTCATT

GTCGCAGAGCTGAGTCTCCATTGGCACTTTCTGGAAGCGCTAGGAATGTGAACGAGCAGA

ACAAAGAGATGGTCAACTCTCCAGTGTAAGTACTCAATACTAGATTTCGTTACACAATAT

AAA-ATTTAAAAATTTGGAAAAAAAA------GTTTTCTACATGCCAACATATATTGTAA

TTAAGGTGTTTACCAATTTTTGAAACAGATAATTTGAT-TTTTTTTTAAATAAAATATTT

CATCTT-----AATTCAATATGTCGTTATCGTCTGAATCGTATTA------TGCAGTCAT

TGAAAAAAA---TAAACACTTGAACTACTCCTGCAAAAGAAGGAAGGGAT-GT-TTT-TT

TCTAAGATCGGTTAATGTGGTGGGAGGCAGATAAGATATCTTTTAGTGTGTCTCTCAACT

-ATTTACATTCAAAAACATTTTTTTTTT-ACATAAATCGATCACTGGTCAATTGCACGAG

AGAAGTCAGAAAAAAAC---TGTTATATAAAACGTTTTAATTGAATTTGTGAAAATAA-C

ACCACATATAAGAGAACATCTACTTTGTTTCAGGATGAATGAGATGGAAAATTTGGACCA

AGAAATGGAAATGTTCTAG

>16_04Gen

ATGAAGGCAACGATCTTGTTAGCTGTTGTAGTGGTAGTCATTGTTGGAGGTAAATATCTT

TATTGAAATATATCTGTGTGTACGTATTTTAGAAGTATAGATCCAACATTTTTTAAGATA

AAAATACGGTAGACCGACTTTCCCAAAATTTGATTGCGAATCCCATATT---------TG

TATATTATGTTAAATTATACTACTTTATTGTAATAATTTTCCATTTATTATAATAACTGT

TTTAAGTAAGAAAC----------------------------------------------

------------------------------------------------------------

-------------------------------------------TTGTTAATGTTTACAAT

TGAAATATGTTTTGTTATTAAAATTAAATCATTCAAATATTCAAACAAATATTTTTT-CT

TTATACGTGTTGTTCAGTTCGTAGTTTTCTATGCTATGCTTTGTATACTGTTGTTTGGTT

TTTTT---CTCCTTTTTTT----ACCATGGCGTTGTCAGTTTATTTTCAACTTAAGATAA

TAAATGTCCC-TGTGGTATATTT--AGCCTCTCTCTAAGACAGGCATCGTT-ATTTGTAT

CGAAA-CTTAAGCAATTTTATTC-A-TTTTATTTTT-CTAC------TTATTCA--GTTC

AGGAAGCCCAATCAATTCCTTGTACATCATACTACTGTAGTAAGTTCTGTGGGTCTGCTG

GTTGCTCATTATATGGATGTTACAAACTTCATCCCGGCAAAATTTGCTACTGCCTTCATT

GTCGCAGAGCTGAGTCTCCATTGGCACTTTCTGGAAGCGCTAGGAATGTGAACGAGCAGA

ACAAAGAGATGGTCAACTCTCCAGTGTAAGTACTCAATACTAGATTTCGTTACACAATAT

AAA-ATTTAAAAATTTGGAAAAAAAA------GTTTTCTACATGCCAACATATATTGTAA

TTAAGGTGTTTACCAATTTTTGAAACAGATAATTTGAT-TTTTTTTTAAATAAAATATTT

CATCTT-----AATTCAATATGTCGTTATCGTCTGAATCGTATTA------TGCAGTCAT

TGAAAAAAA---TAAACACTTGAACTACTCCTGCAAAAGAAGGAAGGGAT-GT-TTT-TT

TCTAAGATCGGTTAATGTGGTGGGAGGCAGATAAGATATCTTTTAGTGTGTCTCTCAACT

-ATTTACATTCAAAGACATTTTTTTTTT-ACATAAATCGATCACTGGTCAATTGCACGAG

AGAAGTCAGAAAAAAAC---TGTTATATAAAACGTTTTAATTGAATTTGTGAAAATAA-C

ACCACATATAAGAGAACATCTACTTTGTTTCAGGATGAATGAGATGGAAAATTTGGACCA

AGAAATGGAAATGTTCTAG

>16_06Gen

ATGAAGGCAACGATCTTGTTAGCTGTTGTAGTGGTAGTCATTGTTGGAGGTAACCATCTT

TATTGAAAGATATCCGTGTGTACGTATTTTAGAAGTATAGATCCAACATTTTTTAAGATA

AAAATACGGTAGACCGACTTTCCCAAAATTTGATTGCGAATCCCATATT---------TG

TATATTATGTTAAATTATACTACTTTATTGTAATAATTTTCCATTTATTATAATAACTGT

TTTAAGTAAGAAAC----------------------------------------------

------------------------------------------------------------

-------------------------------------------TTGTTAATGTTTACAAT

TGAAATATGTTTTGTTATTAAAATTAAATCATTCAAATATTCAAACAAATATTTTTT-CT

TTATACGTGTTGTTCAGTTCGTAGTTTTCTATGCTATGCTTTGTATACTGTTGTTTGGTT

TTTTT---CTCCTTTTTTT----ACCATGGCGTTGTCAGTTTATTTTCAACTTAAGATAA

TAAATGTCCC-TGTGGTATATTT--AGCCTCTCTCTAAGACAGGCATCGTT-ATTTGTAT

CGAAA-CTTAAGCAATTTTATTC-A-TTTTATTTTT-CTAC------TTATTCA--GTTC

AGGAAGCCCAATCAATTCCTTGTACATCATACTACTGTAGTAAGTTCTGTGGGTCTGCTG

GTTGCTCATTATATGGATGTTACAAACTTCATCCCGGCAAAATTTGCTACTGCCTTCATT

GTCGCAGAGCTGAGTCTCCATTGGCACTTTCTGGAAGCGCTAGGAATGTGAACGAGCAGA

ACAAAGAGATGGTCAACTCTCCAGTGTAAGTACTCAATACTAGATTTCGTTACACAATAT

AAA-ATTTAAAAATTTGGAAAAAAAA------GTTTTCTACATGCCAACATATATTGTAA

TTAAGGTGTTTACCAATTTTTGAAACAGATAATTTGAT-TTTTTTTTAAATAAAATATTT

CATCTT-----AATTCAATATGTCGTTATCGTCTGAATCGTATTA------TGCAGTCAT

TGAAAAAAA---TAAACACTTGAACTACTCCTGCAAAAGAAGGAAGGGAT-GT-TTT-TT

TCTAAGATCGGTTAATGTGGTGGGAGGCAGATAAGATATCTTTTAGTGTGTCTCTCAACT

-ATTTACATTCAAAAACATTTTTTTTTT-ACATAAATCGATCACTGGTCAATTGCACGAG

AGAAGTCAGAAAAAAAC---TGTTATATAAAACGTTTTAATTGAATTTGTGAAAATAA-C

ACCACATATAAGAGAACATCTACTTTGTTTCAGGATGAATGAGATGGAAAATTTGGACCA

AGAAATGGAAATGTTCTAG

>16_08Gen

ATGAAGGCAACGATCTTGTTAGCTGTTGTAGTGGTAGTCATTGTTGGAGGTAAATATCTT

TATTGAAATATATCTGTGTGTACGTATTTTAGAAGTATAGATCCAACATTTTTTAAGATA

AAAATACGGTAGACCGACTTTCCCAAAATTTGATTGCGAATCCCATATT---------TG

TATATTATGTTAAATTATACTACTTTATTGTAATAATTTTCCATTTATTATAATAACTGT

TTTAAGTAAGAAAC----------------------------------------------

------------------------------------------------------------

-------------------------------------------TTGTTAATGTTTACAAT

TGAAATATGTTTTGTTATTAAAATTAAATCATTCAAATATTCAAACAAATATTTTTT-CT

TTATACGTGTTGTTCAGTTCGTAGTTTTCTATGCTATGCTTTGTATACTGTTGTTTGGTT

TTTTT---CTCCTTTTTTT----ACCATGGCGTTGTCAGTTTATTTTCAACTTAAGATAA

TAAATGTCCC-TGTGGTATATTT--AGCCTCTCTCTAAGACAGGCATCGTT-ATTTGTAT

CGAAA-CTTAAGCAATTTTATTC-A-TTTTATTTTT-CTAC------TTATTCA--GTTC

AGGAAGCCCAATCAATTCCTTGTACATCATACTACTGTAGTAAGTTCTGTGGGTCTGCTG

GTTGCTCATTATATGGATGTTACAAACTTCATCCCGGCAAAATTTGCTACTGCCTTCATT

GTCGCAGAGCTGAGTCTCCATTGGCACTTTCTGGAAGCGCTAGGAATGTGAACGAGCAGA

ACAAAGAGATGGTCCACTCTCCAGTGTAAGTACTCAATACTAGATTTCATTACACAATAT

AAA-ATTTAAAAATTTGGAAAAAAAA------GTTTTCTACATGCCAACATATATTGTAA

TTAAGGTGTTTACCAATTTTTGAAACAGATAATTTGAT-TTTTTTTTAAATAAAATATTT

CATCTT-----AATTCAATATGTCGTTATCGTCTGAATCGTATTA------TGCAGTCAT

TGAAAAAAA---TAAACACTTGAACTACTCCTGCAAAAGAAGGAAGGGAT-GT-TTT-TT

TCTAAGATCGGTTAATGTGGTGGGAGGCAGATAAGATATCTTTTAGTGCGTCTCTCAACT

-ATTTACATTCAAAAACATTTTTTTTTTTACATAAATCGATCACTGGTCAATTGCACGAG

AGAAGTCAGAAAAAAAC---TGTTATATAAAACGTTTTAATTGAATTTGTGAAAATAA-C

ACCACATATAAGAGAACATCTACTTTGTTTCAGGATGAATGAGATGGAAAATTTGGACCA

AGAAATGGAAATGTTCTAG

>16_10Gen

ATGAAGGCAACGATCTTGTTAGCTGTTGTAGTGGTAGTCATTGTTGGAGGTAAATATCTT

TATTGAAATATATCTGTGTGTACGTATTTTAGAAGTATAGATCCAACATTTTTTAAGATA

AAAATACGGTAGACCGACTTTCCCAAAATTTGATTGCGAATCCCAAATT---------TG

TATATTATGTTAAATTATACTACTTTATTGTAATAATTTTCCATTTATTATAATAACTGT

TTTAAGTAAGAAAC----------------------------------------------

------------------------------------------------------------

-------------------------------------------TTGTTAATGTTTACAAT

TGAAATATGTTTTGTTATTAAAATTAAATCATTCAAATATTCAAACAAATATTTTTT-CT

TTATACGTGTTGTTCAGTTCGTAGTTTTCTATGCTATGCTTTGTATACTGTTGTTTGGTT

TTTTT---CTCCTTTTTTT----ACCATGGCGTTGTCAGTTTATTTTCAACTTAAGATAA

TAAATGTCCC-TGTGGTATATTT--AGCCTCTCTCTAAGACAGGCATCGTT-ATTTGTAT

CGAAA-CTTAAGCAATTTTATTC-A-TTTTATTTTT-CTAC------TTATTCA--GTTC

AGGAAGCCCAATCAATTCCTTGTACATCATACTACTGTAGTAAGTTCTGTGGGTCTGCTG

GTTGCTCATTATATGGATGTTACAAACTTCATCCCGGCAAAATTTGCTACTGCCTTCATT

GTCGCAGAGCTGAGTCTCCATTGGCACTTTCTGGAAGCGCTAGGAATGTGAACGAGCAGA

ACAAAGAGATGGTCAACTCTCCAGTGTAAGTACTCAATACTAGATTTCGTTACACAATAT

AAA-ATTTAAAAATTTGGAAAAAAAA------GTTTTCTACATGCCAACATATATTGTAA

TTAAGGTGTTTACCAATTTTTGAAACAGATAATTTGAT-TTTTTTTTAAATAAAATATTT

CATCTT-----AATTCAATATGTCGTTATCGTCTGAATCGTATTA------TGCAGTCAT

TGAAAAAAA---TAAACACTTGAACTACTCCTGCAAAAGAAGGAAGGGAT-GT-TTT-TT

TCTAAGATCGGTTAATGTGGTGGGAGGCAGATAAGATATCTTTTAGTGTGTCTCTCAACT

-ATTTACATTCAAAAACATTTTTTTTTT-ACATAAATCGATCACTGGTCAATTGCACGAG

AGAAGTCAGAAAAAAAC---TGTTATATAAAACGTTTTAATTGAATTTGTGAAAATAA-C

ACCACATATAAGAGAACATCTACTTTGTTTCAGGATGAATGAGATGGAAAATTTGGACCA

AGAAATGGAAATGTTCTAG

>16_11Gen

ATGAAGGCAACGATCTTGTTAGCTGTTGTAGTGGCAGTCATTGTTGGAGGTAAATATCTT

TATTGAAATATATCTGTGTGTACGTATTTTAGAAGTATGGATCCAGCATTTTT-AGGATA

AATATACGGCAGACCGACTTTTCTGAAA--------------------------------

-------------ATTTTACTACTTTATTGTAATAATTTTTCATTGA---TAATAAATGT

TTTAAGTAAGAAAG----------------------------------------------

------------------------------------------------------------

-------------------------------------------TTGTTAATGTTTACAAC

TGAAATATGTTTTGTTATTAAAATGAGATCATTCAAATATTCAAACAAATATTTTTT-CT

TTATACGTGTTGTTCAGTTCGTAGTTTTCTATGCTATGCTCTGTATACTGTTGTTTTTCT

TTTTTTT-CTCCTTTTTTTT---GCCATGGCGTTGTCAGTTTACTTTCAACTTAAGATAA

TAAATGTTCC-TGTGGTATATTT--AGCCTCTCTCTAAGACAGGCATCG---ATTTGTGT

CGAAA-CTTAAGCAATTTTATTC-A-TTTTATTTTT-CTAC------TTATTCA--GTTC

AGGAAGCCCAATCAGTAGCTTGTAGATCATACTACTGTAGTAAGTTCTGTGGGTCTGCTG

GTTGCTCATTATATGGATGTTACCTACTTCATCCTGGAAAAATTTGCTACTGCCTTCATT

GCAGCAGAGCTGAGTCTCCATTGGCACTTTCTGGAAGCGCTAGGAATGTGAACGACAAGA

ACAACGAGATGGACAACTCTCCAGTGTAAGTACTCAATACTAGATTTCGTTACACAATAT

AAA-ATTTGAAAA---AGAAAAAAAA------GTTTTCTACATGCCAACATATATTTTAA

TTAAGGTGTTTACCAATTTTTGAAACAGATAATTTG-T-TTTTTTTTAACTAAAATATTT

CATTTT-----AATTCCATATGTCGTTATCGTTTGAATCGTATTA------TGCAGTCAT

TGAAAAAAAA---AAATACCTGAACTACTCCTGCAAGAAAAAGAATGGAT-GT-TTT-TT

TCTAAGATCGGTTAATGTGTTGGGAAGCAGATAAGATATCTTCTAGTGTGTCTCTCAATT

TATTTACATTCAAAAACATTTTTTT----ACATAAATCGATCACTGGTCAATTGCACGAG

AGTAGTCAGAAAAAAAAC--TGTTATATAAAACGTTTTAATTGAATTTGTATAAAAAA-C

GCCACATATAAGAGACCATCTACTTTGTTTCAGGATGAATGAGGTGGAAAATTTGGACCA

AGAAATGGAAATGTTCTAG

>16_15Gen

ATGAAGGCAACGATCTTGTTAGCTGTTGTAGTGGTAGTCATTGTTGGAGGTAAATATCTT

TATTGAAATATATCTGTGTGTACGTATTTTAGAAGTATAGATCCAACATTTTTTAAGATA

AAAATACGGTAGACCGACTTTCCCAAAATTTGATTGCGAATCCCATATT---------TG

TATATTATGTTAAATTATACTACTTTATTGTAATAATTTTCCATTTATTATAATAACTGT

TTTAAGTAAGAAAC----------------------------------------------

------------------------------------------------------------

-------------------------------------------TTGTTAATGTTTACAAT

TGAAATATGTTTTGTTATTAAAATTAAATCATTCAAATATTCAAACAAATATTTTTT-CT

TTATACGTGTTGTTCAGTTCGTAGTTTTCTATGCTATGCTTTGTATACTGTTGTTTGGTT

TTTTT---CTCCTTTTTTT----ACCATGGCGTTGTCAGTTTATTTTCAACTTAAGATAA

TAAATGTCCC-TGTGGTATATTT--AGCCTCTCTCTAAGACAGGCATCGTT-ATTTGTAT

CGAAA-CTTAAGCAATTTTATTC-A-TTTTATTTTT-CTAC------TTATTCA--GTTC

AGGAAGCCCAATCAATTCCTTGTACATCATACTACTGTAGTAAGTTCTGTGGGTCTGCTG

GTTGCTCATTATATGGATGTTACAAACTTCATCCCGGCAAAATTTGCTACTGCCTTCATT

GTCGCAGAGCTGAGTCTCCATTGGCACTTTCTGGAAGCGCTAGGAATGTGAACGAGCAGA

ACAAAGAGATGGTCAACTCTCCAGTGTAAGTACTCAATACTAGATTTCGTTACACAATAT

AAA-ATTTAAAAATTTGGAAAAAAAA------GTTTTCTACATGCCAACATATATTGTAA

TTAAGGTGTTTACCAATTTTTGAAACAGATAATTTGAT-TTTTTTTTAAATAAAATATTT

CATCTT-----AATTCAATATGTCGTTATCGTCTGAATCGTATTA------TGCAGTCAT

TGAAAAAAA---TAAACACTTGAACTACTCCTGCAAAAGAAGGAAGGGAT-GT-TTT-TT

TCTAAGATCGGTTAATGTGGTGGGAGGCAGATAAGATATCTTTTAGTGTGTCTCTCAACT

-ATTTACATTCAAAAACATTTTTTTTTT-ACATAAATCGATCACTGGTCAATTGCACGAG

AGAAGTCAGAAAAAAAC---TGTTATATAAAACGTTTTAATTGAATTTGTGAAAATAA-C

ACCACATATAAGAGAACATCTACTTTGTTTCAGGATGAATGAGATGGAAAATTTGGACCA

AGAAATGGAAATGTTCTAG

>16_30Gen

ATGAAGGCAACGATCTTGTTAGCTGTTGTAGTGGTAGTCATTGTTGGAGGTAAATATCTT

TATTGAAATATATCTGTGTGTACGTATTTTAGAAGTATAGATCCAACATTTTTTAAGATA

AAAATACGGTAGACCGACTTTCCCAAAATTTGATTGCGAATCCCATATT---------TG

TATATTATGTTAAATTATACTACTTTATTGTAATAATTTTCCATTTATTATAATAACTGT

TTTAAGTAAGAAAC----------------------------------------------

------------------------------------------------------------

-------------------------------------------TTGTTAATGTTTACAAT

TGAAATATGTTTTGTTATTAAAATTAAATCATTCAAATATTCAAACAAATATTTTTT-CT

TTATACGTGTTGTTCAGTTCGTAGTTTTCTATGCTATGCTTTGTATACTGTTGTTTGGTT

TTTTT---CTCCTTTTTTT----ACCATGGCGTTGTCAGTTTATTTTCAACTTAAGATAA

TAAATGTCCC-TGTGGTATATTT--AGCCTCTCTCTAAGACAGGCATCGTT-ATTTGTAT

CGAAA-CTTAAGCAATTTTATTC-A-TTTTATTTTT-CTAC------TTATTCA--GTTC

AGGAAGCCCAATCAATTCCTTGTACATCATACTACTGTAGTAAGTTCTGTGGGTCTGCTG

GTTGCTCATTATATGGATGTTACAAACTTCATCCCGGCAAAATTTGCTACTGCCTTCATT

GTCGCAGAGCTGAGTCTCCATTGGCACTTTCTGGAAGCGCTAGGAATGTGAACGAGCAGA

ACAAAGAGATGGTCAACTCTCCAGTGTAAGTACTCAATACTAGATTTCGTTACACAATAT

AAA-ATTTAAAAATTTGGAAAAAAAA------GTTTTCTACATGCCAACATATATTGTAA

TTGAGGTGTTTACCAATTTTTGAAACAGATAATTTGAT-TTTTTTTTAAATAAAATATTT

CATCTT-----AATTCAATATGTCGTTATCGTCTGAATCGTATTA------TGCAGTCAT

TGAAAAAAA---TAAACACTTGAACTACTCCTGCAAAAGAAGGAAGGGAT-GT-TTT-TT

TCTAAGATCGGTTAATGTGGTGGGAGGCAGATAAGATATCTTTTAGTGTGTCTCTCAACT

-ATTTACATTCAAAAACATTTTTTTTTT-ACATAAATCGATCACTGGTCAATTGCACGAG

AGAAGTCAGAAAAAAAC---TGTTATATAAAACGTTTTAATTGAATTTGTGAAAATAA-C

ACCACATATAAGAGAACATCTACTTTGTTTCAGGATGAATGAGATGGAAAATTTGGACCA

AGAAATGGAAATGTTCTAG

>16_01cDNA

ATGAAGGCAACGATCTTGTTAGCTGTTGTAGTGGCAGTCATTGTTGGAG-----------

------------------------------------------------------------

------------------------------------------------------------

------------------------------------------------------------

------------------------------------------------------------

------------------------------------------------------------

------------------------------------------------------------

------------------------------------------------------------

------------------------------------------------------------

------------------------------------------------------------

------------------------------------------------------------

---------------------------------------------------------TTC

AGGAAGCCCAATCAGTAGCTTGTAGATCATACTACTGTAGTAAGTTCTGTGGGTCTGCTG

GTTGCTCATTATATGGATGTTACCTACTTCATCCTGGAAAAATTTGCTACTGCCTTCATT

GTAGCAGAGCTGAGTCTCCATTGGCACTTTCTGGAAGCGCTAGGAATGTGAACGACAAGA

ACAACGAGATGGACAACTCTCCAGT-----------------------------------

------------------------------------------------------------

------------------------------------------------------------

------------------------------------------------------------

------------------------------------------------------------

------------------------------------------------------------

------------------------------------------------------------

------------------------------------------------------------

---------------------------------GATGAATGAGGTGGAAAATTTGGACCA

AGAAATGAATATGTTCTAG

>16_02cDNA

ATGAAGGCAACGATCTTGTTAGCTGTTGTAGTGGTAGTCATTGTTGGAG-----------

------------------------------------------------------------

------------------------------------------------------------

------------------------------------------------------------

------------------------------------------------------------

------------------------------------------------------------

------------------------------------------------------------

------------------------------------------------------------

------------------------------------------------------------

------------------------------------------------------------

------------------------------------------------------------

---------------------------------------------------------TTC

AGGAAGCCCAATCAATTCCTTGTACATCATACTACTGTAGTAAGTTCTGTGGGTCTGCTG

GTTGCTCATTATATGGATGTTACAAACTTCATCCCGGCAAAATTTGCTACTGCCTTCATT

GTCGCAGAGCTGAGTCTCCATTGGCACTTTCTGGAAGCGCTAGGAATGTGAACGAGCAGA

ACAAAGAGATGGTCAACTCTCCAGT-----------------------------------

------------------------------------------------------------

------------------------------------------------------------

------------------------------------------------------------

------------------------------------------------------------

------------------------------------------------------------

------------------------------------------------------------

------------------------------------------------------------

---------------------------------GATGAATGAGATGGAAAATTTGGACCA

AGAAATGGAAATGTTCTAG

>16_03cDNA

ATGAAGGCAACGATCTTGTTAGCTGTTGTAGTGGTAGTCATTGTTGGAG-----------

------------------------------------------------------------

------------------------------------------------------------

------------------------------------------------------------

------------------------------------------------------------

------------------------------------------------------------

------------------------------------------------------------

------------------------------------------------------------

------------------------------------------------------------

------------------------------------------------------------

------------------------------------------------------------

---------------------------------------------------------TTC

AGGAAGCCCAATCAATTCCTTGTACATCATACTACTGTAGTAAGTTCTGTGGGTCTGCTG

GTTGCTCATTATATGGATGTTACAAACTTCATCCCGGCAAAATTTGCTACTGCCTTCATT

GTCGCAGAGCTGAGTCTCCATTGGCACTTTCTGGAAGCGCTAGGAATGTGAACGAGCAGA

ACAAAGAGATGGTCAACTCTCCAGT-----------------------------------

------------------------------------------------------------

------------------------------------------------------------

------------------------------------------------------------

------------------------------------------------------------

------------------------------------------------------------

------------------------------------------------------------

------------------------------------------------------------

---------------------------------GATGAATGAGATGGAAAATTTGGGCCA

AGAAATGGAAATGTTCTAG

>16_04cDNA

ATGAAGGCAACGATCTTGTTAGCTGTTGTAGTGGCAGTCATTGTTGGAG-----------

------------------------------------------------------------

------------------------------------------------------------

------------------------------------------------------------

------------------------------------------------------------

------------------------------------------------------------

------------------------------------------------------------

------------------------------------------------------------

------------------------------------------------------------

------------------------------------------------------------

------------------------------------------------------------

---------------------------------------------------------TTC

AGGAAGCCCAATCAGTAGCTTGTAGATCATACTACTGTAGTAAGTTCTGTGGGTCTGCTG

GTCGCTCATTATATGGATGTTACCTACTTCATCCTGGAAAAATTTGCTACTGCCTTCATT

GTAGCAGAGCTGAGTCTCCATTGGCACTTTCTGGAAGCGCTAGGAATGTGAACGACAAGA

ACAACGAGATGGACAACTCTCCAGT-----------------------------------

------------------------------------------------------------

------------------------------------------------------------

------------------------------------------------------------

------------------------------------------------------------

------------------------------------------------------------

------------------------------------------------------------

------------------------------------------------------------

---------------------------------GATGAATGAGGTGGAAAATTTGGACCA

AGAAATGaAtATGTTCTAG

>16_05cDNA

ATGAAGGCAACGATCTTGTTAGCTGTTGTAGTGGTAGTCATTGTTGGAG-----------

------------------------------------------------------------

------------------------------------------------------------

------------------------------------------------------------

------------------------------------------------------------

------------------------------------------------------------

------------------------------------------------------------

------------------------------------------------------------

------------------------------------------------------------

------------------------------------------------------------

------------------------------------------------------------

---------------------------------------------------------TTC

AGGAAGCCCAATCAATTCCTTGTACATCATACTACTGTAGTAAGTTCTGTGGGTCTGCTG

GTTGCTCATTATATGGATGTTACAAACTTCATCCCGGCAAAATTTGCTACTGCCTTCATT

GTCGCAGAGCTGAGTCTCCATTGGCACTTTCTGGAAGCGCTAGGAATGTGAACGAGCAGA

ACAAAGAGATGGTCAACTCTCCAGT-----------------------------------

------------------------------------------------------------

------------------------------------------------------------

------------------------------------------------------------

------------------------------------------------------------

------------------------------------------------------------

------------------------------------------------------------

------------------------------------------------------------

---------------------------------GATGAATGAGATGGAAAATTTGGACCA

AGAAATGGAAATGTTCTAG

>16_06cDNA

ATGAAGGCAACGATCTTGTTAGCTGTTGTAGTGGCAGTCATTGTTGGAG-----------

------------------------------------------------------------

------------------------------------------------------------

------------------------------------------------------------

------------------------------------------------------------

------------------------------------------------------------

------------------------------------------------------------

------------------------------------------------------------

------------------------------------------------------------

------------------------------------------------------------

------------------------------------------------------------

---------------------------------------------------------TTC

AGGAAGCCCAATCAGTAGCTTGTAGATCATACTACTGTAGTAAGTTCTGTGGGTCTGCTG

GTTGCTCATTATATGGATGTTACCTACTTCATCCTGGAAAAATTTGCTACTGCCTTCATT

GTAGCAGAGCTGAGTCTCCATTGGCACTTTCTGGAAGCGCTAGGAATGTGAACGACAAGA

ACAACGAGATGGACAACTCTCCAGT-----------------------------------

------------------------------------------------------------

------------------------------------------------------------

------------------------------------------------------------

------------------------------------------------------------

------------------------------------------------------------

------------------------------------------------------------

------------------------------------------------------------

---------------------------------GATGAATGAGGTGGAAAATTTGGACCA

AGAAATGAATATGTTCTAG

>16_07cDNA

ATGAAGGCAACGATCTTGTTAGCTGTTGTAGTGGTAGTCATTGTTGGAG-----------

------------------------------------------------------------

------------------------------------------------------------

------------------------------------------------------------

------------------------------------------------------------

------------------------------------------------------------

------------------------------------------------------------

------------------------------------------------------------

------------------------------------------------------------

------------------------------------------------------------

------------------------------------------------------------

---------------------------------------------------------TTC

AGGAAGCCCAATCAATTCCTTGTACATCATACTACTGTAGTAAGTTCTGTGGGTCTGCTG

GTTGCTCATTATATGGATGTTACAAACTTCATCCCGGCAAAATTTGCTACTGCCTTCATT

GTCGCAGAGCTGAGTCTCCATTGGCACTTTCTGGAAGCGCTAGGAATGTGAACGAGCAGA

ACAAAGAGATGGTCAACTCTCCAGT-----------------------------------

------------------------------------------------------------

------------------------------------------------------------

------------------------------------------------------------

------------------------------------------------------------

------------------------------------------------------------

------------------------------------------------------------

------------------------------------------------------------

---------------------------------GATGAATGAGATGGAAAATTTGGACCA

AGAAATGGAAATGTTCTAG

>16_08cDNA

ATGAAGGCAACGATCTTGTTAGCTGTTGTAGTGGTAGTCATTGTTGGAG-----------

------------------------------------------------------------

------------------------------------------------------------

------------------------------------------------------------

------------------------------------------------------------

------------------------------------------------------------

------------------------------------------------------------

------------------------------------------------------------

------------------------------------------------------------

------------------------------------------------------------

------------------------------------------------------------

---------------------------------------------------------TTC

AGGAAGCCCAATCAATTCCTTGTACATCATACTACTGTAGTAAGTTCTGTGGGTCTGCTG

GTTGCTCATTATATGGATGTTACAAACTTCATCCCGGCAAAATTTGCTACTGCCTTCATT

GTCGCAGAGCTGAGTCTCCATTGGCACTTTCTGGAAGCGCTAGGAATGTGAACGAGCAGA

ACAAAGAGATGGTCAACTCTCCAGT-----------------------------------

------------------------------------------------------------

------------------------------------------------------------

------------------------------------------------------------

------------------------------------------------------------

------------------------------------------------------------

------------------------------------------------------------

------------------------------------------------------------

---------------------------------GATGAATGAGATGGAAAATTTGGACCA

AGAAATGGAAATGTTCTAG

>16_09cDNA

ATGAAGGCAACGATCTTGTTAGCTGTTGTAGTGGTAGTCATTGTTGGAG-----------

------------------------------------------------------------

------------------------------------------------------------

------------------------------------------------------------

------------------------------------------------------------

------------------------------------------------------------

------------------------------------------------------------

------------------------------------------------------------

------------------------------------------------------------

------------------------------------------------------------

------------------------------------------------------------

---------------------------------------------------------TTC

AGGAAGCCCAATCAATTCCTTGTACATCATACTACTGTAGTAAGTTCTGTGGGTCTGCTG

GTTGCTCATTATATGGATGTTACAAACTTCATCCCGGCAAAATTTGCTACTGCCTTCATT

GTCGCAGAGCTGAGTCTCCATTGGCACTTTCTGGAAGCGCTAGGAATGTGAACGAGCAGA

ACAAAGAGATGGTCAACTCTCCAGT-----------------------------------

------------------------------------------------------------

------------------------------------------------------------

------------------------------------------------------------

------------------------------------------------------------

------------------------------------------------------------

------------------------------------------------------------

------------------------------------------------------------

---------------------------------GATGAATGAGATGGAAAATTTGGACCA

AGAAATGGAAATGTTCTAG

>17_01Gen

ATGAAGGCAACGATCTTGTTAGCTGTTGTAGTGGCAGTCATTGTTGGAGGTAAATATCTT

TATTGAAATATATCTGTGTGTACGTATTTTAGAAGTATAGATCCAGCATTTTT-AAGATA

AAAATACGGCAGACCGACTTTTCCAAAATTTGATTACGAATCCCATATT---------TG

TATATTATGTTAAATTATACCACTTTATTGTAATAATTTTTCATTTA---TAATAACTGT

TTTAAGTAAGAAAGCTGTATTTGGCAAAACTTTTAGGAATTTTGGTCCTGAATGCTCTTC

TTTTT--AACTTTTATGATTCGAGCGTCACTGATGAGTCTTTTGTTGACGAAACGCGCGT

CTGGCGTATATTTAATTTAGTTCTGGTATCTATGATGAGTTTATTGTTAATATTTACAAC

TGAAATATGCTTTGTTATTAAAATTAAATCACTCAAATACTCAAACAAATATTTTTT-CT

TTATACGTGTTGTTCAGTTCGTAGTTTTCTATGCTATGCTTTGTATACTGTTGTTTGTCT

TTTTTTT-CTCCTTTTTTTTTT-GCCATGGCGTTGTCAGTTCAGTTTCAACGTATGATAA

TAAATGTCCC-TGTGGTATATTT--AGCCTCTCTCTAAGACAGTCATCGTT-ATTTGTGT

CGAAA-CTTGAGCAATTTTATTC-A-TTTTATTTTT-CTAC------TTATTCA--GTTC

AGGAAGCCCAATCAGTAGCTTGTACATCATACTACTGTAGTAAGTTCTGTGGGTCTGCTG

GTTGCTCATTATATGGATGTTACCTACTTCATCCTGGCAAAATTTGCTACTGCCTTCATT

GTCGCAGAGCTGAGTCTCCATTGGCACTTTCTGGAAGCGCTAGGAATGTGAACGACAAGA

ACAACGAGATGGACAACTCTCCAGTGTAAGTACTCAATACTAGATTTCGTTACACAATAT

AAACATTTAAAAATTT-GAAAAAACA------GTTTTCTTCATGCCAACATATATTGTAA

TTAAGGTGTTTACCAATTTTTGAAACAGATAATTTGAT-TTTTTTTTAAATAAAATATTT

CATCTT-----AATTCAATATGTCGTTATCGTTTGAATCGTATTA------TGCAGTCAT

TGAAAAAAAAA-TAAACACCTTAACTGCTCCTTCAAAAGAAGGAAGGGAT-GT-TTT-TT

TCTTAGATCGGTTAATGTGTTGGGAGGCAGATAAGATATCTTCTAGTGTGTCTCTCAATT

-ATTTACATTCAAAAACATTTTT------ACATAAATCGATCACTGGTCAATTGCACGAG

GGTAGTCAGAAAAAAAACC-TGTTATATAAAACGTTTTAATTGAATTTGTGAAAATAA-C

GCCACATATAAGAAAACATCTACTTTGTTTCAGGATGAATGAGATGGAAAATTTGGACCA

AGAAATGGATATGTTCTAG

>17_04Gen

ATGAAGGCAACGATCTTGTTAGCTGTTGTAGTGGCAGTCATTGTTGGAGGTAAATATCTT

TATTGAAATATATCTGTGTGTACGTATTTTAGAAGTATAGATCCAGCATTTTT-AAGATA

AAAATACGGCAGACCGACCTTTCCAAAATTTGATTGCGAATCCCATATT---------TG

TATATTATGTTAAATTATACTACTTTATTGTAATAATTTTTCATTTA---TAATAACTGT

TTTAAGTAAGAAAGCTGTATTTGGCAAAACTTTTAGGAATTTTGGTCCTGAATGCTCTTC

TTTTT--AACTTTTATGATTCGAGCGTCACTGATGAGTCTTTTGTTGACGAAACGCGCGT

CTGGCGTATATTTAATTTAGTTCTGGTATCTATGATGAGTTTATTGTTAATATTTACAAC

TGAAATATGCTTTGTTATTAAAATTAAATCACTCAAATACTCAAACAAATATTTTTT-CT

TTATACGTGTTGTTCAGTTCGTAGTTTTCTATGCTATGCTTTGTATACTGTTGTTTGTCT

TTTTTTT-CTCCTTTTTTTTTTTGCCATGGCGTTGTCAGTTCATTTTCAACGTATGATAA

TAAATGTCCC-TGTGGTATATTT--AGCCTCTCTCTAAGACAGGCATCGTT-ATTTGTGT

CGAAAACTTGAGCAATTTTATTC-A-TTTTATTTTT-CTAC------TTATTCA--GTTC

AGGAAGCCCAATCAGTAGCTTGTACATCATACTACTGTAGTAAGTTCTGTGGGTCTGCTG

GTTGCTCATTATATGGATGTTACCTACTTCATCCTGGCAAAATTTGCTACTGCCTTCATT

GTCGCAGAGCTGAGTCTCCATTGGCACTTTCTGGAAGCGCTAGGAATGTGAACGACAAGA

ACAACGAGATGGACAACTCTCCAGTGTAAGTACTCAATACTAGATTTCGTTACACAATAT

AAACATTTAAAAATTTGGAAAAAACA------GTTTTCTTCATGCCAACATATATTGTAA

TTAAGGTGTTTACCAATTTTTGAAACAGATAATTTGAT-TTTTTTTTAAATAAAATATTT

CATCTT-----AATTCAATATGTCGTTATCGTCTGAATCGTATTA------TGCAGTCAT

TGAAAAAAAAA-TAAACACCTTAACTGCTCCTTCAAAAGAAGGAAGGGAT-GT-TTT-TT

TCTTAGATCGGTTAATGTGTTGGGAGGCAGATAAGATATCTTCTAGTGTGTCTCTCAATT

-ATTTACATTCAAAAACATTTTT------ACATAAATCGATCACTGGTCAATTGCACGAG

AGTAGTCAGAAAAAAAACC-TGTTATATAAAACGTTTTAATTGAATTTGTGAAAATAA-C

GCCACATATAAGAAAACATCTACTTTGTTTCAGGATGAATGAGATGGAAAATTTGGACCA

GGAAATGGATATGTTCTAG

>17_05Gen

ATGAAGGCAACGATCTTGTTAGCTGTTGTAGTGGCAGTCATTGTTGGAGGTAAATATCTT

TATTGAAATATATCTGTGTGTACGTATTTTAGAAGTATAGATCCAGCATTTTT-AAGATA

AAAATACGGCAGACCGACTTTTCCAAAATTTGATTGCGAATCCCATATT---------TG

TATATTATGTTAAATTATACTACTTTATTGTAATAATTTTTCATTTA---TAATAACTGT

TTTAAGTAAGAAAGCTGTATTTGGCAAAACTTTTAGGAATTTTGGTCCTGAATGCTCTTC

TTTTT--AACTTTTATGATTCGAGCGTCACTGATGAGTCTTTTGTTGACGAAACGCGCGT

CTGGCGTATATTTAATTTAGTTCTGGTATCTATGATGAGTTTATTGTTAATATTTACAAC

TGAAATATGCTTTGTTATTAAAATTAAATCACTCAAATACTCAAACAAATATTTTTT-CT

TTATACGTGTTGTTCAGTTCGTAGTTTTCTATGCTATGCTTTGTATACTGTTGTTTGTCT

TTTTTTT-CTCCTTTTTTTTTTTGCCATGGCGTTGTCAGTTCATTTTCAACGTATGATAA

TAAATGTCCC-TGTGGTATATTT--AGCCTCTCTCTAAGACAGGCATCGTT-ATTTGTGT

CGAAA-CTTGAGCAATTTTATTC-A-TTTTATTTTT-CTAC------TTATTCA--GTTC

AGGAAGCCCAATCAGTAGCTTGTACATCATACTACTGTAGTAAGTTCTGTGGGTCTGCTG

GTTGCTCATTATATGGATGTTACCTACTTCATCCTGGCAAAATTTGCTACTGCCTTCATT

GTCGCAGAGCTGAGTCTCCATTGGCACTTTCTGGAAGCGCTAGGAATGTGAACGACAAGA

ACAACGAGATGGACAACTCTCCAGTGTAAGTACTCAATACTAGATTTCGTTACACAATAT

AAACATTTAAAAATTTGGAAAAAACA------GTTTTCTTCATGCCAACATATATTGTAA

TTAAGGTGTTTACCAATTTTTGAAACAGATAATTTGAA-TTTTTTTTAAATAAAATATTT

CATCTT-----AATTCAATATGTCGTTATCGTTTGAATCGTATTA------TGCAGTCAT

TGAAAAAAAAA-TAAACACCTTAACTGCTCCTTCAAAAGAAGGAAGGGAT-GT-TTT-TT

TCTTAGATCGGTTAATGTGTTGGGAGGCAGATAAGATATCTTCTAGTGTGTCTCTCAATT

-ATTTACATTCAAAAACATTTTT------ACATAAATCGATCACTGGTCAATTGCACGAG

AGTAGTCAGAAAAAAAACC-TGTTATATAAAACGTTTTAATTGAATTTGTGAAAATAA-C

GCCACATATAAGAAAACATCTACTTTGTTTCAGGATGAATGAGATGGAAAATTTGGACCA

AGAAATGGATATGTTCTAG

>17_06Gen

ATGAAGGCAACGATCTTGTTAGCTGTTGTAGTGGCAGTCATTGTTGGAGGTAAATATCTT

TATTGAAATATATCTGTGTGTACGTATTTTAGAAGTATAGATCCAGCATTTTT-AAGATA

AAAATACGGCAGACCGACTTTTCCAAAATTTGATTGCGAATCCCATATT---------TG

TATATTATGTTAAATTATACTACTTTATTGTAATAATTTTTCATTTA---TAATAACTGT

TTTAAGTAAGAAAGCTGTATTTGGCAAAACTTTTAGGAATTTTGGTCCTGAATGCTCTTC

TTTTT--AACTTTTATGATTCGAGCGTCACTGATGAGTCTTTTGTTGACGAAACGCGCGT

CTGGCGTATATTTAATTTAGTTCTGGTATCTATGATGAGTTTATTGTTAATATTTACAAC

TGAAATATGCTTTGTTATTAAAATTAAATCACTCAAATACTCAAACAAATATTTTTT-CT

TTATACGTGTTGTTCAGTTCGTAGTTTTCTATGCTATGCTTTGTATACTGTTGTTTGTCT

TTTTTTT-CTCCTTTTTTTTTTTGCCATGGCGTTGTCAGTTCAGTTTCAACGTATGATAA

TAAATGTCCC-TGTGGTATATTT--AGCCTCTCTCTAAGACAGTCATCGTT-ATTTGTGT

CGAAA-CTTGAGCAATTTTATTC-A-TTTTATTTTT-CTAC------TTATTCA--GTTC

AGGAAGCCCAATCAGTAGCTTGTACATCATACTACTGTAGTAAGTTCTGTGGGTCTGCTG

GTTGCTCATTATATGGATGTTACCTACTTCATCCTGGCAAAATTTGCTACTGCCTTCATT

GTCGCAGAGCTGAGTCTCCATTGGCACTTTCTGGAAGCGCTAGGAATGTGAACGACAAGA

ACAACGAGATGGACAACTCTCCAGTGTAAGTACTCAATACTAGATTTCGTTACACAATAT

AAACATTTAAAAATTT-GAAAAAACA------GTTTTCTTCATGCCGACATATATTGTAA

TTAAGGTGTTTACCAATTTTTGAAACAGATAATTTGAT-TTTTTTTTAAATAAAATATTT

CATCTT-----AATTCAATATGTCGTTATCGTTTGAATCGTATTA------TGCAGTCAT

TGAAAAAAAAA-TAAACACCTTAACTGCTCCTTCAAAAGAAGGAAGGGAT-GT-TTT-TT

TCTTAGATCGGTTAATGTGTTGGGAGGCAGATAAGATATCTTCTAGTGTGTCTCTCAATT

-ATTTACATTCAAAAACATTTTT------ACATAAATCGATCACTGGTCAATTGCACGAG

AGTAGTCAGAAAAAAAACC-TGTTATATAAAACGTTTTAATTGAATTTGTGAAAATAA-C

GCCACATATAAGAAAACATCTACTTTGTTTCAGGATGAATGAGATGGAAAATTTGGACCA

AGAAATGGATATGTTCTAG

>17_07Gen

ATGAAGGCAACGATCTTGTTAGCTGTTGTAGTGGCAGTCATTGTTGGAGGTAAATATCTT

TATTGAAATATATCTGTGTGTACGTATTTTAGAAGTATAGATCCAGCATTTTT-AAGATA

AAAATACGGCAGACCGACTTTTCCAAAATTTGATTGCGAATCCCATATT---------TG

TATATTATGTTAAATTATACTACTTTATTGTAATAATTTTTCATTTA---TAATAACTGT

TTTAAGTAAGAAAGCTGTATTTGGCAAAACTTTTAGGAATTTTGGTCCTGAATGCTCTTC

TTTTT--AACTTTTATGATTCGAGCGTCACTGATGAGTCTTTTGTTGACGAAACGCGCGT

CTGGCGTATATTTAATTTAGTTCTGGTATCTATGATGAGTTTATTGTTAATATTTACAAC

TGAAATATGCTTTGTTATTAAAATTAAATCACTCAAATACTCAAACAAATATTTTTT-CT

TTATACGTGTTGTTCAGTTCGTAGTTTTCTATGCTATGCTTTGTATACTGTTGTTTGTCT

TTTTTTT-CTCCTTTTTTTTTTTGCCATGGCGTTGTCAGTTCATTTTCAACGTATGATAA

TAAATGTCCC-TGTGGTATATTT--AGCCTCTCTCTAAGACAGGCATCGTT-ATTTGTGT

CGAAA-CTTGAGCAATTTTATTC-A-TTTTATTTTT-CTAC------TTATTCA--GTTC

AGGAAGCCCAATCAGTAGCTTGTACATCATACTACTGTAGTAAGTTCTGTGGGTCTGCTG

GTTGCTCATTATATGGATGTTACCTACTTCATCCTGGCAAAATTTGCTACTGCCTTCATT

GTCGCAGAGCTGAGTCTCCATTGGCACTTTCTGGAAGCGCTAGGAATGTGAACGACAAGA

ACAACGAGATGGACAACTCTCCAGTGTAAGTACTCAATACTAGATTTCGTTACACAATAT

AAACATTTAAAAATTTGGAAAAAACA------GTTTTCTTCATGCCAACATATATTGTAA

TTAAGGTGTTTACCAATTTTTGAAACAGATAATTTGAT-TTTTTTTTAAATAAAATATTT

CATCTT-----AATTCAATATGTCGTTATCGTTTGAATCGTATTA------TGCAGTCAT

TGAAAAAAAAA-TAAACACCTTAACTGCTCCTTCAAAAGAAGGAAGGGAT-GT-TTT-TT

TCTTAGATCGGTTAATGTGTTGGGAGGCAGATAAGATATCTTCTAGTGTGTCTCTCAATT

-ATTTACATTCAAAAACATTTTT------ACATAAATCGATCACTGGTCAATTGCACGAG

AGTAGTCAGAAAAAAA-CC-TGTTATATAAAACGTTTTAATTGAATTTGTGAAAATAA-C

GCCACATATAAGAAAACATCTACTTTGTTTCAGGATGAATGAGATGGAAAATTTGGACCA

AGAAATGGATATGTTCTAG

>17_08Gen

ATGAAGGCAACGATCTTGTTAGCTGTTGTAGTGGCAGTCATTGTTGGAGGTAAATATCTT

TATTGAAATATATCTGTGTGTACGTATTTTAGAAGTATAGATCCAGCATTTTT-AAGATA

AAAATACGGCAGACCGACTTTTCCAAAATTTGATTGCGAATCCCATATT---------TG

TATATTATGTTAAATTATACTACTTTATTGTAATAATTTTTCATTTA---TAATAACTGT

TTTAAGTAAGAAAGCTGTATTTGGCAAAACTTTTAGGAATTTTGGTCCTGAATGCTCTTC

TTTTT--AACTTTTATGATTCGAGCGTCACTGATGAGTCTTTTGTTGACGAAACGCGCGT

CTGGCGTATATTTAATTTAGTTCTGGTATCTATGATGAGTTTATTGTTAATATTTACAAC

TGAAATATGCTTTGTTATTAAAATTAAATCACTCAAATACTCAAACAAATATTTTTT-CT

TTATACGTGTTGTTCAGTTCGTAGTTTTCTATGCTATGCTTTGTATACTGTTGTTTGTCT

TTTTTTT-CTCCTTTTTTTTTTTGCCATGGCGTTGTCAGTTCAGTTTCAACGTATGATAA

TAAATGTCCC-TGTGGTATATTT--AGCCTCTCTCTAAGACAGTCATCGTT-ATTTGTGT

CGAAA-CTTGAGCAATTTTATTC-A-TTTTATTTTT-CTAC------TTATTCA--GTTC

AGGAAGCCCAATCAGTAGCTTGTACATCATACTACTGTAGTAAGTTCTGTGGGTCTGCTG

GTTGCTCATTATATGGATGTTACCTACTTCATCCTGGCAAAATTTGCTACTGCCTTCATT

GTCGCAGAGCTGAGTCTCCATTGGCACTTTCTGGAAGCGCTAGGAATGTGAACGACAAGA

ACAACGAGATGGACAACTCTCCAGTGTAAGTACTCAATACTAGATTTCGTTACACAATAT

AAACATTTAAAAATTT-GAAAAAACA------GTTTTCTTCATGCCAACATATATTGTAA

TTAAGGTGTTTACCAATTTTTGAAACAGATAATTTGAT-TTTTTTTTAAATAAAATATTT

CATCTT-----AATTCAATATGTCGTTATCGTTTGAATCGTATTA------TGCAGTCAT

TGAAAAAAAAA-TAAACACCTTAACTGCTCCTTCAAAAGAAGGAAGGGAT-GT-TTT-TT

TCTTAGATCGGTTAATGTGTTGGGAGGCAGATAAGATATCTTCTAGTGTGTCTCTCAATT

-ATTTACATTCAAAAACATTTTT------ACATAAATCGATCACTGGTCAATTGCACGAG

AGTAGTCAGAAAAAAAACC-TGTTATATAAAACGTTTTAATTGAATTTGTGAAAATAA-C

GCCACATATAAGAAAACATCTACTTTGTTTCAGGATGAATGAGATGGAAAATTTGGACCA

AGAAATGGATATGTTCTAG

>17_09Gen

ATGAAGGCAACGATCTTGTTAGCTGTTGTAGTGGCAGTCATTGTTGGAGGTAAATATCTT

TATTGAAATATATCTGTGTGTACGTATTTTAGAAGTATAGATCCAGCATTTTT-AAGATA

AAAATACGGCAGACCGACTTTTCCAAAATTTGATTGCGAATCCCATATT---------TG

TATATTATGTTAAATTATACTACTTTATTGTAATAATTTTTCATTTA---TAATAACTGT

TTTAAGTAAGAAAGCTGTATTTGGCAAAACTTTTAGGAATTTTGGTCCTGAATGCTCTTC

TTTTT--AACTTTTATGATTCGAGCGTCACTGATGAGTCTTTTGTTGACGAAACGCGCGT

CTGGCGTATATTTAATTTAGTTCTGGTATCTATGATGAGTTTATTGTTAATATTTACAAC

TGAAATATGCTTTGTTATTAAAATTAAATCACTCAAATACTCAAACAAATATTTTTT-CT

TTATACGTGTTGTTCAGTTCGTAGTTTTCTATGCTATGCTTTGTATACTGTTGTTTGTCT

TTTTTTT-CTCCTTTTTTTTTTTGCCATGGCGTTGTCAGTTCAGTTTCAACGTATGATAA

TAAATGTCCC-TGTGGTATATTT--AGCCTCTCTCTAAGACAGTCATCGTT-ATTTGTGT

CGAAA-CTTGAGCAATTTTATTC-A-TTTTATTTTT-CTAC------TTATTCA--GTTC

AGGAAGCCCAATCAGTAGCTTGTACATCATACTACTGTAGTAAGTTCTGTGGGTCTGCTG

GTTGCTCATTATATGGATGTTACCTACTTCATCCTGGCAAAATTTGCTACTGCCTTCATT

GTCGCAGAGCTGAGTCTCCATTGGCACTTTCTGGAAGCGCTAGGAATGTGAACGACAAGA

ACAACGAGATGGACAACTCTCCAGTGTAAGTACTCAATACTAGATTTCGTTACACAATAT

AAACATTTAAAAATTT-GAAAAAACA------GTTTTCTTCATGCCAACATATATTGTAA

TTAAGGTGTTTACCAATTTTTGAAACAGATAATTTGAT-TTTTTTTTAAATAAAATATTT

CATCTT-----AATTCAATATGTCGTTATCGTTTGAATCGTATTA------TGCAGTCAT

TGAAAAAAAAA-TAAACACCTTAACTGCTCCTTCAAAAGAAGGAAGGGAT-GT-TTT-TT

TCTTAGATCGGTTAATGTGTTGGGAGGCAGATAAGATATCTTCTAGTGTGTCTCTCAATT

-ATTTACATTCAAAAACATTTTT------ACATAAATCGATCACTGGTCAATTGCACGAG

AGTAGTCAGAAAAAAAACC-TGTTATATAAAACGTTTTAATTGAATTTGTGAAAATAA-C

GCCACATATAAGAAAACATCTACTTTGTTTCAGGATGAATGAGATGGAAAATTTGGACCA

AGAAATGGATATGTTCTAG

>17_10Gen

ATGAAGGCAACGATCTTGTTAGCTGTTGTAGTGGCAGTCATTGTTGGAGGTAAATATCTT

TATTGAAATATATCTGTGTGTACGTATTTTAGAAGTATAGATCCAGCATTTTT-AAGATA

AAAATACGGCAGACCGACTTTTCCAAAATTTGATTGCGAATCCCATATT---------TG

TATATTATGTTAAATTATACTACTTTATTGTAATAATTTTTCATTTA---TAATAACTGT

TTTAAGTAAGAAAGCTGTATTTGGCAAAACTTTTAGGAATTTTGGTCCTGAATGCTCTTC

TTTTT--AACTTTTATGATTCGAGCGTCACTGATGAGTCTTTTGTTGACGAAACGCGCGT

CTGGCGTATATTTAATTTAGTTCTGGTATCTATGATGAGTTTATTGTTAATATTTACAAC

TGAAATATGCTTTGTTATTAAAATTAAATCACTCAAATACTCAAACAAATATTTTTT-CT

TTATACGTGTTGTTCAGTTCGTAGTTTTCTATGCTATGCTTTGTATACTGTTGTTTGTCT

TTTTTTT-CTCCTTTTTTTTTTTGCCATGGCGTTGTCAGTTCAGTTTCAACGTATGATAA

TAAATGTCCC-TGTGGTATATTT--AGCCTCTCTCTAAGACAGTCATCGTT-ATTTGTGT

CGAAA-CTTGAGCAATTTTATTC-A-TTTTATTTTT-CTAC------TTATTCA--GTTC

AGGAAGCCCAATCAGTAGCTTGTACATCATACTACTGTAGTAAGTTCTGTGGGTCTGCTG

GTTGCTCATTATATGGATGTTACCTACTTCATCCTGGCAAAATTTGCTACTGCCTTCATT

GTCGCAGAGCTGAGTCTCCATTGGCACTTTCTGGAAGCGCTAGGAATGTGAACGACAAGA

ACAACGAGATGGACAACTCTCCAGTGTAAGTACTCAATACTAGATTTCGTTACACAATAT

AAACATTTAAAAATTT-GAAAAAACA------GTTTTCTTCATGCCAACATATATTGTAA

TTAAGGTGTTTACCAATTTTTGAAACAGATAATTTGAT-TTTTTTTTAAATAAAATATTT

CATCTT-----AATTCAATATGTCGTTATCGTTTGAATCGTATTA------TGCAGTCAT

TGAAAAAAAAA-TAAACACCTTAACTGCTCCTTCAAAAGAAGGAAGGGAT-GT-TTT-TT

TCTTAGATCGGTTAATGTGTTGGGAGGCAGATAAGATATCTTCTAGTGTGTCTCTCAATT

-ATTTACATTCAAAAACATTTTT------ACATAAATCGATCACTGGTCAATTGCACGAG

AGTAGTCAGAAAAAAAACC-TGTTATATAAAACGTTTTAATTGAATTTGTGAAAATAA-C

GCCACATATAAGAAAACATCTACTTTGTTTCAGGATGAATGAGATGGAAAATTTGGACCA

AGAAATGGATATGTTCTAG

>17_12Gen

ATGAAGGCAACGATCTTGTTAGCTGTTGTAGTGGCAGTCATTGTTGGAGGTAAATATCTT

TATTGAAATATATCTGTGTGTACGTATTTTAGAAGTATAGATCCAGCATTTTT-AAGATA

AAAATACGGCAGACCGACTTTTCCAAAATTTGATTGCGAATCCCATATT---------TG

TATATTATGTTAAATTATACTACTTTATTGTAATAATTTTTCATTTA---TAATAACTGT

TTTAAGTAAGAAAGCTGTATTTGGCAAAACTTTTAGGAATTTTGGTCCTGAATGCTCTTC

TTTTT--AACTTTTATGATTCGAGCGTCACTGATGAGTCTTTTGTTGACGAAACGCGCGT

CTGGCGTATATTTAATTTAGTTCTGGTATCTATGATGTGTTTATTGTTAATATTTACAAC

TGAAATATGCTTTGTTATTAAAATTAAATCACTCAAATACTCAAACAAATATTTTTT-CT

TTATACGTGTTGTTCAGTTCGTAGTTTTCTATGCTATGCTTTGTATACTGTTGTTTGTCT

TTTTTTT-CTCCTTTTTTTTTTTGCCATGGCGTTGTCAGTTCAGTTTCAACGTATGATAA

TAAATGTCCC-TGTGGTATATTT--AGCCTCTCTCTAAGACAGTCATCGTT-ATTTGTGT

CGAAA-CTTGAGCAATTTTATTC-A-TTTTATTTTT-CTAC------TTATTCA--GTTC

AGGAAGCCCAATCAGTAGCTTGTACATCATACTACTGTAGTAAGTTCTGTGGGTCTGCTG

GTTGCTCATTATATGGATGTTACCTACTTCATCCTGGCAAAATTTGCTACTGCCTTCATT

GTCGCAGAGCTGAGTCTCCATTGGCACTTTCTGGAAGCGCTAGGAATGTGAACGACAAGA

ACAACGAGATGGACAACTCTCCAGTGTAAGTACTCAATACTAGATTTCGTTACACAATAT

AAACATTTAAAAATTT-GAAAAAACA------GTTTTCTTCATGCCAACATATATTGTAA

TTAAGGTGTTTACCAATTTTTGAAACAGATAATTTGAT-TTTTTTT-AAATAAAATATTT

CATCTT-----AATTCAATATGTCGTTATCGTCTGAATCGTATTA------TGCAGTCAT

TGAAAAAAAA--TAAACACCTTAACTGCTCCTTCAAAAGAAGGAAGGGAT-GT-TTT-TT

TCTTAGATCAGTTAATGTGTTGGGAGGCAGATAAGATATCTTCTAGTGTGTCTCTCAATT

-ATTTACATTCAAAAACATTTTT------ACATAAATCGATCACTGGTCAATTGCACGAG

AGTAGTCAGAAAAAAAACC-TGTTATATAAAACGTTTTAATTGAATTTGTGAAAATAA-C

GCCACATATAAGAAAACATCTACTTTGTTTCAGGATGAATGAGATGGAAAATTTGGACCA

AGAAATGGATATGTTCTAG

>17_13Gen

ATGAAGGCAACGATCTTGTTAGCTGTTGTAGTGGCAGTCATTGTTGGAGGTAAATATCTT

TATTGAAATATATCTGTGTGTACGTATTTTAGAAGTATAGATCCAGCATTTTT-AAGATA

AAAATACGGCAGACCGACTTTTCCAAAATTTGATTGCGAATCCCATATT---------TG

TATATTATGTTAAATTATACTACTTTATTGTAATAATTTTTCATTTA---TAATAACTGT

TTTAAGTAAGAAAGCTGTATTTGGCAAAACTTTTAGGAATTTTGGTCCTGAATGCTCTTC

TTTTT--AACTTTTATGATTCGAGCGTCACTGATGAGTCTTTTGTTGACGAAACGCGCGT

CTGGCGTATATTTAATTTAGTTCTGGTATCTATGATGAGTTTATTGTTAATATTTACAAC

TGAAATATGCTTTGTTATTAAAATTAAATCACTCAAATACTCAAACAAATATTTTTT-CT

TTATACGTGTTGTTCAGTTCGTAGTTTTCTATGCTATGCTTTGTATACTGTTGTTTGTCT

TTTTTTT-CTCCTTTTTTTTTTTGCCATGGCGTTGTCAGTTCAGTTTCAACGTATGATAA

TAAATGTCCC-TGTGGTATATTT--AGCCTCTCTCTAAGACAGTCATCGTT-ATTTGTGT

CGAAA-CTTGAGCAATTTTATTC-A-TTTTATTTTT-CTAC------TTATTCA--GTTC

AGGAAGCCCAATCAGTAGCTTGTACATCATACTACTGTAGTAAGTTCTGTGGGTCTGCTG

GTTGCTCATTATATGGATGTTACCTACTTCATCCTGGCAAAATTTGCTACTGCCTTCATT

GTCGCAGAGCTGAGTCTCCATTGGCACTTTCTGGAAGCGCTAGGAATGTGAACGACAAGA

ACAACGAGATGGACAACTCTCCAGTGTAAGTACTCAATACTAGATTTCGTTACACAATAT

AAACATTTAAAAATTT-GAAAAAACA------GTTTTCTTCATGCCAACATATATTGTAA

TTAAGGTGTTTACCAATTTTTGAAACAGATAATTTGAT-TTTTTTTTAAATAAAATATTT

CATCTT-----AATTCAATATGTCGTTATCGTTTGAATCGTATTA------TGCAGTCAT

TGAAAAAAAAA-TAAACACCTTAACTGCTCCTTCAAAAGAAGGAAGGGAT-GT-TTT-TT

TCTTAGATCGGTTAATGTGTTGGGAGGCAGATAAGATATCTTCTAGTGTGTCTCTCAATT

-ATTTACATTCAAAAACATTTTT------ACATAAATCGATCACTGGTCAATTGCACGAG

AGTAGTCAGAAAAAAAACC-TGTTATATAAAACGTTTTAATTGAATTTGTGAAAATAA-C

GCCACATATAAGAAAACATCTACTTTGTTTCAGGATGAATGAGATGGAAAATTTGGACCA

AGAAATGGATATGTTCTAG

>17_15Gen

ATGAAGGCAACGATCTTGTTAGCTGTTGTAGTGGCAGTCATTGTTGGAGGTAAATATCTT

TATTGAAATATATCTGTGTGTACGTATTTTAGAAGTATAGATCCAGCATTTTT-AAGATA

AAAATACGGCAGACCGACTTTTCCAAAATTTGATTGCGAATCCCATATT---------TG

TATATTATGTTAAATTATACTACTTTATTGTAATAATTTTTCATTTA---TAATAACTGT

TTTAAGTAAGAAAGCTGTATTTGGCAAAACTTTTAGGAATTTTGGTCCTGAATGCTCTTC

TTTTT--AACTTTTATGATTCGAGCGTCACTGATGAGTCTTTTGTTGACGAAACGCGCGT

CTGGCGTATATTTAATTTAGTTCTGGTATCTATGATGAGTTTATTGTTAATATTTACAAC

TGAAATATGCTTTGTTATTAAAATTAAATCACTCAAATACTCAAACAAATATTTTTT-CT

TTATACGTGTTGTTCAGTTCGTAGTTTTCTATGCTATGCTTTGTATACTGTTGTTTGTCT

TTTTTTT-CTCCTTTTTTTTTTTGCCATGGCGTTGTCAGTTCAGTTTCAACGTATGATAA

TAAATGTCCC-TGTGGTATATTT--AGCCTCTCTCTAAGACAGTCATCGTT-ATTTGTGT

CGAAA-CTTGAGCAATTTTATTC-A-TTTTATTTTT-CTAC------TTATTCA--GTTC

AGGAAGCCCAATCAGTAGCTTGTACATCATACTACTGTAGTAAGTTCTGTGGGTCTGCTG

GTTGCTCATTATATGGATGTTACCTACTTCATCCTGGCAAAATTTGCTACTGCCTTCATT

GTCGCAGAGCTGAGTCTCCATTGGCACTTTCTGGAAGCGCTAGGAATGTGAACGACAAGA

ACAACGAGATGGACAACTCTCCAGTGTAAGTACTCAATACTAGATTTCGTTACACAATAT

AAACATTTAAAAATTT-GAAAAAACA------GTTTTCTTCATGCCAACATATATTGTAA

TTAAGGTGTTTACCAATTTTTGAAACAGATAATTTGAT-TTTTTTTTAAATAAAATATTT

CATCTT-----AATTCAATATGTCGTTATCGTCTGAATCGTATTA------TGCAGTCAT

TGAAAAAAAAA-TAAACACCTTAACTGCTCCTTCAAAAGAAGGAAGGGAT-GT-TTT-TT

TCTTAGATCGGTTAATGTGTTGGGAGGCAGATAAGATATCTTCTAGTGTGTCTCTCAATT

-ATTTACATTCAAAAACATTTTT------ACATAAATCGATCACTGGTCAATTGCACGAG

AGTAGTCAGAAAAAAAACC-TGCTATATAAAACGTTTTAATTGAATTTGTGAAAATAA-C

GCCACATATAAGAAAACATCTACTTTGTTTCAGGATGAATGAGATGGAAAATTTGGACCA

AGAAATGGATATGTTCTAG

>17_16Gen

ATGAAGGCAACGATCTTGTTAGCTGTTGTAGTGGCAGTCATTGTTGGAGGTAAATATCTT

TATTGAAATATATCTGTGTGTACGTATTTTAGAAGTATAGATCCAGCATTTTT-AAGATA

AAAATACGGCAGACCGACTTTTCCAAAATTTGATTGCGAATCCCATATT---------TG

TATATTATGTTAAATTATACTACTTTATTGTAATAATTTTTCATTTA---TAATAACTGT

TTTAAGTAAGAAAGCTGTATTTGGCAAAACTTTTAGGAATTTTGGTCCTGAATGCTCTTC

TTTTT--AACTTTTATGATTCGAGCGTCACTGATGAGTCTTTTGTTGACGAAACGCGCGT

CTGGCGTATATTTAATTTAGTTCTGGTATCTATGATGAGTTTATTGTTAATATTTACAAC

TGAAATATGCTTTGTTATTAAAATTAAATCACTCAAATACTCAAACAAATATTTTTT-CT

TTATACGTGTTGTTCAGTTCGTAGTTTTCTATGCTATGCTTTGTATACTGTTGTTTGTCT

TTTTTTT-CTCCTTTTTTTTTT-GCCATGGCGTTGTCAGTTCATTTTCAGCGTATGATAA

TAAATGTCCC-TGTGGTATATTT--AGCCTCTCTCTAAGACAGTCATCGTT-ATTTGTGT

CGAAA-CTTGAGCAATTTTATTC-A-TTTTATTTTTTCTAC------TTATTCA--GTTC

AGGAAGCCCAATCAGTAGCTTGTACATCATACTACTGTAGTAAGTTCTGTGGGTCTGCTG

GTTGCTCATTATATGGATGTTACCTACTTCATCCTGGCAAAATTTGCTACTGCCTTCATT

GTCGCAGAGCTGAGTCTCCATTGGCACTTTCTGGAAGCGCTAGGAATGTGAACGACAAGA

ACAACGAGATGGACAACTCTCCAGTGTAAGTACTCAATACTAGATTTCGTTACACAATAT

AAACATTTAAAAATTT-GAAAAAACA------GTTTTCTTCATGCCAACATATATTGTAA

TTAAGGTGTTTACCAATTTTTGAAACAGATAATTTGAT-TTTTTTTTAAATAAAATATTT

CATCTT-----AATTCAATATGTCGTTATCGTTTGAATCGTATTA------TGCAGTCAT

TGAAAAAAAAA-TAAACACCTTAACTGCTCCTTCAAAAGAAGGAAGGGAT-GT-TTT-TT

TCTTAGATCGGTTAATGTGTTGGGAGGCAGATAAGATATCTTCTAGTGTGTCTCTCAATT

-ATTTACATTCAAAAACATTTTT------ACATAAATCGATCACTGGTCAATTGCACGAG

AGTAGTCAGAAAAAAAACC-TGTTATATAAAACGTTTTAATTGAATTTGTGAAAATAA-C

GCCACATATAAGAAAACATCTACTTTGTTTCAGGATGAATGAGATGGAAAATTTGGACCA

AGAAATGGATATGTTCTAG

>17_01cDNA

ATGAAGGCAACGATCTTGTTAGCTGTTGTAGTGGCAGTCATTGTTGGAG-----------

------------------------------------------------------------

------------------------------------------------------------

------------------------------------------------------------

------------------------------------------------------------

------------------------------------------------------------

------------------------------------------------------------

------------------------------------------------------------

------------------------------------------------------------

------------------------------------------------------------

------------------------------------------------------------

---------------------------------------------------------TTC

AGGAAGCCCAATCAGTAGCTTGTACATCATACTACTGTAGTAAGTTCTGTGGGTCTGCTG

GTTGCTCATTATATGGATGTTACCTACTTCATCCTGGCAAAATTTGCTACTGCCTTCATT

GTCGCAGAGCTGAGTCTCCATTGGCACTTTCTGGAAGCGCTAGGAATGTGAACGACAAGA

ACAACGAGATGGACAACTCTCCAGT-----------------------------------

------------------------------------------------------------

------------------------------------------------------------

------------------------------------------------------------

------------------------------------------------------------

------------------------------------------------------------

------------------------------------------------------------

------------------------------------------------------------

---------------------------------GATGAATGAGATGGAAAATTTGGACCA

AGAAATGGATATGTTCTAG

>17_02cDNA

ATGAAGGCAACGATCTTGTTAGCTGTTGTAGTGGCAGTCATTGTTGGAG-----------

------------------------------------------------------------

------------------------------------------------------------

------------------------------------------------------------

------------------------------------------------------------

------------------------------------------------------------

------------------------------------------------------------

------------------------------------------------------------

------------------------------------------------------------

------------------------------------------------------------

------------------------------------------------------------

---------------------------------------------------------TTC

AGGAAGCCCAATCAGTAGCTTGTACATCATACTACTGTAGTAAGTTCTGTGGGTCTGCTG

GTTGCTCATTATATGGATGTTACCTACTTCATCCTGGCAAAATTTGCTACTGCCTTCATT

GTCGCAGAGCTGAGTCTCCATTGGCACTTTCTGGAAGCGCTAGGAATGTGAACGACAAGA

ACAACGAGATGGACAACTCTCCAGT-----------------------------------

------------------------------------------------------------

------------------------------------------------------------

------------------------------------------------------------

------------------------------------------------------------

------------------------------------------------------------

------------------------------------------------------------

------------------------------------------------------------

---------------------------------GATGAATGAGATGGAAAATTTGGACCA

AGAAATGGATATGTTCTAG

>17_03cDNA

ATGAAGGCAACGATCTTGTTAGCTGTTGTAGTGGCAGTCATTGTTGGAG-----------

------------------------------------------------------------

------------------------------------------------------------

------------------------------------------------------------

------------------------------------------------------------

------------------------------------------------------------

------------------------------------------------------------

------------------------------------------------------------

------------------------------------------------------------

------------------------------------------------------------

------------------------------------------------------------

---------------------------------------------------------TTC

AGGAAGCCCAATCAGTAGCTTGTACATCATACTACTGTAGTAAGTTCTGTGGGTCTGCTG

GTTGCTCATTATATGGATGTTACCTACTTCATCCTGGCAAAATTTGCTACTGCCTTCATT

GTCGCAGAGCTGAGTCTCCATTGGCACTTTCTGGAAGCGCTAGGAATGTGAACGACAAGA

ACAACGAGATGGACAACTCTCCAGT-----------------------------------

------------------------------------------------------------

------------------------------------------------------------

------------------------------------------------------------

------------------------------------------------------------

------------------------------------------------------------

------------------------------------------------------------

------------------------------------------------------------

---------------------------------GATGAATGAGATGGAAAATTTGGACCA

AGAAATGGATATGTTCTAG

>17_04cDNA

ATGAAGGCGACGATCTTGTTAGCTGTTGTAGTGGCAGTCATTGTTGGAG-----------

------------------------------------------------------------

------------------------------------------------------------

------------------------------------------------------------

------------------------------------------------------------

------------------------------------------------------------

------------------------------------------------------------

------------------------------------------------------------

------------------------------------------------------------

------------------------------------------------------------

------------------------------------------------------------

---------------------------------------------------------TTC

AGGAAGCCCAATCAGTAGCTTGTACATCATACTACTGTAGTAAGTTCTGTGGGTCTGCTG

GTTGCTCATTATATGGATGTTACCTACTTCATCCAGGCAAAATTTGCTACTGCCTTCATT

GTCGCAGAGCTGAGTCTCCATTGGCACTTTCTGGAAGCGCTAGGAATGTGAACGACAAGA

ACAACGAGATGGACAACTCTCCAGT-----------------------------------

------------------------------------------------------------

------------------------------------------------------------

------------------------------------------------------------

------------------------------------------------------------

------------------------------------------------------------

------------------------------------------------------------

------------------------------------------------------------

---------------------------------GATGAATGAGATGGAAAATTTGGACCA

AGAAATGGATATGTTCTAG

>17_05cDNA

ATGAAGGCAACGATCTTGTTAGCTGTTGTAGTGGCAGTCATTGTTGGAG-----------

------------------------------------------------------------

------------------------------------------------------------

------------------------------------------------------------

------------------------------------------------------------

------------------------------------------------------------

------------------------------------------------------------

------------------------------------------------------------

------------------------------------------------------------

------------------------------------------------------------

------------------------------------------------------------

---------------------------------------------------------TTC

GGGAAGCCCAATCAGTAGCTTGTACATCATACTACTGTAGTAAGTTCTGTGGGTCTGCTG

GTTGCTCATTATATGGATGTTACAAACTTCATCCTGGAAAAATTTGCTACTGCCTTCATT

GTAGCAGAGCTGAGTCTCCATTGGCACTTTCTGGAAGCGCTAGGAATGTGAACGACAAGA

ACAACGAGATGGAGAACTCTCCATT-----------------------------------

------------------------------------------------------------

------------------------------------------------------------

------------------------------------------------------------

------------------------------------------------------------

------------------------------------------------------------

------------------------------------------------------------

------------------------------------------------------------

---------------------------------GATGAATGAGATGGACAATTTGGACCA

AGAAATGAATATGTTCTAG

>17_06cDNA

ATGAAGGCAACGATCTTGTTAGCTGTTGTAGTGGCAGTCATTGTTGGAG-----------

------------------------------------------------------------

------------------------------------------------------------

------------------------------------------------------------

------------------------------------------------------------

------------------------------------------------------------

------------------------------------------------------------

------------------------------------------------------------

------------------------------------------------------------

------------------------------------------------------------

------------------------------------------------------------

---------------------------------------------------------TTC

AGGAAGCCCAATCAGTAGCTTGTACATCATACTACTGTAGTAAGTTCTGTGGGTCTGCTG

GTTGCTCATTATATGGATGTTACCTACTTCATCCTGGCAAAATTTGCTACTGCCTTCATT

GTCGCAGAGCTGAGTCTCCATTGGCACTTTCTGGAAGCGCTAGGAATGTGAACGACAAGA

ACAACGAGATGGACAACTCTCCAGT-----------------------------------

------------------------------------------------------------

------------------------------------------------------------

------------------------------------------------------------

------------------------------------------------------------

------------------------------------------------------------

------------------------------------------------------------

------------------------------------------------------------

---------------------------------GATGAATGAGATGGAAAATTTGGACCA

AGAAATGGATATGTTCTAG

>17_07cDNA

ATGAAGGCAACGATCTTGTTAGCTGTTGTAGTGGCAGTCATTGTTGGAG-----------

------------------------------------------------------------

------------------------------------------------------------

------------------------------------------------------------

------------------------------------------------------------

------------------------------------------------------------

------------------------------------------------------------

------------------------------------------------------------

------------------------------------------------------------

------------------------------------------------------------

------------------------------------------------------------

---------------------------------------------------------TTC

AGGAAGCCCAATCAGTAGCTTGTACATCATACTACTGTAGTAAGTTCTGTGGGTCTGCTG

GTTGCTCATTATATGGATGTTACCTACTTCATCCTGGCAAAATTTGCTACTGCCTTCATT

GTCGCAGAGCTGAGTCTCCATTGGCACTTTCTGGAAGCGCTAGGAATGTGAACGACAAGA

ACAACGAGATGGACAACTCTCCAGT-----------------------------------

------------------------------------------------------------

------------------------------------------------------------

------------------------------------------------------------

------------------------------------------------------------

------------------------------------------------------------

------------------------------------------------------------

------------------------------------------------------------

---------------------------------GATGAATGAGATGGAAAATTTGGACCA

AGAAATGGATATGTTCTAG

>17_08cDNA

ATGAAGGCAACGATCTTGTTAGCTGTTGTAGTGGCAGTCATTGTTGGAG-----------

------------------------------------------------------------

------------------------------------------------------------

------------------------------------------------------------

------------------------------------------------------------

------------------------------------------------------------

------------------------------------------------------------

------------------------------------------------------------

------------------------------------------------------------

------------------------------------------------------------

------------------------------------------------------------

---------------------------------------------------------TTC

AGGAAGCCCAATCAGTAGCTTGTACATCATACTACTGTAGTAAGTTCTGTGGGTCTGCTG

GTTGCTCATTATATGGATGTTACCTACTTCATCCTGGCAAAATTTGCTACTGCCTTCATT

GTCGCAGAGCTGAGTCTCCATTGGCACTTTCTGGAAGCGCTAGGAATGTGAACGACAAGA

ACAACGAGATGGACAACTCTCCAGT-----------------------------------

------------------------------------------------------------

------------------------------------------------------------

------------------------------------------------------------

------------------------------------------------------------

------------------------------------------------------------

------------------------------------------------------------

------------------------------------------------------------

---------------------------------GATGAATGAGATGGAAAATTTGGACCA

AGAAATGGATATGTTCTAG

>17_09cDNA

ATGAAGGCAACGATCTTGTTAGCTGTTGTAGTGGCAGTCATTGTTGGAG-----------

------------------------------------------------------------

------------------------------------------------------------

------------------------------------------------------------

------------------------------------------------------------

------------------------------------------------------------

------------------------------------------------------------

------------------------------------------------------------

------------------------------------------------------------

------------------------------------------------------------

------------------------------------------------------------

---------------------------------------------------------TTC

AGGAAGCCCAATCAGTAGCTTGTACATCATACTACTGTAGTAAGTTCTGTGGGTCTGCTG

GTTGCTCATTATATGGATGTTACCTACTTCATCCTGGCAAAATTTGCTACTGCCTTCATT

GTCGCAGAGCTGAGTCTCCATTGGCACTTTCTGGAAGCGCTAGGAATGTGAACGACAAGA

ACAACGAGATGGACAACTCTCCAGT-----------------------------------

------------------------------------------------------------

------------------------------------------------------------

------------------------------------------------------------

------------------------------------------------------------

------------------------------------------------------------

------------------------------------------------------------

------------------------------------------------------------

---------------------------------GATGAATGAGATGGAAAATTTGGACCA

AGAAATGGATATGTTCTAG

>17_10cDNA

ATGAAGGCAACGATCTTGTTAGCTGTTGTAGTGGCAGTCATTGTTGGAG-----------

------------------------------------------------------------

------------------------------------------------------------

------------------------------------------------------------

------------------------------------------------------------

------------------------------------------------------------

------------------------------------------------------------

------------------------------------------------------------

------------------------------------------------------------

------------------------------------------------------------

------------------------------------------------------------

---------------------------------------------------------TTC

AGGAAGCCCAATCAGTAGCTTGTACATCATACTACTGTAGTAAGTTCTGTGGGTCTGCTG

GTTGCTCATTATATGGATGTTACAAACTTCATCCTGGAAAAATTTGCTACTGCCTTCATT

GTAGCAGAGCTGAGTCTCCATTGGCACTTTCTGGAAGCGCTAGGAATGTGAACGACAAGA

ACAACGAGATGGAGAACTCTCCATT-----------------------------------

------------------------------------------------------------

------------------------------------------------------------

------------------------------------------------------------

------------------------------------------------------------

------------------------------------------------------------

------------------------------------------------------------

------------------------------------------------------------

---------------------------------GATGAATGAGATGGACAATTTGGACCA

AGAAATGAATATGTTCTAG

>21_08Gen

ATGAAGGCAACGATCTTGTTAGCTGTTGTAGTGGCAGTCATTGTTGGAGGTAAATATCTT

TATTGAAATATATCTGTGTGTACGTATTTTAGAAGTATAGATCCAGCATTTTT-AGGATA

AATATACGGCAGACCGACTTTTCTGAAA--------------------------------

-------------ATTTTACTACTTTATTGTAATAATTTTTCATTGA---TAATAAATGT

TTTAAGTAAGAAAG----------------------------------------------

------------------------------------------------------------

-------------------------------------------TTGTTAATGTTTACAAC

TGAAATATGTTTTGTTATTAAAATGAGATCATTCAAATATTCAAACAAATATTTTTT-CT

TTATACGTGTTGTTCAGTTCGTAGTTTTCTATGCTATGCTTTGTATACTGTTGTTTTTCT

TTTTTTT-CTCCTTTTTTT----GCCATGGCGTTGTCAGTTTACTTTCAACTTAAGATAA

TAAATGTTCC-TGTGGTATATTT--AGCCTCTCTCTAAGACAGGCATCG---ATTTGTGT

CGAAA-CTTAAGCAATTTTATTC-G-TTTTATTTTT-CTAC------TTATTCA--GTTC

AGGAAGCCCAATCAGTAGCTTGTAGATCATACTACTGTAGTAAGTTCTGTGGGTCTGCTG

GTTGCTCATTATATGGATGTTACCTACTTCATCCTGGAAAAATTTGCTACTGCCTTCATT

GTAGCAGAGCTGAGTCTCCATTGGCACTTTCTGGAAGCGCTAGGAATGTGAACGACAAGA

ACAACGAGATGGACAACTCTCCAGTGTAAGTACTCAATACTAGATTTCGTTACACAATAT

AAA-ATTTGAAAA---AGAAAAAAAA------GTTTTCTACATGCCAACATATATTTTAA

TTAAGGTGTTTACCAATTTTTGAAACAGATAATTTG-T-TTTTTTTTAACTAAAATATTT

CATTTT-----AATTCCATATGTCGTTATCGTTTGAATCGTATTA------TGCAGTCAT

TGAAAAAAAA---AAATACCTGAACTACTCCTGCAAGAAAAAGAATGGAT-GT-TTT-TT

TCTAAGATCGGTTAATGTGTTGGGAAGCAGATAAGATATCTTCTAGTGTGTCTCTCAATT

TATTTACATTCAAAAACATTTTTTT----ACATAAATCGATCACTGGTCAATTGCACGAG

AGTAGTCAGAAAAAAAAC--TGTTATATAAAACGTTTTAATTGAATTTGTATAAAAAA-C

GCCACATATAAGAGACCATCTACTTTGTTTCAGGATGAATGAGGTGGAAAATTTGGACCA

AGAAATGGAAATGTTCTAG

>21_09Gen

ATGAAGGCAACGATCTTGTTAGCTGTTGTAGTGGCAGTCATTGTTGGAGGTAGATATCTT

TATTGAAATATATCTGTGTGTACGTATTTTAGAAGTATAGATCCAGCATTTTT-AGGATA

AATATACGGCAGACCGACTTTTCTGAAA--------------------------------

-------------ATTTTACTACTTTATTGTAATAATTTTTCATTGA---TAATAAATGT

TTTAAGTAAGAAAG----------------------------------------------

------------------------------------------------------------

-------------------------------------------TTGTTAATGTTTACAAC

TGAAATATGTTTTGTTATTAAAATGAGATCATTCAAATATTCAAACAAATATTTTTT-CT

TTATACGTGTTGTTCAGTTCGTAGTTTTCTATGCTATGCTTTGTATACTGTTGTTTTTCT

TTTTTTT-CTCCTTTTTTT----GCCATGGCGTTGTCAGTTTACTTTCAACTTAAGATAA

TAAATGTTCC-TGTGGTATATTT--AGCCTCTCTCTAAGACAGGCATCG---ATTTGTGT

CGAAA-CTTAAGCAATTTTATTC-A-TTTTATTTTT-CTAC------TTATTCA--GTTC

AGGAAGCCCAATCAGTAGCTTGTAGATCATACTACTGTAGTAAGTTCTGTGGGTCTGCTG

GTTGCTCATTATATGGATGTTACCTACTTCATCCTGGAAAAATTTGCTACTGCCTTCATT

GTAGCAGAGCTGAGTCTCCATTGGCACTTTCTGGAAGCGCTAGGAATGTGAACGACAAGA

ACAACGAGATGGACAACTCTCCAGTGTAAGTACTCAATACTAGATTTCGTTACACAATAT

AAA-ATTTGAAAA---AGAAAAAAAA------GTTTTCTACATGCCAACATATATTTTAA

TTAAGGTGTTTACCAATTTTTGAAACAGATAATTTG-T-TTTTTTTTAACTAAAATATTT

CATTTT-----AATTCCATATGTCGTTATCGTTTGAATCGTATTA------TGCAGTCAT

TGAAAAAAAA---AA-TACCTGAACTACTCCTG-AAGAAAAAGAATGGAT-GT-TTT-TT

TCTAAGATCGGTTAATGTGTTGGGAAGCAGATAAGATATCTTCTAGTGTGTCTCTCAATT

TATTTACATTCAAAAACATTTTTTT----ACATAAATCGATCACTGGTCAATTGCACGAG

AGTAGTCAGAAAAAAAAC--TGTTATATAAAACGTTTTAATTGAATTTGTATAAAAAA-C

GCCACATATAAGAGACCATCTACTTTGTTTCAGGATGAATGAGGTGGAAAATTTGGACCA

AGAAATGGAAATGTTCTAG

>21_10Gen

ATGAAGGCAACGATCTTGTTAGCTGTTGTAGTGGCAGTCATTGTTGGAGGTAAATATCTT

TATTGAAATATATCTGTGTATGCGTATTTTAGAAGTATAGATCCAGCATTTTT-AAGATA

AAAAAACGGCAGACCGACTTCTCAAAAATTTGAGTGCGAATCCCATATT---------TG

TATATTATGTTAAATTATACTACTTTATTGTAATAATTTTTCATTTA---TAATAACTGT

TTTAAGTAAGAAAGCTGTAATTGGCAAAACTTTTAGGAATTTTGGTCCCCAATGCTCTTC

TTTTT--AACTTTTATGATTCGAGCGTCACTGATGAGTCTTTTGCAGACGAAACGCGCGT

CTTGCGTATACGTAATTTAGTTCTGGTATCTACGATGAGTTTATTGTTAATGTTTACAAC

TGAAATATGCTTTGTTATTAAAATTAAATCACTCAAATACTCAAACAAATATTTTTT-CT

TTATACGTGTTGTTCAGTTCGTAGTTTTCTATGCTATGCTTTGTATACTGTTGTTTGTCT

TTTTTTTTCTCCTTTTTTTTTT-GCCATGGCGTTGTCAGTTCACTTTCAACGTATGATAA

TAAATGTCCC-TGTGGTATATTT--AGCCTCTCTCTAAGACAGTCATCGTT-ATTTGTGT

CGAAA-CTTGAGCAATTTTATTC-A-TTTTATTTTT-CTAC------TTATTCA--GTTC

AGGAAGCCCAATCAGTAGCTTGTACATCATACTACTGTAGTAAGTTCTGTGGGTCTGCTG

GTTGCTCATTATATGGATGTTACCTACTTCATCCTGGAAAAATTTGCTACTGCCTTCATT

GTCGCAGAGCTGAGTCTCCATTGGCACTTTCTGGAAGCGCTAGGAATGGGAACGACAAGA

ACAACGAGATGGACAACTCTCCAGTGTAAGTACTCAATACTAGATTTCGTTACACAATAT

AAA-ATTT---------GAAAAAAAA------GTTTTCTACATGCCAACATATATTGTAA

TTAAGGTGTTTACCAATTTTTGAAACAGATGATTTGAT-TTTTTTTTAAATAAAATATTT

CATTTT-----AATTCAATATGTCGTTATCGTTTGAATCGTATTA------TGCAGTCAT

TGAAAAAAAAAATAAACACCTGAACTACTCCTGCAAAAGAAGGAAGGGAT-GT-TTTG-T

TTT-ATATTGGTTAATGTGTTGGGAGGCAGATAAGATATCTTCTAGTGTGTCTCTCAATT

-ATTTACATTCAAAAACATTTTTTTTTT-ACATTAATCGATCACTGGTCAATTGCACGAG

AGTAGTCAGAAAAAA--CCCTGTTATATGAAACGTTTTAATTGAATTTGTGAAAATAA-C

ACCACATATAAGAGAACATCTACTTTGTTTCAGGATGAACGAGATCGAAAATTTGGACCA

AGAAATGGATATGTTCTAG

>21_12Gen

ATGAAGGCAACGATCTTGTTAGCTGTTGTAGTGGCAGTCATTGTTGGAGGTAAATATCTT

TATTGAAATATATCTGTGTGTACGTATTTTAGAAGTATAGATCCAGCATTTTT-AGGATA

AATATACGGCAGACCGACTTTTCTGAAA--------------------------------

-------------ATTTTACTACTTTATTGTAATAATTTTTCATTGA---TAATAAATGT

TTTAAGTAAGAAAG----------------------------------------------

------------------------------------------------------------

-------------------------------------------TTGTTAATGTTTACAAC

TGAAATATGTTTTGTTATTAAAATGAGATCATTCAAATATTCAAACAAATATTTTTT-CT

TTATACGTGTTGTTCAGTTCGTAGTTTTCTATGCTATGCTTTGTATACTGTTGTTTTTCT

TTTTTTT-CTCCTTTTTTT----GCCATGGCGTTGTCAGTTTACTTTCAACTTAAGATAA

TAAATGTTCC-TGTGGTATATTT--AGCCTCTCTCTAAGACAGGCATCG---ATTTGTGT

CGAAA-CTTAAGCAATTTTATTC-A-TTTTATTTTT-CTAC------TTATTCA--GTTC

AGGAAGCCCAATCAGTAGCTTGTAGATCATACTACTGTAGTAAGTTCTGTGGGTCTGCTG

GTTGCTCATTATATGGATGTTACCTACTTCATCCTGGAAAAATTTGCTACTGCCTTCATT

GTAGCAGAGCTGAGTCACCATTGGCACTTTCTGGAAGCGCTAGGAATGTGAACGACAAGA

ACAACGAGATGGACAACTCTCCAGTGTAAGTACTCAATACTAGATTTCGTTACACAATAT

AAA-ATTTAA-------GAAAAAAAA------GTTTTCTACATGCCAACATATATTTTAA

TTAAGGTGTTTACCAATTTTTGAAACAGATAATTTG-T-TTTTTTTTAACTAAAATATTT

CATCTT-----AATTCCATATGTCGTTATCGTCTGAATCGTATTA------TGCAGTCAT

TGAAAAAAAA----AATACCTGAACTACTCCTGCAAGAAAAAGAATGGAT-GT-TTT-TT

TCTAAGATCGGTTAATGTGTTGGGAAGCAGATAAGATATCTTCTAGTGTGTCTCTCAGTT

TATTTACATTCAAAAACATTTTTTT----ACATAAATCGATCACTGGTCAATTGCACGAG

AGTAGTCAGAAAAAAAAC--TGTTATATAAAACGTTTTAATTGAATTTGTATAAAAAA-C

GCCACATATAAGAGACCATCTACTTTGTTTCAGGATGAATGAGGTGGAAAATTTGGACCA

AGAAATGGAAATGTTCTAG

>21_13Gen

ATGAAGGCAACGATCTTGTTAGCTGTTGTAGTGGCAGTCATTGTTGGAGGTAAATATCTT

TATTGAAATATATCTGTGTGTACGTATTTTAGAAGTATAGATCCAGCATTTTT-AGGATA

AATATACGGCAGACCGACTTTTCTGAAA--------------------------------

-------------ATTTTACTACTTTATTGTAATAATTTTTCATTGA---TAATAAATGT

TTTAAGTAAGAAAG----------------------------------------------

------------------------------------------------------------

-------------------------------------------TTGTTAATGTTTACAAC

TGAAATATGTTTTGTTATTAAAATGAGATCATTCAAATATTCAAACAAATATTTTTT-CT

TTATACGTGTTGTTCAGTTCGTAGTTTTCTATGCTATGCTTTGTATACTGTTGTTTTTCT

TTTTTTT-CTCCTTTTTTT----GCCATGGCGTTGTCAGTTTACTTTCAACTTAAGATAA

TAAATGTTCC-TGTGGTATATTT--AGCCTCTCTCTAAGACAGGCATCG---ATTTGTGT

CGAAA-CTTAAGCAATTTTATTC-A-TTTTATTTTT-CTAC------TTATTCA--GTTC

AGGAAGCCCAATCAGTAGCTTGTAGATCATACTACTGTAGTAAGTTCTGTGGGTCTGCTG

GTTGCTCATTATATGGATGTTACCTACTTCATCCTGGAAAAATTTGCTACTGCCTTCATT

GTAGCAGAGCTGAGTCTCCATTGGCACTTTCTGGAAGCGCTAGGAATGTGAACGACAAGA

ACAACGAGATGGACAACTCTCCAGTGTAAGTACTCAATACTAGATTTCGTTACACAATAT

AAA-ATTTGAAAA---AGAAAAAAAA------GTTTTCTACATGCCAACATATATTTTAA

TTAAGGTGTTTACCAATTTTTGAAACAGATAATTTG-T-TTTTTTTTAACTAAAATATTT

CATTTT-----AATTCCATATGTCGTTATCGTTTGAATCGTATTA------TGCAGTCAT

TGAAAAAAAA---AA-TACCTGAACTACTCCTGCAAGAAAAAGAATGGAT-GT-TTT-TT

TCTAAGATCGGTTAATGTGTTGGGAAGCAGATAAGATATCTTCTAGTGTGTCTCTCAATT

TATTTACATTCAAAAACATTTTTTT----ACATAAATCGATCACTGGTCAATTGCACGAG

AGTAGTCAGAAAAAAAAC--TGTTATATAAAACGTTTTAATTGAATTTGTATAAAAAA-C

GCCACATATAAGAGACCATCTACTTTGTTTCAGGATGAATGAGGTGGAAAATTTGGACCA

AGAAATGGAAATGTTCTAG

>21_14Gen

ATGAAGGCAACGATCTTGTTAGCTGTTGTAGTGGCAGTCATTGTTCGAGGTAAATATCTT

TATTGAAATATATTTGTGTGTACGTATTTTAGAAGTATAGATCCAGCATTTTT-AGGATA

AATATACGGCAGACCGACTTTTCTGAAA--------------------------------

-------------ATTTTACTACTTTATTGTAATAATTTTTCATTGA---TAATAAATGT

TTTAAGTAAGAAAG----------------------------------------------

------------------------------------------------------------

-------------------------------------------TTGTTAATGTTTACAAC

TGAAATATGTTTTGTTATTAAAATGAGATCATTCAAATATTCAAACAAATATTTTTT-CT

TTATACGTGTTGTTCAGTTCGTAGTTTTCTATGCTATGCTTTGTATACTGTTGTTTTTCT

TTTTTTT-CTCCTTTTTTT----GCCATGGCGTTGTCAGTTTACTTTCAACTTAAGATAA

TAAATGTTCC-TGTGGTATATTT--AGCCTCTCTCTAAAACAGGCATCG---ATTTGTGT

CGAAA-CTTAAGCAATTTTATTC-A-TTTTATTTTT-CTAC------TTATTCA--GTTC

AGGAAGCCCAATCAGTAGCTTGTAGATCATACTACTGTAGTAAGTTCTGTGGGTCTGCTG

GTTGCTCATTATATGGATGTTACCTACTTCATCCTGGAAAAATTTGCTACTGCCTTCATT

GTAGCAGAGCTGAGTCTCCATTGGCACTTTCTGGAAGCGCTAGGAATGTGAACGACAAGA

ACAACGAGATGGACAACTCTCCAGTGTAAGTACTCAATACTAGATTTCGTTACACAATAT

AAA-ATTTGAAAA----GAAAAAAAA------GTTTTCTACATGCCAACATATATTTTAA

TTAAGGTGTTTACCAATTTTTGAAACAGATAATTTG-T-TTTTTTTTAACTAAAATATTT

CATCTT-----AATTCCATATGTCGTTATCGTCTGAATCGTATTA------TGCAGTCAT

TGAAAAAAAA---A--TACCTGAACTACTCCTGCAAGAAAAAGAATGGAT-GT-TTT-TT

TCTAAGATCGGTTAATGTGTTGGGAAGCAGATAAGATATCTTCTAGTGTGTCTCTCAATT

TATTTACATTCAAAAACATTTTTTT----ACATAAATCGATCACTGGTCAATTGCACGAG

AGTAGTCAGAAAAAAAAC--TGTTATATAAAACGTTTTAATTGAATTTGTATAAAAAA-C

GCCACGTATAAGAGACCATCTACTTTGTTTCAGGATGAATGAGGTGGAAAATTTGGACCA

AGAAATGGAAATGTTCTAG

>21_15Gen

ATGAAGGCAACGATCTTGTTAGCTGTTGTAGTGGCAGTCATTGTTGGAGGTAAATATCTT

TATTGAAATATATCTGTGTGTACGTATTTTAGAAGTATAGATCCAGCATTTTT-AGGATA

AATATACGGCAGACCGACTTTTCTGAAA--------------------------------

-------------ATTTTACTACTTTATTGTAATAATTTTTCATTGA---TAATAAATGT

TTTAAGTAAGAAAG----------------------------------------------

------------------------------------------------------------

-------------------------------------------TTGTTAATGTTTACAAC

TGAAATATGTTTTGTTATTAAAATGAGATCATTCAAATATTCAAACAAATATTTTTT-CT

TTATACGTGTTGTTCAGTTCGTAGTTTTCTATGCTATGCTTTGTATACTGTTGTTTTTCT

TTTTTTT-CTCCTTTTTTT----GCCATGGCGTTGTCAGTTTACTTTCAACTTAAGATAA

TAAATGTTCC-TGTGGTATATTT--AGCCTCTCTCTAAGACAGGCATCG---ATTTGTGT

CGAAA-CTTAAGCAATTTTATTC-A-TTTTATTTTT-CTAC------TTATTCA--GTTC

AGGAAGCCCAATCAGTAGCTTGTAGATCATACTACTGTAGTAAGTTCTGTGGGTCTGCTG

GTTGCTCATTATATGGATGTTACCTACTTCATCCTGGAAAAATTTGCTACTGCCTTCATT

GTAGCAGAGCTGAGTCTCCATTGGCACTTTCTGGAAGCGCTAGGAATGTGAACGACAAGA

ACAACGAGATGGACAACTCTCCAGTGTAAGTACTCAATACTAGATTTCGTTACACAATAT

AAA-ATTTGAA------GAAAAAAAA------GTTTTCTACATGCCAACATATATTTTAA

TTAAGGTGTTTACCAATTTTTGAAACAGATAATTTG-T-TTTTTTTTAACTAAAATATTT

CATCTT-----AATTCCATATGTCGTTATCGTTTGAATCGTATTA------TGCAGTCAT

TGAAAAAAAA---AAATACCTGAACTACTCCTGCAAGAAAAAGAATGGAT-GT-TTT-TT

TCTAAGATCGGTTAATGTGTTGGGAAGCAGATAAGATATCTTCTAGTGTGTCTCTCAATT

TATTTACATTCAAAAACATTTTTTTT---ACATAAATCGATCACTGGTCAATTGCACGAG

AGTAGTCAGAAAAAAAAC--TGTTATATAAAACGTTTTAATTGAATTTGTATAAAAAA-C

GCCACATATAAGAGACCATCTACTTTGTTTCAGGATGAATGAGGTGGAAAATTTGGACCA

AGAAATGGAAATGTTCTAG

>21_16Gen

ATGAAGGCAACGATCTTGTTAGCTGTTGTAGTGGCAGTCATTGTTGGAGGTAAATATCTT

TATTGAAATATATCTGTGTGTACGTATTTTAGAAGTATAGATCCAGCATTTTT-AGGATA

AATATACGGCAGACCGACTTTTCTGAAA--------------------------------

-------------ATTTTACTACTTTATTGTAATAATTTTTCATTGA---TAATAAATGT

TTTAAGTAAGAAAG----------------------------------------------

------------------------------------------------------------

-------------------------------------------TTGTTAATGTTTACAAC

TGAAATATGTTTTGTTATTAAAATGAGATCATTCAAATATTCAAACAAATATTTTTT-CT

TTATACGTGTTGTTCAGTTCGTAGTTTTCTATGCTATGCTTTGTATACTGTTGTTTTTCT

TTTTTTT-CTCCTTTTTTT----GCCATGGCGTTGTCAGTTTACTTTCAACTTAAGATAA

TAAATGTTCC-TGTGGTATATTT--AGCCTCTCTCTAAGACAGGCATCG---ATTTGTGT

CGAAA-CTTAAGCAATTTTATTC-A-TTTTATTTTT-CTAC------TTATTCA--GTTC

AGGAAGCCCAATCAGTAGCTTGTAGATCATACTACTGTAGTAAGTTCTGTGGGTCTGCTG

GTTGCTCATTATATGGATGTTACCTACTTCATCCTGGAAAAATTTGCTACTGCCTTCATT

GTAGCAGAGCTGAGTCTCCATTGGCACTTTCTGGAAGCGCTAGGAATGTGAACGACAAGA

ACATCGAGATGGACAACTCTCCAGTGTAAGTACTCAATACTAGATTTCGTTACACAATAT

AAA-ATTT---------GAAAAAAAA------GTTTACTACATGCCAACATATATTTTAA

TTAAGGTGTTTACCAATTTTTGAAACAGATAATTTG-T-TTTTTTTTAACTAAAATATTT

CATCTT-----AATTCCATATGTCGTTATCGTCTGAATCGTATTA------TGCAGTCAT

TGAAAAAAAA---AAATACCTGAACTACTCCTGCAAGAAAAAGAATGGAT-GT-TTT-TT

TCTAAGATCGGTTAATGTGTTGGGAAGCAGATAAGATATCTTCTAGTGTGTCTCTCAATT

TATTTACATTCAAAAACATTTTTTT----ACATAAATCGATCACTGGTCAATTGCACGAG

AGTAGTCAGAAAAAAAAC--TGTTATATAAAACGTTTTAATTGAATTTGTATAAAAAA-C

GCCACATATAAGAGACCATCTACTTTGTTTCAGGATGAATGAGGTGGAAAATTTGGACCA

AGAAATGGAAATGTTCTAG

>21_17Gen

ATGAAGGCAACGATCTTGTTAGCTGTTGTAGTGGCAGTCATTGTTGGAGGTAAATATCTT

TATTGAAATATATCTGTGTGTACGTATTTTAGAAGTATAGATCCAGCATTTTT-AGGATA

AATATACGGCAGACCGACTTTTCTGAAA--------------------------------

-------------ATTTTACTACTTTATTGTAATAATTTTTCATTGA---TAATAAATGT

TTTAAGTAAGAAAG----------------------------------------------

------------------------------------------------------------

-------------------------------------------TTGTTAATGTTTACAAC

TGAAATATGTTTTGTTATTAAAATGAGATCATTCAAATATTCAAACAAATATTTTTT-CT

TTATACGTGTTGTTCAGTTCGTAGTTTTCTATGCTATGCTTTGTATACTGTTGTTTTTCT

TTTTTTT-CTCCTTTTTTT----GCCATGGCGTTGTCAGTTTACTTTCAACTTAAGATAA

TAAATGTTCC-TGTGGTATATTT--AGCCTCTCTCTAAGACAGGCATCG---ATTTGTGT

CGAAA-CTTAAGCAATTTTATTC-A-TTTTATTTTT-CTAC------TTATTCA--GTTC

AGGAAGCCCAATCAGTAGCTTGTAGATCATACTACTGTAGTAAGTTCTGTGGGTCTGCTG

GTTGCTCATTATATGGATGTTACCTACTTCATCCTGGAAAAATTTGCTACTGCCTTCATT

GTAGCAGAGCTGAGTCTCCATTGGCACTTTCTGGAAGCGCTAGGAATGTGAACGACAAGA

ACAACGAGATAGACAACTCTCCAGTGTAAGTACTCAATACTAGATTTCGTTACACAATAT

AAA-ATTT---------GAAAAAAAA------GTTTTCTACATGCCAACATATATTTTAA

TTAAGGTGTTTACCAATTTTTGAAACAGATAATTTG-T-TTTTTTTTAACTAAAATATTT

CATCTT-----AATTCCATATGTCGTTATCGTCTGAATCGTATTA------TGCAGTCAT

TGAAAAAAAA---AAATACCTGAACTACTCCTGCAAGAAAAAGAATGGAT-GT-TTT-TT

TCTAAGATCGGTTAATGTGTTGGGAAGCAGATAAGATATCTTCTAGTGTGTCTCTCAATT

TATTTACATTCAAAAACATTTTTTT----ACATAAATCGATCACTGGTCAATTGCACGAG

AGTAGTCAGAAAAAAAAC--TGTTATATAAAACGTTTTAATTGAATTTGTATAAAAAA-C

GCCACATATAAGAGACCATCTACTTTGTTTCAGGATGAATGAGGTGGAAAATTTGGACCA

AGAAATGGAAATGTTCTAG

>21_18Gen

ATGAAGGCAACGATCTTGTTAGCTGTTGTAGTGGCAGTCATTGTTGGAGGTAAATATCTT

TATTGAAATATATCTGTGTGTACGTATTTTAGAAGTATAGATCCAGCATTTTT-AGGATA

AATATACGGCAGACCGACTTTTCTGAAA--------------------------------

-------------ATTTTACTACTTTATTGTAATAATTTTTCATTGA---TAATAAATGT

TTTAAGTAAGAAAG----------------------------------------------

------------------------------------------------------------

-------------------------------------------TTGTTAATGTTTACAAC

TGAAATATGTTTTGTTATTAAAATGAGATCATTCAAATATTCAAACAAATATTTTTT-CT

TTATACGTGTTGTTCAGTTCGTAGTTTTCTATGCTATGCTTTGTATACTGTTGTTTTTCT

TTTTTTT-CTCCTTTTTTT----GCCATGGCGTTGTCAGTTTACTTTCAACTTAAGATAA

TAAATGTTCC-TGTGGTATATTT--AGCCTCTCTCTAAGACAGGCATCG---ATTTGTGT

CGAAA-CTTAAGCAATTTTATTC-A-TTTTATTTTT-CTAC------TTATTCA--GTTC

AGGAAGCCCAATCAGTAGCTTGTAGATCATACTACTGTAGTAAGTTCTGTGGGTCTGCTG

GTTGCTCATTATATGGATGTTACCTACTTCATCCTGGAAAAATTTGCTACTGCCTTCATT

GTAGCAGAGCTGAGTCTCCATTGGCACTTTCTGGAAGCGCTAGGAATGTGAACGACAAGA

ACAACGAGATGGACAACTCTCCAGTGTAAGTACTCAATACTAGATTTCGTTACACAATAT

AAA-ATTT---------GAAAAAAAA------GTTTTCTACATGCCAACATATATTTTAA

TTAAGGTGTTTACCAATTTTTGAAACAGATAATTTG-T-TTTTTTTTAACTAAAATATTT

CATCTT-----AATTCCATATGTCGTTATCGTCTGAATCGTATTA------TGCAGTCAT

TGAAAAAAAA---AAATACCTGAACTACTCCTGCAAGAAAAAGAATGGAT-GT-TTT-TT

TCTAAGATCGGTTAATGTGTTGGGAAGCAGATAAGATATCTTCTAGTGTGTCTCTCAATT

TATTTACATTCAAAAACATTTTTTT----ACATAAATCGATCACTGGTCAATTGCACGAG

AGTAGTCAGAAAAAAAAC--TGTTATATAAAACGTTTTAATTGAATTTGTATAAAAAA-C

GCCACATATAAGAGACCATCTACTTTGTTTCAGGATGAATGAGGTGGAAAATTTGGACCA

AGAAATGGAAATGTTCTAG

>21_19Gen

ATGAAGGCAACGATCTTGTTAGCTGTTGTAGTGGCAGTCATTGTTGGAGGTAAATATCTT

TATTGAAATATATCTGTGTGTACGTATTTTAGAAGTATAGATCCAGCATTTTT-AGGATA

AATATACGGCAGACCGACTTTTCTGAAA--------------------------------

-------------ATTTTACTACTTTATTGTAATAATTTTTCATTGA---TAATAAATGT

TTTAAGTAAGAAAG----------------------------------------------

------------------------------------------------------------

-------------------------------------------TTGTTAATGTTTACAAC

TGAAATATGTTTTGTTATTAAAATGAGATCATTCAAATATTCAAACAAATATTTTTT-CT

TTATACGTGTTGTTCAGTTCGTAGTTTTCTATGCTATGCTTTGTATACTGTTGTTTTTCT

TTTTTTT-CTCCTTTTTTT----GCCATGGCGTTGTCAGTTTACTTTCAACTTAAGATAA

TAAATGTTCC-TGTGGTATATTT--AGCCTCTCTTTAAGACAGGCATCG---ATTTGTGT

CGAAA-CTTAAGCAATTTTATTC-A-TTTTATTTTT-CTAC------TTATTCA--GTTC

AGGAAGCCCAATCAGTAGCTTGCAGATCATACTACTGTAGTAAGTTCTGTGGGTCTGCTG

GTTGCTCATTATATGGATGTTACCTACTTCATCCTGGAAAAATTTGCTACTGCCTTCATT

GTAGCAGAGCTGAGTCTCCATTGGCACTTTCTGGAAGCGCTAGGAATGTGAACGACAAGA

ACAACGAGATGGACAACTCTCCAGTGTAAGTACTCAATACTAGATTTCGTTACACAATAT

AAA-ATTT---------GAAAAAAAA------GTTTTCTACATGCCAACATATATTTTAA

TTAAGGTGTTTACCAATTTTTGAAACAGATAATTTG-T-TTTTTTTTAACTAAAATATTT

CATCTT-----AATTCCATATGTCGTTATCGTCTGAATCGTATTA------TGCAGTCAT

TGAAAAAAAA---AAATACCTGAACTACTCCTGCAAGAAAAAGAATGGAT-GT-TTT-TT

TCTAAGATCGGTTAATGTGTTGGGAAGCAGATAAGATATCTTCTAGTGTGTCTCTCAATT

TATTTACATTCAAAAACATTTTTTT----ACATAAATCGATCACTGGTCAATTGCACGAG

AGTAGTCAGAAAAAAAAC--TGTTATATAAAACGTTTTAATTGAATTTGTATAAAAAA-C

GCCACATATAAGAGACCATCTACTTTGTTTCAGGATGAATGAGGTGGAAAATTTGGACCA

AGAAATGGAAATGTTCTAG

>21_20Gen

ATGAAGGCAACGATCTTGTTAGCTGTTGTAGTGGCAGTCATTGTTGGAGGTAAATATCTT

TATTGAAATATATCTGTGTGTACGTATTTTAGAAGTATAGATCCAGCATTTTT-AGGATA

AATATACGGCAGACCGACTTTTCTGAAA--------------------------------

-------------ATTTTACTACTTTATTGTAATAATTTTTCATTGA---TAATAAATGT

TTTAAGTAAGAAAG----------------------------------------------

------------------------------------------------------------

-------------------------------------------TTGTTAATGTTTACAAC

TGAAATATGTTTTGTTATTAAAATGAGATCATTCAAATATTCAAACAAATATTTTTT-CT

TTATACGTGTTGTTCAGTTCGTAGTTTTCTATGCTATGCTTTGTATACTGTTGTTTTTCT

TTTTTTT-CTCCTTTTTTT----GCCATGGCGTTGTCAGTTTACTTTCAACTTAAGATAA

TAAATGTTCC-TGTGGTATATTT--AGCCTCTCTTTAAGACAGGCATCG---ATTTGTGT

CGAAA-CTTAAGCAATTTTATTC-A-TTTTATTTTT-CTAC------TTATTCA--GTTC

AGGAAGCCCAATCAGTAGCTTGCAGATCATACTACTGTAGTAAGTTCTGTGGGTCTGCTG

GTTGCTCATTATATGGATGTTACCTACTTCATCCTGGAAAAATTTGCTACTGCCTTCATT

GTAGCAGAGCTGAGTCTCCATTGGCACTTTCTGGAAGCGCTAGGAATGTGAACGACAAGA

ACAACGAGATGGACAACTCTCCAGTGTAAGTACTCAATACTAGATTTCGTTACACAATAT

AAA-ATTT---------GAAAAAAAA------GTTTTCTACATGCCAACATATATTTTAA

TTAAGGTGTTTACCAATTTTTGAAACAGATAATTTG-T-TTTTTTTTAACTAAAATATTT

CATCTT-----AATTCCATATGTCGTTATCGTCTGAATCGTATTA------TGCAGTCAT

TGAAAAAAAA---AAATACCTGAACTACTCCTGCAAGAAAAAGAATGGAT-GT-TTT-TT

TCTAAGATCGGTTAATGTGTTGGGAAGCAGATAAGATATCTTCTAGTGTGTCTCTCAATT

TATTTACATTCAAAAACATTTTTTT----ACATAAATCGATCACTGGTCAATTGCACGAG

AGTAGTCAGAAAAAAAAC--TGTTATATAAAACGTTTTAATTGAATTTGTATAAAAAA-C

GCCACATATAAGAGACCATCTACTTTGTTTCAGGATGAATGAGGTGGAAAATTTGGACCA

AGAAATGGAAATGTTCTAG

>21_22Gen

ATGAAGGCAACGATCTTGTTAGCTGTTGCAGTGGCAGTCATTGTTGGAGGTAAATATCTT

TATTGAAATATATCTGTGTGTACGTATTTTAGAAGTATAGATCCAATATTTTT-AAGATA

AAAATACGGCAGACCGACTTTTCCCAAATTTGATTGCGAAACCCATATT---------TG

TATATTATGTAAAATTGTACTACTTTATTGCAATAATTTTTCATTAA---TAAATACTGT

TTTATGTAAGATAA----------------------------------------------

------------------------------------------------------------

------------------------------------------ATTGTTAATGTTTACAAC

TGAAATATGTTTTGTTATTAAAATTCAATCATTCAAATATTCAAACAAACATTTTTTTCT

TTATACGTGTTGTTCAGTTTGTAGTTTTCTATGCTATGCTTTGTATACTGTTGTTTGTCT

TTT--------------------GCCATGTCGTTGTCAGTAGATTTTTAACTTATGATAT

CGAATGTCCC-TGTGGTATATTT--TGTCTCTCTTTTAGATAGGCATCGTT-ATTTGTG-

CATTA-TTTAATCAATTTTATTTTA-TTTCATTTTTTCTCCGTTTTTTATTTATAAGTTC

AGGAAGTCCAATCAGTACCTTGTGCATCAACCTTGTGTAGTAGGTTCTGTGGGTCTGCTG

GTTGCAGATTATATGGATGTTACAGACTTCATCCCGGCAAAATTTGCTACTGCCTTCATT

GTCGCAGAGCTGAGTCTCCATTGGCACTTTCTGGAAGCGCTAGGAATGTGAACGATCAGA

ACAAAGAGATGGACAACTCTCCAATGTAAGTA--CAATACAAGACTTCGTTACATAATAT

AAACGTTTACAAATTTACAAAAGAAAAACATTGTATTCTACATGCCAACATATACTGTAT

TTAAGGGA--TATCATTTTTTTAAACAGATAATTTGAATTTTTTTTTAGATAAAGCATTT

CATCTTTGTTTAATTCAATAGGTCGTTATCGTCTG--------CA------CGTATTATT

TACAGTCATA----AACACATGAACTACTCCTGCAAAAGAAGGAAGGGAT-GT-TTT-TT

TCTTAGATCGGTTAATGTGTTGGGAGGCAGATAAGATATCTTGTATTGTGTCTCACAATT

-ATTTACATTCAAAAACATTTTT------ACATAAATCGATCACTGGTCAATTGCACCCG

AATAGTCAGAAAAAAAAAC-TGTTATATGAAACGTTTTAATTGAATTTGTGAAAAAAA-C

GCCACATATAAGAGAACAACTTATTTGTTTCAGGATGAATGAGATGGAACATTTGGACCA

AGAAATGGATATGTTCTAG

>21_24Gen

ATGAAGGCAACGATCTTGTTAGCTGTTGTAGTGGCAGTCATTGTTGGAGGTAAATATCTT

TATTGAAATATATCTGTGTGTACGTATTTTAGAAGTATAGATCCAATATTTTT-AAGATA

AAAATACGGCAGACCGACTTTTCCCAAATTTGATTGCGAAACCCATATT---------TG

TATATTATGTAAAATTGTACTACTTTATTGCAATAATTTTTCATTAA---TAAATACTGT

TTTATGTAAGATAA----------------------------------------------

------------------------------------------------------------

------------------------------------------ATTGTTAATGTTTACAAC

TGAAATATGTTTTGTTATTAAAATTCAATCATTCAAATATTCAAACAAACATTTTTTTCT

TTATACGTGTTGTTCAGTTTGTAGTTTTCTATGCTATGCTTTGTATACTGTTGTTTGTCT

TTT--------------------GCCATGTCGTTGTCAGTAGATTTTTAACTTATGATAT

CGAATGTCCC-TGTGGTATATTT--TGTCTCTCTTTTAGACAGGCATCGTT-ATTTGTG-

CATTA-TTTAATCAATTTTATTTTA-TTTCATTTTTTCTCCGTTTTTTATTTATAAGTTC

AGGAAGTCCAATCAGTACCTTGTGCATCAACCTTGTGTAGTAGGTTCTGTGGGTCTGCTG

GTTGCAGATTATATGGATGTTACAGACTTCATCCCGGCAAAATTTGCTACTGCCTTCATT

GTCGCAGAGCTGAGTCTCCATTGGCACTTTCTGGAAGCGCTAGGAATGTGAACGATCAGA

ACAAAGAGATGGACAACTCTCCAATGTAAGTA--CAATACAAGACTTCGTTACATAATAT

AAACGTTTACAAATTTACAAAAGAAAAACATTGTATTCTACATGCCAACATATACTGTAT

TTAAGGGA--TATCATTTTTTTAAACAGATAATTTGAATTTTTTTTTAGATAAAGCATTT

CATCTTTGTTTAATTCAATAGGTCGTTATCGTCTG--------CA------CGTATTATT

TACAGTCATA----AACACATGAACTACTCCTGCAAAAGAAGGAAGGGAT-GT-TTT-TT

TCTTAGATCGGTTAATGTGTTGGGAGGCAGATAAGATATCTTGTATTGTGTCTCACAATT

-ATTTACATTCAAAAACATTTTT------ACATAAATCGATCACTGGTCAATTGCACCCG

AATAGTCAGAAAAAAAAAC-TGTTATATGAAACGTTTTAATTGAATTTGTGAAAAAAA-C

GCCACATATAAGAGAACAACTTATTTGTTTCAGGATGAATGAGATGGAACATTTGGACCA

AGAAATGGATATGTTCTAG

>21_25Gen

ATGAAGGCAACGATCTTGTTAGCTGTTGTAGTGGCAGTCATTGTTGGAGGTAAATATCTT

TATTGAAATATATCTGTGTGTACGTATTTTAGAAGTATAGATCCAATATTTTT-AAGATA

AAAATACGGCAGACCGACTTTTCCCAAATTTGATTGCGAAACCCATATT---------TG

TATATTATGTAAAATTGTACTACTTTATTGCAATAATTTTTCATTAA---TAAATACTGT

TTTATGTAAGATAA----------------------------------------------

------------------------------------------------------------

------------------------------------------ATTGTTAATGTTTACAAC

TGAAATATGTTTTGTTATTAAAATTCAATCATTCAAATATTCAAACAAACATTTTTTTCT

TTATACGTGTTGTTCAGTTTGTAGTTTTCTATGCTATGCTTTGTATACTGTTGTTTGTCT

TTT--------------------GCCATGTCGTTGTCAGTAGATTTTTAACTTATGATAT

CGAATGTCCC-TGTGGTATATTT--TGTCTCTCTTTTAGACAGGCATCGTT-ATTTGTG-

CATTA-TTTAATCAATTTTATTTTA-TTTCATTTTTTCTCCGTTTTTTATTTATAAGTTC

AGGAAGTCCAATCAGTACCTTGTGCGTCAACCTTGTGTAGTAGGTTCTGTGGGTCTGCTG

GTTGCAGATTATATGGATGTTACAGACTTCATCCCGGCAAAATTTGCTACTGCCTTCATT

GTCGCAGAGCTGAGTCTCCATTGGCACTTTCTGGAAGCGCTAGGAATGTGAACGATCAGA

ACAAAGAGATGGACAACTCTCCAATGTGAGTA--CAATACAAGACTTCGTTACATAATAT

AAACGTTTACAAATTTACAAAAGAAAAACACTGTATTCTACATGCCAACATATACTGTAT

TTAAGGGA--TATCATTTTTTTAAACAGATAATTTGAATTTTTTTTTAGATGAAGCATTT

CATCTTTGTTTAATTCAATAGGTCGTTATCGTCTG--------CA------CGTATTATT

TACAGTCATA----AACACATGAACTACTCCTGCAAAAGAAGGAAGGGAT-GT-TTT-TT

TCTTAGATCGGTTAATGTGTTGGGAGGCAGATAAGATATCTTGTATTGTGTCTCACAATT

-ATTTACATTCAAAAACATTTTT------ACATAAATCGATCACTGGTCAATTGCACCCG

AATAGTCAGAAAAAAAAAC-TGTTATATGAAACGTTTTAATTGAATTTGTGAAAAAAA-C

GCCACATATAAGAGAACAACTTATTTGTTTCAGGATGAATGAGATGGAACATTTGGACCA

AGAAATGGATATGTTCTAG

>21_27Gen

ATGAAGGCAACGATCTTGTTAGCTGTTGTAGTGGCAGTCATTGTTGGAGGTAAATATCTT

TATTGAAATATATCTGTGTGTACGTATTTTAGAAGTATAGATCCAGCATTTTT-AGGATA

AATATACGGCAGACCGACTTTTCTGAAA--------------------------------

-------------ATTTTACTACTTTATTGTAATAATTTTTCATTGA---TAATAAATGT

TTTAAGTAAGAAAG----------------------------------------------

------------------------------------------------------------

-------------------------------------------TTGTTAATGTTTACAAC

TGAAATATGTTTTGTTATTAAAATGAGATCATTCAAATATTCAAACAAATATTTTTT-CT

TTATACGTGTTGTTCAGTTCGTAGTTTTCTATGCTATGCTTTGTATACTGTTGCTTTTCT

TTTTTTT-CTCCTTTTTTT----GCCATGGCGTTGTCAGTTTACTTTCAACTTAAGATAA

TAAATGTTCC-TGTGGTATATTT--AGCCTCTCTCTAAGACAGGCATCG---ATTTGTGT

CGAAA-CTTAAGCAATTATATTC-A-TTTTATTTTT-CTAC------TTATTCA--GTTC

AGGAAGCCCAATCAGTAGCTTGTAGATCATACTACTGTAGTAAGTTCTGTGGGTCTGCTG

GTTGCTCATTATATGGATGTTACCTACTTCATCCTGGAAAAATTTGCTACTGCCTTCATT

GTAGCAGAGCTGAGTCTCCATTGGCACTTTCTGGAAGCGCAAGGAATGTGAACGACAAGA

ACAACGAGATGGACAACTCTCCAGTGTAAGTACTCAATACTAGATTTCGTTACACAATAT

AAAATTT----------GAAAAAAAA------GTTTTCTACATGCCAACATATATTTTAA

TTAAGGTGTTTACCAATTTTTGAAACAGATAATTTG-T-TTTTTTTTAACTAAAATATTT

CATCTT-----AATTCCATATGTCGTTATCGTCTGAATCGTATTA------TGCAGTCAT

TGAAAAAAAA---AAATACCTGAACTACTCCTGCAAGAAAAAGAATGGAT-GT-TTT-TT

TCTAAGATCGGTTAATGTGTTGGGAAGCAGATAAGATATCTTCTAGTGTGTCTCTCAATT

TATTTACATTCAAAAACATTTTTTT----ACATAAATCGATCACTGGTCAATTGCACGAG

AGTAGTCAGAAAAAAAAC--TGTTATATAAAACGTTTTAATTGAATTTGTATAAAAAA-C

GCCACATATAAGAGACCATCTACTTTGTTTCAGGATGAATGAGGTGGAAAATTTGGACCA

AGAAATGGAAATGTTCTAG

>21_01cDNA

ATGAAGGCAACGATCTTGTTAGCTGTTGTAGTGGCAGTCATTGTTGGAG-----------

------------------------------------------------------------

------------------------------------------------------------

------------------------------------------------------------

------------------------------------------------------------

------------------------------------------------------------

------------------------------------------------------------

------------------------------------------------------------

------------------------------------------------------------

------------------------------------------------------------

------------------------------------------------------------

---------------------------------------------------------TTC

AGGAAGTCCAATCAGTACCTTGTGCATCAACCTTGTGTAGTAGGTTCTGTGGGTCTGCTG

GTTGCAGATTATATGGATGTTACAGACTTCATCCCGGCAAAATTTGCTACTGCCTTCATT

GTCGCAGAGCTGAGTCTCCATTGGCACTTTCTGGAAGCGCTAGGAATGTGAACGATCAGA

ACAAAGAGATGGACAACTCTCCAAT-----------------------------------

------------------------------------------------------------

------------------------------------------------------------

------------------------------------------------------------

------------------------------------------------------------

------------------------------------------------------------

------------------------------------------------------------

------------------------------------------------------------

---------------------------------GATGAATGAGATGGAACATTTGGACCA

AGAAATGGATATGTTCTAG

>21_02cDNA

ATGAAGGCAACGATCTTGTTAGCTGTtGTAGTGGCAGTCATTGTTGGAG-----------

------------------------------------------------------------

------------------------------------------------------------

------------------------------------------------------------

------------------------------------------------------------

------------------------------------------------------------

------------------------------------------------------------

------------------------------------------------------------

------------------------------------------------------------

------------------------------------------------------------

------------------------------------------------------------

---------------------------------------------------------TTC

AGGAAGTCCAATCAGTACCTTGTGCATCAACCTTGTGTAGTAGGTTCTGTGGGTCTGCTG

GTTGCAGATTATATGGATGTTACAGACTTCATCCCGGCAAAATTTGCTACTGCCTTCATT

GTCGCAGAGCTGAGTCTCCATTGGCACTTTCTGGAAGCGCTAGGAATGTGAACGATCAGA

ACAAAGAGATGGACAACTCTCCAAT-----------------------------------

------------------------------------------------------------

------------------------------------------------------------

------------------------------------------------------------

------------------------------------------------------------

------------------------------------------------------------

------------------------------------------------------------

------------------------------------------------------------

---------------------------------GATGAATGAGATGGAACATTTGGACCA

AGAAATGGATATGTTCTAG

>21_03cDNA

ATGAAGGCAACGATCTTGTTAGCTGTTGTAGTGGCAGTCATTGTTGGAG-----------

------------------------------------------------------------

------------------------------------------------------------

------------------------------------------------------------

------------------------------------------------------------

------------------------------------------------------------

------------------------------------------------------------

------------------------------------------------------------

------------------------------------------------------------

------------------------------------------------------------

------------------------------------------------------------

---------------------------------------------------------TTC

AGGAAGTCCAATCAGTACCTTGTGCATCAACCTTGTGTAGTAGGTTCTGTGGGTCTGCTG

GTTGCAGATTATATGGATGTTACAGACTTCATCCCGGCAAAATTTGCTACTGCCTTCATT

GTCGCAGAGCTGAGTCTCCATTGGCACTTTCTGGAAGCGCTAGGAATGTGAACGATCAGA

ACAAAGAGATGGACAACTCTCCAAT-----------------------------------

------------------------------------------------------------

------------------------------------------------------------

------------------------------------------------------------

------------------------------------------------------------

------------------------------------------------------------

------------------------------------------------------------

------------------------------------------------------------

---------------------------------GATGAATGAGATGGAACATTTGGACCA

AGAAATGGATATGTTCTAG

>21_04cDNA

ATGAAGGCAACGATCTTGTTAGCTGTTGTAGTGGCAGTCATTGTTGGAG-----------

------------------------------------------------------------

------------------------------------------------------------

------------------------------------------------------------

------------------------------------------------------------

------------------------------------------------------------

------------------------------------------------------------

------------------------------------------------------------

------------------------------------------------------------

------------------------------------------------------------

------------------------------------------------------------

---------------------------------------------------------TTC

AGGAAGTCCAATCAGTACCTTGTGCATCAACCTTGTGTAGTAGGTTCTGTGGGTCTGCTG

GTTGCAGATTATATGGATGTTACAGACTTCATCCCGGCAAAATTTGCTACTGCCTTCATT

GTCGCAGAGCTGAGTCTCCATTGGCACTTTCTGGAAGCGCTAGGAATGTGAACGATCAGA

ACAAAGAGATGGACAACTCTCCAAT-----------------------------------

------------------------------------------------------------

------------------------------------------------------------

------------------------------------------------------------

------------------------------------------------------------

------------------------------------------------------------

------------------------------------------------------------

------------------------------------------------------------

---------------------------------GATGAATGAGATGGAACATTTGGACCA

AGAAATGGATATGTTCTAG

>21_05cDNA

ATGAAGGCAACGATCTTGTTAGCTGTTGTAGTGGCAGTCATTGTTGGAG-----------

------------------------------------------------------------

------------------------------------------------------------

------------------------------------------------------------

------------------------------------------------------------

------------------------------------------------------------

------------------------------------------------------------

------------------------------------------------------------

------------------------------------------------------------

------------------------------------------------------------

------------------------------------------------------------

---------------------------------------------------------TTC

AGGAAGTCCAATCAGTACCTTGTGCATCAACCTTGTGTAGTAGGTTCTGTGGGTCTGCTG

GTTGCAGATTATATGGATGTTACAGACTTCATCCCGGCAAAATTTGCTACTGCCTTCATT

GTCGCAGAGCTGAGTCTCCATTGGCACTTTCTGGAAGCGCTAGGAATGTGAACGATCAGA

ACAAAGAGATGGACAACTCTCCAAT-----------------------------------

------------------------------------------------------------

------------------------------------------------------------

------------------------------------------------------------

------------------------------------------------------------

------------------------------------------------------------

------------------------------------------------------------

------------------------------------------------------------

---------------------------------GATGAATGAGATGGAACATTTGGACCA

AGAAATGGATATGTTCTAG

>21_06cDNA

ATGAAGGCAACGATCTTGTTAGCTGTTGTAGTGGCAGTCATTGTTGGAG-----------

------------------------------------------------------------

------------------------------------------------------------

------------------------------------------------------------

------------------------------------------------------------

------------------------------------------------------------

------------------------------------------------------------

------------------------------------------------------------

------------------------------------------------------------

------------------------------------------------------------

------------------------------------------------------------

---------------------------------------------------------TTC

AGGAAGTCCAATCAGTACCTTGTGCATCAACCTTGTGTAGTAGGTTCTGTGGGTCTGCTG

GTTGCAGATTATATGGATGTTACAGACTTCATCCTGGAAAAATTTGCTACTGCCTTCATT

GTAGCAGAGCTGAGTCTCCATTGGCACTTTCTGGAAGCGCTAGGAATGTGAACGACAAGA

ACAACGAGATGGACAACTCTCCAGT-----------------------------------

------------------------------------------------------------

------------------------------------------------------------

------------------------------------------------------------

------------------------------------------------------------

------------------------------------------------------------

------------------------------------------------------------

------------------------------------------------------------

---------------------------------GATGAATGAGGTGGAAAATTTGGACCA

AGAAATGGAAATGTTCTAG

>21_07cDNA

ATGAAGGCAACGATCTTGTTAGCTGTTGTAGTGGCAGTCATTGTTGGAG-----------

------------------------------------------------------------

------------------------------------------------------------

------------------------------------------------------------

------------------------------------------------------------

------------------------------------------------------------

------------------------------------------------------------

------------------------------------------------------------

------------------------------------------------------------

------------------------------------------------------------

------------------------------------------------------------

---------------------------------------------------------TTC

AGGAAGTCCAATCAGTACCTTGTGCATCAACCTTGTGTAGTAGGTTCTGTGGGTCTGCTG

GTTGCAGATTATATGGATGTTACAGACTTCATCCCGGCAAAATTTGCTACTGCCTTCATT

GTCGCAGAGCTGAGTCTCCATTGGCACTTTCTGGAAGCGCTAGGAATGTGAACGATCAGA

ACAAAGAGATGGACAACTCTCCAAT-----------------------------------

------------------------------------------------------------

------------------------------------------------------------

------------------------------------------------------------

------------------------------------------------------------

------------------------------------------------------------

------------------------------------------------------------

------------------------------------------------------------

---------------------------------GATGAATGAGATGGAACATTTGGACCA

AGAAATGGATATGTTCTAG

>21_08cDNA

ATGAAGGCAACGATCTTGTTAGCTGTTGTAGTGGCAGTCATTGTTGGAG-----------

------------------------------------------------------------

------------------------------------------------------------

------------------------------------------------------------

------------------------------------------------------------

------------------------------------------------------------

------------------------------------------------------------

------------------------------------------------------------

------------------------------------------------------------

------------------------------------------------------------

------------------------------------------------------------

---------------------------------------------------------TTC

AGGAAGTCCAATCAGTACCTTGTGCATCAACCTTGTGTAGTAGGTTCTGTGGGTCTGCTG

GTTGCAGATTATATGGATGTTACAGACTTCATCCCGGCAAAATTTGCTACTGCCTTCATT

GTCGCAGAGCTGAGTCTCCATTGGCACTTTCTGGAAGCGCTAGGAATGTGAACGATCAGA

ACAAAGAGATGGGCAACTCTCCAAT-----------------------------------

------------------------------------------------------------

------------------------------------------------------------

------------------------------------------------------------

------------------------------------------------------------

------------------------------------------------------------

------------------------------------------------------------

------------------------------------------------------------

---------------------------------GATGAATGAGATGGAACATTTGGACCA

AGAAATGGATATGTTCTAG

>21_09cDNA

ATGAAGGCAACGATCTTGTTAGCTGTTGTAGTGGCAGTCATTGTTGGAG-----------

------------------------------------------------------------

------------------------------------------------------------

------------------------------------------------------------

------------------------------------------------------------

------------------------------------------------------------

------------------------------------------------------------

------------------------------------------------------------

------------------------------------------------------------

------------------------------------------------------------

------------------------------------------------------------

---------------------------------------------------------TTC

AgGAAGCCCAATCAGTAGCTTGTAGATCATACTACTGTAGTAAGTTCTGTGGGTCTGCTG

GTTGCTCATTATATGGATGTTACCTACTTCATCCtGGAAAAATTTGCTACTGCCTTCATT

GTAgCAgAGCTGAGTCTCCATTGGCACTTTCTGGAAGCGCTAGGAATGTGAACGACAAGA

ACAACGAGATGGACAACTCTCCAGT-----------------------------------

------------------------------------------------------------

------------------------------------------------------------

------------------------------------------------------------

------------------------------------------------------------

------------------------------------------------------------

------------------------------------------------------------

------------------------------------------------------------

---------------------------------GATGAATGAGGTGGAAAATTTGGACCA

AGAAATGGAAATGTTCTAG

>21_10cDNA

ATGAAGGCAACGATCTTGTTAGCTGTTGTAGTGGCAGTCATTGTTGGAG-----------

------------------------------------------------------------

------------------------------------------------------------

------------------------------------------------------------

------------------------------------------------------------

------------------------------------------------------------

------------------------------------------------------------

------------------------------------------------------------

------------------------------------------------------------

------------------------------------------------------------

------------------------------------------------------------

---------------------------------------------------------TTC

AGGAAGTCCAATCAGTACCTTGTGCATCAACCTTGTGTAGTAGGTTCTGTGGGTCTGCTG

GTTGCAGATTATATGGATGTTACAGACTTCATCCCGGCAAAATTTGCTACTGCCTTCATT

GTCGCAGAGCTGAGTCTCCATTGGCACTTTCTGGAAGCGCTAGGAATGTGAACGATCAGA

ACAAAGAGATGGACAACTCTCCAAT-----------------------------------

------------------------------------------------------------

------------------------------------------------------------

------------------------------------------------------------

------------------------------------------------------------

------------------------------------------------------------

------------------------------------------------------------

------------------------------------------------------------

---------------------------------GATGAATGAGATGGAACATTTGGACCA

AGAAATGGATATGTTCTAG

>31_02Gen

ATGAAGGCAACGATCGTGTTAGCTGTTGTAGTGGCAGTCATTGTTGGGGGTAAATATCTT

TATTGAAATATATCTGTGTGTACGTATTTTAGAAGTATAGATCCAGCATTTTT-AAGATA

AAAATACGGCAGACCGACTTCTCCAAAATTTGATTGCGAATCACATATTGTAAATTTATG

TATTATGTTAAA--TTATACTACTTTATTGTAATAATTTTTCATTTA---TAATACCTGT

TTTAAGTAAGAAAGCTGTATTTGGCAAAACTTTTAGGAATTTTGGTCCTCAATGCTCTTC

TTTTTT-AACTTTTATGATTCGAGCGACACTGATGAGTCTTTTGTAGACGAAACGCGCGT

CTGGCGTATACGTAATTTGGTTCTGGTATCTATGATGAGTTTATTGTTAATGTTTACAAC

TGAAATATGTTTTGTTATTAAAATTAAATCACTCTAATACTCAAACAAATATTTTTT-CT

TTATACGTGTTGTTCAGTTCGTAGTTTTCTATTCTATGCTTTGTATACTGTTGTTTTTCT

TTTTTT--CTCTTTTTTTTT---GCCATGGCGTTGTCAGTTTATTTTCAACTTATGATAA

TAAATGTCCC-TGTGGTATATTT--AGCCTCTCTCTAAGACAGGCATCG---ATTTGTGT

CGAAA-CTTAAGCAATTTTATTC-A-TTTTATCTTT-CTAC------TTATTCA--GTTC

AGGAAGCCCAATCAATTCCTTGTACATCATACTACTGTAGTAAGTTCTGTGGGTCTGCTG

GTTGCTCATTATATGGATGTTACCAACTTCATCCCGGCAAAATTTGCTACTGCCTTCATT

ATCGCAGAGCTGAGTCTCCATTGGCACTTTCTGGAAGCGCTAGGAATGTGAACGACAAGA

ACAAAGAGATGGACAACTCTCCAGTGTAAGTACTCAGTACTAGATTTCGTTACACAATAT

AAACATTTAAAAATTT-GAAAAAACA------GTTTTCTTCATGCCAACATATATTGTAA

TTAAGGTGTTTACCAATTTTTGAAACAGATAATTTT---TTTTTTTTAAATAAAATATTT

CATCTT-----AATTCAATATGTCGTTATCGTTTGAATCGTATTACATGTATGCAGTCAT

TGAAAAAAAA--TAAACACCTGAACTACTCCTGCAAGAGAAAGAAGGGAT-GT-TTT-TT

TTTTAGATCTGTTAATGTGTTGGGAGGCAGATAAGATATCTTCTAGTGTGTCTCTCAACT

-ATTTACATTCAAAAACATTTTTTTTTTTACATTAATCGATCACTGGTCAATTGCACGAG

AGTAGTCAGAAAAAAAACCCTGTTATATGAAACGTTTTAATTGAATTTGTGAAAATAA-C

ACCACATATAAGAAAACATCTACTTTGTTTCAGGATGAATGAGATGGAAAATTTGGACCA

AGAAATGGATATGTTCTAG

>31_03Gen

ATGAAGGCAACGATCGTGTTAGCTGTTGTAGTGGCAGTCATTGTTGGGGGTAAATATCTT

TATTGAAATATATCTGTGTGTACGTATTTTAGAAGTATAGATCCAGCATTTTT-AAGATA

AGAATACGGCAGACCGACTTCTCCAAAATTTGATTGCGAATCACATATTGTAAATTTATG

TATTATGTTAAA--TTATACTACTTTATTGTAATAATTTTTCATTTA---TAATAACTGT

TTTAAGTAAGAAAGCTGTATTTGGCAAAACTTTTAGGAATTTTGGTCCTCAATGCTCTTC

TTTTTT-AACTTTTATGATTCGAGCGACACTGATGAGTCTTTTGTAGACGAAACGCGCGT

CTGGCGTATACGTAATTTAGTTCTGGTATCTATGATGAGTTTATTGTTAATGTTTACAAC

TGAAATATGTTTTGTTATTAAAATTAAATCACTCTAATACTCAAACAAATATTTTTT-CT

TTATACGTGTTGTTCAGTTCGTAGTTTTCTATTCTATGCTTTGTATACTGTTGTTTTTCT

TTTTTT--CTCTTTTTTTTT---GCCATGGCGTTGTCAGTTTATTTTCAACTTATGATAA

TAAATGTCCC-TGTGGTATATTT--AGCCTCTCTCTAAGACAGGCATCG---ATTTGTGT

CGAAA-CTTAAGCAATTTTATTC-A-TTTTATTTTT-CTAC------TTATTCA--GTTC

AGGAAGCCCAATCAATTCCTTGTACATCATACTACTGTAGTAAGTTCTGTGGGTCTGCTG

GTTGCTCATTATATGGATGTTACAAACTTCATCCCGGCAAAATTTGCTACTGCCTTCATT

GTCGCAGAGCTGAGTCTCCATTGGCACTTTCTGGAAGCGCTAGGAATGTGAACGAGCAGA

ACAAAGAGATGGACAACTCTCCAGTGTAAGTACTCAGTACTAGATTTCGTTACACAATAT

AAACATTTAAAAATTT-GAAAAAACA------GTTTTCTTCATGCCAACATATATTGTAA

TTAAGGTGTTTACCAATTTTTGAAACAGATAATTTT---TTTTTTTTAAATAAAATATTT

CATCTT-----AATTCAATATGTCGTTATCGTTTGAATCGTATTACATGTATGCAGTCAT

TGAAAAAAACA-TAAACACCTGAACTACTCCTGCAAGAGAAAGAAGGGAT-GT-TTT-TT

TTTTAGATCTGTTAATGTGTTGGGAGGCAGATAAGATATCTTCTAGTGTGTCTCTCAACT

-ATTTACATTCAAAAACATTTTTTTTTTTACATTAATCGATCACTGGTCAATTGCACGAG

AGTAGTCAGAAAAAAAACCCTGTTATATGAAACGTTTTAATTGAATTTGTGAAAATAA-C

ACCACATATAAGAAAACATCTACTTTGTTTCAGGATGAATGAGATGGAAAATTTGGACCA

AGAAATGGATATGTTCTAG

>31_04Gen

ATGAAGGCAACGATCGTGTTAGCTGTTGTAGTGGCAGTCATTGTTGGGGGTAAATATCTT

TATTGAAATATATCTGTGTGTACGTATTTTAGAAGTATAGATCCAGCATTTTT-AAGATA

AAAATACGGCAGACCGACTTCTCCAGAATTTGATTGCGAATCACATATTGTAAATTTATG

TATTATGTTAAA--TTATACTACTTTATTGTAATAATTTTTCATTTA---TAATAACTGT

TTTAAGTAAGAAAGCTGTATTTGGCAAAACTTTTAGGAATTTTGGTCCTCAATGCTCTTC

TTTTTT-AACTTTTATGATTCGAGCGACACTGATGAGTCTTTTGTAGACGAAACGCGCGT

CTGGCGTATACGTAATTTAGTTCTGGTATCTATGATGAGTTTATTGTTAATGTTTACAAC

TGAAATATGTTTTGTTATTAAAATTAAATCACTCTAATACTCAAACAAATATTTTTT-CT

TTATACGTGTTGTTCAGTTCGTAGTTTTCTATTCTATGCTTTGTATACTGTTGTTTTTCT

TTTTTT--CTCTTTTTTTTT---GCCATGGCGTTGTCAGTTTATTTTCAACTTATGATAA

TAAATGTCCC-TGTGGTATATTT--AGCCTCTCTCTAAGACAGGCATCG---ATTTGTGT

CGAAA-CTTAAGCAATTTTATTC-A-TTTTATTTTT-CTAC------TTATTCA--GTTC

AGGAAGCCCAATCAATTCCTTGTACATCATACTACTGTAGTAAGTTCTGTGGGTCTGCTG

GTTGCTCATTATATGGATGTTACAAACTTCATCCCGGCGAAATTCGCTACTGCCTTCATT

GTCGCAGAGCTGAGTCTCCATTGGCACTTTCTGGAAGCGCTAGGAATGTGAACGAGCAGA

ACAAAGAGATGGACAACTCTCCAGTGTAAGTACTCAGTACTAGATTTCGTTACACAATAT

AAACATTTAAAAATTT-GAAAAAACA------GTTTTCTTCATGCCAACATATATTGTAA

TTAAGGTGTTTACCAATTTTTGAAACAGATAATTTT---TTTTTTTTAAATAAAATATTT

CATTTT-----AATTCAATATGTCGTTATCGTTTGAATCGTATTACATGTATGCAGTCAT

TGAAAAAAAAA-TAAACACCTGAACTACTCCTGCAAGAGAAAGAAAGGAT-GT-TTT-TT

TTTTAGATCTGTTAATGTGTTGGGAGGCAGATAAGATATTTTTTAGTGTGTCTTTCAACT

-ATTTACATTCAAAAACATTTTTTTTTTTACATTAATCGATCACTGGTCAATTGCACGAG

AGTAGTCAGAAAAAAAACCCTGTTATATGAAACGTTTTAATTGAATTTGTGAAAATAA-C

ACCACATATAAGAAAACATCTACTTTGTTTCAGGATGAATGAGATGGAAAATTTGGACCA

AGAAATGGATATGTTCTAG

>31_05Gen

ATGAAGGCAACGATCGTGTTAGCTGTTGTAGTGGCAGTCATTGTTGGGGGTAAATATCTT

TATTGAAATATATCTGTGTGTACGTATTTTAGAAGTATAGATCCAGCATTTTT-AAGATA

AAAATACGGCAGACCGACTTCTCCAAAATTTGATTGCGAATCACATATTGTAAATTTATG

TATTATGTTAAA--TTATACTACTTTATTGTAATAATTTTTCATTTA---TAATAACTGT

TTTAAGTAAGAAAGCTGTATTTGGCAAAACTTTTAGGAATTTTGGTCCTCAATGCTCTTC

TTTTTT-AACTTTTATGATTCGAGCGACACTGATGAGTCTTTTGTAGACGAAACGCGCGT

CTGGCGTATACGTAATTTAGTTCTGGTATCTATGATGAGTTTATTGTTAATGTTTACAAC

TGAAATATGTTTTGTTATTAAAATTAAATCACTCTAATACTCAAACAAATATTTTTT-CT

TTATACGTGTTGTTCAGTTCGTAGTTTTCTATTCTATGCTTTGTATACTGTTGTTTTTCT

TTTTTT--CTCTTTTTTTTT---GCCATGGCGTTGTCAGTTTATTTTCAACTTATGATAA

TAAATGTCCC-TGTGGTATATTT--AGCCTCTCTCTAAGACAGGCATCG---ATTTGTGT

CGAAA-CTTAAGCAATTTTATTC-A-TTTTATTTTT-CTAC------TTATTCA--GTTC

AGGAAGCCCAATCAATTCCTTGTACATCATACTACTGTAGTAAGTTCTGTGGGTCTGCTG

GTTGCTCATTATATGGATGTTACAAACTTCATCCCGGCAAAATTTGCTACTGCCTTCATT

GTCGCAGAGCTGAGTCTCCATTGGCACTTTCTGGAAGCGCTAGGAATGTGAACGAGCAGA

ACAAAGAGATGGACAACTCTCCAGTGTAAGTACTCAGTACTAGATTTCGTTACACAATAT

AAACATTTAAAAATTT-GAAAAAACA------GTTTTCTTCATGCCAACATATATTGTAA

TTAAGGTGTTTACCAATTTTTGAAACAGATAATTTT---TTTTTTTTAAATAAAATATTT

CATCTT-----AATTCAATATGTCGTTATCGTTTGAATCGTATTACATGTATGCAGTCAT

TGAAAAAAACA-TAAACACCTGAACTACTCCTGCAAGAGAAAGAAGGGAT-GT-TTT-TT

TTTTAGATCTGTTAATGTGTTGGGAGGCAGATAAGATATCTTCTAGTGTGTCTCTCAACT

-ATTTACATTCAAAAACATTTTTTTTTTTACATTAATCGATCACTGGTCAATTGCACGAG

AGTAGTCAGAAAAAAAACCCTGTTATATGAAACGTTTTAATTGAATTTGTGAAAATAA-C

ACCACATATAAGAAAACATCTACTTTGTTTCAGGATGAATGAGATGGAAAATTTGGACCA

AGAAATGGATATGTTCTAG

>31_06Gen

ATGAAGGCAACGATCGTGTTAGCTGTTGTAGTGGCAGTCATTGTTGGGGGTAAATATCTT

TATTGAAATATATCTGTGTGTACGTATTTTAGAAGTATAGATCCAGCATTTTT-AAGATA

AAAATACGGCAGACCGACTTCTCCAAAATTTGATTGCGAATCACATATTGTAAATTTATG

TATTATGTTAAA--TTATACTACTTTATTGTAATAATTTTTCATTTA---TAATAACTGT

TTTAAGTAAGAAAGCTGTATTTGGCAAAACTTTTAGGAATTTTGGTCCTCAACGCTCTTC

TTTTTT-AACTTTTATGATTCGAGCGACACTGATGAGTCTTTTGTAGACGAAACGCGCGT

CTGGCGTATACGTAATTTAGTTCTGGTATCTATGATGAGTTTATTGTTAATGTTTACAAC

TGAAATATGTTTTGTTATTAAAATTAAATCACTCTAATACTCAAACAAATATTTTTT-CT

TTATACGTGTTGTTCAGTTCGTAGTTTTCTATTCTATGCTTTGTATACTGTTGTTTTTCT

TTTTTT--CTCTTTTTTTTT---GCCATGGCGTTGTCAGTTTATTTTCAACTTATGATAA

TAAATGTCCC-TGTGGTATATTT--AGCCTCTCTCTAAGACAGGCATCG---ATTTGTGT

CGAAA-CTTAAGCAATTTTATTC-A-TTTTATTTTT-CTAC------TTATTCA--GTTC

AGGAAGCCCAATCAATTCCTTGTACATCATACTACTGTAGTAAGTTCTGTGGGTCTGCTG

GTTGCTCATTATATGGATGTTACAAACTTCATCCCGGCAAAATTTGCTACTGCCTTCATT

GTCGCAGAGCTGAGTCTCCATTGGCACTTTCTGGAAGCGCTAGGAATGTGAACGTGCAGA

ACAAAGAGATGGACAACTCTCCAGTGTAAGTACTCAGTACTAGATTTCGTTACACAATAT

AAACATTTAAAAATTT-GAAAAAACA------GTTTTCTTCATGCCAACATATATTGTAA

TTAAGGTGTTTACCAATTTTTGAAACAGATAATTTT---TTTTTTTTAAATAAAATATTT

CATCTT-----AATTCAATATGTCGTTATCGTTTGAATCGTATTACATGTATGCAGTCAT

TGAAAAAAACA-TAAACACCTGAACTACTCCTGCAAGAGAAAGAAGGGAT-GT-TTT-TT

TTTTAGATCTGTTAATGTGTTGGGAGGCAGATAAGATATCTTCTAGTGTGTCTCTCAACT

-ATTTACATTCAAAAACATTTTTTTTTTTACATTAATCGATCACTGGTCAATTGCACGAG

AGTAGTCAGAAAAAAAACCCTGTTATATGAAACGTTTTAATTGAATTTGTGAAAATAA-C

ACCACATATAAGAAAACATCTACTTTGTTTCAGGATGAATGAGATGGAAAATTTGGACCA

AGAAATGGATATGTTCTAG

>31_09Gen

ATGAAGGCAACGATCGTGTTAGCTGTTGTAGTGGCAGTCATTGTTGGGGGTAAATATCTT

TATTGAAATATATCTGTGTGTACGTATTTTAGAAGTATAGATCCAGCATTTTT-AAGATA

AAAATACGGCAGACCGACTTCTCCAAAATTTGATTGCGAATCACATATTGTAAATTTATG

TATTATGTTAAA--TTATACTACTTTATTGTAATAATTTTTCATTTA---TAATAACTGT

TTTAAGTAAGAAAGCTGTATTTGGCAAAACTTTTAGGAATTTTGGTCCTCAATGCTCTTC

TTTTTT-AACTTTTATGATTCGAGCGACACTGATGAGTCTTTTGTAGACGAAACGCGCGT

CTGGCGTATACGTAATTTAGTTCTGGTATCTATGATGAGTTTATTGTTAATGTTTACAAC

TGAAATATGTTTTGTTATTAAAATTAAATCACTCTAATACTCAAACAAATATTTTTT-CT

TTATACGTGTTGTTCAGTTCGTAGTTTTCTATTCTATGCTTTGTATACTGTTGTTTTTCT

TTTTTT--CTCTTTTTTTTT---GCCATGGCGTTGTCAGTTTATTTTCAACTTATGATAA

TAAATGTCCC-TGTGGTATATTT--AGCCTCTCTCTAAGACAGGCATCG---ATTTGTGT

CGAAA-CTTAAGCAATTTTATTC-A-TTTTATTTTT-CTAC------TTATTCA--GTTC

AGGAAGCCCAATCAATTCCTTGTACATCATACTACTGTAGTAAGTTCTGTGGGTCTGCTG

GTTGCTCATTATATGGATGTTACAAACTTCATCCCGGCAAAATTTGCTACTGCCTTCATT

GTCGCAGAGCTGAGTCTCCATTGGCACTTTCTGGAAGCGCTAGGAATGTGAACGAGCAGA

ACAAAGAGATGGACAACTCTCCAGTGTAAGTACTCAGTACTAGATTTCGTTACACAATAT

AAACATTTAAAAATTT-GAAAAAACA------GTTTTCTTCATGCCAACATATATTGTAA

TTAAGGTGTTTACCAATTTTTGAAACAGATAATTTT---TTTTTTTTTAATAAAATATTT

CATTTT-----AATTCAATATGTCGTTATCGTTTGAATCGTATTACATGTATGCAGTCAT

TGAAAAAAACA-TAAACACCTGAACTACTCCTGCAAGAGAAAGAAGGGAT-GT-TTT-TT

TTTTAGATCTGTTAATGTGTTGGGAGGCAGATAAGATATTTTTTAGTGTGTCTTTCAACT

-ATTTACATTCAAAAACATTTTTTTTTTTACATTAATCGATCACTGGTCAATTGCACGAG

AGTAGTCAGAAAAAAAACCCTGTTATATGAAACGTTTTAATTGAATTTGTGAAAATAA-C

ACCACATATAAGAAAACATCTACTTTGTTTTAGGATGAATGAGATGGAAAATTTGGACCA

AGAAATGGATATGTTCTAG

>31_11Gen

ATGAAGGCAACGATCGTGTTAGCTGTTGTAGTGGCAGTCATTGTTGGGGGTAAATATCTT

TATTGAAATATATCTGTGTGTACGTATTTTAGAAGTATAGATCCAGCATTTTT-AAGATA

AAAATACGGCAGACCGACTTCTCCAAAATTTGATTGCGAATCACATATTGTAAATTTATG

TATTATGTTAAA--TTATACTACTTTATTGTAATAATTTTTCATTTA---TAATAACTGT

TTTAAGTAAGAAAGCTGTATTTGGCAAAACTTTTAGGAATTTTGGTCCTCAATGCTCTTC

TTTTTT-AACTTTTATGATTCGAGCGACACTGATGAGTCTTTTGTAGACGAAACGCGCGT

CTGGCGTATACGTAATTTAGTTCTGGTATCTATGATGAGTTTATTGTTAATGTTTACAAC

TGAAATATGTTTTGTTATTAAAATTAAATCACTCTAATACTCAAACAAATATTTTTT-CT

TTATACGTGTTGTTCAGTTCGTAGTTTTCTATTCTATGCTTTGTATACTGTTGTTTTTCT

TTTTTT--CTCTTTTTTTTT---GCCATGGCGTTGTCAGTTTATTTTCAACTTATGATAA

TAAATGTCCC-TGTGGTATATTT--AGCCTCTCTCTAAGACAGGCGTCG---ATTTGTGT

CGAAA-CTTAAGCAATTTTATTC-A-TTTTATTTTT-CTAC------TTATTCA--GTTC

AGGAAGCCCAATCAATTCCTTGTACATCATACTACTGTAGTAAGTTCTGTGGGTCTGCTG

GTTGCTCATTATATGGATGTTACAAACTTCATCCCGGCAAAATTTGCTACTGCCTTCATT

GTCGCAGAGCTGAGTCTCCATTGGCACTTTCTGGAAGCGCTAGGAATGTGAACGAGCAGA

ACAAAGAGATGGACAACTCTCCAGTGTAAGTACTCAGTACTAGATTTCGTTACACAATAT

AAACATTTAAAAATTT-GAAAAAACA------GTTTTCTTCATGCCAACATATATTGTAA

TTAAGGTGTTTACCAATTTTTGAAACAGATAATTTT-T-TTTTTTTTAAATAAAATATTT

CATCTT-----AATTCAATATGTCGTTATCGTTTGAATCGTATTACATGTATGCAGTCAT

TGAAAAAAACA-TAAACACCTGAACTACTCCTGCAAGAGAAAGAAGGGAT-GT-TTTTTT

CTT-AGATCTGTTAATGTGTTGGGAGGCAGATAAGATATCTTCTAGTGTGTCTCTCAACT

-ATTTGCATTCAAAAACATTTTTTTTTTTACATTAATCGATCACTGGTCAATTGCACGAG

AGTAGTCAGAAAAAAAACCCTGTTATATGAAACGTTTTAATTGAATTTGTGAAAATAA-C

ACCACATATAAGAAAACATCTACTTTGTTTCAGGATGAATGAGATGGAAAATTTGGACCA

AGAAATGGATATGTTCTAG

>31_66Gen

ATGAAGGCAACGATCGTGTTAGCTGTTGTAGTGGCAGTCATTGTTGGGGGTAAATATCTT

TATTGAAATATATCTGTGTGTACGTATTTTAGAAGTATAGATCCAGCATTTTT-AAGATA

AAAATACGGCAGACCGACTTCTCCAAAATTTGATTGCGAATCACATATTGTAAATTTATG

TATTATGTTAAA--TTATACTACTTTATTGTAATAATTTTTCATTTA---TAATAACTGT

TTTAAGTAAGAAAGCTGTATTTGGCAAAACTTTTAGGAATTTTGGTCCTCAATGCTCTTC

TTTTTT-AACTTTTATGATTCGAGCGACACTGATGAGTCTTTTGTAGACGAAACGCGCGT

CTGGCGTATACGTAATTTAGTTCTGGTATCTATGATGAGTTTATTGTTAATGTTTACAAC

TGAAATATGTTTTGTTATTAAAATTAAATCACTCTAATACTCAAACAAATATTTTTT-CT

TTATACGTGTTGTTCAGTTCGTAGTTTTCTATTCTATGCTTTGTATACTGTTGTTTTTCT

TTTTTT--CTCTTTTTTTTTT--GCCATGGCGTTGTCAGTTTATTTTCAACTTATGATAA

TAAATGTCCC-TGTGGTATATTT--AGCCTCTCTCTAAGACAGGCATCG---ATTTGTGT

CGAAA-CTTAAGCAATTTTATTC-A-TTTTATTTTT-CTAC------TTATTCA--GTTC

AGGAAGCCCAATCAATTCCTTGTACATCATACTACTGTAGTAAGTTCTGTGGGTCTGCTG

GTTGCTCATTATATGGATGTTACAAACTTCATCCCGGCAAAATTTGCTACTGCCTTCATT

GTCGCAGAGCTGAGTCTCCATTGGCACTTTCTGGAAGCGCTAGGAATGTGAACGAGCAGA

ACAAAGAGATGGACAACTCTCCAGTGTAAGTACTCAGTACTAGATTTCGTTACACAATAT

AAACATTTAAAAATTT-GAAAAAACA------GTTTTCTTCATGCCAACATATATTGTAA

TTAAGGTGTTTACCAATTTTTGAAACAGATAATTT--T-TTTTTTTTAAATAAAATATTT

CATCTT-----AATTCAATATGTCGTTATCGTTTGAATCGTATTACACGTATGCAGTCAT

TGAAAAAAACA-TAAACACCTGAACTACTCCTGCAAGAGAAAGAAGGGAT-GT-TTTTTT

CTT-AGATCTGTTAATGTGTTGGGAGGCAGATAAGATATCTTCTAGTGTGTCTCTCAACT

-ATTTACATTCAAAAACATTTTTTTTTTTACATTAATCGATCACTGGTCAATTGCACGAG

AGTAGTCAGAAAAAAAACCCTGTTATATGAAACGTTTTAATTGAATTTGTGAAAATAA-C

ACCACATATAAGAAAACATCTACTTTGTTTCAGGATGAATGAGATGGAAAATTTGGACCA

AGAAATGGATATGTTCTAG

>31_68Gen

ATGAAGGCAACGATCTTGTTAGCTGTTGTAGTGGCAGTCATTGTTGGAGGTAAATATCTT

TATTGAAATATATCTGTGTGTACGTATTTTAGAAGTATAGATCCAGCATTTTT-AGGATA

AATATACGGCAGACCGACTTTTCTGAAAA-------------------------------

---T-----------TTTACTACTTTATTGTAATAATTTTTCATTGA---TAATAACTGT

TTTAAGTAAGAAAG----------------------------------------------

------------------------------------------------------------

-------------------------------------------TTGTTAATGTTTACAAC

TGAAGTATGTTTTGTTATTAAAATTAGATCATTCAAATATTCAAACAAATATTTTTT-CT

TTATACGTGTTGTTCAGTTCGTAGTTTTCTATACTATGCTTTGTATACTGTTGTTTTTCT

TTTTTTTTCTCCTTTTTTT----GCCATGGCGTTGTCAGTTTACTTTCAACTTAAGATAA

TAAATGTTCC-TGTGGTATATTT--AGCCTCTTTCTAAGACAGGCATCG---ATTTGTGT

CGAAA-CTTAAGCAATTTTATTC-A-TTCTATTTTA-CTAC------TTATTCA--GTTC

AGGAAGCCCAATCAGTAGCTTGTACATCATACTACTGTAGTAAGTTCTGTGGGTCTGCTG

GTTGCTCATTATATGGATGTTACCTACTTCATCCTGGAAAAATTTGCTACTGCCTTCATT

GTAGCAGAGCTGAGTCTCCATTGGCACTTTCTGGAAGCGCTAGGAATGTGAACGACAAGA

ACAACGAGATGGACAACTCTCCAGTGTAAGTACTCAATACTAGATTTCGTT--ACAATAT

AAAATTTGAAAAAATT-GAAAAAAAA------GTTTTCTACACGCCAACATATATTTTAA

TTAAGGTGTTTACCAATTTTTGAAACAGATAATTTGAT-TTTTTTTTAACTAAAATATTT

CATCTT-----AATTCCATATGTCGTTATTGTTTGAATCGTATTA------TGCAGTCAT

TGAAAAAAA-A---AATACCTGAACTACTCCTGCAAGAGAAGGAAGGGAT-GT-TTTTT-

TTTTATATTGGTTAATGTGGTGGGAGGCAAATAAGATATTTTTTATTGTGTCTCTCAATT

-ATTTACATTCAAAAACATTTTTTT----ACATAAATCGATCACTGGTCAATTGCACGAG

AGTAGTCAGAAAAAAAC---TGTTATATGAAATGTTTTAATTGAATTTGTGAAAATAA-C

ACCACATATAAGAGAACATCTACTTTGTTTCAGGATGAATGAGATGGAACATTTGGACCA

AGAAATGAATATGTTCTAG

>31_69Gen

ATGAAGGCAACGATCTTGTTAGCTGTTGTAGTGGCAGTCATTGTTGGAGGTAAATATCTT

TATTGAAATATATCTGTGTGTACGTATTTTAGAAGTATAGATCCAGCATTTTT-AGGATA

AATATACGGCAGACCGACTTTTCTGAAAA-------------------------------

---T-----------TTTACTACTTTATTGTAATAATTTTTCATTGA---TAATAACTGT

TTTAAGTAAGAAAG----------------------------------------------

------------------------------------------------------------

-------------------------------------------TTGTTAATGTTTACAAC

TGAAGTATGTTTTGTTATTAAAATTAGATCATTCAAATATTCAAACAAATATTTTTT-CT

TTATACGTGTTGTTCAGTTCGTAGTTTTCTATACTATGCTTTGTATACTGTTGTTTTTCT

TTTTTTTTCTCCTTTTTTT----GCCATGGCGTTGTCAGTTTACTTTCAACTTAAGATAA

TAAATGTTCC-TGTGGTATATTT--AGCCTCTTTCTAAGACAGGCATCG---ATTTGTGT

CGAAA-CTTAAGCAATTTTATTC-A-TTCTATTTTA-CTAC------TTATTCA--GTTC

AGGAAGCCCAATCAGTAGCTTGTACATCATACTACTGTAGTAAGTTCTGTGGGTCTGCTG

GTTGCTCATTATATGGATGTTACCTACTTCATCCTGGAAAAATTTGCTACTGCCTTCATT

GTAGCAGAGCTGAGTCTCCATTGGCACTTTCTGGAAGCGCTAGGAATGTGAACGACAAGA

ACAACGAGATGGACAACTCTCCAGTGTAAGTACTCAATACTAGATTTCGTTACACAATAT

AAAATTT----------GAAAAAAAA------GTTTTCTACATGCCAACATATATTTTAA

TTAAGGTGTTTACCAATTTTTGAAACAGATAATTTGAT-TTTTTTTTAACTAAAATATTT

CATCTT-----AATTCCATATGTCGTTATTGTTTGAATCGTATTA------TGCAGTCAT

TGAAAAAAA-A---AATACCTGAACTACTCCTGCAAGAGAAGGAAGGGAT-GT-TTTTT-

TTTTATATTGGTTAATGTGGTGGGAGGCAAATAAGATATTTTTTATTGTGTCTCTCAATT

-ATTTACATTCAAAAACATTTTTTT----ACATAAATCGATCACTGGTCAATTGCACGAG

AGTAGTCAGAAAAAAAC---TGTTATATGAAACGTTTTAATTGAATTTGTGAAAATAA-C

ACCACATATAAGAGAACATCTACTTTGTTTCAGGATGAATGAGATGGAACATTTGGACCA

AGAAATGAATATGTTCTAG

>31_01cDNA

ATGAAGGCAACGATCGTGTTAGCTGTTGTAGTGGCAGTCATTGTTGGGG-----------

------------------------------------------------------------

------------------------------------------------------------

------------------------------------------------------------

------------------------------------------------------------

------------------------------------------------------------

------------------------------------------------------------

------------------------------------------------------------

------------------------------------------------------------

------------------------------------------------------------

------------------------------------------------------------

---------------------------------------------------------TTC

AGGAAGCCCAATCAATTCCTTGTACATCATACTACTGTAGTAAGTTCTGTGGGTCTGCTG

GTTGCTCATTATATGGATGTTACAAACTTCATCCCGGCAAAATTTGCTACTGCCTTCATT

GTCGCAGAGCTGAGTCTCCATTGGCACTTTCTGGAAGCGCTAGGAATGTGAACGAGCAGA

ACAAAGAGATGGACAACTCTCCAGT-----------------------------------

------------------------------------------------------------

------------------------------------------------------------

------------------------------------------------------------

------------------------------------------------------------

------------------------------------------------------------

------------------------------------------------------------

------------------------------------------------------------

---------------------------------GATGAATGAGATGGAAAATTTGGACCA

AGAAATGGATATGTTCTAG

>31_02cDNA

ATGAAGGCAACGATCTTGTTAGCTGTTGTAGTGGCAGTCATTGTTGGAG-----------

------------------------------------------------------------

------------------------------------------------------------

------------------------------------------------------------

------------------------------------------------------------

------------------------------------------------------------

------------------------------------------------------------

------------------------------------------------------------

------------------------------------------------------------

------------------------------------------------------------

------------------------------------------------------------

---------------------------------------------------------TTC

AGGAAGCCCAATCAGTAGCTTGTACATCATACTACTGTAGTAAGTTCTGTGGGTCTGCTG

GTTGCTCATTATATGGATGTTACCTACTTCATCCTGGAAAAATTTGCTACTGCCTTCATT

GTAGCAGAGCTGAGTCTCCATTGGCACTTTCTGGAAGCGCTAGGAATGTGAACGACAAGA

ACAACGAGATGGACAACTCTCCAGT-----------------------------------

------------------------------------------------------------

------------------------------------------------------------

------------------------------------------------------------

------------------------------------------------------------

------------------------------------------------------------

------------------------------------------------------------

------------------------------------------------------------

---------------------------------GATGAATGAGATGGAACATTTGGACCA

AGAAATGAATATGTTCTAG

>31_03cDNA

ATGAAGGCAACGATCTTGTTAGCTGTTGTAGTGGCAGTCATTGTTGGAG-----------

------------------------------------------------------------

------------------------------------------------------------

------------------------------------------------------------

------------------------------------------------------------

------------------------------------------------------------

------------------------------------------------------------

------------------------------------------------------------

------------------------------------------------------------

------------------------------------------------------------

------------------------------------------------------------

---------------------------------------------------------TTC

AGGAAGCCCAATCAGTAGCTTGTACATCATACTACTGTAGTAAGTTCTGTGGGTCTGCTG

GTTGCTCATTATATGGATGTTACCTACTTCATCCTGGAAAAATTTGCTACTGCCTTCATT

GTAGCAGAGCTGAGTCTCCATTGGCACTTTCTGGAAGCGCTAGGAGTGTGAACGACAAGA

ACAACGAGATGGACAACTCTCCAGT-----------------------------------

------------------------------------------------------------

------------------------------------------------------------

------------------------------------------------------------

------------------------------------------------------------

------------------------------------------------------------

------------------------------------------------------------

------------------------------------------------------------

---------------------------------GATGAATGAGATGGAACATTTGGACCA

AGAAATGAATATGTTCTAG

>31_04cDNA

ATGAAGGCAACGATCGTGTTAGCTGTTGTAGTGGCAGTCATTGTTGGGG-----------

------------------------------------------------------------

------------------------------------------------------------

------------------------------------------------------------

------------------------------------------------------------

------------------------------------------------------------

------------------------------------------------------------

------------------------------------------------------------

------------------------------------------------------------

------------------------------------------------------------

------------------------------------------------------------

---------------------------------------------------------TTC

AGGAAGCCCAATCAATTCCTTGTACATCATACTACTGTAGTAAGTTCTGTGGGTCTGCTG

GTTGCTCATTATATGGATGTTACAAACTTCATCCCGGCAAAATTTGCTACTGCCTTCATT

GTCGCAGAGCTGAGTCTCCATTGGCACCTTCTGGAAGCGCTAGGAATGTGAACGAGCAGA

ACAAAGAGATGGACAACTCTCCAGT-----------------------------------

------------------------------------------------------------

------------------------------------------------------------

------------------------------------------------------------

------------------------------------------------------------

------------------------------------------------------------

------------------------------------------------------------

------------------------------------------------------------

---------------------------------GATGAATGAGATGGAAAATTTGGACCA

AGAAATGGATATGTTCTAG

>31_05cDNA

ATGAAGGCAACGATCGTGTTAGCTGTTGTAGTGGCAGTCATTGTTGGGG-----------

------------------------------------------------------------

------------------------------------------------------------

------------------------------------------------------------

------------------------------------------------------------

------------------------------------------------------------

------------------------------------------------------------

------------------------------------------------------------

------------------------------------------------------------

------------------------------------------------------------

------------------------------------------------------------

---------------------------------------------------------TTC

AGGAAGCCCAATCAATTCCTTGTACATCATACTACTGTAGTAAGTTCTGTGGGTCTGCTG

GTTGCTCATTATATGGATGTTACAAACTTCATCCCGGCAAAATTTGCTACTGCCTTCATT

GTCGCAGAGCTGAGTCTCCATTGGCACTTTCTGGAAGCGCTAGGAATGTGAACGAGCAGA

ACAAAGAGATGGACAACTCTCCAGT-----------------------------------

------------------------------------------------------------

------------------------------------------------------------

------------------------------------------------------------

------------------------------------------------------------

------------------------------------------------------------

------------------------------------------------------------

------------------------------------------------------------

---------------------------------GATGAATGAGATGGAAAATTTGGACCA

AGAAATGGATATGTTCTAG

>31_06cDNA

ATGAAGGCAACGATCTTGTTAGCTGTTGTAGTGGCAGTCATTGTTGGAG-----------

------------------------------------------------------------

------------------------------------------------------------

------------------------------------------------------------

------------------------------------------------------------

------------------------------------------------------------

------------------------------------------------------------

------------------------------------------------------------

------------------------------------------------------------

------------------------------------------------------------

------------------------------------------------------------

---------------------------------------------------------TTC

AGGAAGCCCAATCAGTAGCTTGTACATCATACTACTGTAGTAAGTTCTGTGGGTCTGCTG

GTTGCTCATTATATGGATGTTACCTACTTCATCCTGGAAAAATTTGCTACTGCCTTCATT

GTAGCAGAGCTGAGTCTCCATTGGCACTTTCTGGAAGCGCTAGGAATGTGAACGACAAGA

ACAACGAGATGGACAACTCTCCAGT-----------------------------------

------------------------------------------------------------

------------------------------------------------------------

------------------------------------------------------------

------------------------------------------------------------

------------------------------------------------------------

------------------------------------------------------------

------------------------------------------------------------

---------------------------------GATGAATGAGATGGAACATTTGGACCA

AGAAATGAATATGTTCTAG

>31_07cDNA

ATGAAGGCAACGATCTTGTTAGCTGTTGTAGTGGCAGTCATTGTTGGAG-----------

------------------------------------------------------------

------------------------------------------------------------

------------------------------------------------------------

------------------------------------------------------------

------------------------------------------------------------

------------------------------------------------------------

------------------------------------------------------------

------------------------------------------------------------

------------------------------------------------------------

------------------------------------------------------------

---------------------------------------------------------TTC

AGGAAGCCCAATCAGTAGCTTGTACATCATACTACTGTAGTAAGTTCTGTGGGTCTGCTG

GTTGCTCATTATATGGATGTTACCTACTTCATCCTGGAAAAATTTGCTACTGCCTTCATT

GTAGCAGAGCTGAGTCTCCATTGGCACTTTCTGGAAGCGCTAGGAATGTGAACGACAAGA

ACAACGAGATGGACAACTCTCCAGT-----------------------------------

------------------------------------------------------------

------------------------------------------------------------

------------------------------------------------------------

------------------------------------------------------------

------------------------------------------------------------

------------------------------------------------------------

------------------------------------------------------------

---------------------------------GATGAATGAGATGGAACATTTGGACCA

AGAAATGAATATGTTCTAG

>31_08cDNA

ATGAAGGCAACGATCTTGTTAGCTGTTGTAGTGGCAGTCATTGTTGGAG-----------

------------------------------------------------------------

------------------------------------------------------------

------------------------------------------------------------

------------------------------------------------------------

------------------------------------------------------------

------------------------------------------------------------

------------------------------------------------------------

------------------------------------------------------------

------------------------------------------------------------

------------------------------------------------------------

---------------------------------------------------------TTC

AGGAAGCCCAATCAGTAGCTTGTACATCATACTACTGTAGTAAGTTCTGTGGGTCTGCTG

GTTGCTCATTATATGGATGTTACCTACTTCATCCTGGAAAAATTTGCTACTGCCTTCATT

GTAGCAGAGCTGAGTCTCCATTGGCACTTTCTGGAAGCGCTAGGAATGTGAACGACAAGA

ACAACGAGATGGACAACTCTCCAGT-----------------------------------

------------------------------------------------------------

------------------------------------------------------------

------------------------------------------------------------

------------------------------------------------------------

------------------------------------------------------------

------------------------------------------------------------

------------------------------------------------------------

---------------------------------GATGAATGAGATGGAACATTTGGACCA

AGAAATGAATATGTTCTAG

>31_09cDNA

ATGAAGGCAACGATCGTGTTAGCTGTTGTAGTGGCAGTCATTGTTGGGG-----------

------------------------------------------------------------

------------------------------------------------------------

------------------------------------------------------------

------------------------------------------------------------

------------------------------------------------------------

------------------------------------------------------------

------------------------------------------------------------

------------------------------------------------------------

------------------------------------------------------------

------------------------------------------------------------

---------------------------------------------------------TTC

AGGAAGCCCAATCAATTCCTTGTACATCATACTACTGTAGTAAGTTCTGTGGGTCTGCTG

GTTGCTCATTATATGGATGTTACAAACTTCATCCCGGCAAAATTTGCTACTGCCTTCATT

GTCGCAGAGCTGAGTCTCCATTGGCACTTTCTGGAAGCGCTAGGAATGTGAACGAGCAGA

ACAAAGAGATGGACAACTCTCCAGT-----------------------------------

------------------------------------------------------------

------------------------------------------------------------

------------------------------------------------------------

------------------------------------------------------------

------------------------------------------------------------

------------------------------------------------------------

------------------------------------------------------------

---------------------------------GATGAATGAGATGGAAAATTTGGACCA

AGAAATGGATATGTTCTAG

>31_10cDNA

ATGAAGGCAACGATCGTGTTAGCTGTTGTAGTGGCAGTCATTGTTGGGG-----------

------------------------------------------------------------

------------------------------------------------------------

------------------------------------------------------------

------------------------------------------------------------

------------------------------------------------------------

------------------------------------------------------------

------------------------------------------------------------

------------------------------------------------------------

------------------------------------------------------------

------------------------------------------------------------

---------------------------------------------------------TTC

AGGAAGCCCAATCAATTCCTTGTACATCATACTACTGTAGTAAGTTCTGTGGGTCTGCTG

GTTGCTCATTATATGGATGTTACAAACTTCATCCCGGCAAAATTTGCTACTGCCTTCATT

GTCGCAGAGCTGAGTCTCCATTGGCACTTTCTGGAAGCGCTAGGAATGTGAACGAGCAGA

ACAAAGAGATGGACAACTCTCCAGT-----------------------------------

------------------------------------------------------------

------------------------------------------------------------

------------------------------------------------------------

------------------------------------------------------------

------------------------------------------------------------

------------------------------------------------------------

------------------------------------------------------------

---------------------------------GATGAATGAGATGGAAAATTTGGACCA

AGAAATGGATATGTTCTAG

>33_01Gen

ATGAAGGCAACGATCTTGTTAGCTGTTGTAGTGGTAGTCATTGTTGGAGGTAAATATCTT

TATTGAAATATATCTGTGTGTATGTGTTTTAGAAGTATAGATCCAGCATTTTT-AAGATA

AAAATACGGCAGACCGACTTTTCCAAAATTTGATTGCGAATCCCATATT---------TG

TATATTATGTTAAATTATACTACTTAATTGTAATAATTTTTCATTTA---TAATAACTGT

TTTAAGTAAGAAAGCTGTATTTGGCAAAACTTTTAGGAATTTTGGTCCTCAATGCTCTTC

TTTTTT-AACTTTTATGATTCGAGCGTCACTGATGAATCTTTTGTAGACGAAACGCGCGG

CTGGCGTATATTTAATTTAGTTCTGGTATCTATGATGAGTTTATTGTTAATGTTTACAAC

TGAAATATGTTTTGTTATTAAAATTAAATCACTCAAATACTCAAACAAATATTTTTT-CT

TTATACGTGTTGTTCAGTTCGTAGTTTTCTATGCTATGCTTTGTATACTGTTGTTTTTCT

TTTTTTTTCTCCTTTTGTTT---GCCATGGCGTTGTTAGTTTATTTTCAACTTATGATAA

TAAATGTCCC-TGTGGTATATTT--AGCCTCTCTCTAAGACAGGCATCGTT-ATTTGTGT

CGAAA-CTTAAGCAATTTTATTC-A-TTTCATTTTT-CTAC------TTATTCA--GTTC

AGGAAGCCCAATCAGTAGCTTGTAGATCATACTACTGTAGTAAGTTCTGTGGGTCTGCTG

GTTGCTCATTATATGGATGTTACCTACTTCATCCTGGAAAAATTTGCTACTGCCTTCATT

GTAGCAGAGCTGAGTCTCCATTGGCACTTTCTGGAAGCGCTAGGAATGTGAACGACAAGA

ACAACGAGATGGACAACTCTCCAGTGTAAGTACTCAATACTAGATTTCGTTACACAGTAT

AAAAATTTAAAAATTTGGAAAAAAAA------GTTTTCTACATTCCAACATATATTGTAA

TTAAGGTGTTTACCAATTTTTGAAACAGATAATTTAAT-TTTTTTTTAAATAAAATATTT

CATCTT-----AATTCAATATGTCGTTATCGTTTGAATCGTATTA------TGCAGTCAT

TGAAAAAAAAA-TAAACACCTGAACTCCTCCTGCAAGAGAAAGAAGGGAT-GT-TTT-TT

TCTAAGATCGGTTAATGTGGTGGGAGGCAGATGAGATATCATCTAGTGTGTCTCTCAACT

-ATTTACATTCAAAAACATTTTTTTTTT-ACATTAATCGATCACTGGTCAATTGCACGAG

AGTAGTCAGAAAAAAA-CCCTGTTATATGAAACGTTTTAATTGAATTTGTGAAAATAA-C

ACCACATATAAGAAAACATCTACTTTGTTTCAGGATGAATGAGATGGACAATTTGGACCA

AGAAATGAATATGTTCTAG

>33_02Gen

ATGAAGGCAACGATCGTGTTAGCTGTTGTAGTGGCAGTCATTGTTGGAGGTAAATATCTT

TATTGAAATATATCTGTGTGTACGTATTTTAGAAGTATAGATCCAGCATTTTT-AGGATA

AATATACGGCAGACCGACTTTTCTGAAA--------------------------------

-------------ATTTTACTTCTTTATT-TATT--TTTTTCATTGA---TAATAACTGC

TTTAAGTAAGAAAG----------------------------------------------

------------------------------------------------------------

-------------------------------------------TTGTTAATGTTTACAAC

TGAAATATGTTTTGTTATTAAAATTAGATCATTCAAATATTCAAACAAATATTTTTT-CT

TTATACGTGTTGTTCAGTTCGTAGTTTTCTATGCTATGCTTTGTATACTGTTGTTTTTCT

TTTTTTT-CTCCTTTTTT-----GCCATGGCGTTGTCAGTTTACTTTCAAGTTAAGATAA

TAAATGTTCC-TGTGGTATATTT--AGCCTCTCTCTAAGACAGGCATCG---ATTTGTGT

CGAAA-CTTAAGCAATTTTATTC-A-TTTTATTTTT-CTAC------TTATTCA--GTTC

AGGAAGCCCAATCAGTAGCTTGTACATCATACTACTGTAGTAAGTTCTGTGGGTCTGCTG

GTTGCTCATTATATGGATGTTACAAACTTCATCCTGGAAAAATTTGCTACTGCCTTCATT

GTAGCAGAGCTGAGTCTCCATTGGCACTTTCTGGAAGCGCTAGGAATGTGAACGACAAGA

ACAACGAGATGGAGAACTCTCCATTGTAAGTACTCAATACAAGATTTCGTTACACAATAT

AAA-ATTTAAAAATTTGGAAAAAAAA------GTTTTCTACATGCCAACATATATTGTAA

TTAAGGTGTTTACCAATTTTTGAAACAGATAATTTGAT-TTTTTTTTAAATAAAATATTT

CATCTT-----AATTCAATATGTCGTTATCGTCTGAATCGTATTA------TGCAGTCAT

TGAAAAAAAA--TAAACACCTGAATTACTCCTGCAAGAGAAGGAATGGAT-GT-TTTGTT

CTT-AGATCGGTTAATGTGTTGGCAGGTAGATAAGATATCTTCTAGTGTGTCTCTCAATT

-ATTTACATTCAAAAACATTTTTTT----ACATAAATCGATCACTGGTCAATTGCACAAG

AATAGTCAGAAAAAGAAC--TGTTATATAAAACGTTTTAATTGAATTTGTGAAAATAA-C

ACCACATATAAGAAAACATCTACTTTGTTTCAGGATGAATGAGGTGGAAAATTTGGACCA

AGAAATGGATATGTTCTAG

>33_03Gen

ATGAAGGCAACGATCTTGTTAGCTGTTGTAGTGGCAGTCATTGTTGGAGGTAAATATCTT

TATTGAAATATATCTGTGTGTACGTATTTTAGAAGTATAGATCCAGCATTTTT-AGGATA

AATATACGGCAGACCGACTTTTCTGAAA--------------------------------

-------------ATTTTACTTCTTTATT-TATT--TTTTCCATTGA---TAATAACTGT

TTTAAGTAAGAAAG----------------------------------------------

------------------------------------------------------------

-------------------------------------------TTGTTAATGTTTACAAC

TGAAATATGTTTTGTTATTAAAATTAGATCATTCAAATATTCAAACAAATATTTTTT-CT

TTATACGTGTTGTTCAGTTCGTAGTTTTCTATGCTATGCTTTGTATACTGTTGTTTTTCT

TTTTTTT-CTCCTTTTTT-----GCCATGGCGTTGTCAGTTTACTTTCAAGTTAGGATAA

TAAATGTTCC-TGTGGTATATTT--AGCCTCTCTCTAAGACAGGCATCG---ATTTGTGT

CGAAA-CTTAAGCAATTTTATTC-A-TTTTATTTTT-CTAC------TTATTCA--GTTC

AGGAAGCCCAATCAGTAGCTTGTACATCATACTACTGTAGTAAGTTCTGTGGGTCTGCTG

GTTGCTCATTATATGGATGTTACAAACTTCATCCTGGAAAAATTTGCTACTGCCTTCATT

GTAGCAGAGCTGAGTCTCCATTGGCACTTTCTGGAAGCGCTAGGAATGTGAACGACAAGA

ACAACGAGATGGAGAACTCTCCATTGTAAGTACTCAATACAAGATTTCGTTACACAATAT

AAA-ATTTAAAAATTTGGAAAAAAAA------GTCTTCTACATGCCAACATATATTGTAA

TTAAGGTGTTTACCAATTTTTGAAACAGATAATTTGAT-TTTTTTTTAAATAAAATATTT

CATCTT-----AATTCAATATGTCGTTATCGTCTGAATCGTATTA------TGCAGTCAT

TGAAAAAAAA--TAAACACCTGAATTACTCCTGCAAGAGAAGGAATGGAT-GT-TTTGTT

-CTTAGATCGGTTAATGTGTTGGCAGGTAGATAAGATATCTTCTAGTGTGTCTCTCAATT

-ATTTACATTCAAAAACATTTTTTT----ACATAAATCGATCACTGGTCAATTGCACAAG

AATAGTCAGAAAAAAAAC--TGTTATATAAAACGTTTTAATTGAATTTGTGAAAATAA-C

ACCACATATAAGAAAACATCTACTTTGTTTCAGGATGAATGAGGTGGAAAATTTGGACCA

AGAAATGGATATGTTCTAG

>33_04Gen

ATGAAGGCAACGATCTTGTTAGCTGTTGTAGTGGCAGTCATTGTTGGAGGTAAATATCTT

TATTGAAATATATCTGTGTGTACGTATTTTAGAAGTATAGATCCAGCATTTTT-AGGATA

AATATACGGCAGACCGACTTTTCTGAAA--------------------------------

-------------ATTTTACTTCTTTATT-TATT--TTTTTCATTGA---TAATAACTGC

TTTAAGTAAGAAAG----------------------------------------------

------------------------------------------------------------

-------------------------------------------TTGTTAATGTTTACAAC

TGAAATATGTTTTGTTATTAAAATTAGATCATTCAAATATTCAAACAAATATTTTTT-CT

TTATACGTGTTGTTCAGTTCGTAGTTTTCTATGCTATGCTTTGTATACTGTTGTTTTTCT

TTTTTTT-CTCCTTTTTT-----GCCATGGCGTTGTCAGTTTACTTTCAAGTTAAGATAA

TAAATGTTCC-TGTGGTATATTT--AGCCTCTCTCTAAGACAGGCATCG---ATTTGTGT

CGAAA-CTTAAGCAATTTTATTC-A-TTTTATTTTT-CTAC------TTATTCA--GTTC

AGGAAGCCCAATCAGTAGCTTGTACATCATACTACTGTAGTAAGTTCTGTGGGTCTGCTG

GTTGCTCATTATATGGATGTTACAAACTTCATCCTGGAAAAATTTGCTACTGCCTTCATT

GTAGCAGAGCTGAGTCTCCATTGGCACTTTCTGGAAGCGCTAGGAATGTGAACGACAAGA

ACAACGAGATGGAGAACTCTCCATTGTAAGTACTCAATACAAGATTTCGTTACACAATAT

AAA-ATTTAAAAATTTGGAAAAAAAA------GTTTTCTACATGCCAACATATATTGTAA

TTAAGGTGTTTACCAATTTTTGAAACAGATAATTTGAT-TTTTTTTTAAATAAAATATTT

CATCTT-----AATTCAATATGTCGTTATCGTCTGAATCGTATTA------TGCAGTCAT

TGAAAAAAAA--TAAACACCTGAATTACTCCTGCAAGAGAAGGAATGGAT-GT-TTTGTT

CTT-AGATCGGTTAATGTGTTGGCAGGTAGATAAGATATCTTCTAGTGTGTCTCTCAATT

-ATTTACATTCAAAAACATTTTTTT----ACATAAATCGATCACTGGTCAATTGCACAAG

AATAGTCAGAAAAAGAAC--TGTTATATAAAACGTTTTAATTGAATTTGTGAAAATAA-C

ACCACATATAAGAAAACATCTACTTTGTTTCAGGATGAATGAGGTGGAAAATTTGGACCA

AGAAATGGATATGTTCTAG

>33_06Gen

ATGAAGGCAACGATCTTGTTAGCTGTTGTAGTGGCAGTCATTGTTGGAGGTAAATATCTT

TATTGAAATATATCTGTGTGTACGTATTTTAGAAGTATAGATCCAGCATTTTT-AGGATA

AATATACGGCAGACCTACTTTTCTGAAA--------------------------------

-------------ATTTTACTTCTTTATT-TATT--TTTTTCATTGA---TAATAACTGT

TTTAAGTAAGAAAG----------------------------------------------

------------------------------------------------------------

-------------------------------------------TTGTTAATGTTTACAAC

TGAAATATGTTTTGTTATTAAAATTAGATCATTCAAATATTCAAACAAATATTTTTT-CT

TTATACGTGTTGTTCAGTTCGTAGTTTTCTATGCTATGCTTTGTATACTGTTGTTTTTCT

TTTTTTT-CTCCTTTTTT-----GCCATGGCGTTGTCAGTTTACTTTCAAGTTAAGATAA

TAAATGTTCC-TGTGGTATATTT--AGCCTCTCTCTAAGACAGGCATCG---ATTTGTGT

CGAAA-CTTAAGCAATTTTATTC-A-TTTTATTTTT-CTAC------TTATTCA--GTTC

AGGAAGCCCAATCAGTAGCTTGTACATCATACTACTGTAGTAAGTTCTGTGGGTCTGCTG

GTTGCTCATTATATGGATGTTACAAACTTCATCCTGGAAAAATTTGCTACTGCCTTCATT

GTAGCAGAGCTGAGTCTCCATTGGCACTTTCTGGAAGCGCTAGGAATGTGAACGACAAGA

ACAACGAGATGGAGAACTCTCCATTGTAAGTACTCAATACAAGATTTCGTTACACAATAT

AAA-ATTTAAAAATTTGGAAAAAAAA------GTTTTCTACATGCCAACATATATTGTAA

TTAAGGTGTTTGCCAATTTTTGAAACAGATAATTTGAT-TTTTTTTTAAATAAAATATTT

CATCTT-----AATTCAATATGTCGTTATCGTCTGAATCGTATTA------TGCAGTCAT

TGAAAAAAAA--TAAACACCTGAATTACTCCTGCAAGAGAAGGAATGGAT-GT-TTTGTT

-CTTAGATCGGTTAATGTGTTGGCAGGTAGATAAGATATCTTCTAGTGTGTCTCTCAATT

-ATTTACATTCAAAAACATTTTTTT----ACATAAATCGATCACTGGTCAATTGCACAAG

AATAGTCAGAAAAAAAAC--TGTTATATAAAACGTTTTAATTGAATTTGTGAAAATAA-C

ACCACATATAAGAAAACATCTACTTTGTTTCAGGATGAATGAGGTGGAAAATTTGGACCA

AGAAATGGATATGTTCTAG

>33_08Gen

ATGAAGGCAACGATCTTGTTAGCTGTTGTAGTGGCAGTCATTGTTGGAGGTAAATATCTT

TATTGAAATATATCTGTGTGTACGTATTTTAGAAGTATAGATCCAGCATTTTT-AGGATA

AATATACGGCAGACCGACTTTTCTGAAA--------------------------------

-------------ATTTTACTTCTTTATT-TATT--TTTTTCATTGC---TAATAACTGT

TTTAAGTAAGAAAG----------------------------------------------

------------------------------------------------------------

-------------------------------------------TTGTTAATGTTTACAAC

TGAAATATGTTTTGTTATTAAAATTAGATCATTCAAATATTCAAACAAATATTTTTT-CT

TTATACGTGTTGTTCAGTTCGTAGTTTTCTATGCTATGCTTTGTATACTGTTGTTTTTCT

TTTTTTT-CTCCTTTTTT-----GCCATGGCGTTGTCAGTTTACTTTCAAGTTAAGATAA

TAAATGTTCC-TGTGGTATATTT--AGCCTCTCTCTAAGACAGGCATCG---ATTTGTGT

CGAAA-CTTAAGCAATTTTATTC-A-TTTTATTTTT-CTAC------TTATTCA--GTTC

AGGAAGCCCAATCAGTAGCTTGTACATCATACTACTGTAGTAAGTTCTGTGGGTCTGCTG

GTTGCTCATTATATGGATGTTACAAACTTCATCCTGGAAAAATTTGCTACTGCCTTCATT

GTAGCAGAGCTGAGTCTCCATTGGCACTTTCTGGAAGCGCTAGGAATGTGAACGACAAGA

ACAACGAGATGGAGAACTCTCCATTGTAAGTACTCAATACAAGATTTCGTTACACAATAT

AAA-ATTTAAAAATTTGGAAAAAAAA------GTTTTCTACATGCCAACATATATTGTAA

TTAAGGTGTTTACCAATTTTTGAAGCAGATAATTTGAT-TTTTTTTTAAATAAAATATTT

CATCTT-----AATTCAATATGTCGTTATCGTCTGAATCGTATTA------TGCAGTCAT

TGAAAAAAAA--TAAACACCTGAATTACTCCTGCAAGAGAAGGAATGGAT-GT-TTTGTT

-CTTAGATCGGTTAATGTGTTGGCAGGTAGATAAGATATCTTCTAGTGTGTCTCTCAATT

-ATTTACATTCAAAAACATTTTTTT----ACATAAATCGATCACTGGTCAATTGCACAAG

AATAGTCAGAAAAAAAAC--TGTTATATAAAACGTTTTAATTGAATTTGTGAAAATAA-C

ACCACATATAAGAAAACATCTACTTTGTTTCAGGATGAATGAGGTGGAAAATTTGGACCA

AGAAATGGATATGTTCTAG

>33_11Gen

ATGAAGGCAACGATCTTGTTAGCTGTTGTAGTGGTAGTCATTGTTGGAGGTAAATATCTT

TATTGAAATATATCTGTGTGTATGTGTTTTAGAAGTATAGATCCAGCATTTTT-AAGATA

AAAATACGGCAGACCGACTTTTCCAAAATTTGATTGCGAATCCCATATT---------TG

TATATTATGTTAAATTATACTACTTAATTGTAATAATTTTTCATTTA---TAATAACTGT

TTTAAGTAAGAAAGCTGTATTTGGCAAAACTTTTAGGAATTTTGGTCCTCAATGCTCTTC

TTTTTT-AACTTTTATGATTCGAGCGTCACTGATGAATCTTTTGTAGACGAAACGCGCGG

CTGGCGTATATTTAATTTAGTTCTGGTATCTATGATGAGTTTATTGTTAATGTTTACAAC

TGAAATATGTTTTGTTATTAAAATTAAATCACTCAAATACTCAAACAAATATTTTTT-CT

TTATACGTGTTGTTCAGTTCGTAGTTTTCTATGCTATGCTTTGTATACTGTTGTTTTTCT

TTTTTTTTCTCCTTTTGTTT---GCCATGGCGTTGTTAGTTTATTTTCAACTTATGATAA

TAAATGTCCC-TGTGGTATATTT--AGCCTCTCTCTAAGACAGGCATCGTT-ATTTGTGT

CGAAA-CTTAAGCAATTTTATTC-A-TTTCATTTTT-CTAC------TTATTCA--GTTC

AGGAAGCCCAATCAGTAGCTTGTAGATCATACTACTGTAGTAAGTTCTGTGGGTCTGCTG

GTTGCTCATTATATGGATGTTACCTACTTCATCCTGGAAAAATTTGCTACTGCCTTCATT

GTAGCAGAGCTGAGTCTCCATTGGCACTTTCTGGAAGCGCTAGGAATGTGAACGACAAGA

ACAACGAGATGGACAACTCTCCAGTGTAAGTACTCAATACTAGATTTCGTTACACAGTAT

AAAAATTTAAAAATTTGGAAAAAAAA------GTTTTCTACATTCCAACATATATTGTAA

TTAAGGTGTTTACCAATTTTTGAAACAGATAATTTAAT-TTTTTTTTAAATAAAATATTT

CATCTT-----AATTCAATATGTCGTTATCGTTTGAATCGTATTA------TGCAGTCAT

TGAAAAAAAAA-TAAACACCTGAACTCCTCCTGCAAGAGAAAGAAGGGAT-GT-TTT-TT

TCTAAGATCGGTTAATGTGGTGGGAGGCAGATGAGATATCATCTAGTGTGTCTCTCAACT

-ATTTACATTCAAAAACATTTTTTTTTT-ACATTAATCGATCACTGGTCAATTGCACGAG

AGTAGTCAGAAAAAAA-CCCTGTTATATGAAACGTTTTAATTGAATTTGTGAAAATAA-C

ACCACATATAAGAAAACATCTACTTTGTTTCAGGATGAATGAGATGGACAATTTGGACCA

AGAAATGAATATGTTCTAG

>33_21Gen

ATGAAGGCAACGATCTTGTTAGCTGTTGTAGTGGCAGTCATTGTTGGAGGTAAATATCTT

TATTGAAATATATCTGTGTGTACGTATTTTAGAAGTATAGATCCAGCATTTTT-AGGATA

AATATACGGCAGACCGACTTTTCTGAAA--------------------------------

-------------ATTTTACTTCTTTATT-TATT--TTTTTCATTGA---TAATAACTGT

TTTAAGTAAGAAAG----------------------------------------------

------------------------------------------------------------

-------------------------------------------TTGTTAATGTTTACAAC

TGAAATATGTTTTGTTATTAAAATTAGATCATTCAAATATTCAAACAAATATTTTTT-CT

TTATACGTGTTGTTCAGTTCGTAGTTTTCTATGCTATGCTTTGTATACTGTTGTTTTTCT

TTTTTTT-CTCCTTTTTT-----GCCATGGCGTTGTCAGTTTACTTTCAAGTTAAGATAA

TAAATGTTCC-TGTGGTATATTT--AGCCTCTCTCTAAGACAGGCATCG---ATTTGTGT

CGAAA-CTTAAGCAATTTTATTC-A-TTTTATTTTT-CTAC------TTATTCA--GTTC

AGGAAGCCCAATCAGTAGCTTGTACATCATACTACTGTAGTAAGTTCTGTGGGTCTGCTG

GTTGCTCATTATATGGATGTTACAAACTTCATCCTGGAAAAATTTGCTACTGCCTTCATT

GTAGCAGAGCTGAGTCTCCATTGGCACTTTCTGGAAGCGCTAGGAATGTGAACGACAAGA

ACAACGAGATGGAGAACTCTCCATTGTAAGTACTCAATACAAGATTTCGTTACACAATAT

AAA-ATTTAAAAATTTGGAAAAAAAA------GTTTTCTACATGCCAACATATATTGTAA

TTAAGGTGTTTACCAATTTTTGAAACAGATAATTTGAT-TTTTTTTTAAATAAAATATTT

CATCTT-----AATTCAATATGTCGTTATCGTCTGAATCGTATTA------TGCAGTCAT

TGAAAAAAAA--TAAACACCTGAATTACTCCTGCAAGAGAAGGAATGGAT-GT-TTTGTT

CTT-AGATCGGTTAATGTGTTGGCAGGTAGATAAGATATCTTCTAGTGTGTCTCTCAATT

-ATTTACATTCAAAAACATTTTTTT----ACATAAATCGATCACTGGTCAATTGCACAAG

AATAGTCAGAAAAAAAAC--TGTTATATAAAACGTTTTAATTGAATTTGTGAAAATAA-C

ACCACATATAAGAAAACATCTACTTTGTTTCAGGATGAATGAGGTGGAAAATTTGGACCA

AGAAATGGATATGTTCTAG

>33_22Gen

ATGAAGGCAACGATCTTGTTAGCTGTTGTAGTGGCAGTCATTGTTGGAGGTAAATATCTT

TATTGAAATATATCTGTGTGTACGTATTTTAGAAGTATAGACCCAGCATTTTT-AGGATA

AATATACGGCAGACCGACTTTTCTGAAA--------------------------------

-------------ATTTTACTTCTTTATT-TATT--TTTTTCATTGA---TAATAACTGT

TTTAAGTAAGAAAG----------------------------------------------

------------------------------------------------------------

-------------------------------------------TTGTTAATGTTTACAAC

TGAAATATGTTTTGTTATTAAAATTAGATCATTCAAATATTCAAACAAATATTTTTT-CT

TTATACGTGTTGTTCAGTTCGTAGTTTTCTATGCTATGCTTTGTATACTGTTGTTTTTCT

TTTTTTT-CTCCTTTTTT-----GCCATGGCGTTGTCAGTTTACTTTCAAGTTAAGATAA

TAAATGTTCC-TGTGGTATATTT--AGCCTCTCTCTAAGACAGGCATCG---ATTTGTGT

CGAAA-CTTAAGCAATTTTATTC-A-TTTTATTTTT-CTAC------TTATTCA--GTTC

AGGAAGCCCAATCAGTAGCTTGTACATCATACTACTGTAGTAAGTTCTGTGGGTCTGCTG

GTTGCTCATTATATGGATGTTACAAACTTCATCCTGGAAAAATTTGCTACTGCCTTCATT

GTAGCAGAGCTGAGTCCCCATTGGCACTTTCTGGAAGCGCTAGGAATGTGAACGACAAGA

ACAACGAGATGGAGAACTCTCCATTGTAAGTACTCAATACAAGATTTCGTTACACAATAT

AAA-ATTTAAAAATTTGGAAAAAAAA------GTTTTCTACATGCCAACATATATTGTAA

TTAAGGTGTTTACCAATTTTTGAAACAGATAATTTGAT-TTTTTTTTAAATAAAATATTT

CATCTT-----AATTCAATATGTCGTTATCGTCTGAATCGTATTA------TGCAGTCAT

TGAAAAAAAA--TAAACACCTGAATTACTCCTGCAAGAGAAGGAATGGAT-GT-TTTGTT

CTT-AGATCGGTTAATGTGTTGGCAGGTAGATAAGATATCTTCTAGTGTGTCTCTCAATT

-ATTTACATTCAAAAACATTTTTTT----ACATAAATCGATCACTGGTCAATTGCACAAG

AATAGTCAGAAAAAAAAC--TGTTATATAAAACGTTTTAATTGAATTTGTGAAAATAA-C

ACCACATATAAGAAAACATCTACTTTGTTTCAGGATGAATGAGGTGGAAAATTTGGACCA

AGAAATGGATATGTTCTAG

>33_23Gen

ATGAAGGCAACGACCTTGTTAGCTGTTGTAGTGGCAGTCATTGTTGGAGGTAAATATCTT

TATTGAAATATATCTGTGTGTACGTATTTTAGAAGTATAGATCCAGCATTTTT-AGGATA

AATATACGGCAGACCGACTTTTCTGAAA--------------------------------

-------------ATTTTACTTCTTTATT-TATT--TTTTTCATTGA---TAATAACTGT

TTTAAGTAAGAAAG----------------------------------------------

------------------------------------------------------------

-------------------------------------------TTGTTAATGTTTACAAC

TGAAATATGTTTTGTTATTAAAATTAGATCATTCAAATATTCAAACAAATATTTTTT-CT

TTATACGTGTTGTTCAGTTCGTAGTTTTCTATGCTATGCTTTGTATACTGTTGTTTTTCT

TTTTTTT-CTCCTTTTTT-----GCCATGGCGTTGTCAGTTTACTTTCAAGTTAAGATAA

TAAATGTTCC-TGTGGTATATTT--AGCCTCTCTCTAAGACAGGCATCG---ATTTGTGT

CGAAA-CTTAAGCAATTTTATTC-A-TTTTATTTTT-CTAC------TTATTCA--GTTC

AGGAAGCCCAATCAGTAGCTTGTACATCATACTACTGTAGTAAGTTCTGTGGGTCTGCTG

GTTGCTCATTATATGGACGTTACAAACTTCATCCTGGAAAAATTTGCTACTGCCTTCATT

GTAGCAGAGCTGAGTCTCCATTGGCACTTTCTGGAAGCGCTAGGAATGTGAACGACAAGA

ACAACGAGATGGAGAACTCTCCATTGTAAGTACTCAATACAAGATTTCGTTACACAATAT

AAA-ATTTAAAAATTTGGAAAAAAAA------GTTTTCTACATGCCAACATATATTGTAA

TTAAGGTGTTTACCAATTTTTGAAACAGATAATTTGAT-TTTTTTTTAAATAAAATATTT

CATCTT-----AATTCAATATGTCGTTATCGTCTGAATCGTATTA------TGCAGTCAT

TGAAAAAAAA--TAAACACCTGAATTACTCCTGCAAGAGAAGGAATGGAT-GT-TTTGTT

CTT-AGATCGGTTAATGTGTTGGCAGGTAGATAAGATATCTTCTAGTGTGTCTCTCAATT

-ATTTACATTCAAAAACATTTTTTT----ACATAAATCGATCACTGGTCAATTGCACAAG

AATAGTCAGAAAAAAAAC--CGTTATATAAAACGTTTTAATTGAATTTGTGAAAATAA-C

ACCACATATAAGAAAACATCTACTTTGTTTCAGGATGAATGAGGTGGAAAATTTGGACCA

AGAAATGGATATGTTCTAG

>33_52Gen

ATGAAGGCAACGATCTTGTTAGCTGTTGTAGTGGCAGTCATTGTTGGAGGTAAATATCTT

TATTGAAATATATCTGTGTGTACGTATTTTAGAAGTATAGATCCAGCATTTTT-AGGATA

AATATACGGCAGACCGACTTTTCTGAAA--------------------------------

-------------ATTTTACTTCTTTATT-TATT--TTTTTCATTGA---TAATAACTGT

TTTAAGTAAGAAAG----------------------------------------------

------------------------------------------------------------

-------------------------------------------TTGTTAATGTTTACAAC

TGAAATATGTTTTGTTATTAAAATTAGATCATTCAAATATTCAAACAAATATTTTTT-CT

TTATACGTGTTGTTCAGTTCGTAGTTTTCTATGCTATGCTTTGTATACTGTTGTTTTTCT

TTTTTTT-CTCCTTTTTT-----GCCATGGCGTTGTCAGTTTACTTTCAAGTTAAGATAA

TAAATGTTCC-TGTGGTATATTT--AGCCTCTCTCTAAGACAGGCATCG---ATTTGTGT

CGAAA-CTTAAGCAATTTTATTC-A-TCTTATTTTT-CTAC------TTATTCA--GTTC

AGGAAGCCCAATCAGTAGCTTGTACATCATACTACTGTAGTAAGTTCTGTGGGTCTGCTG

GTTGCTCATTATATGGATGTTACAAACTTCATCCTGGAAAAATTTGCTACTGCCTTCATT

GTAGCAGAGCTGAGTCTCCATTGGCACTTTCTGGAAGCGCTAGGAATGTGAACGACAAGA

ACAACGAGATGGAGAACTCTCCATTGTAAGTACTCAATACAAGATTTCGTTACACAATAT

AAA-ATTTAAAAATTTGGAAAAAAAA------GTTTTCTACATGCCAACATATATTGTAA

TTAAGGTGTTTACCAATTTTTGAAACAGATAATTTGAT-TTTTTTTTAAATAAAATATTT

CATCTT-----AATTCAATATGTCGTTATCGTCTGAATCGTATTA------TGCAGTCAT

TGAAAAAAAA--TAAACACCTGAATTACTCCTGCAAGAGAAGGAATGGAT-GT-TTTGTT

-CTTAGATCGGTTAATGTGTTGGCAGGTAGATAAGATATCTTCTAGTGTGTCTCTCAATT

-ATTTACATTCAAAAACATTTTTTT----ACATAAATCGATCACTGGTCAATTGCACAAG

AATAGTCAGAAAAAAAAC--TGTTATATAAAACGTTTTAATTGAATTTGTGAAAATAA-C

ACCACATATAAGAAAACATCTACTTTGTTTCAGGATGAATGAGGTGGAAAATTTGGACCA

AGAAATGGATATGTTCTAG

>33_01cDNA

ATGAAGGCAACGATCTTGTTAGCTGTTGTAGTGGCAGTCATTGTTGGAG-----------

------------------------------------------------------------

------------------------------------------------------------

------------------------------------------------------------

------------------------------------------------------------

------------------------------------------------------------

------------------------------------------------------------

------------------------------------------------------------

------------------------------------------------------------

------------------------------------------------------------

------------------------------------------------------------

---------------------------------------------------------TTC

AGGAAGCCCAATCAGTAGCTTGTACATCATACTACTGTAGTAAGTTCTGTGGGTCTGCTG

GTTGCTCATTATATGGATGTTACAAACTTCATCCTGGAAAAATTTGCTACTGCCTTCATT

GTAGCAGAGCTGAGTCTCCATTGGCACTTTCTGGAAGCGCTAGGAATGTGAACGACAAGA

ACAACGAGATGGAGAACTCTCCATT-----------------------------------

------------------------------------------------------------

------------------------------------------------------------

------------------------------------------------------------

------------------------------------------------------------

------------------------------------------------------------

------------------------------------------------------------

------------------------------------------------------------

---------------------------------GATGAATGAGGTGGAAAATTTGGACCA

AGAAATGGATATGTTCTAG

>33_02cDNA

ATGAAGGCAACGATCTTGTTAGCTGTTGTAGTGGCAGTCATTGTTGGAG-----------

------------------------------------------------------------

------------------------------------------------------------

------------------------------------------------------------

------------------------------------------------------------

------------------------------------------------------------

------------------------------------------------------------

------------------------------------------------------------

------------------------------------------------------------

------------------------------------------------------------

------------------------------------------------------------

---------------------------------------------------------TTC

AGGAAGCCCAATCAGTAGCTTGTACATCATACTACTGTAGTAAGTTCTGTGGGTCTGCTG

GTTGCTCATTATATGGATGTTACAAACTTCATCCTGGAAAAATTTGCTACTGCCTTCATT

GTAGCAGAGCTGAGTCTCCATTGGCACTTTCTGGAAGCGCTAGGAATGTGAACGACAAGA

ACAACGAGATGGACAACTCTCCAGT-----------------------------------

------------------------------------------------------------

------------------------------------------------------------

------------------------------------------------------------

------------------------------------------------------------

------------------------------------------------------------

------------------------------------------------------------

------------------------------------------------------------

---------------------------------GATGAATGAGATGGACAATTTGGACCA

AGAAATGAATATGTTCTAG

>33_03cDNA

ATGAAGGCAACGATCTTGTTAGCTGTTGTAGTGGCAGTCATTGTTGGAG-----------

------------------------------------------------------------

------------------------------------------------------------

------------------------------------------------------------

------------------------------------------------------------

------------------------------------------------------------

------------------------------------------------------------

------------------------------------------------------------

------------------------------------------------------------

------------------------------------------------------------

------------------------------------------------------------

---------------------------------------------------------TTC

AGGAAGCCCAATCAGTAGCTTGTACATCATACTACTGTAGTAAGTTCTGTGGGTCTGCTG

GTTGCTCATTATATGGATGTTACAAACTTCATCCTGGAAAAATTTGCTACTGCCTTCATT

GTAGCAGAGCTGAGTCTCCATTGGCACTTTCTGGAAGCGCTAGGAATGTGAACGACAAGA

ACAACGAGATGGAGAACTCTCCATT-----------------------------------

------------------------------------------------------------

------------------------------------------------------------

------------------------------------------------------------

------------------------------------------------------------

------------------------------------------------------------

------------------------------------------------------------

------------------------------------------------------------

---------------------------------GATGAATGAGGTGGAAAATTTGGACCA

AGAAATGGATATGTTCTAG

>33_05cDNA

ATGAAGGCAACGATCTTGTTAGCTGTTGTAGTGGCAGTCATTGTTGGAG-----------

------------------------------------------------------------

------------------------------------------------------------

------------------------------------------------------------

------------------------------------------------------------

------------------------------------------------------------

------------------------------------------------------------

------------------------------------------------------------

------------------------------------------------------------

------------------------------------------------------------

------------------------------------------------------------

---------------------------------------------------------TTC

AGGAAGCCCAATCAGTAGCTTGTAGATCATACTACTGTAGTAAGTTCTGTGGGTCTGCTG

GTTGCTCATTaTATGGATGTTACCTACTTCATCCTGGAAAAATTTGCTACTGCCTTCATT

GTAGCAGAGCTGAGTCTCCATTGGCACTTTCTGGAAGCGCTAGGAATGTGAACGACAAGA

ACAACGAGATGGACAACTCTCCAGT-----------------------------------

------------------------------------------------------------

------------------------------------------------------------

------------------------------------------------------------

------------------------------------------------------------

------------------------------------------------------------

------------------------------------------------------------

------------------------------------------------------------

---------------------------------GATGAATGAGATGGACAATTTGGACCA

AGAAATAAATATGTTCTAG

>33_06cDNA

ATGAAGGCAACGATCTTGTTAGCTGTTGTAGTGGCAGTCATTGTTGGAG-----------

------------------------------------------------------------

------------------------------------------------------------

------------------------------------------------------------

------------------------------------------------------------

------------------------------------------------------------

------------------------------------------------------------

------------------------------------------------------------

------------------------------------------------------------

------------------------------------------------------------

------------------------------------------------------------

---------------------------------------------------------TTC

AGGAAGCCCAATCAGTAGCTTGTACATCATACTACTGTAGTAAGTTCTGTGGGTCTGCTG

GTTGCTCATTATATGGATGTTACAAACTTCATCCTGGAAAAATTTGCTACTGCCTTCATT

GTAGCAGAGCTGAGTCTCCATTGGCACTTTCTGGAAGCGCTAGGAATGTGAACGACAAGA

ACAACGAGATGGAGAACTCTCCATT-----------------------------------

------------------------------------------------------------

------------------------------------------------------------

------------------------------------------------------------

------------------------------------------------------------

------------------------------------------------------------

------------------------------------------------------------

------------------------------------------------------------

---------------------------------GATGAATGAGGTGGAAAATTTGGACCA

AGAAATGGATATGTTCTAG

>33_07cDNA

ATGAAGGCAACGATCTTGTTAGCTGTTGTAGTGGCAGTCATTGTTGGAG-----------

------------------------------------------------------------

------------------------------------------------------------

------------------------------------------------------------

------------------------------------------------------------

------------------------------------------------------------

------------------------------------------------------------

------------------------------------------------------------

------------------------------------------------------------

------------------------------------------------------------

------------------------------------------------------------

---------------------------------------------------------TTC

AGGAAGCCCAATCAGTAGCTTGTAGATCATACTACTGTAGTAAGTTCTGTGGGTCTGCTG

GTTGCTCATTATATGGATGTTACCTACTTCATCCTGGAAAAATTTGCTACTGCCTTCATT

GTAGCAGAGCTGAGTCTCCATTGGCACTTTCTGGAAGCGCTAGGAATGTGAACGACAAGA

ACAACGAGATGGACAACTCTCCAGT-----------------------------------

------------------------------------------------------------

------------------------------------------------------------

------------------------------------------------------------

------------------------------------------------------------

------------------------------------------------------------

------------------------------------------------------------

------------------------------------------------------------

---------------------------------GATGAATGAGATGGACAATTTGGACCA

AGAAATGAATATGTTCTAG

>33_08cDNA

ATGAAGGCAACGATCTTGTTAGCTGTCGTAGTGGCAGTCATTGTTGGAG-----------

------------------------------------------------------------

------------------------------------------------------------

------------------------------------------------------------

------------------------------------------------------------

------------------------------------------------------------

------------------------------------------------------------

------------------------------------------------------------

------------------------------------------------------------

------------------------------------------------------------

------------------------------------------------------------

---------------------------------------------------------TTC

AGGAAGCCCAATCAGTAGCTTGTACATCATACTACTGTAGTAAGTTCTGTGGGTCTGCTG

GTTGCTCATTATATGGATGTTACAAACTTCATCCTGGAAAAATTTGCTACTGCCTTCATT

GTAGCAGAGCTGAGTCTCCATTGGCACTTTCTGGAAGCGCTAGGAATGTGAACGACAAGA

ACAACGAGATGGAGAACTCTCCATT-----------------------------------

------------------------------------------------------------

------------------------------------------------------------

------------------------------------------------------------

------------------------------------------------------------

------------------------------------------------------------

------------------------------------------------------------

------------------------------------------------------------

---------------------------------GATGAATGAGGTGGAAAATTTGGACCA

AGAAATGGATATGTTCTAG

>33_09cDNA

ATGAAGGCAACGATCTTGTTAGCTGTTGTAGTGGCAGTCATTGTTGGAG-----------

------------------------------------------------------------

------------------------------------------------------------

------------------------------------------------------------

------------------------------------------------------------

------------------------------------------------------------

------------------------------------------------------------

------------------------------------------------------------

------------------------------------------------------------

------------------------------------------------------------

------------------------------------------------------------

---------------------------------------------------------TTC

AGGAAGCCCAATCAGTAGCTTGTACATCATACTACTGTAGTAAGTTCTGTGGGTCTGCTG

GTTGCTCATTATATGGATGTTACAAACTTCATCCTGGAAAAATTTGCTACTGCCTTCATT

GTAGCAGAGCTGAGTCTCCATTGGCACTTTCTGGAAGCGCTAGGAATGTGAACGACAAGA

ACAACGAGATGGAGAACTCTCCATT-----------------------------------

------------------------------------------------------------

------------------------------------------------------------

------------------------------------------------------------

------------------------------------------------------------

------------------------------------------------------------

------------------------------------------------------------

------------------------------------------------------------

---------------------------------GATGAATGAGGTGGAAAATTTGGACCA

AGAAATGGATATGTTCTAG

>33_10cDNA

ATGAAGGCAACGATCTTGTTAGCTGTTGTAGTGGCAGTCATTGTTGGAG-----------

------------------------------------------------------------

------------------------------------------------------------

------------------------------------------------------------

------------------------------------------------------------

------------------------------------------------------------

------------------------------------------------------------

------------------------------------------------------------

------------------------------------------------------------

------------------------------------------------------------

------------------------------------------------------------

---------------------------------------------------------TTC

AGGAAGCCCAATCAGTAGCTTGTACATCATACTACTGTAGTAAGTTCTGTGGGTCTGCTG

GTTGCTCATTATATGGATGTTACAAACTTCATCCTGGAAAAATTTGCTACTGCCTTCATT

GTAGCAGAGCTGAGTCTCCATTGGCACTTTCTGGAAGCGCTAGGAATGTGAACGACAAGA

ACAACGAGATGGAGAACTCTCCATT-----------------------------------

------------------------------------------------------------

------------------------------------------------------------

------------------------------------------------------------

------------------------------------------------------------

------------------------------------------------------------

------------------------------------------------------------

------------------------------------------------------------

---------------------------------GATGAATGAGGTGGAAAATTTGGACCA

AGAAATGGATATGTTCTAG

>22_01cDNA

ATGAAGGCAACGATCTTGTTAGCTGTTGTAGTGGTAGTCATTGTTGGAG-----------

------------------------------------------------------------

------------------------------------------------------------

------------------------------------------------------------

------------------------------------------------------------

------------------------------------------------------------

------------------------------------------------------------

------------------------------------------------------------

------------------------------------------------------------

------------------------------------------------------------

------------------------------------------------------------

---------------------------------------------------------TTC

AGGAAGCCCAATCAATTCCTTGTACATCATACTACTGTAGTAAGTTCTGTGGGTCTGCTG

GTTGCTCATTATATGGATGTTACAAACTTCATCCCGGCAAAATTTGCTACTGCCTTCATT

GTCGCAGAGCTGAGTCTCCATTGGCACTTTCTGGAAGCGCTAGGAATGTGAACGAGCAGA

ACAAAGAGATGGACAACTCTCCAGT-----------------------------------

------------------------------------------------------------

------------------------------------------------------------

------------------------------------------------------------

------------------------------------------------------------

------------------------------------------------------------

------------------------------------------------------------

------------------------------------------------------------

---------------------------------GATGAATGAGGTGGAAAATTTGGACCA

AGAAATGGATATGTTCTAG

>22_02cDNA

ATGAAGGCAACGATCTTGTTAGCTGTTGTAGTGGTAGTCATTGTTGGAG-----------

------------------------------------------------------------

------------------------------------------------------------

------------------------------------------------------------

------------------------------------------------------------

------------------------------------------------------------

------------------------------------------------------------

------------------------------------------------------------

------------------------------------------------------------

------------------------------------------------------------

------------------------------------------------------------

---------------------------------------------------------TTC

AGGAAGCCCAATCAATTCCTTGTACATCATACTACTGTAGTAAGTTCTGTGGGTCTGCTG

GTTGCTCATTATATGGATGTTACAAACTTCATCCCGGCAAAATTTGCTACTGCCTTCATT

GTCGCAGAGCTGAGTCTCCATTGGCACTTTCTGGAAGCGCTAGGAATGTGAACGAGCAGA

ACAAAGAGATGGACAACTCTCCAGT-----------------------------------

------------------------------------------------------------

------------------------------------------------------------

------------------------------------------------------------

------------------------------------------------------------

------------------------------------------------------------

------------------------------------------------------------

------------------------------------------------------------

---------------------------------GATGAATGAGGTGGAAAATTTGGACCA

AGAAATGGATATGTTCTAG

>22_03cDNA

ATGAAGGCAACGATCTTGTTAGCTGTTGTAGTGGTAGTCATTGTTGGAG-----------

------------------------------------------------------------

------------------------------------------------------------

------------------------------------------------------------

------------------------------------------------------------

------------------------------------------------------------

------------------------------------------------------------

------------------------------------------------------------

------------------------------------------------------------

------------------------------------------------------------

------------------------------------------------------------

---------------------------------------------------------TTC

AGGAAGCCCAATCAATTCCTTGTACATCATACTACTGTAGTAAGTTCTGTGGGTCTGCTG

GTTGCTCATTATATGGATGTTACAAACTTCATCCCGGCAAAATTTGCTACTGCCTTCATT

GTCGCAGAGCTGAGTCTCCATTGGCACTTTCTGGAAGCGCTAGGAATGTGAACGAGCAGA

ACAAAGAGATGGACAACTCTCCAGT-----------------------------------

------------------------------------------------------------

------------------------------------------------------------

------------------------------------------------------------

------------------------------------------------------------

------------------------------------------------------------

------------------------------------------------------------

------------------------------------------------------------

---------------------------------GATGAATGAGGTGGAAAATTTGGACCA

AGAAATGGATATGTTCTAG

>22_04cDNA

ATGAAGGCAACGATCTTGTTAGCTGTTGTAGTGGTAGTCATTGTTGGAG-----------

------------------------------------------------------------

------------------------------------------------------------

------------------------------------------------------------

------------------------------------------------------------

------------------------------------------------------------

------------------------------------------------------------

------------------------------------------------------------

------------------------------------------------------------

------------------------------------------------------------

------------------------------------------------------------

---------------------------------------------------------TTC

AGGAAGCCCAATCAATTCCTTGTACATCATACTACTGTAGTAAGTTCTGTGGGTCTGCTG

GTTGCTCATTATATGGATGTTACAAACTTCATCCCGGCAAAATTTGCTACTGCCTTCATT

GTCGCAGAGCTGAGTCTCCATTGGCACTTTCTGGAAGCGCTAGGAATGTGAACGAGCAGA

ACAAAGAGATGGTCAACTCTCCAGT-----------------------------------

------------------------------------------------------------

------------------------------------------------------------

------------------------------------------------------------

------------------------------------------------------------

------------------------------------------------------------

------------------------------------------------------------

------------------------------------------------------------

---------------------------------GATGAATGAGATGGAAAATTTGGACCA

AGAAATGGATATGTTCTAG

>22_05cDNA

ATGAAGGCAACGATCTTGTTAGCTGTTGTAGTGGTAGTCATTGTTGGAG-----------

------------------------------------------------------------

------------------------------------------------------------

------------------------------------------------------------

------------------------------------------------------------

------------------------------------------------------------

------------------------------------------------------------

------------------------------------------------------------

------------------------------------------------------------

------------------------------------------------------------

------------------------------------------------------------

---------------------------------------------------------TTC

AGGAAGCCCAATCAATTCCTTGTACATCATACTACTGTAGTAAGTTCTGTGGGTCTGCTG

GTTGCTCATTATATGGATGTTACAAACTTCATCCCGGCAAAATTTGCTACTGCCTTCATT

GTCGCAGAGCTGAGTCTCCATTGGCACTTTCTGGAAGCGCTAGGAATGTGAACGAGCAGA

ACAAAGAGATGGACAACTCTCCAGT-----------------------------------

------------------------------------------------------------

------------------------------------------------------------

------------------------------------------------------------

------------------------------------------------------------

------------------------------------------------------------

------------------------------------------------------------

------------------------------------------------------------

---------------------------------GATGAATGAGGTGGAAAATTTGGACCA

AGAAATGGATATGTTCTAG

>22_07cDNA

ATGAAGGCAACGATCTTGTTAGCTGTTGTAGTGGTAGTCATTGTTGGAG-----------

------------------------------------------------------------

------------------------------------------------------------

------------------------------------------------------------

------------------------------------------------------------

------------------------------------------------------------

------------------------------------------------------------

------------------------------------------------------------

------------------------------------------------------------

------------------------------------------------------------

------------------------------------------------------------

---------------------------------------------------------TTC

AGGAAGCCCAATCAATTCCTTGTACATCATACTACTGTAGTAAGTTCTGTGGGTCTGCTG

GTTGCTCATTATATGGATGTTACAAACTTCATCCCGGCAAAATTTGCTACTGCCTTCATT

GTCGCAGAGCTGAGTCTCCATTGGCACTTTCTGGAAGCGCTAGGAATGTGAACGAGCAGA

ACAAAGAGATGGTCAACTCTCCAGT-----------------------------------

------------------------------------------------------------

------------------------------------------------------------

------------------------------------------------------------

------------------------------------------------------------

------------------------------------------------------------

------------------------------------------------------------

------------------------------------------------------------

---------------------------------GATGAATGAGATGGAAAATTTGGACCA

AGAAATGGATATGTTCTAG

>22_08cDNA

ATGAAGGCAACGATCTTGTTAGCTGTTGTAGTGGTAGTCATTGCTGGAG-----------

------------------------------------------------------------

------------------------------------------------------------

------------------------------------------------------------

------------------------------------------------------------

------------------------------------------------------------

------------------------------------------------------------

------------------------------------------------------------

------------------------------------------------------------

------------------------------------------------------------

------------------------------------------------------------

---------------------------------------------------------TTC

AGGAAGCCCAATCAATTCCTTGTACATCATACTACTGTAGTAAGTTCTGTGGGTCTGCTG

GTTGCTCATTATATGGATGTTACAAACTTCATCCCGGCAAAATTTGCTACTGCCTTCATT

GTCGCAGAGCTGAGTCTCCATTGGCACTTTCTGGAAGCGCTAGGAATGTGAACGAGCAGA

ACAAAGAGATGGACAACTCTCCAGT-----------------------------------

------------------------------------------------------------

------------------------------------------------------------

------------------------------------------------------------

------------------------------------------------------------

------------------------------------------------------------

------------------------------------------------------------

------------------------------------------------------------

---------------------------------GATGAATGAGGTGGAAAATTTGGACCA

AGAAATGGATATGTTCTAG

>22_09cDNA

ATGAAGGCAACGATCTTGTTAGCTGTTGTAGTGGTAGTCATTGTTGGAG-----------

------------------------------------------------------------

------------------------------------------------------------

------------------------------------------------------------

------------------------------------------------------------

------------------------------------------------------------

------------------------------------------------------------

------------------------------------------------------------

------------------------------------------------------------

------------------------------------------------------------

------------------------------------------------------------

---------------------------------------------------------TTC

AGGAAGCCCAATCAATTCCTTGTACATCATACTACTGTAGTAAGTTCTGTGGGTCTGCTG

GTTGCTCATTATATGGATGTTACAAACTTCATCCCGGCAAAATTTGCTACTGCCTTCATT

GTCGCAGAGCTGAGTCTCCATTGGCACTTTCTGGAAGCGCTAGGAATGTGAACGAGCAGA

ACAAAGAGATGGACAACTCTCCAGT-----------------------------------

------------------------------------------------------------

------------------------------------------------------------

------------------------------------------------------------

------------------------------------------------------------

------------------------------------------------------------

------------------------------------------------------------

------------------------------------------------------------

---------------------------------GATGAATGAGGTGGAAAATTTGGACCA

AGAAATGGATATGTTCTAG

>22_10cDNA

ATGAAGGCAACGATCTTGTTAGCTGTTGTAGTGGTAGTCATTGTTGGAG-----------

------------------------------------------------------------

------------------------------------------------------------

------------------------------------------------------------

------------------------------------------------------------

------------------------------------------------------------

------------------------------------------------------------

------------------------------------------------------------

------------------------------------------------------------

------------------------------------------------------------

------------------------------------------------------------

---------------------------------------------------------TTC

AGGAAGCCCAATCAATTCCTTGTACATCATACTACTGTAGTAAGTTCTGTGGGTCTGCTG

GTTGCTCATTATATGGATGTTACAAACTTCATCCCGGCAAAATTTGCTACTGCTTTCATT

GTCGCAGAGCTGAGTCTCCATTGGCACTTTCTGGAAGCGCTAGGAATGTGAACGAGCAGA

ACAAAGAGATGGTCAACTCTCCAGT-----------------------------------

------------------------------------------------------------

------------------------------------------------------------

------------------------------------------------------------

------------------------------------------------------------

------------------------------------------------------------

------------------------------------------------------------

------------------------------------------------------------

---------------------------------GATGAATGAGATGGAAAATTTGGACCA

AGAAATGGATATGTTCTAG

>30_01cDNA

ATGAAGGCAACGATCTTGTTAGCTGTTGTAGTGGTAGTCATTGTTGGAG-----------

------------------------------------------------------------

------------------------------------------------------------

------------------------------------------------------------

------------------------------------------------------------

------------------------------------------------------------

------------------------------------------------------------

------------------------------------------------------------

------------------------------------------------------------

------------------------------------------------------------

------------------------------------------------------------

---------------------------------------------------------TTC

AGGAAGCCCAATCAATTCCTTGTACATCATACTACTGTAGTAAGTTCTGTGGGTCTGCTG

GTTGCTCATTATATGGATGTTACAAACTTCATCCCGGCAAAATTTGCTACTGCCTTCATT

GTCGCAGAGCTGAGTCTCCATTGGCACTTTCTGGAAGCGCTAGGAATGTGAACGAGCAGA

ACAAAGAGATGGTCAACTCTCCAGT-----------------------------------

------------------------------------------------------------

------------------------------------------------------------

------------------------------------------------------------

------------------------------------------------------------

------------------------------------------------------------

------------------------------------------------------------

------------------------------------------------------------

---------------------------------GATGAATGAGGTGGAAAATTTGGACCA

AGAAATGGATATGTTCTAG

>30_02cDNA

ATGAAGGCAACGATCTTGTTAGCTGTTGTAGTGGTAGTCATTGTTGGAG-----------

------------------------------------------------------------

------------------------------------------------------------

------------------------------------------------------------

------------------------------------------------------------

------------------------------------------------------------

------------------------------------------------------------

------------------------------------------------------------

------------------------------------------------------------

------------------------------------------------------------

------------------------------------------------------------

---------------------------------------------------------TTC

AGGAAGCCCAATCAATTCCTTGTACATCATACTACTGTAGTAAGTTCTGTGGGTCTGCTG

GTTGCTCATTATATGGATGTTACAAACTTCATCCCGGCAAAATTTGCTACTGCCTTCATT

GTCGCAGAGCTGAGTCTCCATTGGCACTTTCTGGAAGCGCTAGGAATGTGAACGAGCAGA

ACAAAGAGATGGTCAACTCTCCAGT-----------------------------------

------------------------------------------------------------

------------------------------------------------------------

------------------------------------------------------------

------------------------------------------------------------

------------------------------------------------------------

------------------------------------------------------------

------------------------------------------------------------

---------------------------------GATGAATGAGGTGGAAAATTTGGACCA

AGAAATGGATATGTTCTAG

>30_03cDNA

ATGAAGGTAACTATCTTGTTAGCTGTTGTAGTGGCAGTCATTGTTGGAG-----------

------------------------------------------------------------

------------------------------------------------------------

------------------------------------------------------------

------------------------------------------------------------

------------------------------------------------------------

------------------------------------------------------------

------------------------------------------------------------

------------------------------------------------------------

------------------------------------------------------------

------------------------------------------------------------

---------------------------------------------------------TTC

AGGAAGTCCAATCAGTACCTTGTGCATCAACCTGGTGTAGTAGGTTCTGTGGGTCTGCTG

GTTGCAGATTATATGGATGTTACAGACTTCATCCCGGCAAAATTTGCTACTGCCTTCATT

GTAGCAGAGCTGAGTCTCCATTGGCACTTTCTGGAAGCGCTAGGAATGTGAACGAGCAGA

ACAAAGAGATGGACAACTCTCCAAT-----------------------------------

------------------------------------------------------------

------------------------------------------------------------

------------------------------------------------------------

------------------------------------------------------------

------------------------------------------------------------

------------------------------------------------------------

------------------------------------------------------------

---------------------------------GATGAATGAGGTGGAAAATTTGGACCA

AGAAATGAATATGTTCTAG

>30_04cDNA

ATGAAGGCAACGATCTTGTTAGCTGTTGTAGTGGTAGTCATTGTTGGAG-----------

------------------------------------------------------------

------------------------------------------------------------

------------------------------------------------------------

------------------------------------------------------------

------------------------------------------------------------

------------------------------------------------------------

------------------------------------------------------------

------------------------------------------------------------

------------------------------------------------------------

------------------------------------------------------------

---------------------------------------------------------TTC

AGGAAGCCCAATCAATTCCTTGTACATCATACTACTGTAGTAAGTTCTGTGGGTCTGCTG

GTTGCTCATTATATGGATGTTACAAACTTCATCCCGGCAAAATTTGCTACTGCCTTCATT

GTCGCAGAGCTGAGTCTCCATTGGCACTTTCTGGAAGCGCTAGGAATGTGAACGAGCAGA

ACAAAGAGATGGTCAACTCTCCAGT-----------------------------------

------------------------------------------------------------

------------------------------------------------------------

------------------------------------------------------------

------------------------------------------------------------

------------------------------------------------------------

------------------------------------------------------------

------------------------------------------------------------

---------------------------------GATGAATGAGGTGGAAAATTTGGACCA

AGAAATGGATATGTTCTAG

>30_05cDNA

ATGAAGGCAACGATCTTGTTAGCTGTTGTAGTGGTAGTCATTGTTGGAG-----------

------------------------------------------------------------

------------------------------------------------------------

------------------------------------------------------------

------------------------------------------------------------

------------------------------------------------------------

------------------------------------------------------------

------------------------------------------------------------

------------------------------------------------------------

------------------------------------------------------------

------------------------------------------------------------

---------------------------------------------------------TTC

AGGAAGCCCAATCAATTCCTTGTACATCATACTACTGTAGTAAGTTCTGTGGGTCTGCTG

GTTGCTCATTATATGGATGTTACAAACTTCATCCCGGCAAAATTTGCTACTGCCTTCATT

GTCGCAGAGCTGAGTCTCCATTGGCACTTTCTGGAAGCCCTAGGAATGTGAACGAGCAGA

ACAAAGAGATGGTCAACTCTCCAGT-----------------------------------

------------------------------------------------------------

------------------------------------------------------------

------------------------------------------------------------

------------------------------------------------------------

------------------------------------------------------------

------------------------------------------------------------

------------------------------------------------------------

---------------------------------GATGAATGAGGTGGAAAATTTGGACCA

AGAAATGGATATGTTCTAG

>30_06cDNA

ATGAAGGCAACGATCTTGTTAGCTGTTGTAGTGGTAGTCATTGTTGGAG-----------

------------------------------------------------------------

------------------------------------------------------------

------------------------------------------------------------

------------------------------------------------------------

------------------------------------------------------------

------------------------------------------------------------

------------------------------------------------------------

------------------------------------------------------------

------------------------------------------------------------

------------------------------------------------------------

---------------------------------------------------------TTC

AGGAAGCCCAATCAATTCCTTGTACATCATACTACTGTAGTAAGTTCTGTGGGTCTGCTG

GTTGCTCATTATATGGATGTTACAAACTTCATCCCGGCAAAATTTGCTACCGCCTTCATT

GTCGCAGAGCTGAGTCTCCATTGGCACTTTCTGGAAGCGCTAGGAATGTGAACGAGCAGA

ACAAAGAGATGGTCAACTCTCCAGT-----------------------------------

------------------------------------------------------------

------------------------------------------------------------

------------------------------------------------------------

------------------------------------------------------------

------------------------------------------------------------

------------------------------------------------------------

------------------------------------------------------------

---------------------------------GATGAATGAGGTGGAAAATTTGGACCA

AGAAATGGATATGTTCTAG

>30_07cDNA

ATGAAGGCAACGATCTTGTTAGCTGTTGTAGTGGTAGTCATTGTTGGAG-----------

------------------------------------------------------------

------------------------------------------------------------

------------------------------------------------------------

------------------------------------------------------------

------------------------------------------------------------

------------------------------------------------------------

------------------------------------------------------------

------------------------------------------------------------

------------------------------------------------------------

------------------------------------------------------------

---------------------------------------------------------TTC

AGGAAGCCCAATCAATTCCTTGTACATCATACTACTGTAGTAAGTTCTGTGGGTCTGCTG

GTTGCTCATTATATGGATGTTACAAACTTCATCCCGGCAAAATTTGCTACTGCCTTCATT

GTCGCAGAGCTGAGTCTCCATTGGCACTTTCTGGAAGCGCTAGGAATGTGAACGAGCAGA

ACAAAGAGATGGTCAACTCTCCAGT-----------------------------------

------------------------------------------------------------

------------------------------------------------------------

------------------------------------------------------------

------------------------------------------------------------

------------------------------------------------------------

------------------------------------------------------------

------------------------------------------------------------

---------------------------------GATGAATGAGGTGGAAAATTTGGACCA

AGAAATGGATATGTTCTAG

>30_08cDNA

ATGAAGGCAACGATCTTGTTAGCTGTTGTAGTGGTAGTCATTGTTGGAG-----------

------------------------------------------------------------

------------------------------------------------------------

------------------------------------------------------------

------------------------------------------------------------

------------------------------------------------------------

------------------------------------------------------------

------------------------------------------------------------

------------------------------------------------------------

------------------------------------------------------------

------------------------------------------------------------

---------------------------------------------------------TTC

AGGAAGCCCAATCAATTCCTTGTACATCATACTACTGTAGTAAGTTCTGTGGGTCTGCTG

GTTGCTCATTATATGGATGTTACAAACTTCATCCCGGCAAAATTTGCTACTGCCTTCATT

GTCGCAGAGCTGAGTCTCCATTGGCACTTTCTGGAAGCGCTAGGAATGTGAACGAGCAGA

ACAAAGAGATGGTCAACTCTCCAGT-----------------------------------

------------------------------------------------------------

------------------------------------------------------------

------------------------------------------------------------

------------------------------------------------------------

------------------------------------------------------------

------------------------------------------------------------

------------------------------------------------------------

---------------------------------GATGAATGAGGTGGAAAATTTGGACCA

AGAAATGGATATGTTCTAG

>30_09cDNA

ATGAAGGTAACTATCTTGTTAGCTGTTGTAGTGGCAGTCATTGTTGGAG-----------

------------------------------------------------------------

------------------------------------------------------------

------------------------------------------------------------

------------------------------------------------------------

------------------------------------------------------------

------------------------------------------------------------

------------------------------------------------------------

------------------------------------------------------------

------------------------------------------------------------

------------------------------------------------------------

---------------------------------------------------------TTC

AGGAAGTCCAATCAGTACCTTGTGCATCAACCTGGTGTAGTAGGTTCTGTGGGTCTGCTG

GTTGCAGATTATATGGATGTTACAGACTTCATTCCGGCAAAATTTGCTACTGCCTTCATT

GTCGCAGAGCTGAGTCTCCATTGGCACTTTCTGGAAGCGCTAGGAATGTGAACGAGCAGA

ACAAAGAGATGGTCAACTCTCCAGT-----------------------------------

------------------------------------------------------------

------------------------------------------------------------

------------------------------------------------------------

------------------------------------------------------------

------------------------------------------------------------

------------------------------------------------------------

------------------------------------------------------------

---------------------------------GGTGAATGAGGTGGAAAATTTGGACCA

AGAAATGGATATGTTCTAG

>30_10cDNA

ATGAAGGCAACGATCTTGTTAGCTGTTGTAGTGGTAGTCATTGTTGGAG-----------

------------------------------------------------------------

------------------------------------------------------------

------------------------------------------------------------

------------------------------------------------------------

------------------------------------------------------------

------------------------------------------------------------

------------------------------------------------------------

------------------------------------------------------------

------------------------------------------------------------

------------------------------------------------------------

---------------------------------------------------------TTC

AGGAAGCCCAATCAATTCCTTGTACATCATACTACTGTAGTAAGTTCTGTGGGTCTGCTG

GTTGCTCATTATATGGATGTTACAAACTTCATCCCGGCAAAATTTGCTACTGCCTTCATT

GTCGCAGAGCTGAGTCTCCATTGGCACTTTCTGGAAGCGCTAGGAATGTGAACGAGCAGA

ACAAAGAGATGGTCAACTCTCCAGT-----------------------------------

------------------------------------------------------------

------------------------------------------------------------

------------------------------------------------------------

------------------------------------------------------------

------------------------------------------------------------

------------------------------------------------------------

------------------------------------------------------------

---------------------------------GATGAATGAGGTGGAAAATTTGGACCA

AGAAATGGATATGTTCTAG

>myticin_C_CDS1

ATGAAGGCAACGATCTTGTTAGCTGTTGTAGTGGCAGTCATTGTTGGAG-----------

------------------------------------------------------------

------------------------------------------------------------

------------------------------------------------------------

------------------------------------------------------------

------------------------------------------------------------

------------------------------------------------------------

------------------------------------------------------------

------------------------------------------------------------

------------------------------------------------------------

------------------------------------------------------------

------------------------------------------------------------

------------------------------------------------------------

------------------------------------------------------------

------------------------------------------------------------

------------------------------------------------------------

------------------------------------------------------------

------------------------------------------------------------

------------------------------------------------------------

------------------------------------------------------------

------------------------------------------------------------

------------------------------------------------------------

------------------------------------------------------------

------------------------------------------------------------

-------------------

>myticin_C_CDS2

------------------------------------------------------------

------------------------------------------------------------

------------------------------------------------------------

------------------------------------------------------------

------------------------------------------------------------

------------------------------------------------------------

------------------------------------------------------------

------------------------------------------------------------

------------------------------------------------------------

------------------------------------------------------------

------------------------------------------------------------

---------------------------------------------------------TTC

AGGAAGCCCAATCAGTAGCTTGTAGATCATACTACTGTAGTAAGTTCTGTGGGTCTGCTG

GTTGCTCATTATATGGATGTTACCTACTTCATCCTGGAAAAATTTGCTACTGCCTTCATT

GTAGCAGAGCTGAGTCTCCATTGGCACTTTCTGGAAGCGCTAGGAATGTGAACGACAAGA

ACAACGAGATGGACAACTCTCCAGT-----------------------------------

------------------------------------------------------------

------------------------------------------------------------

------------------------------------------------------------

------------------------------------------------------------

------------------------------------------------------------

------------------------------------------------------------

------------------------------------------------------------

------------------------------------------------------------

-------------------

>myticin_C_CDS3

------------------------------------------------------------

------------------------------------------------------------

------------------------------------------------------------

------------------------------------------------------------

------------------------------------------------------------

------------------------------------------------------------

------------------------------------------------------------

------------------------------------------------------------

------------------------------------------------------------

------------------------------------------------------------

------------------------------------------------------------

------------------------------------------------------------

------------------------------------------------------------

------------------------------------------------------------

------------------------------------------------------------

------------------------------------------------------------

------------------------------------------------------------

------------------------------------------------------------

------------------------------------------------------------

------------------------------------------------------------

------------------------------------------------------------

------------------------------------------------------------

------------------------------------------------------------

---------------------------------GATGAATGAGATGGAACATTTGGACCA

AGAAATGGAAATGTTCTAG
