# Supplementary material for: Genomic Organization, Molecular Diversification, and Evolution of Antimicrobial Peptide Myticin-C Genes in the Mussel (Mytilus galloprovincialis)
Source: PLoS One. 2011 Aug 31;6(8):e24041. doi: 10.1371/journal.pone.0024041 (PMC3164099; doi:10.1371/journal.pone.0024041)
Supplement: Figure S2 — Variable positions of myticin-C cDNA in the 21 different basic sequences detected. The sequence AM497977 from Genebank was included for reference in the analysis. (DOC) [file pone.0024041.s002.doc]

**Figure S2.**

**Variable sites**

1111111111 1112222222 22222223

113455667 7778888999 0011122334 5772233445 56777990

8265849790 2672367125 7808912677 0578907565 68034240

Miticin-C_cDNA CGTCAGCGAG TACTAACAGA CTCTCTTCTT AACCAACACA GAGAAGAC

21_09cDNA .......... ..G....... .......... .......... .G......

16_01cDNA .......... ..G....... .......... .......... .G...AT.

33_07cDNA .......... ..G....... .......... .......... ...C.AT.

31_02cDNA .......... .......... .......... .......... ....CAT.

11_07cDNA .......... .......... .......... CC...G.... ......T.

17_06cDNA .......... .......... .......... CC........ ......T.

13_01cDNA .......... .......... .......... CC........ .G....T.

13_06cDNA .......... G......... .......... CC........ .G....T.

17_10cDNA .......... .......... .......AA. ........G. T..C.AT.

33_06cDNA .......... .......... .......AA. ........G. TG....T.

14_06cDNA ..G.G..ATC .......... .......AAC CC.GC.A... ..A..AT.

31_10cDNA ..G.G..ATC .......... .......AAC CC.GC.A... ......T.

11_03cDNA .......ATC .......... .......AAC CC.GC.A... .G....TT

14_10cDNA ...T...ATC .......... TAG....AAC CC.GC.A... .G.C..T.

22_01cDNA ...T...ATC .......... .......AAC CC.GC.A... .G....T.

30_10cDNA ...T...ATC .......... .......AAC CC.GC.AT.. .G....T.

16_02cDNA ...T...ATC .......... .......AAC CC.GC.AT.. ........

22_04cDNA ...T...ATC .......... .......AAC CC.GC.AT.. ......T.

11_04cDNA .....AT..C .G.AC..GCG .....AAAGC C.AGC.A..C A...C.T.

21_10cDNA ......T..C .G.ACTG..G ...AG..AGC CC.TC.A... A...C.T.

30_03cDNA TT....T..C .G.ACGG..G ...AG..AGC C..GC.A... AG...AT.
